# A One-Pot Divergent Sequence to Pyrazole and Quinoline Derivatives

**DOI:** 10.3390/molecules25092160

**Published:** 2020-05-05

**Authors:** Guido Gambacorta, David C. Apperley, Ian R. Baxendale

**Affiliations:** Department of Chemistry, University of Durham, South Road Durham DH1 3LE, UK; guido.gambacorta2@durham.ac.uk (G.C.); d.c.apperley@durham.ac.uk (D.C.A.)

**Keywords:** pyrazole, oxindole, isatin, Pfitzinger reaction, Knorr pyrazole synthesis, quinoline, ^15^N-MASNMR

## Abstract

The hydroxy-pyrazole and 3-hydroxy-oxindole motifs have been utilised in several pharma and agrochemical leads but are distinctly underrepresented in the scientific literature due to the limited routes of preparation. We have developed a one-pot procedure for their synthesis starting from simple isatins. The method employs cheap and easy-to-handle building blocks and allows easy isolation.

## 1. Introduction

**Figure 1:**
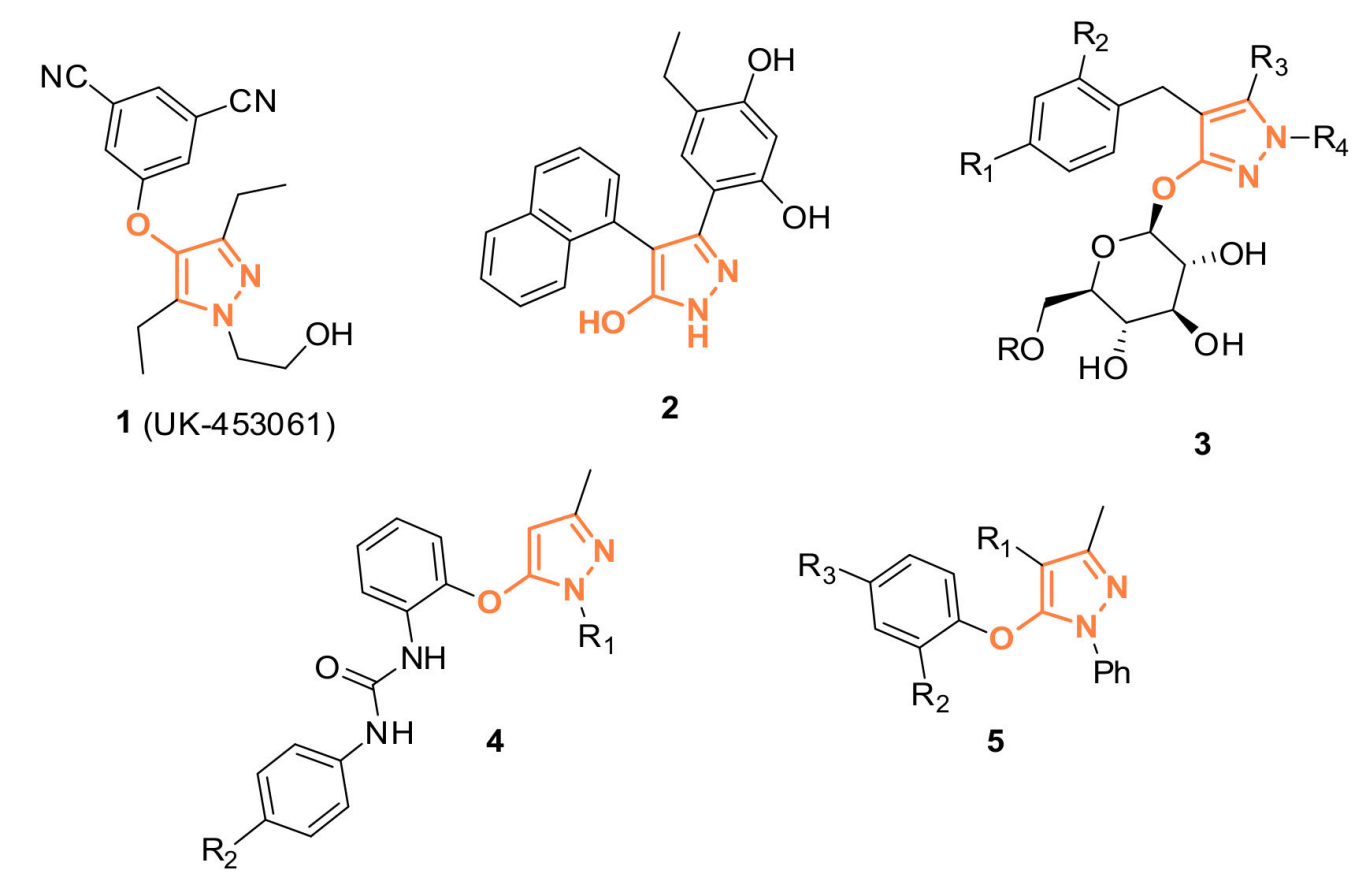
Examples of hydroxy-pyrazole derivatives described in literature.

The pyrazole moiety is commonly found in several pharmaceuticals and agrochemicals [1]. In particular, hydroxy-pyrazole units are described as antiviral (HIV-1 non-nucleoside reverse transcriptase inhibitors, **1**) [2], anti-diabetic (Na-glucose cotransporter inhibitors, **3**) [3,4,5], anti-cancer (Hsp90 inhibitor, **2**) [6], anti-thrombotic (P2Y_1_ antagonist **4**) [7], and antibacterial agents (**5**) [8,9] (examples in Figure 1).

**Figure 2:**
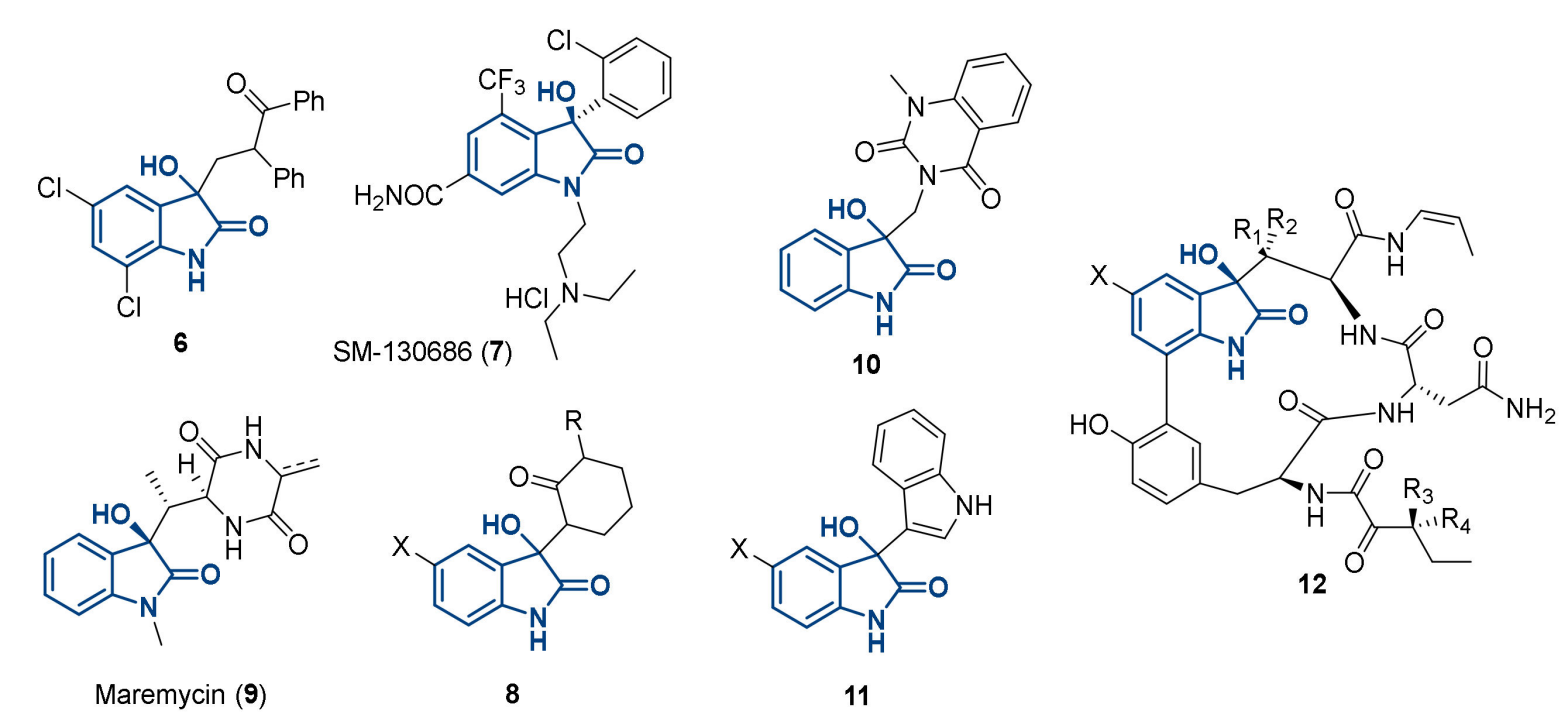
Examples of 3-hydroxy-2-oxindoles derivatives described in literature.

The 3-hydroxy-2-oxindole moiety is another related and widely represented motif present in several natural products (**9**,**10**,**12**) [10,11,12,13,14]. This scaffold has recently been employed in several areas of pharmacology (examples in Figure 2), and it has been described in the context of anti-cancer (tryptophan 2,3-dioxygenase inhibitor, **6**) [15], anticonvulsant (**8**) [16], cytotoxic (**11**) [17] and growth hormone releasing agents (i.e., SM-130686, **7**) [18,19].

Several methodologies for the generation of oxindole systems have been reported with one of these employing isatin [20]. Isatin (**13**) is an endogenous compound whose structure appears many times in natural products which have wide-ranging pharmacological activities. It has thus been widely utilised as a starting material in the preparations of a range of drug candidates [21].

**Scheme 1:**
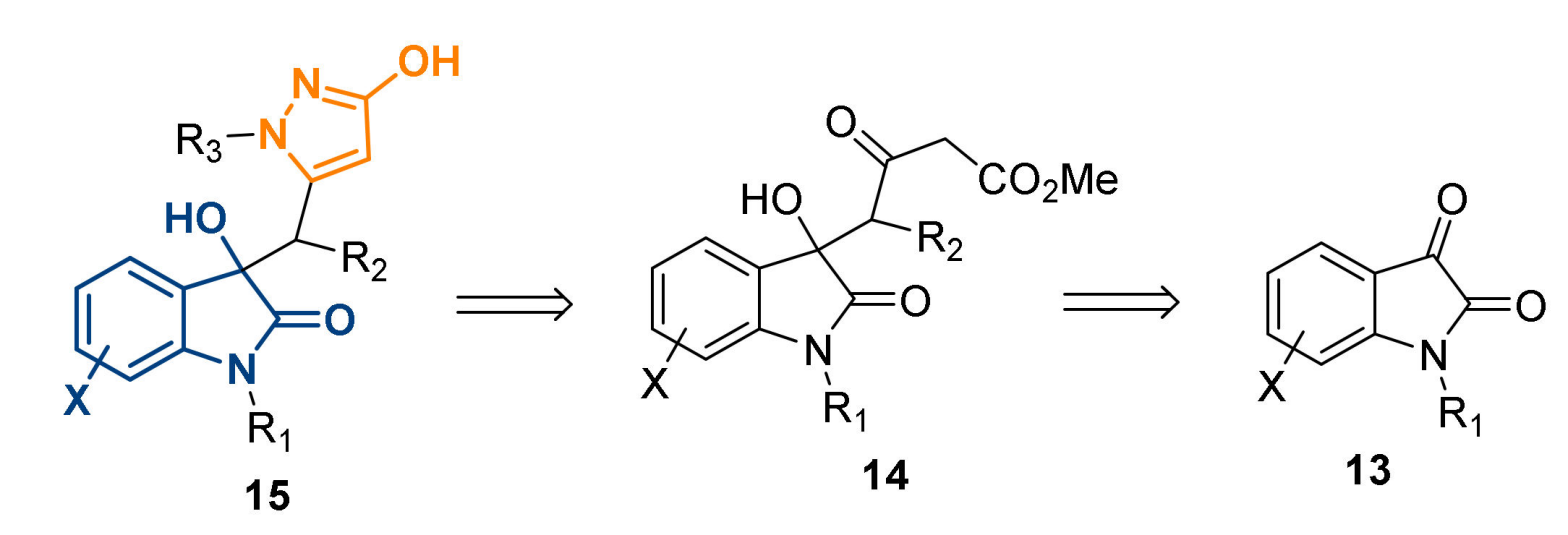
Proposed retrosynthetic synthesis of compound **15** starting from isatin (**13**).

Our research aim was to devise and validate a simple preparation comprising of the above cited pharmacophores, 3-hydroxy-pyrazole and 3-hydroxy-2-oxindole, based upon presenting the cores in a unique 3D architecture which could be easily tailored to further any medicinal chemistry exploration. To the best of our knowledge, structures such as compound **15** have not previously been made, and therefore, it would be of great interest for exploring these new areas of chemical space (Scheme 1).

## 2. Results and Discussion

### 2.1. Optimisation of the Synthesis Method

**Scheme 2:**
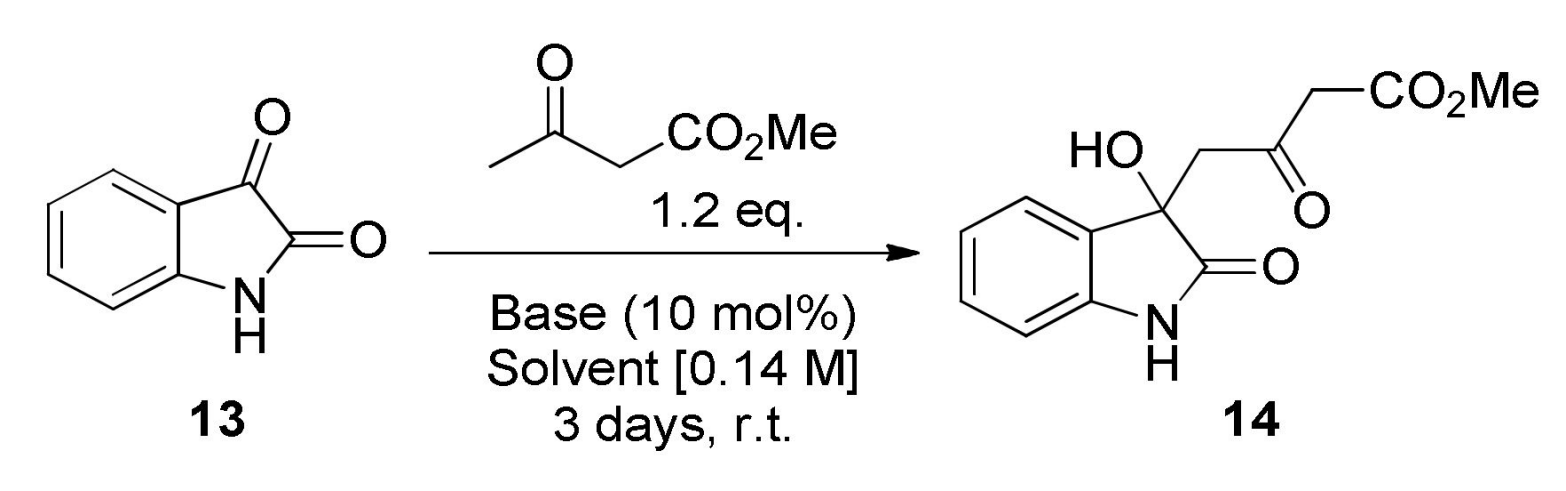
General conditions applied for the preparation of **14**.

To achieve this aim we first investigated a base-catalysed aldol reaction as described by Liu et al. [22]. We decided to screen different reaction conditions, including evaluation of bases, in order to optimise the reaction for isatin **13** (Scheme 2 and Table 1).

Piperidine was found to be the most effective catalyst allowing the desired compound to be obtained in 87% isolated yield (*Entry 8*). Whilst pursuing this work, Zhang et al. [23] reported the use of 1,8-diazabicyclo(5.4.0)undece-7-ene (DBU) in tetrahydrofuran (THF) as the preferred solvent for such a reaction. We had previously attempted to use DBU in MeOH, which had been disappointing (*Entry 2*). Unfortunately, changing the solvent to THF, although consuming the isatin, generated the desired product in only a modest 46% isolated yield (*Entry 3*). The by-products were difficult to isolate and were found unstable. Therefore, progressing with our catalyst of choice, piperidine, we investigated an expanded range of solvents. Additionally, inspired by the effect of water on 1,6-Michael additions as described by Kashinath et al., we also investigated water. However, for this reaction only a 15% conversion was detected, probably due to the low solubility of the starting materials in the aqueous media (*Entry 11*) [24]. No mixture with water was screened. Overall, MeOH still remained the best solvent; nonetheless, less polar solvents such as AcOEt and *i*PrOH also gave promising results (*Entries 10 and 15*). Attempts to avoid chromatography, by isolating and purifying the product by recrystallization, failed. Having determined effective conditions for the preparation of intermediate **14**, we next examined the pyrazole formation using different hydrazine sources starting with hydrazine monohydrate.

**Scheme 3:**
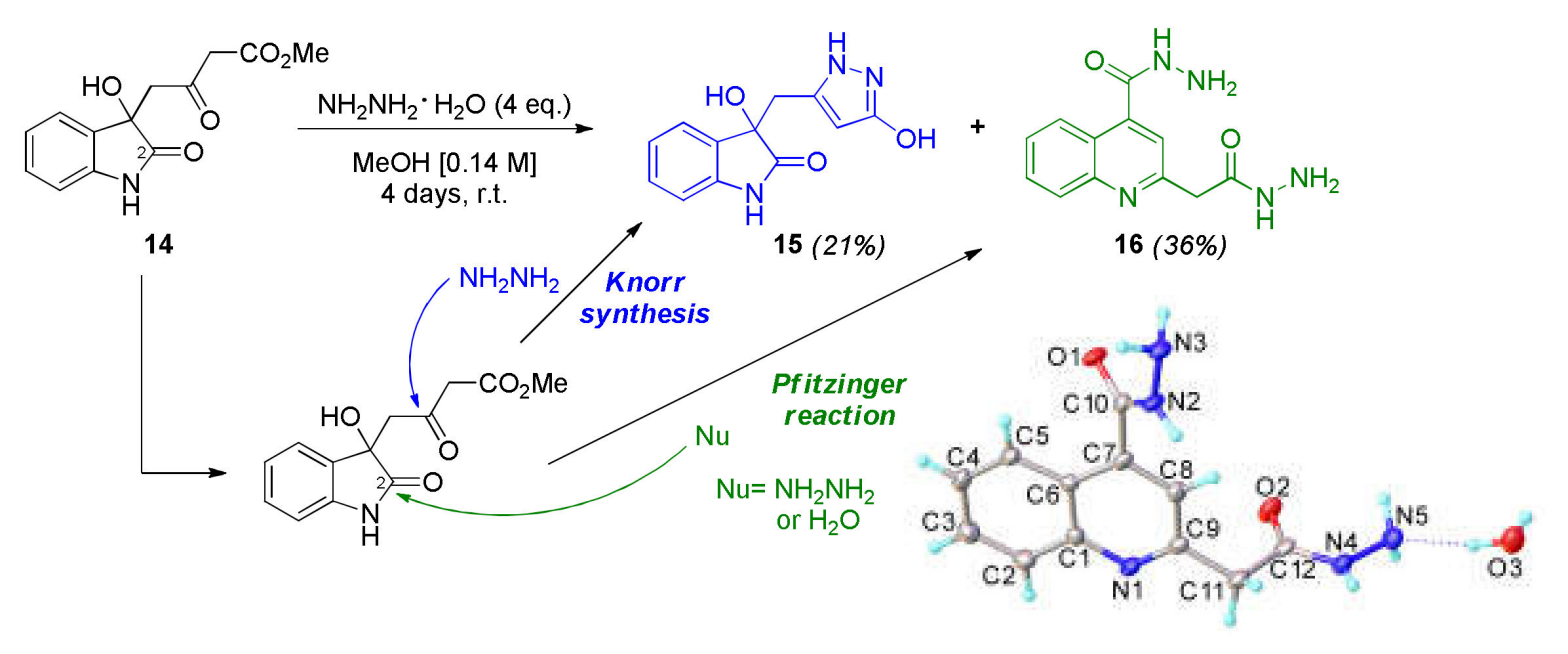
Initial reaction conditions employed for the preparation of **15**. When hydrazine attacks the carbonyl, the pyrazole **15** is formed (Knorr synthesis), whereas the addition of water or hydrazine into the amide group (marked position 2) triggers the Pfitzinger reaction. The X-ray structure of product **16** is shown in the bottom right of the picture.

To facilitate the transformation, intermediate **14** was mixed with excess hydrazine monohydrate and stirred at ambient temperature (Scheme 3). After 4 days, a precipitate was formed, which was isolated and identified as the quinoline **16**, which is formed via a Pfitzinger type reaction [25,26], occurring via either water or hydrazine attack on the position 2 of the oxindole intermediate **14** (green route, Scheme 3).

From this initial result we also investigated other hydrazine sources (Table 2). As our ultimate challenge was to develop a one-pot process, we examined the second step as a telescoped transformation of **13** to **15**. Although the THF solution of hydrazine gave good results (*Entry 3*), we elected for safety considerations to keep using hydrazine monohydrate, as it is more stable, much cheaper and more widely available [27]. The better outcomes obtained from the hydrazine solution may be attributable to a dilution effect as diluted hydrazine is added in the mixture.

As Table 3 shows, the conversion to the oxindole **15** increased, and the quantity of quinoline **16** halved when the mixture was only reacted for 2 h (*Entry 2*). We later discovered that compound **15** completely decomposed after 10 days under the reaction conditions, whereas compound **16** was stable. We showed that the formation of compound **15** is partially reversible under the reaction conditions (**15**→**14**), and it cannot be attributed to the solvent. Consequently, compound **16** acts as a thermodynamic sink, and over time, its proportion increases in the reaction mixture. Overall, our best results were obtained when the hydrazine was slowly added to the mixture, with the desired material **15** being isolated via reverse-phase column chromatography in 38% yield (Table 3, *entry 3*).

Having established a set of optimised conditions, we decided to screen other isatin derivatives and examine the different reactivity (Table 4). Despite optimisation, the purification step of the compounds **15a**–**j** and **16a**–**j** remained challenging in some cases. Consequently, we managed to isolate the compounds **15a**–**j**, but not all of the corresponding quinoline **16** derivatives could be isolated cleanly. Attempts of crystallisations with different solvents (MeOH, EtOH, *i*PrOH, H_2_O, Et_2_O, THF, Toluene) brought about sticky materials as they incorporate solvent. The 5-, 6- and 7-chloro derivatives (respectively, **15d**, **15g** and **15h**) were isolated in slightly better yields compared to **15** (*Entries 4*, *7* and *8*). However, the highest conversions of **16** are detected when the 5-chloro and 5-fluoro substrates are used, attributed to the electronic effect of an electro-withdrawing group in *para* to the amide. This is consistent with the fact that an electro-donating group such as a methoxy in the 5-position reduces the quinoline formation (*Entry 5*). A similar electronic effect is also noticed when the chlorine is in the position 7, although the fluorine and the bulkier trifluoromethyl group do still form traces of the quinoline (*Entries 8*–*10*).

When 4-fluoroisatin (**13a**) was exploited in the preparation method (*Entry 1*), the reaction occurred in very low conversions (only 30% of the isatin reacts). This outcome is attributed to the low electrophilicity of the isatin ketone which hampers the first step from occurring. This hypothesis is supported up by the ^13^C chemical shift of the carbonyl acquired in solid state (^13^C δ 179.15 ppm) compared with isatin **13** (^13^C δ 184.39 ppm), and a resultant higher stretching frequency of the same carbonyl (1739 cm^−1^ vs. 1721 cm^−1^) in Fourier transform infrared (FT-IR) spectra (see supporting materials). Indeed, substrate **13a** shows only poor conversion to the corresponding aldol adduct **14a**.

**Figure 3:**
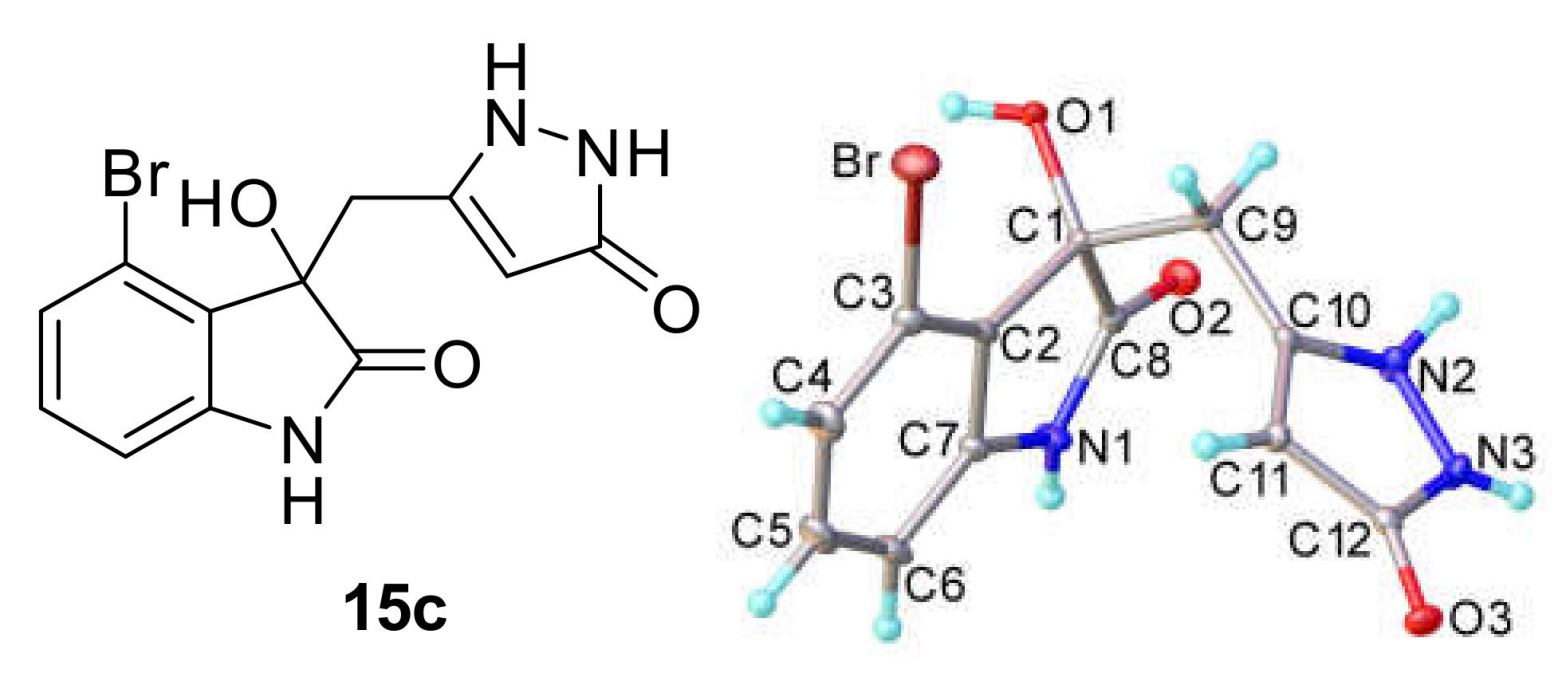
X-ray structure of the compound **15c** where the pyrazole is in its keto form.

To our delight, when other halogens in position 4 were tested, the pyrazoles (**15b**–**c**) were isolated in high yields (*Entries 2 and 3*). We suggested a Thorpe–Ingold type effect due to the bulk of the halogen group, which lowers the ring opening of the oxindole ring and accelerates the pyrazole cyclization. This could be confirmed by the raise in conversions with increasing halogen size (from chloro to bromo). Compound **15c** was crystallized and the structure of the crystal was resolved (Figure 3).

**Scheme 4:**
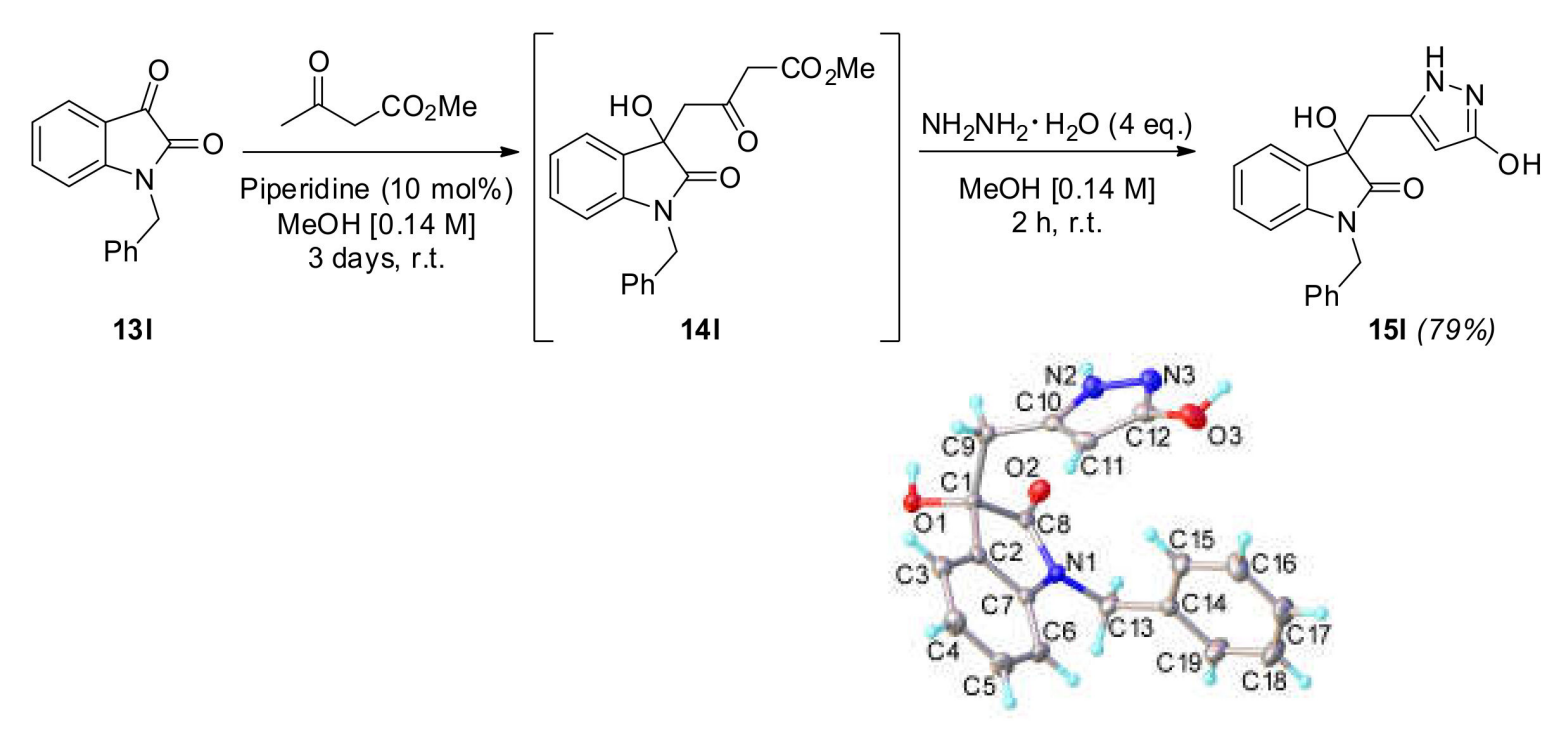
Methodology applied to the *N*-benzyl-protected isatin **13l**. The X-ray structure of **15l** is also pictured, where the pyrazole is in its enol form.

In an attempt to reduce the competitive quinoline formation (compound **16**) by making the nitrogen a worse leaving group, we *N-*benzyl-protected the nitrogen (Scheme 4). To our delight, the corresponding compound **15l** was isolated in a superior yield (79%). This compound was also highly crystalline, and an X-ray structure was also obtained to validate the molecular connectivity.

### 2.2. Observations of the Spectroscopic Data Obtained Illustrated for Compound ***15***

**Figure 4:**
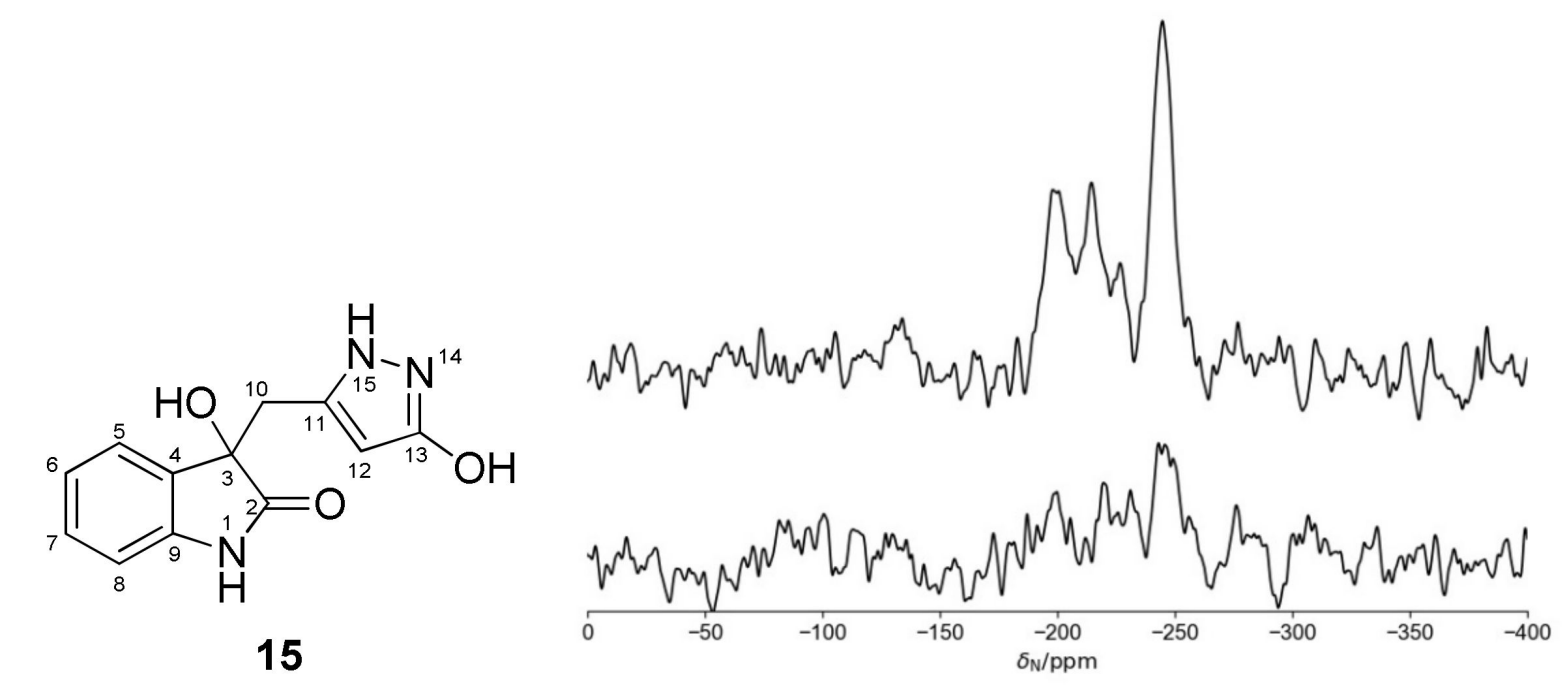
Structure of the desired material **15** and its ^15^N- cross polarisation (CP) (top) and cross-polarisation polarisation-inversion (CPPI) (bottom) MASNMR spectra.

In order to confirm the structure of the molecules obtained, we exploited different spectroscopic methodologies. Recorded solution-state Nuclear Magnetic Resonance (NMR) spectra confirm the presence of two diastereotopic protons (δ 3.03 and 2.88 ppm) that by 2D NMR correlate with carbons present on the 2-oxindole group and on the pyrazole. The signals regarding the 3-hydoxypyrazole (^13^C δ 160.46, 138.04, 88.86 ppm) match those obtained from similar scaffolds by Ayoub et al. in 1981 [28]. In their report, the authors noticed a proton exchange process occurs in solution which involves the pyrazole’s protons and all the other labile protons present in the molecule. Solution-state ^15^N-NMR heteronuclear single quantum coherence (HSQC) and heteronuclear multiple bond coherence (HMBC) spectra were acquired and allowed the assignment of the ^15^N chemical shift of N1; however, due to the fast proton exchange, the experiments were unable to identify the other two nitrogen. N14 and N15 (^15^N δ −198, −215 ppm) were instead identified, along with the N1 (−248 ppm), when solid-state ^15^N- magic angle spinning NMR (^15^N-MASNMR) spectra were acquired. Furthermore, suppression of both the N14 and N15 signals in a cross-polarisation polarisation-inversion (CPPI) experiment is an indication of a proton exchange process occurring in the solid state (Figure 4).

**Figure 5:**
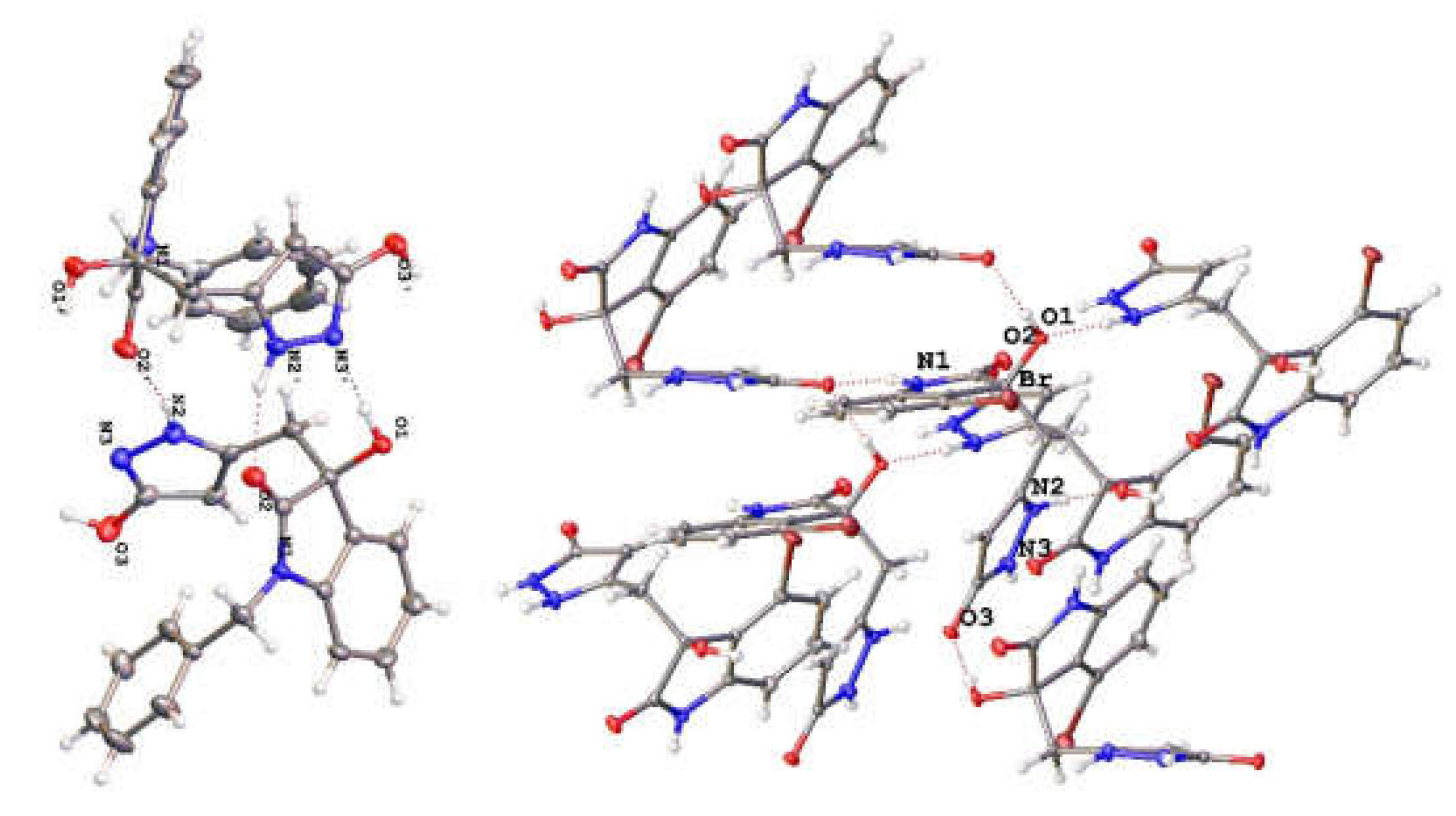
Pictures showing the packaging in the crystal structure of **15l** (left side) and **15c** (right side). The H-bonding interactions between the molecules can be noticed (N2/N3 = N15/14).

Unfortunately, all attempts to generate crystals of compound **15** produced amorphous powders. However, to our delight, we managed to crystallise certain derivatives of **15** and confirm the structures of the analogous products (e.g., **15c**, **15l**, Figure 5). In the solid state, the molecules **15** form closely packed lattices with extensive H-bonding interaction which would account for the ease of the proton exchange identified in the ^15^N-MASNMR analysis.

## 3. Materials and Methods

Unless otherwise stated, all solvents were purchased from Fisher Scientific (Loughborough, Leicestershire, UK) and used without further purification. Substrates, their precursors and reagents were purchased from Alfa Aesar (Haverhill, MA, USA), Sigma Aldrich (Merck KGaA, Darmstadt, Germany), Fluorochem (Hadfield, Derbyshire, United Kingdom) or TCI (Tokyo, Tokyo, Japan).

^1^H-NMR spectra were recorded on either Bruker Avance-400, Varian VNMRS-700 or Varian VNMRS-500 instruments (Bruker UK Limited, Coventry, UK) and are reported relative to residual solvent DMSO-*d*_6_ (δ 2.50 ppm). ^13^C-NMR spectra were recorded on the same instruments and are reported relative to DMSO-*d*_6_ (δ 39.52 ppm). Data for ^1^H-NMR are reported as follows: chemical shift (δ/ppm) (multiplicity, coupling constant (Hz), integration). Multiplicities are reported as follows: s = singlet, d = doublet, t = triplet, q = quartet, m = multiplet, brs = broad signal. Data for ^13^C-NMR are reported in terms of chemical shift (δ_C_/ppm). DEPT-135, COSY, HSQC, HMBC, PSYCHE and NOESY experiments were used in structural assignments. Solid-state NMR experimental information is included in the Supplementary Information. Single crystals X-ray diffraction experiments were carried out on a Bruker D8 Venture diffractometer with PHOTON 100 CMOS area detector (Bruker UK Limited, Coventry, UK), using Mo-*K*α (**15c**, **15l**) or Cu-*K*α (**16**) radiation from Incoatec IμS microsources with focusing mirrors. The crystals were cooled using a Cryostream 700 (Oxford Cryosystems, (Oxford, Oxfordshire, UK) open-flow N_2_ gas cryostat. The structures were solved by dual-space intrinsic phasing (SHELXT [29] program) and refined by full-matrix least squares using SHELXL [30] software on Olex2 platform [31].

IR spectra were obtained using a Perkin Elmer Spectrum Two UATR Two FT-IR Spectrometer (neat, ATR sampling, (Waltham, MA, USA)) with the intensities of the characteristic signals being reported as weak (w, <20% of tallest signal), medium (m, 21–70% of tallest signal) or strong (s, >71% of tallest signal).

Low resolution liquid chromatography mass spectrometry (LC-MS) was performed using a Waters TQD mass spectrometer (Waters Corp., Milford, MA, USA) and an Acquity UPLC BEH C18 1.7 µm column (2.1 mm × 50 mm) in ESI mode. ESI-HRMS was performed using a Waters QtoF Premier mass spectrometer (Waters Corp., Milford, MA, USA). For accurate mass measurements the deviation from the calculated formula is reported in mDa. Melting points were recorded on an Optimelt automated melting point system with a heating rate of 1 °C/min and are uncorrected. All the purifications were performed using a Teledyne CombiFlash^®^ Rf+ (Teledyne ISCO, Lincoln, NE, USA) equipped with a RediSep Rf Gold 150 g high performance C18 cartridge. The purification was performed at a flow of 75 mL/min using a gradient from 15% methanol in water to 60% methanol in water in 20 min. The cartridge was then washed with 100% methanol and conditioned in 50% methanol in water for storage. The water from the sample was removed using a Labconco FreeZone 4.5 L freeze dry system (Labconco, Kansas City, MO, USA) connected with a Leybold Trivac^®^ B D4B rotary cane vacuum pump (Leybold GmbH, Cologne, Germany). Organic solutions were concentrated under reduced pressure using a Buchi rotary evaporator and high vacuum was achieved using an Edwards RV5 pump and Schlenk line (BÜCHI Labortechnik AG, Postfach, Flawil, Switzerland).

### 3.1. General Procedure for the Synthesis of ***14***

To a heterogeneous mixture of isatin (735 mg, 5 mmol, 1 eq.) in MeOH (36 mL), was added methyl acetoacetate (650 μL, 6 mmol, 1.2 eq.) and piperidine (50 μL, 0.5 mmol, 0.1 eq.). The mixture was stirred at room temperature for 3 days. The solvent was then removed under reduced pressure to furnish **14** as a crude amorphous powder. The residue was purified using flash chromatography (eluent: 2.5% MeOH in dichloromethane) to isolate a pale orange amorphous solid (87% isolated yield).

### 3.2. General Procedure for the Synthesis of ***15a**–**j***

To a heterogeneous mixture of the appropriate isatin **13a**–**j** (5 mmol, 1 eq.) in MeOH (36 mL), was added methyl acetoacetate (650 μL, 6 mmol, 1.2 eq.) and piperidine (50 μL, 0.5 mmol, 0.1 eq.). The mixture was stirred at room temperature for 3 days. Hydrazine monohydrate (970 μL, 20 mmol, 4 eq.) was added dropwise at room temperature. After stirring the solution for 2 h, the mixture was partly concentrated in vacuo and Celite^®^ was added before all the solvent was removed. The residue was purified via reversed-phase chromatography as described above.

### 3.3. Characterisation of the Compounds ***14*** and ***15a-j*** and ***16*** Derivatives

*Methyl 4-(3-hydroxy-2-oxoindolin-3-yl)-3-oxobutanoate (***14***):*^1^H NMR (700 MHz, DMSO-*d*_6_) δ 10.24 (s, 1H), 7.23 (d, *J* = 7.3 Hz, 1H), 7.18 (td, *J* = 7.7, 1.3 Hz, 1H), 6.91 (td, *J* = 7.3, 1.0 Hz, 1H), 6.78 (d, *J* = 7.7 Hz, 1H), 6.07 (s, 1H), 3.61 (s, 2H), 3.57 (s, 3H), 3.38 – 3.34 (m, 1H, overlapping with H_2_O), 3.09 (d, *J* = 17.0 Hz, 1H). ^13^C NMR (176 MHz, DMSO-*d*_6_) δ 200.27, 177.89, 167.36, 142.48, 131.20, 129.09, 123.79, 121.28, 109.48, 72.52, 51.76, 49.49, 49.20. IR (neat): ν (cm^−1^) = 3264 (OH, br), 2955 (CH, w), 1705 (C=O, s), 16,121 (CH, m), 1472 (OH, m), 1185 (C-O, m), 1013 (w), 753 (m). LC-MS Rt = 1.18 min *m*/*z* [M + H]^+^ = 264.2; HR-MS calculated for C_13_H_14_NO_5_ 264.0872, found 264.0855 (Δ = −1.7 mDa). Melting point (°C): 100 °C decomposition.

*3-Hydroxy-3-[(3-hydroxy-1H-pyrazol-5-yl)methyl]indolin-2-one (***15***):*^1^H NMR (700 MHz, DMSO-*d*_6_) δ 10.20 (brs, 2H), 7.17 (td, *J* = 7.7, 1.3 Hz, 1H), 7.11 (dd, *J* = 7.4, 1.3 Hz, 1H), 6.93 (td, *J* = 7.5, 1.0 Hz, 1H), 6.73 (d, *J* = 7.7 Hz, 1H), 4.80 (s, 1H), 3.05 (d, *J* = 14.0 Hz, 1H), 2.90 (d, *J* = 14.0 Hz, 1H). ^13^C NMR (176 MHz, DMSO-*d*_6_) δ 178.51, 160.48, 142.01, 138.04, 131.20, 129.12, 124.32, 121.45, 109.48, 88.86, 74.89, 34.38. ^15^N NMR (71 MHz, DMSO-*d*_6_) δ -246.15 (^15^N-^1^H HSQC shows correlation with the peak at δ 10.19 ppm in the ^1^H-NMR). ^13^C CPMAS MS-NMR (101 MHz) δ 179.98, 162.65, 142.72, 139.51, 132.79, 129.78, 127.43, 123.91, 110.57, 91.77, 76.77, 35.52. ^15^N CPMAS MS-NMR (40.556 MHz) δ −197.54, −214.53, −244.66. LC-MS (ESI+) Rt = 0.70 min *m*/*z* [M + H]^+^ = 246.7 HR-MS calculated for C_12_H_12_N_3_O_3_ 246.0879, found 246.0878 (∆ = −0.1 mDa). IR (neat): ν (cm^−1^) = 3161 (NH, w), 2966 (CH, w), 1703 (C=O, s), 1595 (C=N, s), 1471 (s), 1340 (w), 1259 (m), 1078 (m), 1043 (s), 778 (w), 748 (w). Melting point (°C): 188 °C decomposition.

*2-(2-Hydrazinyl-2-oxoethyl)quinoline-4-carbohydrazide (***16***):*^1^H NMR (700 MHz, DMSO-*d*6) δ 9.90 (s, 1H), 9.37 (s, 1H), 8.10 (d, *J* = 8.4 Hz, 1H), 7.99 (d, *J* = 8.4 Hz, 1H), 7.78 (t, *J* = 7.7 Hz, 1H), 7.61 (t, *J* = 7.6 Hz, 1H), 7.50 (s, 1H), 4.67 (s, 2H), 4.29 (s, 2H), 3.78 (s, 2H). ^13^C NMR (176 MHz, DMSO-*d*6) δ 168.5, 166.3, 156.9, 147.8, 141.8, 130.3, 129.2, 127.1, 125.7, 123.7, 120.3, 44.1. IR (neat): ν (cm^−1^)= 3285 (NH, m), 3176 (NH, m), 1620 (C=O, s), 1533 (s), 1362 (m), 1174 (w), 1035.9 (w), 1005 (m), 879.4 (m), 758.4 (s), 699.1 (s). LC-MS: Rt= 2.41 min *m*/*z* [M + H]^+^ = 176.6; HR-MS calculated for C_12_H_14_N_5_O_2_ 260.1147, found 260.1136 (Δ = −1.1 mDa). Melting point (°C): 220 °C decomposition. *Crystal data:* C_12_H_15_N_5_O_3_·H_2_O (*M* = 277.29 g/mol), monoclinic, space group P2_1_/n (no. 14), *a* = 4.9407(5) Å, *b* = 14.0587(13) Å, *c* = 18.2746(16) Å, *β* = 97.040(5)°, *V* = 1259.8(2) Å^3^, *Z* = 4, *T* = 120 K, μ(Cu-*K*α) = 0.91 mm^−1^, *Dcalc* = 1.462 g/cm^3^, 7750 reflections measured (2Θ ≤ 117°), 1719 unique (*R*_int_ = 0.086, R_σ_ = 0.080), *R*_1_ = 0.049 on 1183 data with *I* > 2σ(*I*), *wR*_2_ = 0.106 on all data, CCDC-1986566.

*4-Fluoro-3-hydroxy-3-((3-hydroxy-1H-pyrazol-5-yl)methyl)indolin-2-one (***15a***):*^1^H NMR (700 MHz, DMSO-*d*_6_) δ 10.18 (brs, 3H), 7.22 (td, *J* = 8.1, 5.4 Hz, 1H), 6.73 (t, *J* = 8.8 Hz, 1H), 6.55 (d, *J* = 7.7 Hz, 1H), 4.65 (s, 1H), 3.19 (d, *J* = 14.0 Hz, 1H), 3.08 (d, *J* = 14.0 Hz, 1H). ^13^C NMR (176 MHz, DMSO-*d*_6_) δ 177.58, 159.75, 158.35, 144.36 (d, *J* = 9.6 Hz), 137.71, 131.45 (d, *J* = 8.5 Hz), 116.53 (d, *J* = 19.7 Hz), 109.01 (d, *J* = 20.6 Hz), 106.03, 87.87, 75.21, 33.30.^13^C CPMAS NMR (101 MHz) δ 179.99, 163.78, 161.32, 158.58, 143.68, 132.32, 115.96, 108.95, 91.29, 77.24, 34.06. ^15^N CPMAS NMR (40.556 MHz) δ −203.58, −221.81, −243.52. LC-MS (ESI+) Rt = 0.80 min m/z [M+H]^+^ = 264.2. HR-MS calculated for C_12_H_11_N_3_O_3_F 264.0784, found 264.0766 (∆ = −1.8 mDa). IR (neat): ν (cm^−1^) = 3103 (NH, w), 2748 (CH, w), 1709 (C=O, s), 1638 (C=N, s), 1567 (s), 1493 (m), 1464 (s), 1256 (w), 1229 (m), 1053 (m), 1033 (w), 1005 (w), 765 (s), 682 (w). Melting point (°C): 100 °C decomposition.

*4-Chloro-3-hydroxy-3-((3-hydroxy-1H-pyrazol-5-yl)methyl)indolin-2-one (***15b***):*^1^H NMR (599 MHz, DMSO-*d*_6_) δ 10.32 (brs, 3H), 7.20 (t, *J* = 8.0 Hz, 1H), 6.95 (d, *J* = 8.2 Hz, 1H), 6.67 (d, *J* = 7.8 Hz, 1H), 6.27 (s, 1H), 4.55 (s, 1H), 3.47 (d, *J* = 14.0 Hz, 1H), 3.05 (d, *J* = 14.0 Hz, 1H). ^13^C NMR (151 MHz, DMSO-*d*_6_) δ 177.46, 160.57, 144.34, 137.42, 130.90, 130.54, 127.14, 122.35, 108.44, 87.63, 76.14, 32.08. ^13^C CPMAS NMR (101 MHz) δ 179.71, 164.07, 144.60, 134.63, 129.32, 123.84, 110.28, 92.38, 77.59, 33.72. ^15^N CPMAS NMR (40.556 MHz) δ −217.29, −224.15, −241.86. LC-MS (ESI+) Rt = 1.35 min *m*/*z* [M + H]^+^ = 280.5 HR-MS calculated for C_12_H_11_N_3_O_3_Cl 280.0489, found 280.0485 (∆= 0.4 mDa). IR (neat): ν (cm^−1^) = 3074 (NH, w), 2703 (CH, w), 1713 (C=O, s), 1589 (C=N, s), 1449 (s), 1415 (m), 1175 (m), 1083 (m), 948 (w), 765 (s), 657 (w). Melting point (°C): 135 °C decomposition.

*4-Bromo-3-hydroxy-3-((3-hydroxy-1H-pyrazol-5-yl)methyl)indolin-2-one (***15c***):*^1^H NMR (700 MHz, DMSO-*d*_6_) δ 10.32 (brs, 3H), 7.19 – 7.03 (m, 2H), 6.70 (dd, *J* = 6.6, 2.1 Hz, 1H), 6.24 (s, 1H), 4.51 (s, 1H), 3.56 (d, *J* = 14.0 Hz, 1H), 3.01 (d, *J* = 14.0 Hz, 1H). ^13^C NMR (176 MHz, DMSO-*d*_6_) δ 177.52, 160.25, 144.62, 137.61, 131.14, 128.77, 125.44, 119.06, 108.93, 87.68, 76.71, 31.92. ^13^C CPMAS NMR (101 MHz) δ 179.77, 164.39, 145.28, 134.65, 125.47, 111.34, 92.26, 78.21, 33.85. ^15^N CPMAS NMR (40.556 MHz) δ −217.48, −223.79, −243.05. LC-MS (ESI+) Rt= 1.45 min *m*/*z* [M + H]^+^ = 323.8 HR-MS calculated for C_12_H_11_N_3_O_3_Br 323.9984, found 323.9973 (∆= −1.1 mDa). IR (neat): ν (cm^−1^) = 3000 (NH, w), 2710 (CH, w), 1715 (C=O, s), 1585 (C=N, s), 1445 (s), 1413 (m), 1329 (w), 1169 (m), 1082 (m), 929 (w), 763 (s), 660 (s). Melting point (°C): 103 °C decomposition. *Crystal data:* C_12_H_10_BrN_3_O_3_ (*M* = 324.14 g/mol), orthorhombic, space group P2_1_2_1_2_1_ (no. 19), *a* = 6.9156(3) Å, *b* = 13.1025(6) Å, *c* = 13.3565(6) Å, *V* = 1210.25(9) Å^3^, *Z* = 4, *T* = 120 K, μ(MoKα) = 3.40 mm^−1^, *Dcalc* = 1.779 g/cm^3^, 32,088 reflections measured (2Θ ≤ 66.5°), 4654 unique (*R*_int_ = 0.036, R_σ_ = 0.027), *R*_1_ = 0.025 on 4238 reflections with *I* > 2σ(*I*), *wR*_2_ = 0.058 on all data. CCDC 1986565.

*5-Chloro-3-hydroxy-3-((3-hydroxy-1H-pyrazol-5-yl)methyl)indolin-2-one (***15d***):*^1^H NMR (599 MHz, DMSO-*d*_6_) δ 10.30 (brs, 1H), 7.22 (dd, *J* = 8.3, 2.2 Hz, 1H), 7.12 (d, *J* = 2.3 Hz, 1H), 6.73 (d, *J* = 8.3 Hz, 1H), 6.28 (s, 1H), 4.84 (s, 1H), 3.06 (d, *J* = 14.0 Hz, 1H), 2.90 (d, *J* = 14.0 Hz, 1H). ^13^C NMR (151 MHz, DMSO-*d*_6_) δ 178.11, 140.87, 133.20, 128.88, 125.45, 124.48, 110.89, 88.83, 75.08, 24.39. ^13^C CPMAS NMR (101 MHz) δ 181.73, 163.08, 146.44, 140.53, 132.67, 128.35, 112.33, 90.37, 76.86, 36.96. ^15^N CPMAS NMR (40.556 MHz) −198.84, −211.43, −245.96. LC-MS (ESI+) Rt = 1.07 min *m*/*z* [M + H]^+^ = 280.6 HR-MS calculated for C_12_H_11_N_3_O_3_Cl 280.0489, found 280.0505 (∆ = 1.6 mDa). IR (neat): ν (cm^−1^) = 3173 (NH, w), 2627 (CH, w), 1670 (C=O, m), 1610 (C=N, s), 1480 (m), 1440 (m), 1249 (w), 1192 (w), 973 (w), 817 (w), 758 (s), 615 (s). Melting point (°C): 140 °C decomposition.

*3-Hydroxy-3-((3-hydroxy-1H-pyrazol-5-yl)methyl)-5-methoxyindolin-2-one (***15e***):*^1^H NMR (599 MHz, DMSO-*d*_6_) δ 10.02 (brs, 3H), 6.75 – 6.69 (m, 2H), 6.64 (d, *J* = 8.2 Hz, 1H), 4.86 (s, 1H), 3.68 (s, 3H), 3.04 (d, *J* = 14.1 Hz, 1H), 2.87 (d, *J* = 14.1 Hz, 1H). ^13^C NMR (151 MHz, DMSO-*d*_6_) δ 178.41, 160.48, 154.70, 137.96, 135.13, 132.37, 113.70, 111.30, 109.78, 88.96, 75.27, 55.37, 34.35. ^13^C CPMAS NMR (101 MHz) δ 180.57, 162.56, 156.41, 139.29, 133.63, 117.12, 112.15, 93.07, 77.07, 55.43, 34.66. ^15^N CPMAS NMR (40.556 MHz) δ −200.09, −221.84, −246.55. LC-MS (ESI+) Rt = 1.04 min *m*/*z* [M + H]^+^ = 276.6. HR-MS calculated for C_13_H_14_N_3_O_4_ 276.0984, found 276.0980 (∆ = −0.4 mDa). IR (neat): ν (cm^−1^) = 3064 (NH, w), 2758 (CH, w), 1714 (C=O, s), 1637 (C=N, s), 1597 (s), 1466 (s), 1233 (m), 1053 (m), 765 (s), 537 (m). Melting point (°C): 101 °C decomposition.

*5-Fluoro-3-hydroxy-3-((3-hydroxy-1H-pyrazol-5-yl)methyl)indolin-2-one (***15f**): ^1^H NMR (700 MHz, DMSO-*d*_6_) δ 10.23 (brs, 3H), 7.03 – 6.99 (m, 1H), 6.93 (dd, *J* = 8.2, 2.7 Hz, 1H), 6.71 (dd, *J* = 8.5, 4.3 Hz, 1H), 6.29 (s, 1H), 4.86 (s, 1H), 3.05 (d, *J* = 14.0 Hz, 1H), 2.91 (d, *J* = 14.0 Hz, 1H). ^13^C NMR (176 MHz, DMSO-*d*_6_) δ 178.43, 160.47 (d, *J* = 33.2 Hz), 158.56, 157.21, 138.11 (d, *J* = 1.7 Hz), 137.64, 132.93 (d, *J* = 7.6 Hz), 115.30 (d, *J* = 23.2 Hz), 112.08 (d, *J* = 24.3 Hz), 110.22 (d, *J* = 7.9 Hz), 88.88, 75.28, 34.27. ^13^C CPMAS NMR (101 MHz) δ 180.04, 163.66, 159.53, 143.98, 138.00, 130.76, 119.82, 111.70, 92.78, 77.29, 35.66. ^15^N CPMAS NMR (40.556) δ −216.10, −225.14, −242.50. LC-MS (ESI+) Rt = 0.80 min *m*/*z* [M + H]^+^ = 264.4 HR-MS calculated for C_12_H_11_N_3_O_3_F 264.0784, found 264.0763 (∆ = −2.1 mDa). IR (neat): ν (cm^−1^) = 3250 (NH, w), 2965 (w), 2862 (CH, w), 1708 (C=O, m), 1588 (C=N, m), 1485 (s), 1363 (w), 1187 (m), 1077 (w), 1033 (w), 825 (m), 601 (s). Melting point (°C): 110 °C decomposition.

*6-Chloro-3-hydroxy-3-((3-hydroxy-1H-pyrazol-5-yl)methyl)indolin-2-one (***15g***):*^1^H NMR (599 MHz, DMSO-*d*_6_) δ 10.31 (brs, 3H), 7.10 (d, *J* = 7.9 Hz, 1H), 6.98 (dd, *J* = 7.9, 1.9 Hz, 1H), 6.74 (d, *J* = 1.9 Hz, 1H), 6.23 (s, 1H), 4.83 (s, 1H), 3.04 (d, *J* = 14.0 Hz, 1H), 2.90 (d, *J* = 14.0 Hz, 1H). ^13^C NMR (151 MHz, DMSO-*d*_6_) δ 178.38, 143.52, 133.28, 130.06, 125.78, 121.16, 109.52, 88.84, 74.61, 39.10, 34.19. ^13^C CPMAS NMR (101 MHz) δ 180.52, 163.52, 142.20, 136.09, 127.70, 111.70, 92.75, 75.70, 35.04. ^15^N CPMAS NMR (40.556 MHz) δ −199.82, −209.90, −242.92. LC-MS (ESI+) Rt = 1.48 min *m*/*z* [M + H]^+^ = 280.6 HR-MS calculated for C_12_H_11_N_3_O_3_Cl 280.0489, found 280.0498 (∆ = 0.9 mDa). IR (neat): ν (cm^−1^) = 3147 (NH, w), 2771 (CH, w), 1711 (C=O, s), 1592 (C=N, s), 1482 (m), 1447 (w), 1331 (w), 1068 (m), 917 (w), 738 (w), 593 (w). Melting point (°C): 120 °C decomposition.

*7-Chloro-3-hydroxy-3-((3-hydroxy-1H-pyrazol-5-yl)methyl)indolin-2-one (***15h***):*^1^H NMR (400 MHz, DMSO-*d*_6_) δ 7.25 (dd, *J* = 8.1, 1.1 Hz, 1H), 7.07 (dd, *J* = 7.3, 1.2 Hz, 1H), 6.96 (dd, *J* = 8.1, 7.4 Hz, 1H), 4.80 (s, 1H), 3.05 (d, *J* = 14.1 Hz, 1H), 2.92 (d, *J* = 14.0 Hz, 1H). ^13^C NMR (101 MHz, DMSO-*d*_6_) δ 178.45, 144.15, 139.69, 133.18, 129.10, 122.95, 122.88, 113.68, 88.71, 75.53, 46.10. ^13^C CPMAS NMR (101 MHz) δ 180.70, 162.73, 145.14, 139.39, 131.75, 130.50, 123.84, 115.65, 90.98, 77.51, 35.83. ^15^N CPMAS NMR (40.556 MHz) −200.62, −216.52, −247.87. LC-MS (ESI+) Rt = 1.33 min *m*/*z* [M + H]^+^ = 280.5 HR-MS calculated for C_12_H_11_N_3_O_3_Cl 280.0489, found 280.0501 (∆ = 1.2 mDa). IR (neat): ν (cm^−1^) = 3137 (NH, m), 2752 (CH, w), 1713 (C=O, s), 1620 (C=N, s), 1562 (s), 1474 (m), 1454 (m), 1223 (w), 1171 (m), 1138, (s), 782 (s), 736 (s). Melting point (°C): 134 °C decomposition.

*3-Hydroxy-3-((3-hydroxy-1H-pyrazol-5-yl)methyl)-7-(trifluoromethyl)indolin-2-one (***15i***):*^1^H NMR (599 MHz, DMSO-*d*_6_) δ 10.00 (brs, 3H), 7.47 (d, *J* = 8.0 Hz, 1H), 7.35 (d, *J* = 7.3 Hz, 1H), 7.12 (t, *J* = 7.7 Hz, 1H), 6.37 (s, 1H), 4.82 (s, 1H), 3.10 (d, *J* = 14.1 Hz, 1H), 2.93 (d, *J* = 14.1 Hz, 1H). ^13^C NMR (151 MHz, DMSO-*d*_6_) δ 178.93, 160.32, 139.45, 137.62, 133.13, 128.23, 125.50 (q, *J* = 4.4 Hz), 123.95 (q, *J* = 271.7 Hz), 121.65, 110.50 (q, *J* = 32.6 Hz), 88.76, 73.66, 34.21. ^13^C CPMAS NMR (101 MHz) δ 181.20, 163.58, 142.93, 138.75, 132.50, 130.62, 126.20, 122.33, 112.60, 93.37, 72.54, 48.96, 32.16. ^15^N CPMAS NMR (40.556 MHz) δ −206.98, −217.10, −246.09. LC-MS (ESI+) Rt = 1.93 min *m*/*z* [M + H]^+^ = 314.3 HR-MS calculated for C_13_H_11_N_3_O_3_F_3_ 314.0753, found 314.0758 (∆ = 0.5 mDa). IR (neat): ν (cm^−1^) = 3175 (NH, s), 2826 (CH, w), 1734 (C=O, s), 1594 (s), 1455 (s), 1325 (s), 1170 (s), 1119 (s), 1104 (s), 1038 (s), 801 (s), 752 (s), 711 (s), 612 (s). Melting point (°C): 115 °C decomposition.

*7-Fluoro-3-hydroxy-3-((3-hydroxy-1H-pyrazol-5-yl)methyl)indolin-2-one (***15j***):*^1^H NMR (599 MHz, DMSO-*d*_6_) δ 10.42 (brs, 3H), 7.10 (ddd, *J* = 10.4, 7.9, 1.5 Hz, 1H), 7.02 – 6.83 (m, 2H), 6.30 (brs, 1H), 4.78 (s, 1H), 3.06 (d, *J* = 14.1 Hz, 1H), 2.93 (d, *J* = 14.0 Hz, 1H). ^13^C NMR (151 MHz, DMSO-*d*_6_) δ 178.28, 160.33, 147.03, 145.43, 137.71, 134.26 (d, *J* = 3.2 Hz), 128.89 (d, *J* = 12.1 Hz), 122.39 (d, *J* = 5.6 Hz), 120.36 (d, *J* = 3.0 Hz), 116.17 (d, *J* = 17.2 Hz), 88.70, 75.10 (d, *J* = 2.8 Hz), 34.42. ^13^C CPMAS NMR (101 MHz) δ 178.95, 162.17, 148.70, 146.06, 142.92, 132.71, 129.95, 124.18, 119.87, 118.74, 90.85, 76.82, 35.85. ^15^N CPMAS NMR (40.556) δ −216.39, −223.03, −250.73. LC-MS (ESI+) Rt = 0.77 min *m*/*z* [M + H]^+^= 264.5. HR-MS calculated for C_12_H_11_N_3_O_3_F 264.0784, found 264.0764 (∆ = −2.0 mDa). IR (neat): ν (cm^−1^)= 3698 (NH, w), 3358 (NH, w), 2974 (CH, s), 1731 (C=O, s), 1647 (w), 1587 (C=N, s), 1557 (s), 1493 (s), 1469 (s), 1332 (w), 1192 (w), 1053 (s), 1033 (s), 1014 (s), 791 (s), 758 (s), 731 (s), 567 (s). Melting point (°C): 115 °C decomposition.

*6-Chloro-2-(2-hydrazinyl-2-oxoethyl)quinoline-4-carbohydrazide (***16d***):*^1^H NMR (400 MHz, DMSO-*d*_6_) δ 9.97 (s, 1H), 9.38 (s, 1H), 8.17 (d, *J* = 2.4 Hz, 1H), 8.02 (d, *J* = 9.0 Hz, 1H), 7.81 (dd, *J* = 9.0, 2.5 Hz, 1H), 7.58 (s, 1H), 4.71 (s, 2H), 4.30 (s, 2H), 3.78 (s, 2H). ^13^C NMR (101 MHz, DMSO) δ 167.91, 165.43, 157.31, 145.92, 140.34, 131.34, 131.00, 130.42, 124.16, 123.99, 121.03, 43.64. LC-MS (ESI+) Rt = 1.42 min *m*/*z* [M + H]^+^ = 294.3 HR-MS calculated for C_12_H_13_N_5_O_2_Cl 294.0758, found 294.0771 (∆ = 1.3 mDa). IR (neat): ν (cm^−1^) = 3295 (NH, s), 3274 (NH, s), 3047 (CH, w), 1639 (C=O, s), 1618 (C=O, m), 1587 (C=N, m), 1528 (m), 1373 (m), 1303 (w), 1211 (w), 1013 (m), 1011 (m), 836 (s), 690 (s), 606 (s), 565 (s), 491 (s). Melting point (°C): 221 °C decomposition.

*6-Fluoro-2-(2-hydrazinyl-2-oxoethyl)quinoline-4-carbohydrazide (***16f***):*^1^H NMR (700 MHz, DMSO-*d*_6_) δ 9.95 (s, 1H), 9.37 (s, 1H), 8.07 (dd, *J* = 9.3, 5.6 Hz, 1H), 7.86 (dd, *J* = 10.2, 2.9 Hz, 1H), 7.71 (td, *J* = 8.8, 2.9 Hz, 1H), 7.58 (s, 0H), 4.55 (brs, 1H), 3.78 (s, 1H). ^13^C NMR (176 MHz, DMSO-*d*_6_) δ 168.00, 165.52, 160.53, 159.14, 156.14, 144.72, 140.54, 131.71, 123.97 (d, *J* = 10.5 Hz), 120.85, 119.88 (d, *J* = 25.7 Hz), 108.81 (d, *J* = 23.6 Hz), 43.53. LC-MS (ESI+) Rt = 0.97 min *m*/*z* [M + H]^+^ = 278.2. HR-MS calculated for C_12_H_13_N_5_O_2_F 278.1053, found 278.1035 (∆ = −1.8 mDa). IR (neat): ν (cm^−1^) = 3275 (NH, s), 3196 (w), 3054 (CH, w), 1643 (C=O, s), 1610 (m), 1533 (s), 1470 (w), 1381 (w), 1227 (w), 1012 (w), 837 (w), 682 (w), 579 (w), 500 (w). Melting point (°C): 210 °C decomposition.

*7-Chloro-2-(2-hydrazinyl-2-oxoethyl)quinoline-4-carbohydrazide (***16g***):*^1^H NMR (400 MHz, DMSO-*d*_6_) δ 9.97 (s, 1H), 9.39 (s, 1H), 8.14 (d, *J* = 9.0 Hz, 1H), 8.05 (s, 1H), 7.67 (d, *J* = 9.0 Hz, 1H), 7.54 (s, 1H), 4.69 (brs, 2H), 4.31 (brs, 2H), 3.78 (s, 2H). ^13^C NMR (101 MHz, DMSO) δ 167.93, 165.49, 158.18, 147.92, 141.33, 134.54, 127.48, 121.98, 120.54, 43.68. LC-MS (ESI+) Rt = 1.45 min *m*/*z* [M + H]^+^ = 294.3 HR-MS calculated for C_12_H_13_N_5_O_2_Cl 294.0758, found 294.0771 (∆ = 1.3 mDa). IR (neat): ν (cm^−1^) = 3288.5 (NH, m), 3250.2 (NH, m), 3074.61 (CH, w), 1713.87 (C=O, s), 1643.3 (C=O, s), 1616.38 (s), 1590.61 (s), 1533.77 (s), 1493.34 (m), 1361.98 (m), 1257.41 (w), 1138.43 (w), 961.82 (m), 839.91 (m), 756.43 (m). Melting point (°C): 223 °C decomposition.

*2-(2-Hydrazinyl-2-oxoethyl)-8-(trifluoromethyl)quinoline-4-carbohydrazide (***16i***):*^1^H NMR (599 MHz, DMSO-*d*_6_) δ 9.97 (s, 1H), 9.38 (s, 1H), 8.38 (dd, *J* = 8.5, 1.4 Hz, 1H), 8.21 (dd, *J* = 7.5, 1.4 Hz, 1H), 7.76 (dd, *J* = 8.5, 7.5 Hz, 1H), 7.66 (s, 1H), 4.70 (brs, 1H), 4.30 (brs, 1H), 3.84 (s, 2H). ^13^C NMR (151 MHz, DMSO-*d*_6_) δ 168.18, 165.79, 158.16, 143.93, 142.17, 130.78, 128.95 (q, *J* = 5.3 Hz), 126.44, 126.23, 124.50 (q, *J* = 272 Hz), 124.22, 121.36, 44.27. LC-MS (ESI+) Rt = 1.65 min *m*/*z* [M + H]^+^ = 328.3 HR-MS calculated for C_13_H_13_N_5_O_2_F_3_ 328.1021, found 328.1028 (∆ = 0.7 mDa). IR (neat): ν (cm^−1^) = 3295.15 (br, NH), 1670.75 (m, C=O), 1644.92 (s, C=O), 1596.49 (s), 1530.03 (s), 1363.51 (m), 1297.84 (s), 1162.63 (m), 1130.38 (s), 1051.34 (m), 1005.59 (m), 769.31 (m). Melting point (°C): 225 °C decomposition.

*1-Benzyl-3-hydroxy-3-((3-hydroxy-1H-pyrazol-5-yl)methyl)indolin-2-one* (**15l**): ^1^H NMR (599 MHz, DMSO-*d*_6_) δ 10.97 (brs, 2H), 7.30–7.24 (m, 3H), 7.24–7.20 (m, 1H), 7.17 (td, *J* = 7.7, 1.3 Hz, 1H), 7.06 – 6.99 (m, 3H), 6.66 (d, *J* = 7.8 Hz, 1H), 6.37 (s, 1H), 4.90 (d, *J* = 16.0 Hz, 1H), 4.71 – 4.65 (m, 2H), 3.18 (d, *J* = 13.9 Hz, 1H), 3.04 (d, *J* = 13.9 Hz, 1H). ^13^C NMR (151 MHz, DMSO-*d*_6_) δ 176.80, 160.57, 142.55, 137.73, 135.88, 130.69, 129.16, 128.57, 127.11, 126.81, 124.07, 122.25, 108.99, 89.00, 74.93, 42.46, 34.66. ^13^C CPMAS NMR (101 MHz) δ 178.20, 177.34, 164.17, 159.74, 143.92, 141.99, 136.37, 134.72, 131.23, 129.81, 128.03, 126.61, 125.39, 124.44, 123.57, 109.93, 91.69, 90.57, 77.74, 76.23, 44.55, 41.28, 35.56, 34.18. ^15^N CPMAS NMR (40.556) δ −134.38, −138.40, −199.90, −202.03, −237.79, −239.63. LC-MS (ESI+) Rt = 1.55 min *m*/*z* [M + H]^+^ = 335.8. HR-MS calculated for C_19_H_18_N_3_O_3_ 336.1348, found 336.1337 (∆ = −1.1 mDa). IR (neat): ν (cm^−1^) = 3228 (NH, w), 2940 (CH, W), 1699 (C=O, s), 1616 (C=N, s), 1569 (w), 1489 (m), 1469 (s), 1355 (s), 1259 (w), 1179 (m), 1074 (s), 1000 (m), 773 (s), 748 (s), 732 (s), 693 (s). Melting point (°C): 166 °C decomposition. *Crystal data*: C_19_H_17_N_3_O_3_ (*M* = 335.35 g/mol), monoclinic, space group P2_1_/n (no. 14), *a* = 8.5554(6), *b* = 29.118(2), *c* = 13.1745(9) Å, *β* = 92.172(3)°, *V* = 3279.6(4) Å^3^, *Z* = 8, *T* = 120 K, μ(Mo-*K*α) = 0.09 mm^−1^, *Dcalc* = 1.358 g/cm^3^, 48,703 reflections measured (2Θ ≤ 50°), 5792 unique (*R*_int_ = 0.067, *R*_σ_ = 0.049), *R*_1_ = 0.041 on 4146 reflections with *I* > 2σ(*I*), *wR*_2_ = 0.091 on all data, CCDC-1986567.

## 4. Conclusions

We developed a methodology for the synthesis of a new scaffold comprising of two key pharmacophores units. The procedure is simple and employs readily available hydrazine monohydrate and other cheap building blocks, such as methyl acetoacetate and isatin. A library of 12 compounds was prepared in order to study the versatility of the method which appears to be applicable to a range of isatin starting materials in which the 3-oxo group is active for aldol reactions. Furthermore, we acquired different spectroscopic analyses such as ^15^N-MASNMR, ^15^N-NMR and SXRD to confirm the structure of the products. To the best our knowledge, no ^15^N-MASNMR spectra on 3-hydroxypyrazole scaffolds had previously been performed. The compounds generated are currently under investigation for specific biological activity and will be reported separately.

## Figures and Tables

**Table 1:** Screening of different bases for the synthesis of **14.**

Entry *^a^*	Base (10 mol%)	Solvent	Conversion (%) *^b^*	Yield (%) *^c^*
1	Triethylamine	MeOH	38	36
2	DBU	MeOH	13	0
3***^d^***	DBU	THF	88	46
4	Pyrrolidine	MeOH	92	84
5	K_2_CO_3_	MeOH	77	26
6	Morpholine	MeOH	36	36
7	*N,N-*diethylethylendiamine	MeOH	55	33
**8**	Piperidine	MeOH	93	87
**9*^e^***	Piperidine	MeOH	-	-
10	Piperidine	*i*PrOH	96	84
11	Piperidine	H_2_O	53	15
12	Piperidine	MeCN	87	59
13	Piperidine	THF	90	67
14	Piperidine	Toluene	61	27
15	Piperidine	AcOEt	84	81

*^a^* The experiments were carried out at 1 mmol scale; *^b^* Detected conversions based on ^1^H-Nuclear Magnetic Resonance (^1^H-NMR) spectra; *^c^* Isolated yield following chromatography; *^d^* Note the original literature [23] used 5 equiv. of methyl acetoacetate; *^e^* The reaction mixture was at 65 °C for 2 h and a complex mixture was obtained, no further analysis was performed.

**Table 2:**
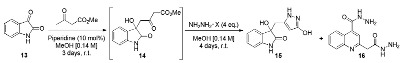
Screening of different hydrazine sources for the synthesis of the pyrazole **15**.

Entry *^a^*	Reagent *^b^*	Conv. 15 (%) *^c^*	Conv. 16 (%) *^c^*
1	NH_2_NH_2_ ∙H_2_O	21	36
2	NH_2_NH_2_ ∙HCl	20	20
3*^d^*	NH_2_NH_2_	53	42

*^a^* The experiments were carried out on 5 mmol scale; *^b^* 4 equivalents of reagent were used; *^c^* detected conversion based on ^1^H-NMR spectra; *^d^* A 1 M THF solution was employed.

**Table 3:**
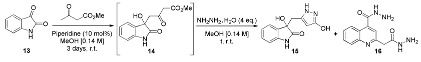
Optimisation of the reaction conditions for the preparation of the pyrazole **15**.

Entry *^a^*	Reaction Time (h)	Hydrazine Addition	Conv. 15 (%) *^b^*	Conv. 16 (%) *^b^*
1	96	fast	21	36
2	2	fast	28	17
3	2	dropwise *^c^*	50 (38)	16 (15)
4*^d^*	2	dropwise	43	11
5*^e^*	2	dropwise	53	10

*^a^* The experiments were carried out on 5 mmol scale; ^*b*^ detected conversions based on ^1^H-NMR spectra; *^c^* the hydrazine was added over 15 min; *^d^* addition performed at −78 ℃. Isolated yields in parentheses; *^e^* the hydrazine monohydrate was added as a 1 M solution in MeOH.

**Table 4:**
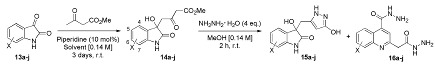
Application of the optimised conditions for the synthesis of the derivatives **15a**–**j**.

Entry *^a^*	X	Name	Conv. 15 (%) *^b^*	Conv. 16 (%) *^b^*
1	4-F	a	13 (10)	9 (- *^c^*)
2	4-Cl	b	92 (81)	0
3	4-Br	c	93 (91)	0
4	5-Cl	d	46 (40)	22 (20)
5	5-MeO	e	45 (41)	- *^c^* (11)
6	5-F	f	46 (44)	30 (25)
7	6-Cl	g	57 (55)	10 (5)
8	7-Cl	h	44 (38)	12 (10)
9	7-CF_3_	i	54 (53)	5 (- *^c^*)
10	7-F	j	32 (31)	- *^c^* (3)

^a^ The experiments were carried out on 5 mmol scale; ^b^ detected conversions based on ^1^H-NMR spectra, isolated yields in parentheses; ^c^ it was not possible to be acquired due to signal overlapping.

## Supplementary Materials

The following are available online, ^1^H and ^13^C-NMR spectra, ^13^C and ^15^N MASNMR spectra, mass spectra, infrared spectra and XRD crystallographic data of compounds. Crystallographic data in CIF format have been deposited with Cambridge Structural Database (CCDC-1986565 to 1986567) and are available online.

## References

[1] Karrouchi K., Radi S., Ramli Y., Taoufik J., Mabkhot Y.N., Al-Aizari F.A., Ansar M. (2018). Synthesis and Pharmacological Activities of Pyrazole Derivatives: A Review. Molecules.

[2] Li D., Zhan P., De Clercq E., Liu X. (2012). Strategies for the Design of HIV-1 Non-Nucleoside Reverse Transcriptase Inhibitors: Lessons from the Development of Seven Representative Paradigms. J. Med. Chem..

[3] Ohsumi K., Matsueda H., Hatanaka T., Hirama R., Umemura T., Oonuki A., Ishida N., Kageyama Y., Maezono K., Kondo N. (2003). Pyrazole-O-glucosides as novel Na(+)-glucose cotransporter (SGLT) inhibitors. Bioorg. Med. Chem. Lett..

[4] Pinnetti S., Streicher R., Thomas L., Dugi K. (2008). Pharmaceutical Composition Comprising a Pyrazole-O-Glucoside Derivative. U.S. Patent.

[5] Fushimi N., Fujikura H., Shiohara H., Teranishi H., Shimizu K., Yonekubo S., Ohno K., Miyagi T., Itoh F., Shibazaki T. (2012). Structure–activity relationship studies of 4-benzyl-1H-pyrazol-3-yl β-d-glucopyranoside derivatives as potent and selective sodium glucose co-transporter 1 (SGLT1) inhibitors with therapeutic activity on postprandial hyperglycemia. Bioorg. Med. Chem..

[6] Foley K.P., Du Z. (2015). Method for Treating Proliferative Disorders Associated with Mutations in C-Met. U.S. Patent.

[7] Pfefferkorn J.A., Choi C., Winters T., Kennedy R., Chi L., Perrin L.A., Lu G., Ping Y.-W., McClanahan T., Schroeder R. (2008). P2Y1 receptor antagonists as novel antithrombotic agents. Bioorg. Med. Chem. Lett..

[8] Song M.-X., Zheng C.-J., Deng X.-Q., Sun L.-P., Wu Y., Hong L., Li Y.-J., Liu Y., Wei Z.-Y., Jin M.-J. (2013). Synthesis and antibacterial evaluation of rhodanine-based 5-aryloxy pyrazoles against selected methicillin resistant and quinolone-resistant Staphylococcus aureus (MRSA and QRSA). Eur. J. Med. Chem..

[9] Jardosh H., Sangani C.B., Patel M.P., Patel R.G. (2013). One step synthesis of pyrido[1,2-a]benzimidazole derivatives of aryloxypyrazole and their antimicrobial evaluation. Chin. Chem. Lett..

[10] Tang Y.-Q., Sattler I., Thiericke R., Grabley S., Feng X.-Z. (2010). ChemInform Abstract: Maremycins C (I) and D (II), New Diketopiperazines, and Maremycins E (III) and F (IV), Novel Polycyclic spiro-Indole Metabolites Isolated from *Streptomyces* sp.. Eur. J. Org. Chem..

[11] Wang Y., Wang C., Lin H., Liu Y., Li Y., Zhao Y., Li P., Liu J. (2018). Discovery of the Potential Biomarkers for Discrimination between Hedyotis diffusa and Hedyotis corymbosa by UPLC-QTOF/MS Metabolome Analysis. Molecules.

[12] Qian P., Zhang Y.-B., Yang Y.-F., Xu W., Yang X. (2017). Pharmacokinetics Studies of 12 Alkaloids in Rat Plasma after Oral Administration of Zuojin and Fan-Zuojin Formulas. Molecules.

[13] Koguchi Y., Kohno J., Nishio M., Takahashi K., Okuda T., Ohnuki T., Komatsubara S. (2000). TMC-95A, B, C, and D, novel proteasome inhibitors produced by Apiospora montagnei Sacc. TC 1093. Taxonomy, production, isolation, and biological activities. J. Antibiot..

[14] Lin S., Yang Z.-Q., Kwok B.H.B., Koldobskiy M., Crews C.M., Danishefsky S.J. (2004). Total Synthesis of TMC-95A and -B via a New Reaction Leading toZ-Enamides. Some Preliminary Findings as to SAR. J. Am. Chem. Soc..

[15] Pantouris G., Loudon-Griffiths J., Mowat C.G. (2016). Insights into the mechanism of inhibition of tryptophan 2,3-dioxygenase by isatin derivatives. J. Enzym. Inhib. Med. Chem..

[16] Raj M., Veerasamy N., Singh V.K. (2010). Highly enantioselective synthesis of 3-cycloalkanone-3-hydroxy-2-oxindoles, potential anticonvulsants. Tetrahedron Lett..

[17] Prathima P.S., Rajesh P., Rao V.J., Kailash U.S., Sridhar B., Rao M.M. (2014). “On water” expedient synthesis of 3-indolyl-3-hydroxy oxindole derivatives and their anticancer activity in vitro. Eur. J. Med. Chem..

[18] Tokunaga T., Hume W.E., Umezome T., Okazaki K., Ueki Y., Kumagai K., Hourai S., Nagamine J., Seki H., Taiji M. (2001). Oxindole derivatives as orally active potent growth hormone secretagogues. J. Med. Chem..

[19] Tomita D., Yamatsugu K., Kanai M., Shibasaki M. (2009). Enantioselective Synthesis of SM-130686 Based on the Development of Asymmetric Cu(I)F Catalysis to Access 2-Oxindoles Containing a Tetrasubstituted Carbon. J. Am. Chem. Soc..

[20] Bergman J. (2015). Oxindoles. Advances in Heterocyclic Chemistry.

[21] Medvedev A.E., Buneeva O., Glover V. (2007). Biological targets for isatin and its analogues: Implications for therapy. Boil. Targets Ther..

[22] Liu H., Wu H.-Y., Luo Z., Shen J., Kang G., Liu B., Wan Z., Jiang J. (2012). Regioselectivity-Reversed Asymmetric Aldol Reaction of 1,3-Dicarbonyl Compounds. Chem. A Eur. J..

[23] Zhang D., Chen Y., Cai H., Yin L., Zhong J., Man J., Zhang Q.-F., Bethi V., Tanaka F. (2019). Direct Catalytic Asymmetric Synthesis of Oxindole-Derived δ-Hydroxy-β-ketoesters by Aldol Reactions. Org. Lett..

[24] Nagaraju S., Satyanarayana N., Paplal B., Vasu A.K., Kanvah S., Kashinath D. (2015). Synthesis of functionalized isoxazole–oxindole hybrids via on water, catalyst free vinylogous Henry and 1,6-Michael addition reactions. RSC Adv..

[25] Shvekhgeimer M.G.-A. (2004). The Pfitzinger Reaction. Eur. J. Org. Chem..

[26] Ramann G.A., Cowen B. (2016). Recent Advances in Metal-Free Quinoline Synthesis. Molecules.

[27] Health and Safety Executive (2020). EH40/2005 Workplace Exposure Limits Limits for Use with the Control of Substances.

[28] Ayoub M.T., Shandala M.Y., Bashi G.M.G., Pelter A. (1981). The conversion of 5,6-dihydro-4-methoxy-2-pyrones into 3-alkyl-5-hydroxypyrazoles. J. Chem. Soc. Perkin Trans. 1.

[29] Dolomanov O., Bourhis L.J., Gildea R., Howard J.A., Puschmann H. (2009). OLEX2: A complete structure solution, refinement and analysis program. J. Appl. Crystallogr..

[30] Sheldrick G.M. (2015). SHELXT - integrated space-group and crystal-structure determination. Acta Crystallogr. Sect. A Found. Adv..

[31] Sheldrick G.M. (2015). Crystal structure refinement with SHELXL. Acta Crystallogr. Sect. C Struct. Chem..

