# Supplementary material for: A One-Pot Divergent Sequence to Pyrazole and Quinoline Derivatives
Source: Molecules. 2020 May 5;25(9):2160. doi: 10.3390/molecules25092160 (PMC7249113; doi:10.3390/molecules25092160)
Supplement: Supplementary file 1 [file molecules-25-02160-s001.pdf]

Article

A one-pot synthesis of isatin derivatives

Guido Gambacorta[1], David C. Apperley,[1] and Ian R. Baxendale[1,*]

[1] Department of Chemistry, University of Durham, South Road Durham, DH1 3LE, United Kingdom.

* Correspondence; i.r.baxendale@durham.ac.uk Tel.: +44 (0) 191 33 42185.

Table of contents

1.1 Data of methyl 4-(3-hydroxy-2-oxoindolin-3-yl)-3-oxobutanoate (**14**) 3
1.2 Data of 3-hydroxy-3-((3-hydroxy-1H-pyrazol-5-yl)methyl)indolin-2-one (**15**) 7
1.3 Data of 4-fluoro-3-hydroxy-3-((3-hydroxy-1H-pyrazol-5-yl)methyl)indolin-2-one (**15a**) 15
1.4 Data of 4-chloro-3-hydroxy-3-((3-hydroxy-1H-pyrazol-5-yl)methyl)indolin-2-one (**15b**) 21
1.5 Data of 4-bromo-3-hydroxy-3-((3-hydroxy-1H-pyrazol-5-yl)methyl)indolin-2-one (**15c**) 27
1.6 Data of 5-chloro-3-hydroxy-3-((3-hydroxy-1H-pyrazol-5-yl)methyl)indolin-2-one (**15d**) 42
1.7 Data of 3-hydroxy-3-((3-hydroxy-1H-pyrazol-5-yl)methyl)-5-methoxyindolin-2-one (**15e**) 48
1.8 Data of 5-fluoro-3-hydroxy-3-((3-hydroxy-1H-pyrazol-5-yl)methyl)indolin-2-one (**15f**) 54
1.9 Data of 6-chloro-3-hydroxy-3-((3-hydroxy-1H-pyrazol-5-yl)methyl)indolin-2-one (**15g**) 60
1.10 Data of 7-chloro-3-hydroxy-3-((3-hydroxy-1H-pyrazol-5-yl)methyl)indolin-2-one (**15h**) 66
1.11 Data of 3-hydroxy-3-((3-hydroxy-1H-pyrazol-5-yl)methyl)-7-(trifluoromethyl)indolin-2-one (**15i**) 72
1.12 Data of 7-fluoro-3-hydroxy-3-((3-hydroxy-1H-pyrazol-5-yl)methyl)indolin-2-one (**15j**) 78
1.13 Data of 1-benzyl-3-hydroxy-3-((3-hydroxy-1H-pyrazol-5-yl)methyl)indolin-2-one (**15l**) 84
1.14 Data of 2-(2-hydrazinyl-2-oxoethyl)quinoline-4-carbohydrazide (**16**) 101
1.15 Data of 6-chloro-2-(2-hydrazinyl-2-oxoethyl)quinoline-4-carbohydrazide (**16d**) 111
1.16 Data of 6-fluoro-2-(2-hydrazinyl-2-oxoethyl)quinoline-4-carbohydrazide (**16f**) 115
1.17 Data of 7-chloro-2-(2-hydrazinyl-2-oxoethyl)quinoline-4-carbohydrazide (**16g**) 119
1.18 Data of 2-(2-hydrazinyl-2-oxoethyl)-8-(trifluoromethyl)quinoline-4-carbohydrazide (**16i**) 123
1.19 Spectra of Isatin (**13**) 127
1.20 Spectra of 4-fluoroisatin (**13a**) 130
1.21 Spectra of 5-chloroisatin (**13d**) 133
1.22 Spectra of 5-methoxyisatin (**13e**) 136
1.23 Spectra of 5-fluoroisatin (**13f**) 139
1.24 Spectra of 7-trifluoromethylisatin (**13i**) 142
1.25 Spectra of 7-fluroisatin (**13j**) 145

All the products characterized have NMR, FT-IR spectrums and accurate mass. The Solid-state NMR spectra were also acquired in order to allow the appearance of the signals (δ ~160, ~140, ~90 ppm) attributed to the two quaternary carbons present in the pyrrole ring that in the solution-state NMR appeared to be broad and undetectable in few cases.

Solid-state carbon-13 NMR spectra were obtained at 100.63 MHz using a Bruker Avance III HD spectrometer and a 4 mm (rotor outside diameter) magic-angle spinning probe. They were recorded using cross polarisation (CP) with TOSS spinning sideband suppression at a spin-rate of either 8 or 10 kHz. Spectral referencing was with respect to external, neat tetramethylsilane, carried out by setting the high-frequency signal from adamantine to 38.5 ppm. Nitrogen-15 spectra were obtained at 40.56 MHz using the same instrument and probe. They were recorded using cross polarisation and at a spin rate of 8 kHz. The cross-polarisation polarisation-inversion (CPPI) experiment was carried out with a polarisation inversion time of 200 µ Spectral referencing is with respect to neat nitromethane, carried out by setting the signal from glycine to -346.8 ppm. Recycle delays and contact times are given with the spectra.

In order to supplement our understanding of the electronic relationship and observed reactivity of the isatin starting materials we acquired ^{15}N-MASNMR spectra (**13**, **13a**, **13b**, **13e**, **13f**, **13i**, **13j**). This we conceived would potentially be indicative of the electronic environment around the nitrogen and thus its donating ability into the adjacent carbonyl. Unfortunately, no reasonable connection was found, however the acquired spectra are hereby attached.

Proposed mechanism for the formation of both compounds **15** and **16** are herein depicted (*Scheme S1*). In the formation of the quinoline, both water and hydrazine are nucleophilic to attack the amidic carbonyl and we do not have any evidence to speculate on when the acyl hydrazide are formed during the reaction.

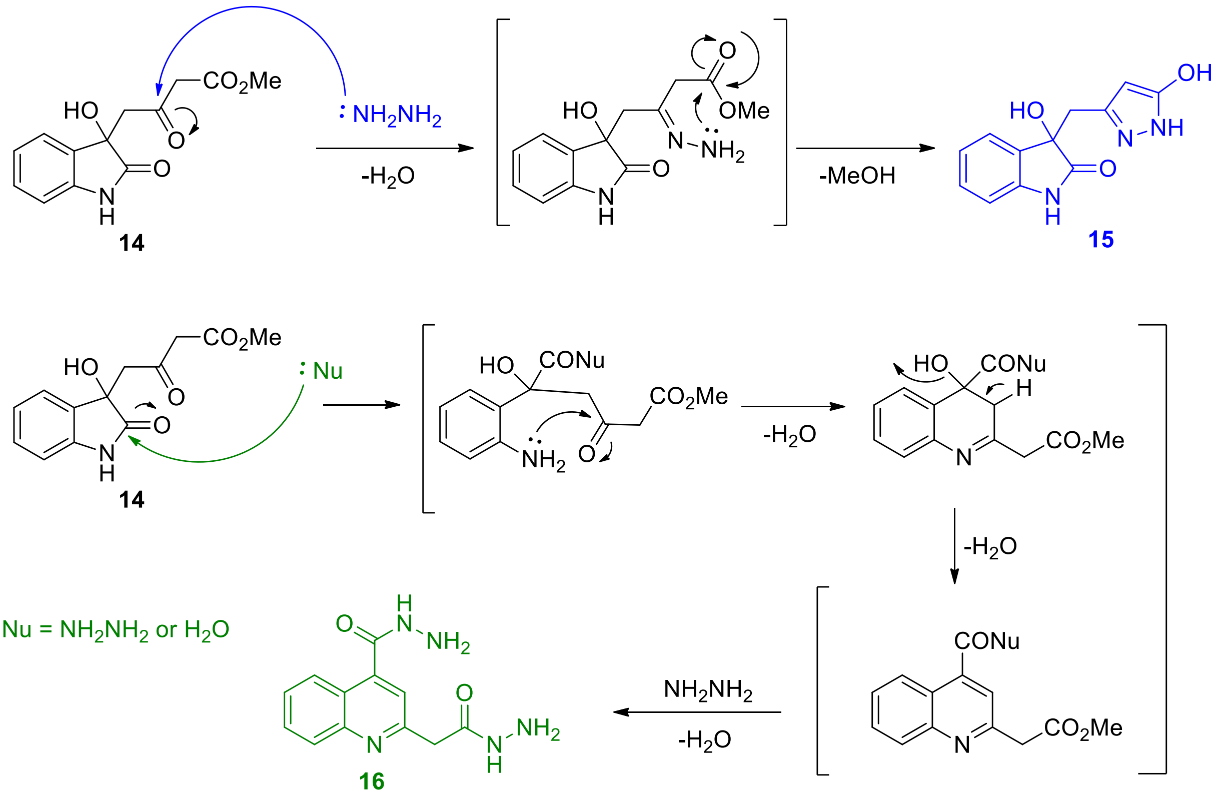

Scheme S1. *Proposed mechanism for the formation of the compounds* ***15*** *and* ***16****.*

*1.1 Data of methyl 4-(3-hydroxy-2-oxoindolin-3-yl)-3-oxobutanoate (**14**)*

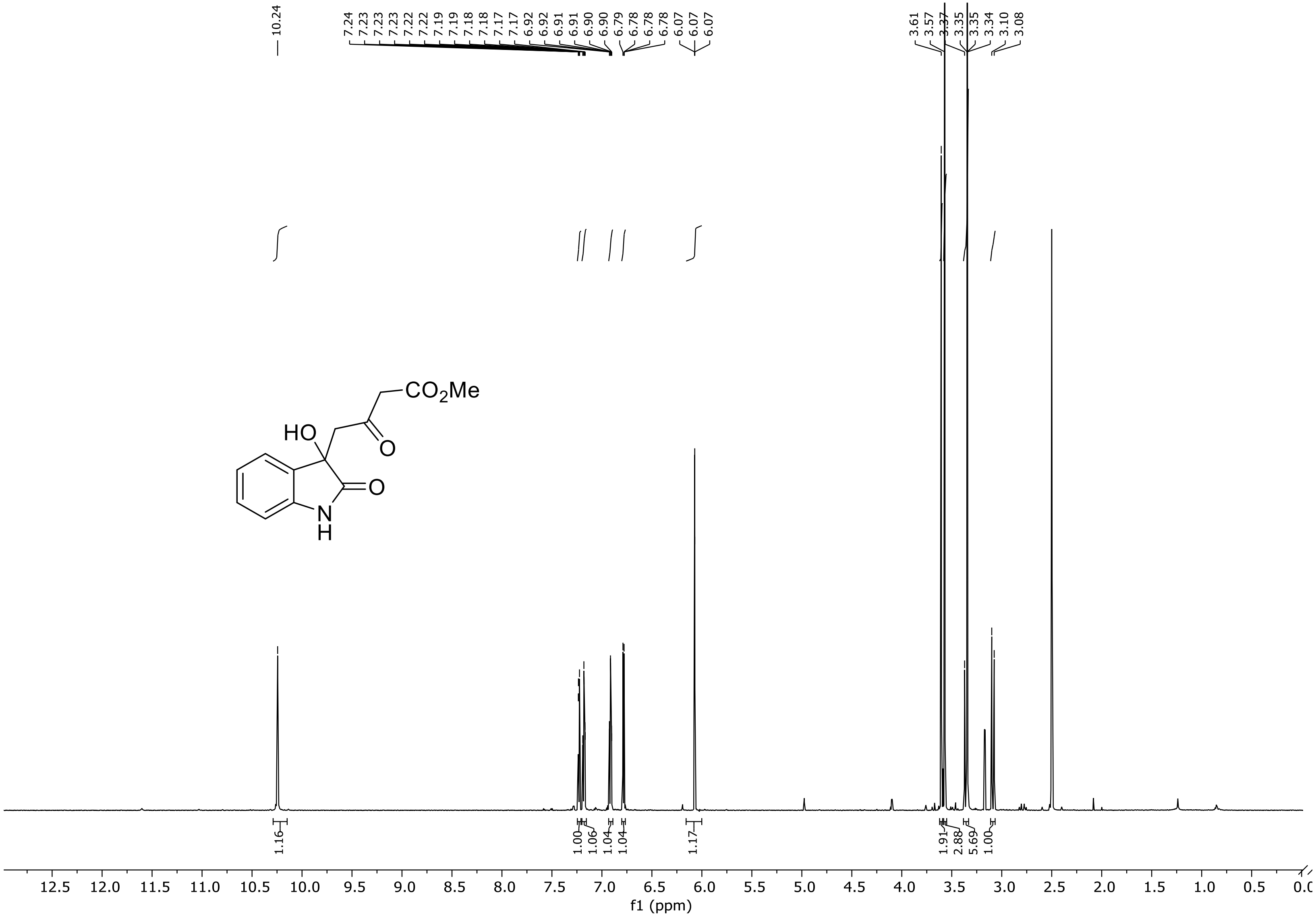

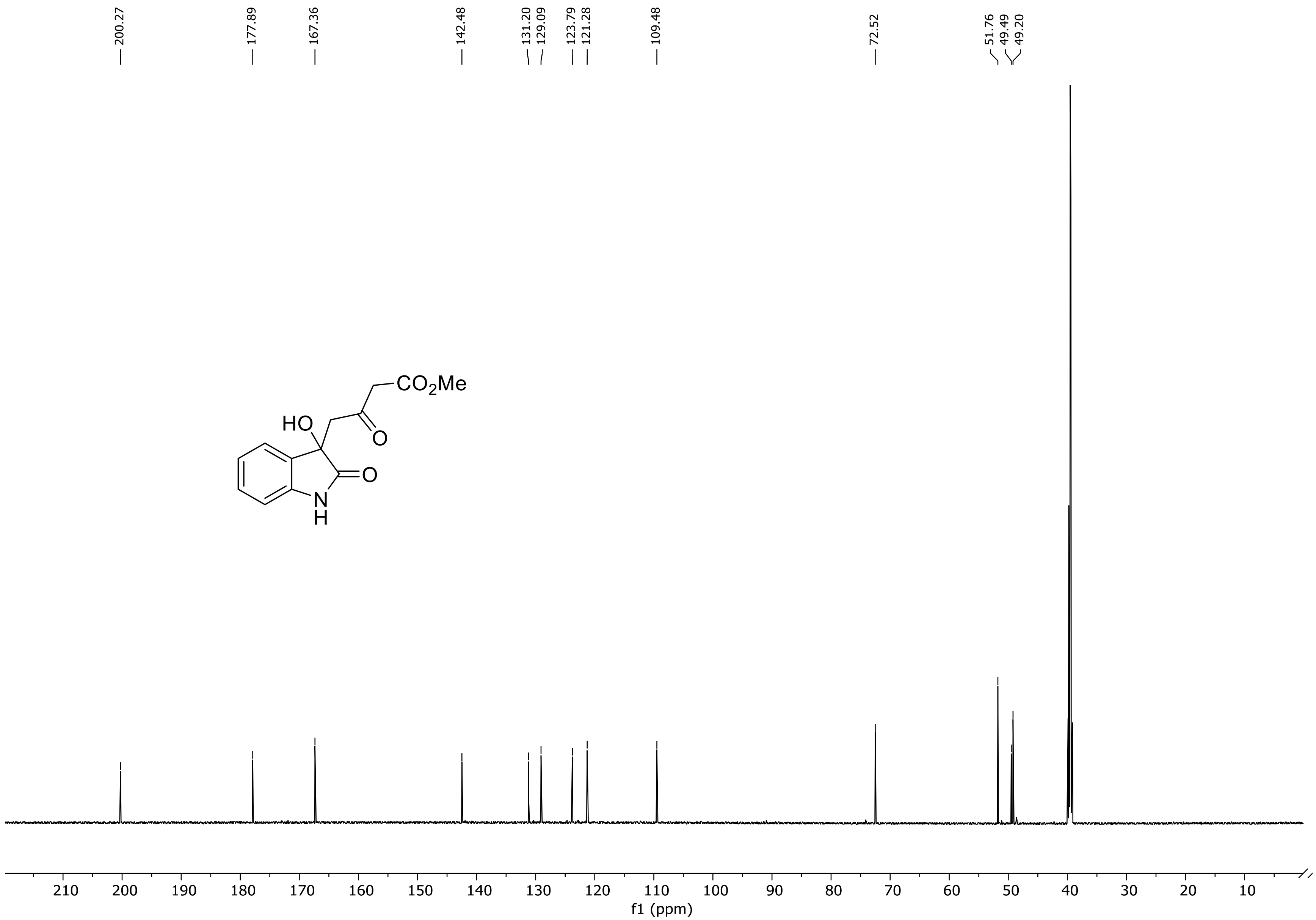
200.27
177.89
167.36
142.48
131.20
129.09
123.79
121.28
109.48
72.52
51.76
49.49
49.20
CO_2Me
HO
O
O
N
H
210 200 190 180 170 160 150 140 130 120 110 100 90 80 70 60 50 40 30 20 10
f1 (ppm)

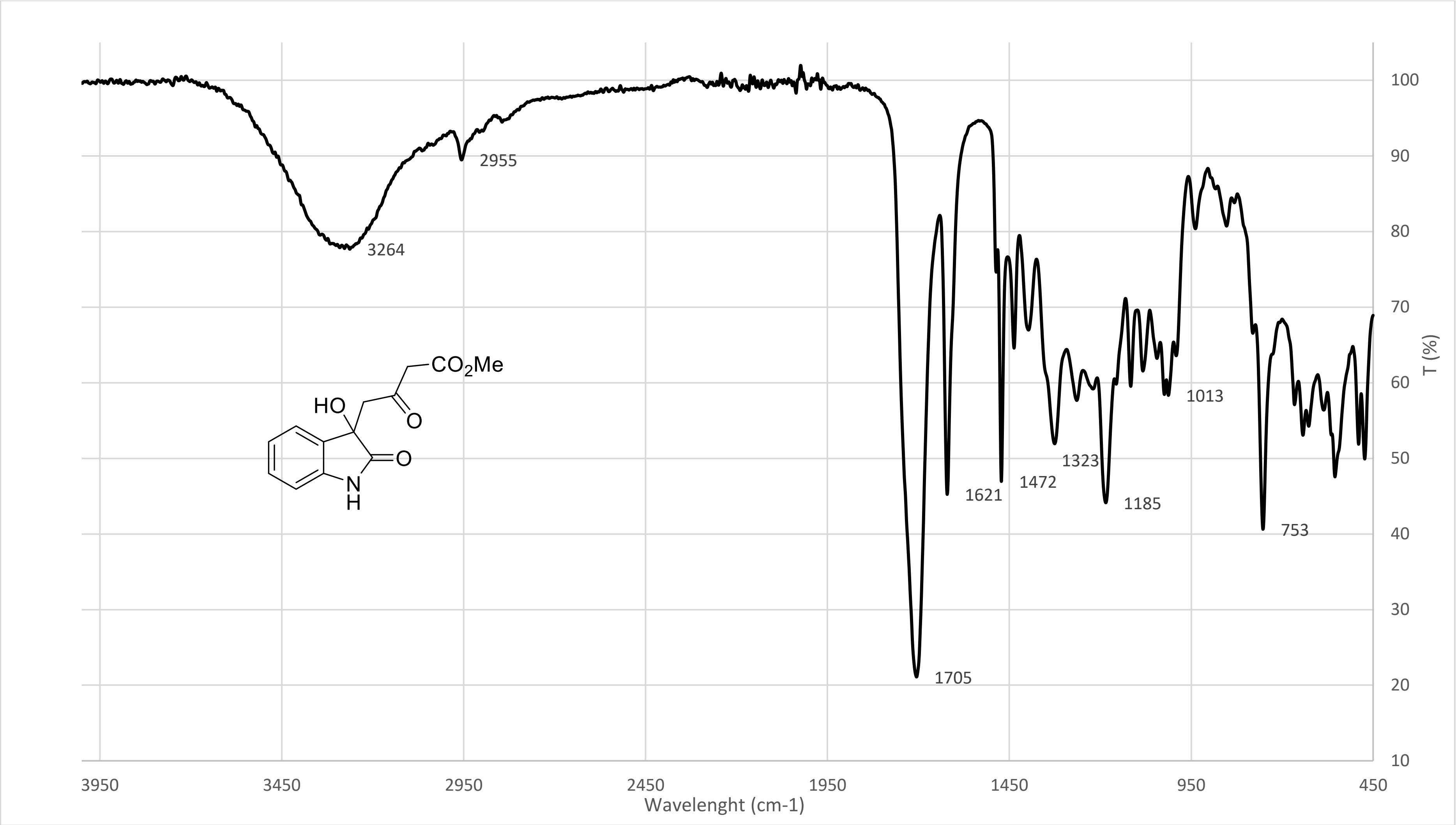

3264
2955
1705
1621
1472
1323
1185
1013
753
HO
CO2Me
O
O
N
H
T (%)
Wavelenght (cm-1)

Elemental Composition Report

Single Mass Analysis

Tolerance = 10.0 PPM / DBE: min = -1.5, max = 100.0
Element prediction: Off
Number of isotope peaks used for i-FIT = 5

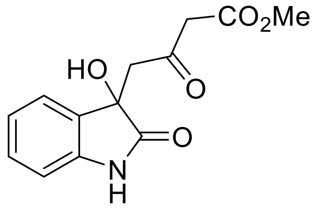

Monoisotopic Mass, Even Electron Ions
168 formula(e) evaluated with 2 results within limits (up to 500 closest results for each mass)
Elements Used:
C: 0-20 H: 0-40 N: 0-8 O: 0-5

QToF Premier

10-Dec-2019
GG314 244 (2.067) Cm (239:260)

1: TOF MS ES+
5.31e+005

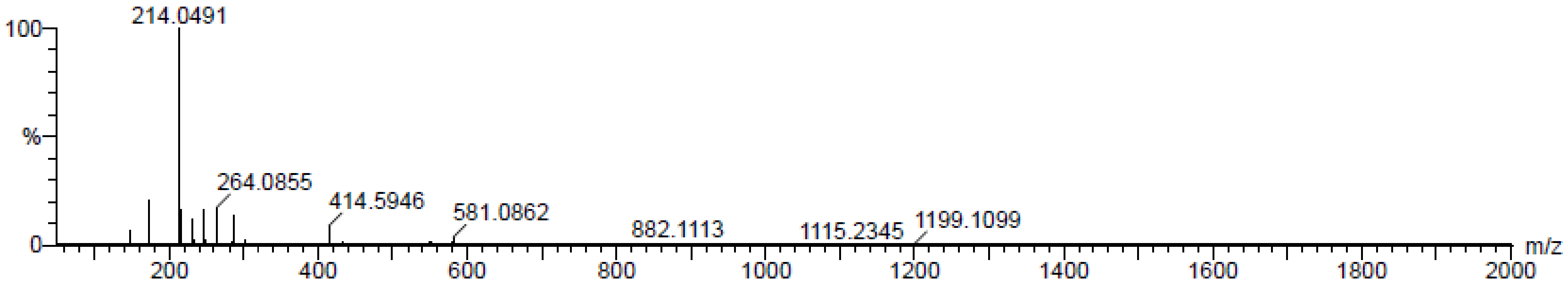

Minimum:				-1.5			
Maximum:		3.0	10.0	100.0			

Mass	Calc. Mass	mDa	PPM	DBE	i-FIT	i-FIT (Norm)	Formula
264.0855	264.0845	1.0	3.8	8.5	1014.8	4.9	C9 H10 N7 O3
	264.0872	-1.7	-6.4	7.5	1010.0	0.0	C13 H14 N O5

1.2 *Data of 3-hydroxy-3-((3-hydroxy-1H-pyrazol-5-yl)methyl)indolin-2-one (**15**)*

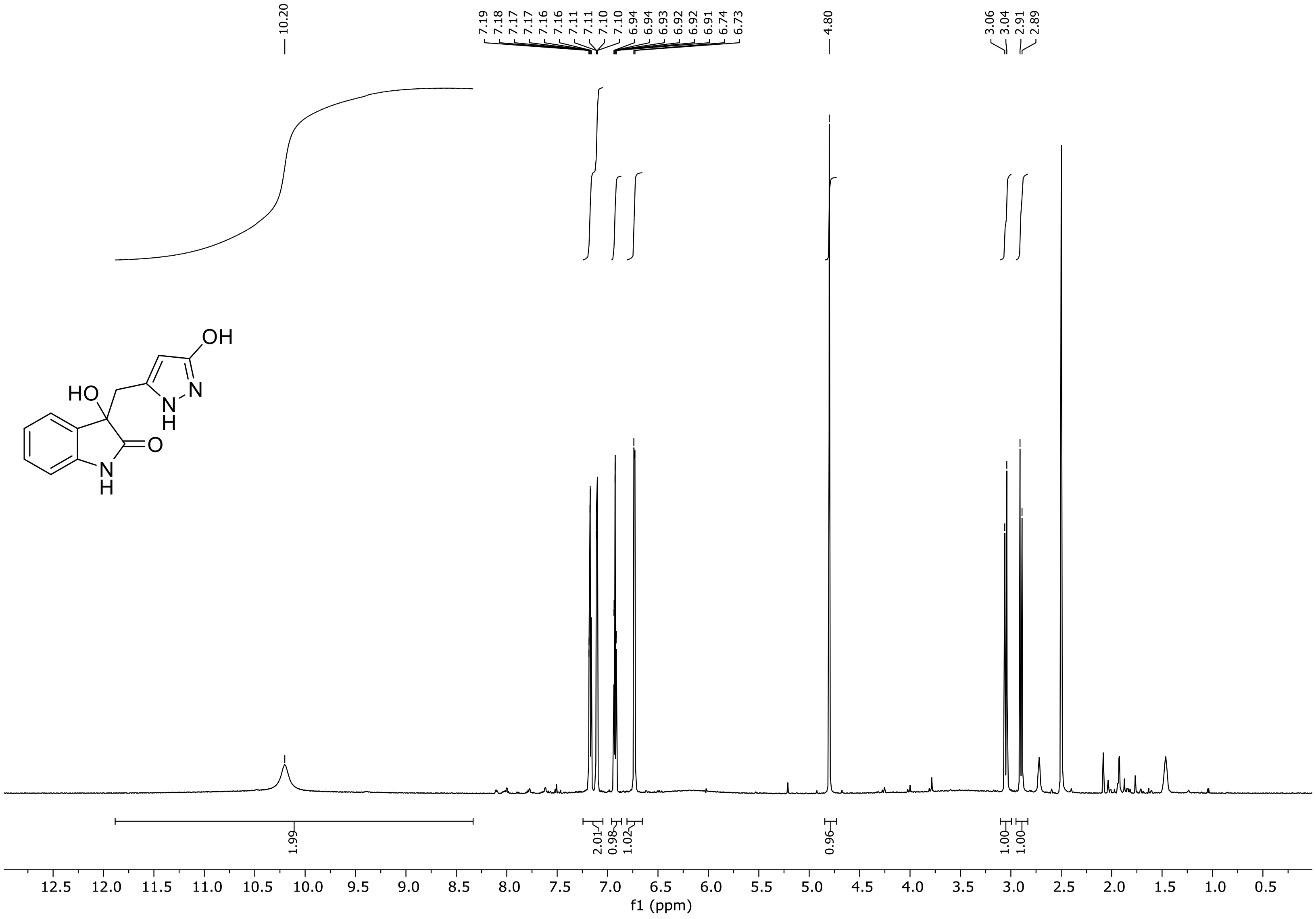

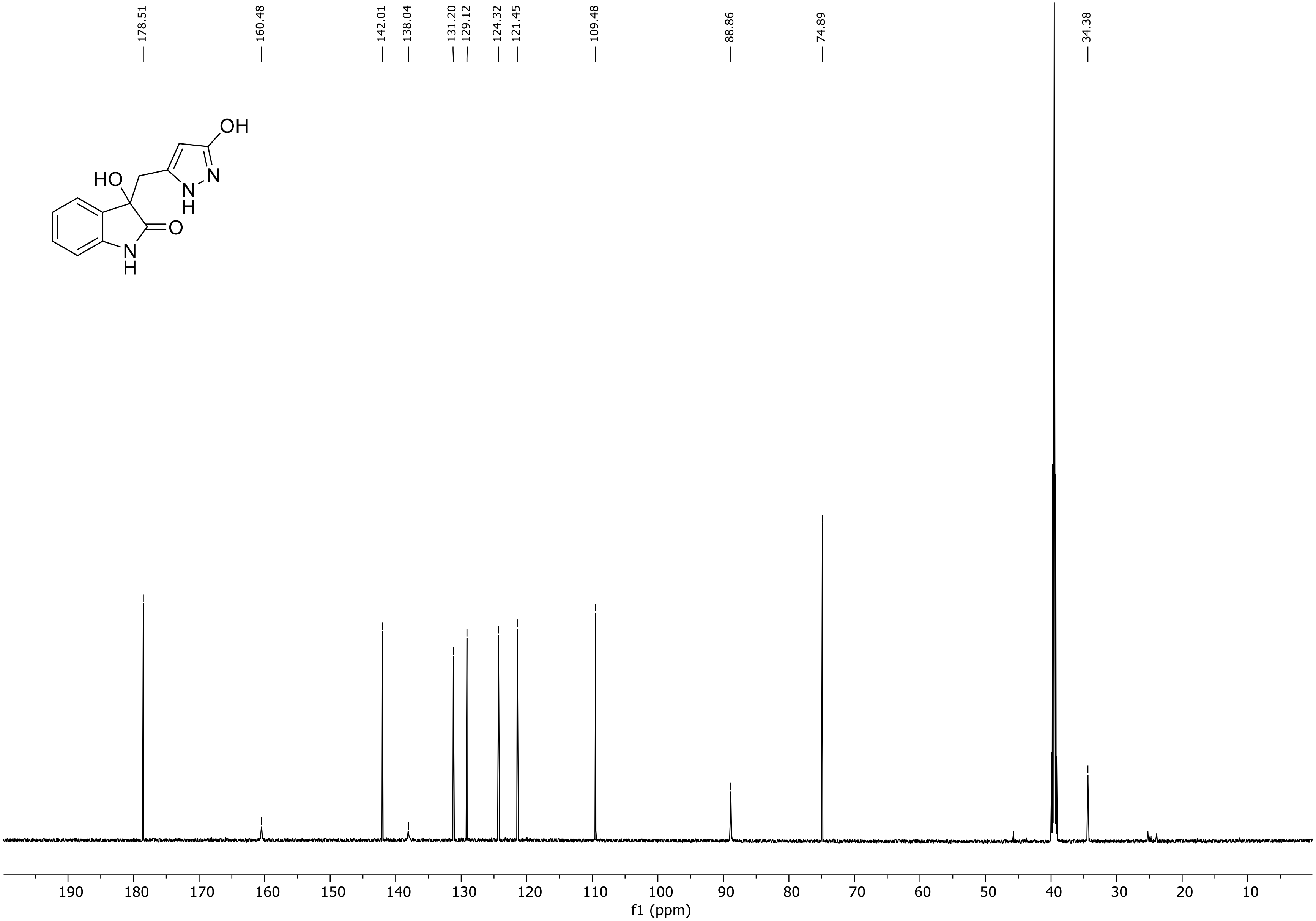
178.51
160.48
142.01
138.04
131.20
129.12
124.32
121.45
109.48
88.86
74.89
34.38
OH
HO
N
H
N
H
O
190 180 170 160 150 140 130 120 110 100 90 80 70 60 50 40 30 20 10
f1 (ppm)

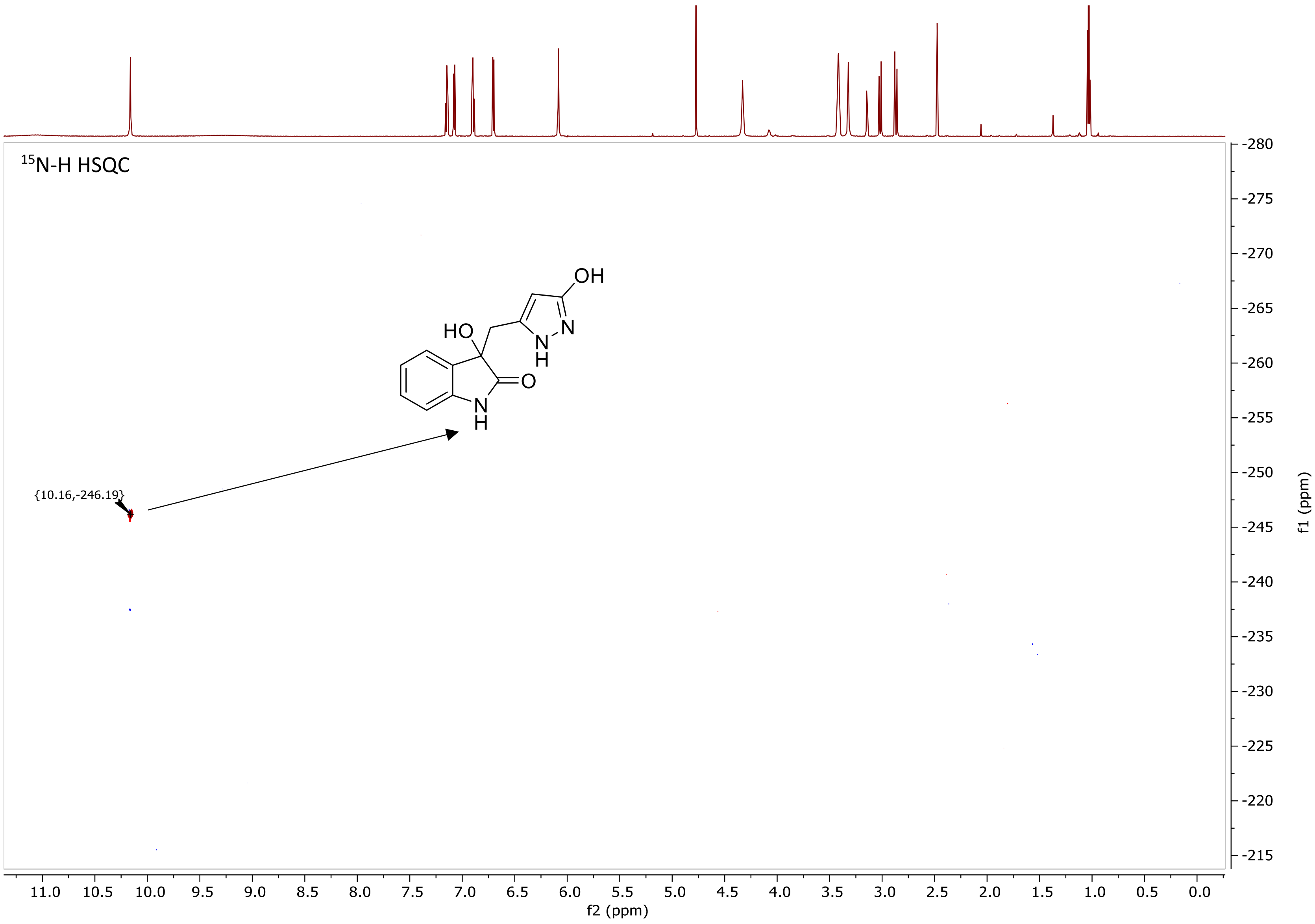

^{15}N-H HSQC
{10.16,-246.19}
HO
OH
N
N
H
O
N
H
f1 (ppm)
f2 (ppm)

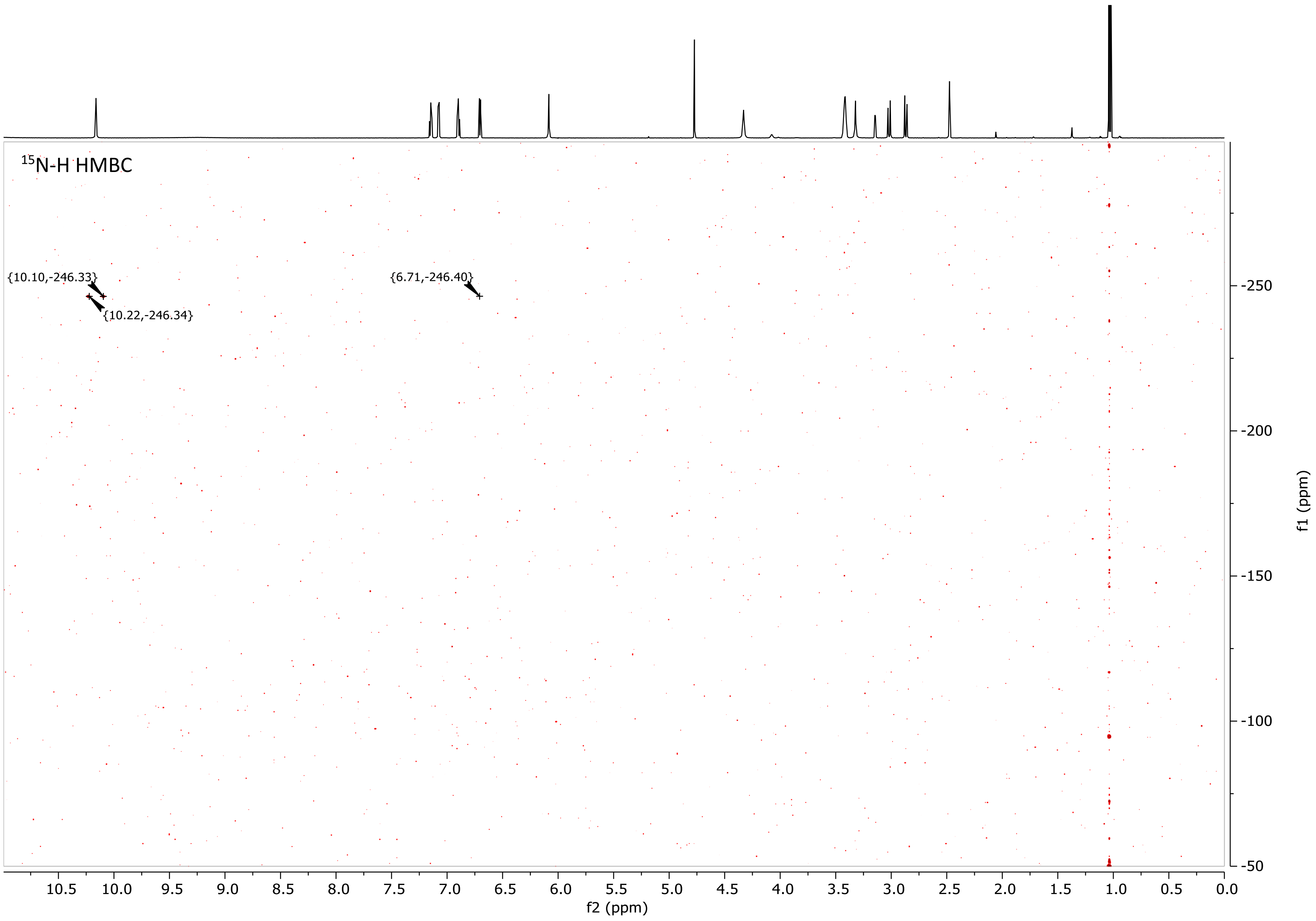

^{15}N-H HMBC
{10.10,-246.33}
{10.22,-246.34}
{6.71,-246.40}
f1 (ppm)
f2 (ppm)

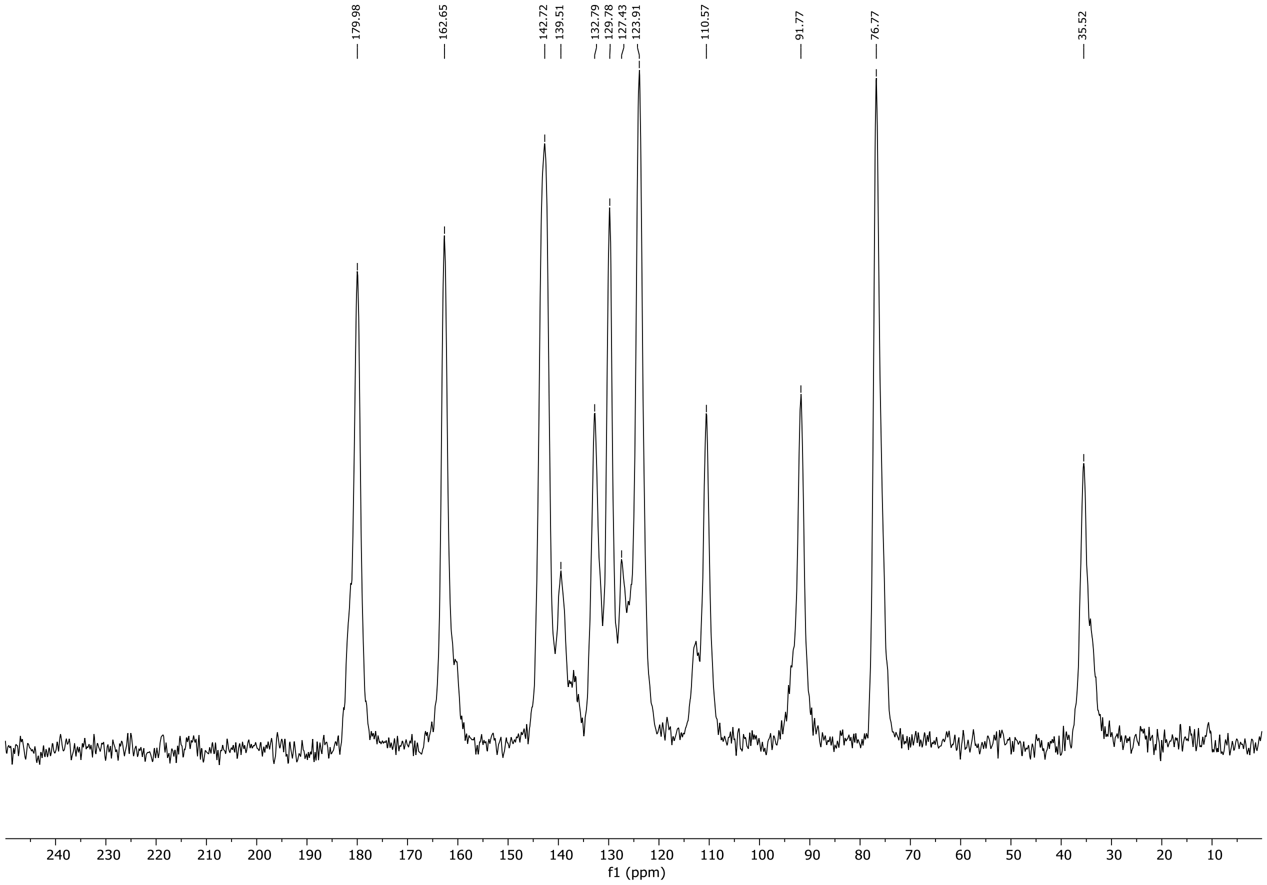

Solid-state carbon-13 NMR spectrum recorded with a 4 s recycle delay and 10 ms contact time.

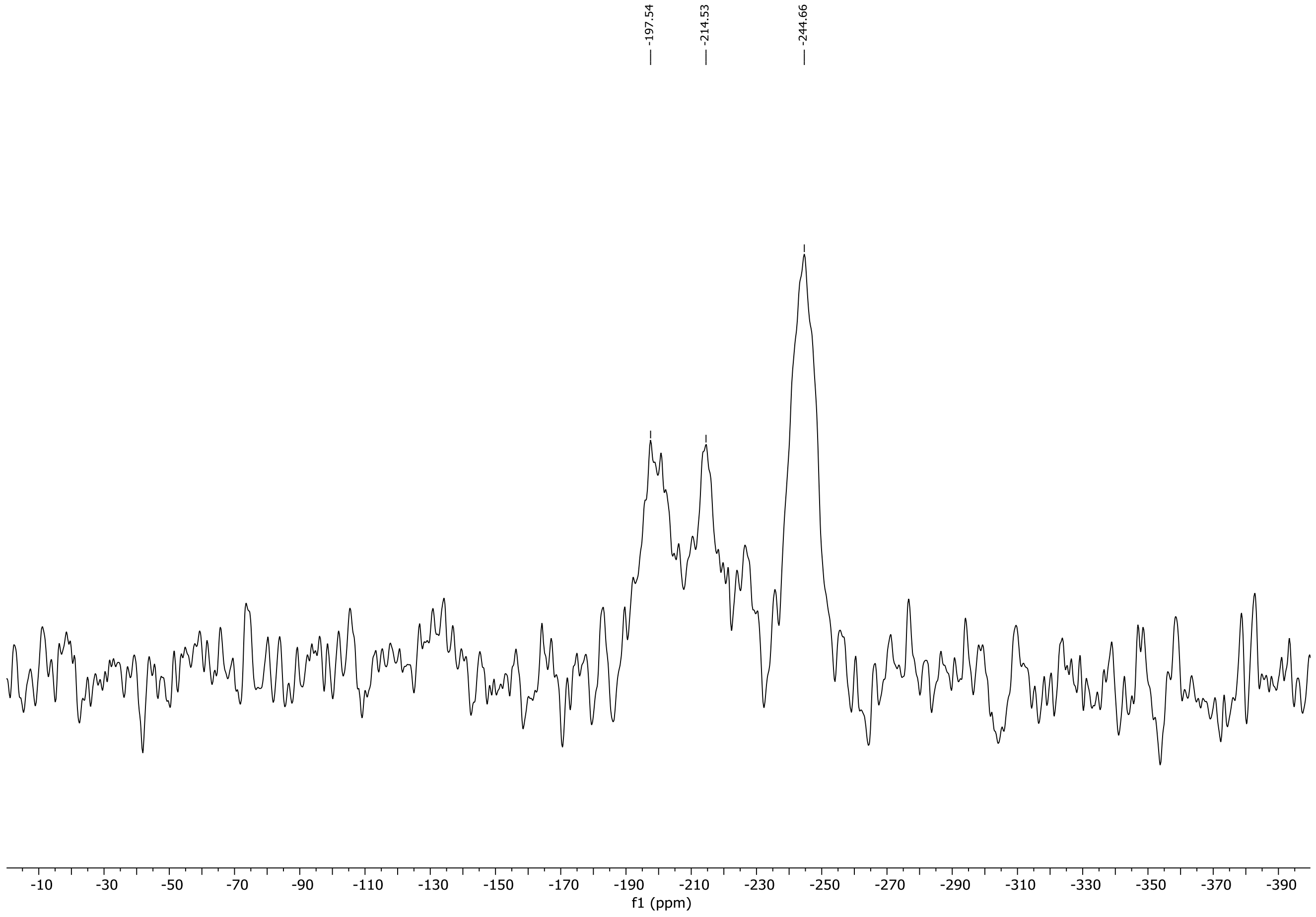

Solid-state nitrogen-15 NMR spectrum recorded with a 4 s recycle delay and 2 ms contact time.

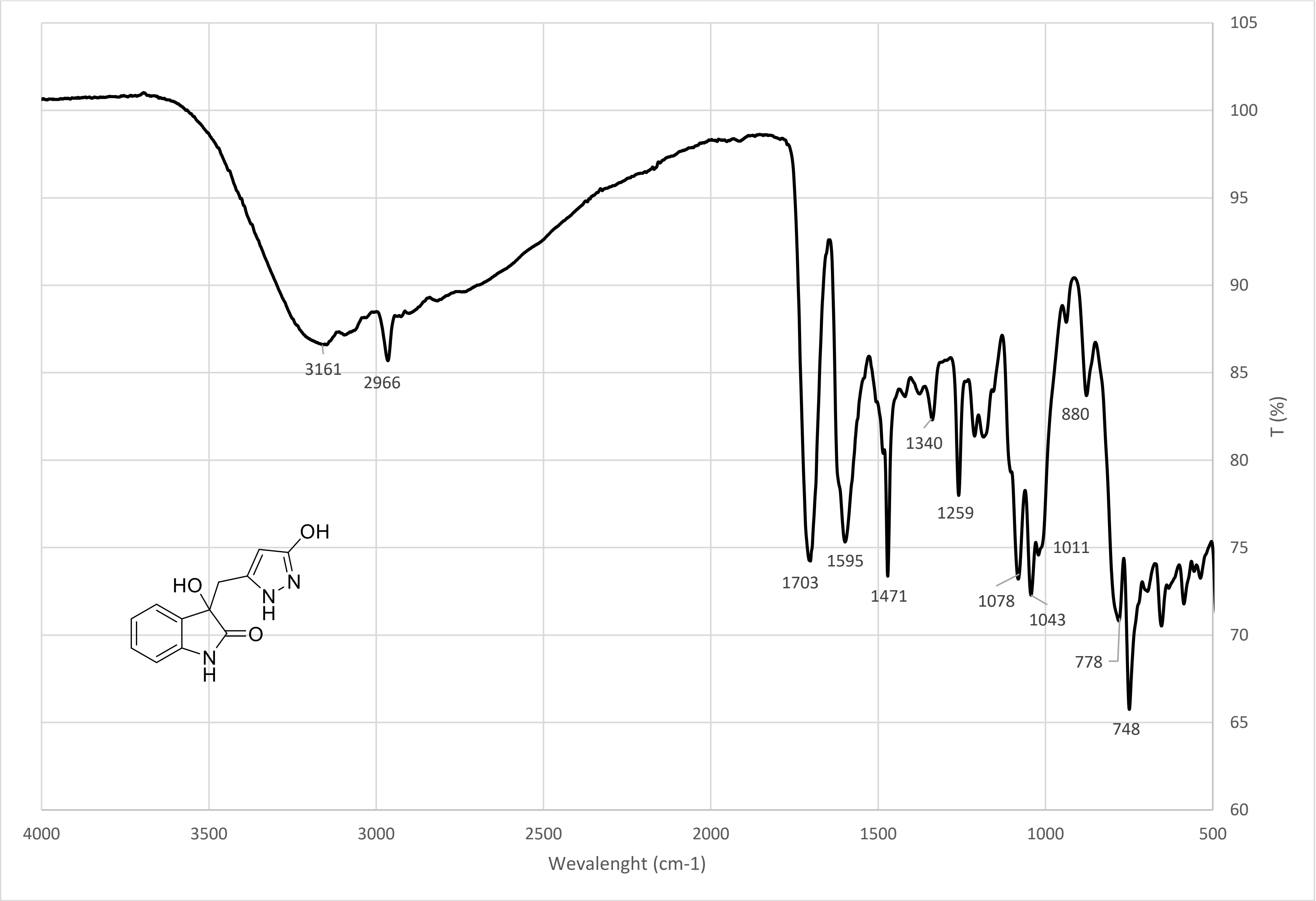

3161
2966
1703
1595
1471
1340
1259
1078
1043
1011
880
778
748
HO
OH
N
N
H
O
N
H
4000
3500
3000
2500
2000
1500
1000
500
Wevalenght (cm-1)
105
100
95
90
85
80
75
70
65
60
T (%)

Elemental Composition Report

Single Mass Analysis
Tolerance = 3.0 mDa / DBE: min = -1.5, max = 100.0
Element prediction: Off
Number of isotope peaks used for i-FIT = 5

Monoisotopic Mass, Even Electron Ions
325 formula(e) evaluated with 2 results within limits (up to 500 best isotopic matches for each mass)
Elements Used:
C: 0-50 H: 0-40 N: 0-5 O: 0-5 S: 0-2 191Ir: 0-1

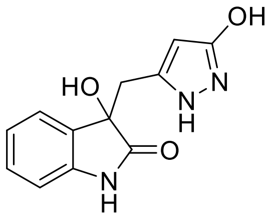

15-Oct-2018
GGI27L 181 (1.531) Cm (179:185)

1: TOF MS ES+
3.63e+005

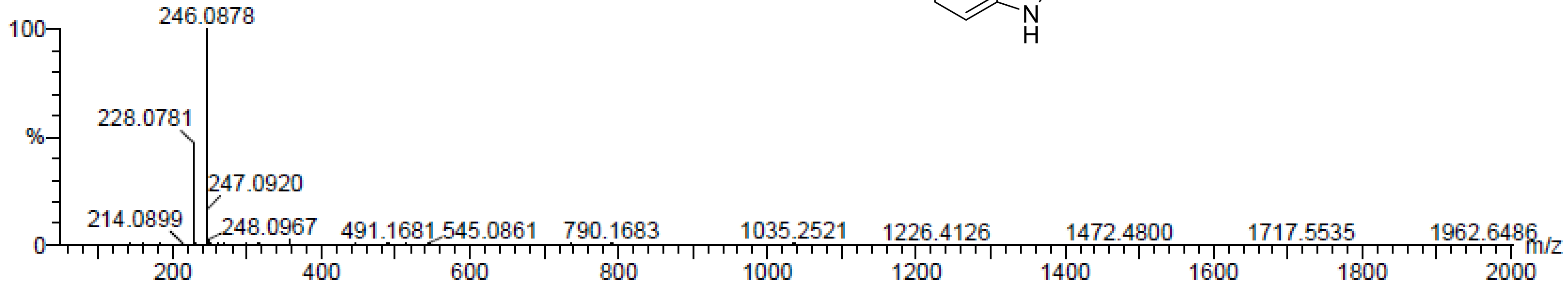

Mass	Calc. Mass	mDa	PPM	DBE	i-FIT	i-FIT (Norm)	Formula
Minimum:				-1.5			
Maximum:		3.0	5.0	100.0			
246.0878	246.0879	-0.1	-0.4	8.5	1073.4	0.0	C12 H12 N3 O3
	246.0872	0.6	2.4	-0.5	1085.5	12.1	C4 H16 N5 O5 S

1.3 Data of 4-fluoro-3-hydroxy-3-((3-hydroxy-1H-pyrazol-5-yl)methyl)indolin-2-one (**15a**)

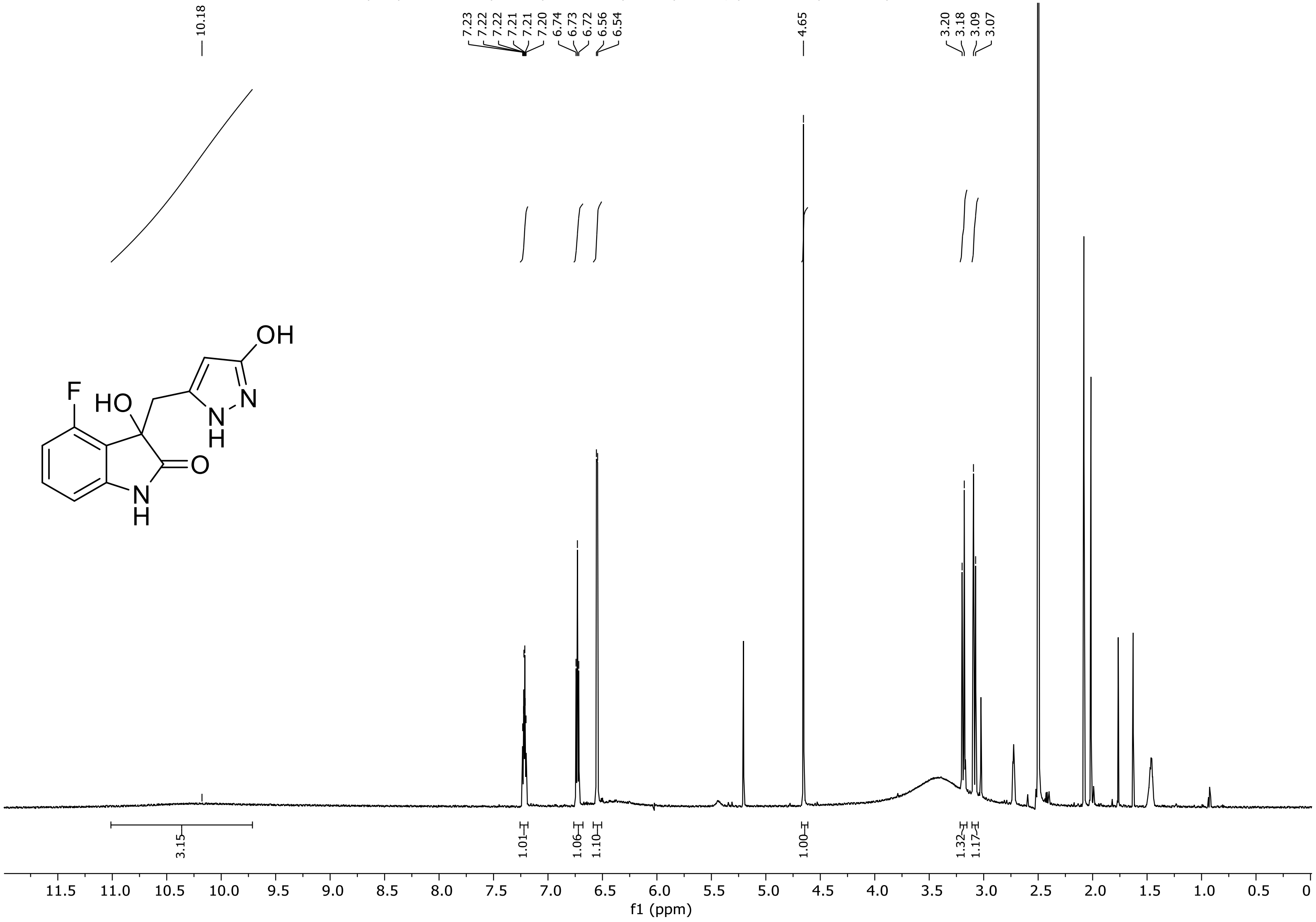

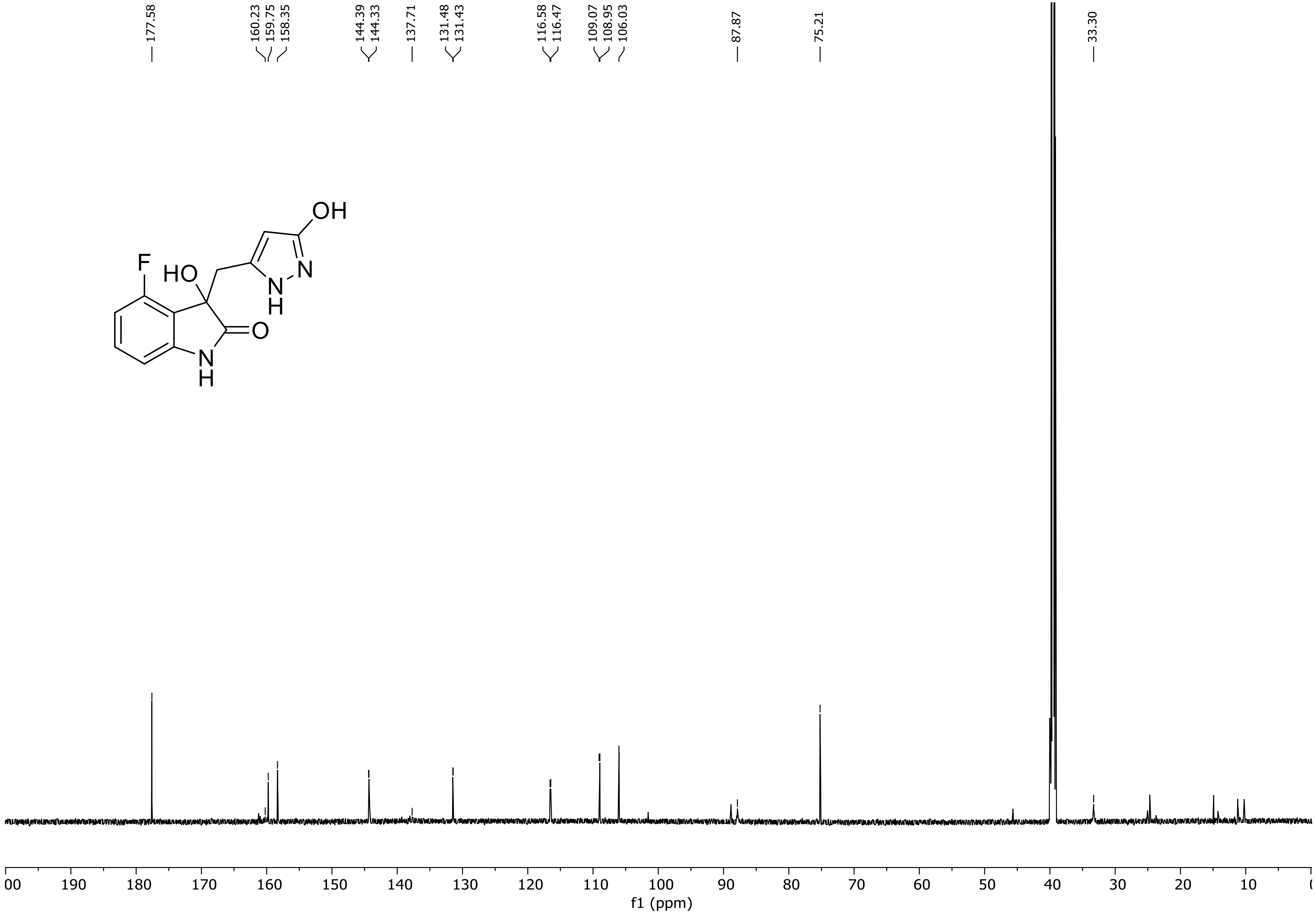
177.58
160.23
159.75
158.35
144.39
144.33
137.71
131.48
131.43
116.58
116.47
109.07
108.95
106.03
87.87
75.21
33.30
OH
F
HO
N
H
O
N
H
190
180
170
160
150
140
130
120
110
100
90
80
70
60
50
40
30
20
10
f1 (ppm)

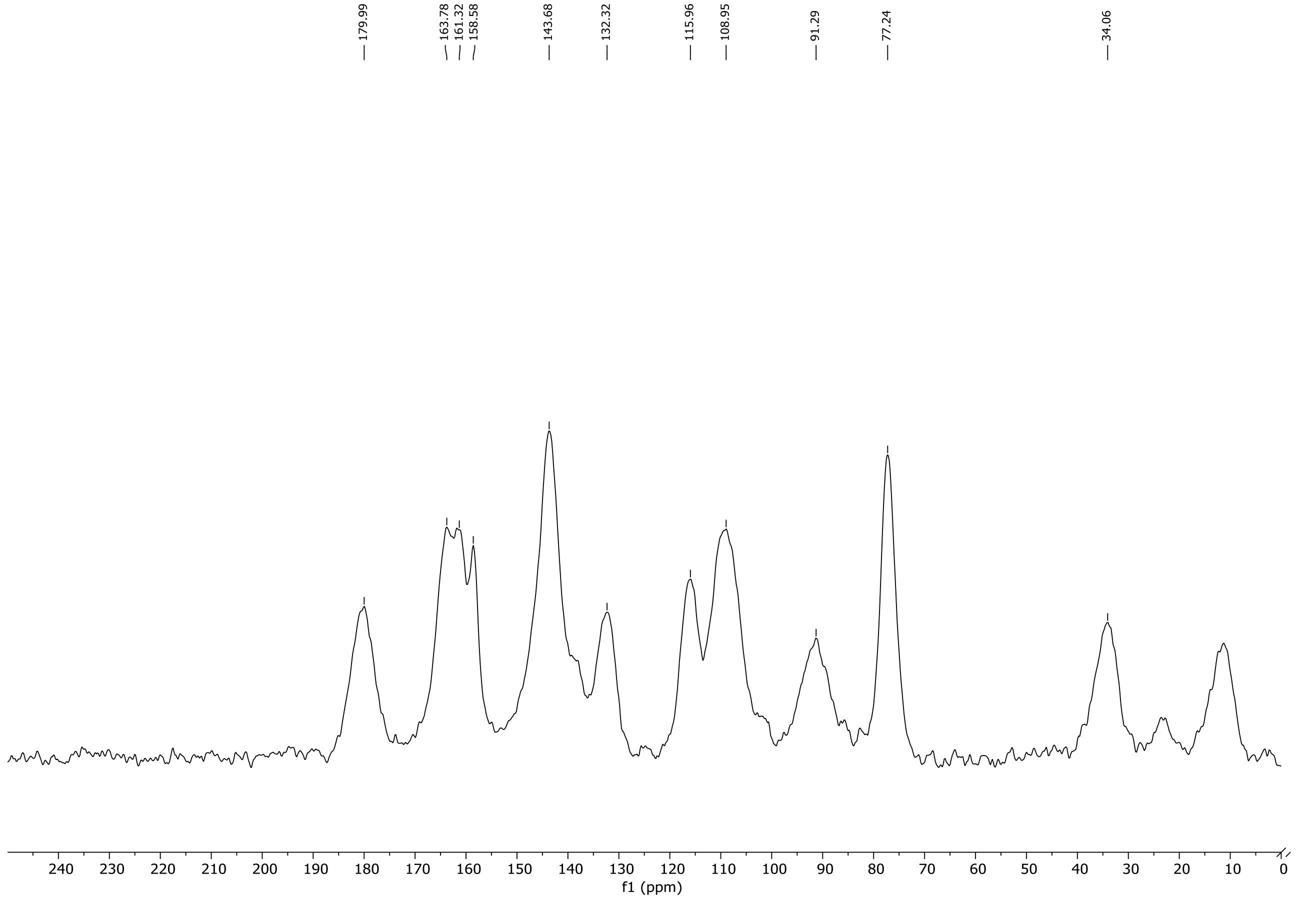

Solid-state carbon-13 NMR spectrum recorded with a 2.5 s recycle delay and 1 ms contact time.

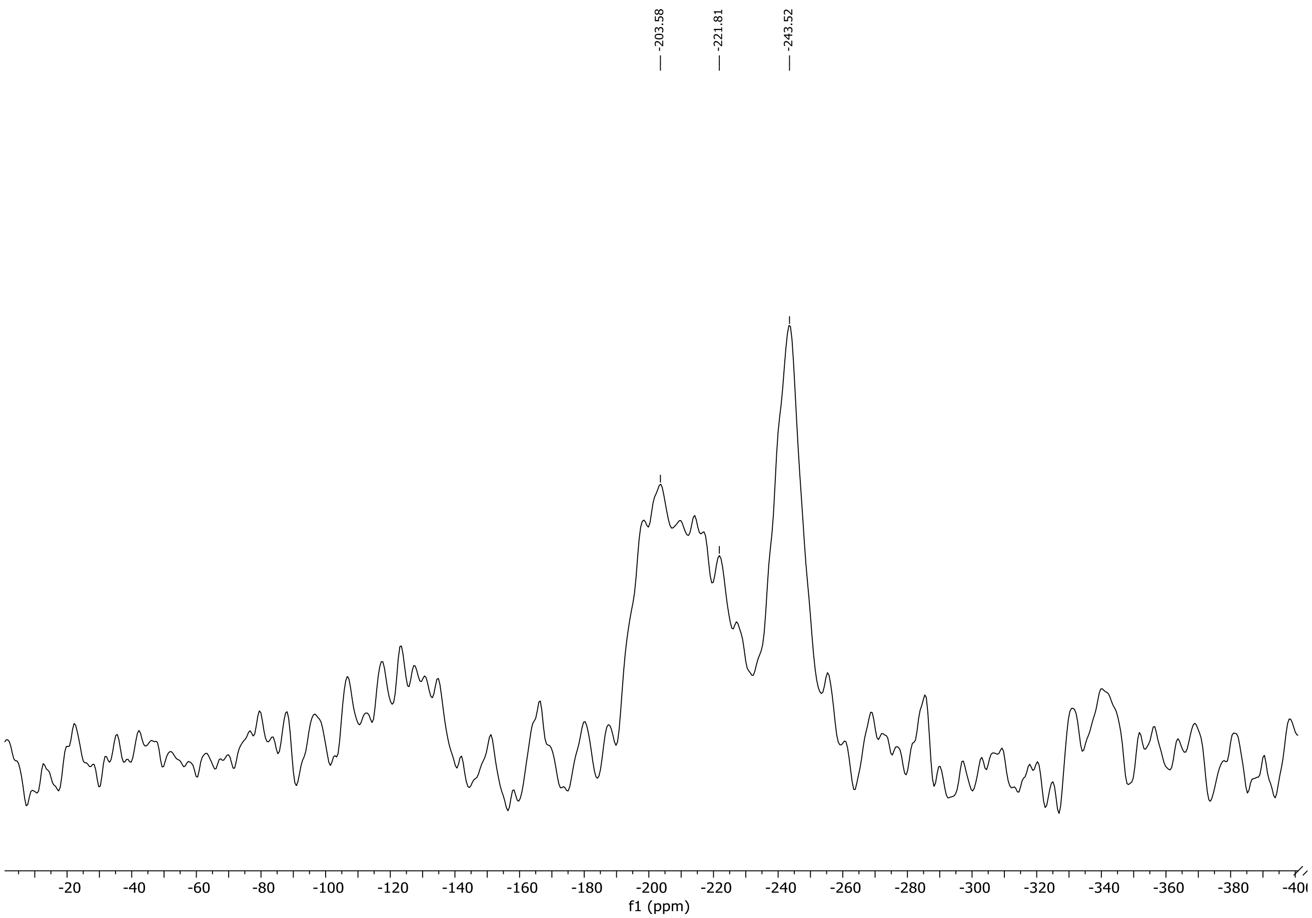

Solid-state nitrogen-15 NMR spectrum recorded with a 2.5 s recycle delay and 4 ms contact time.

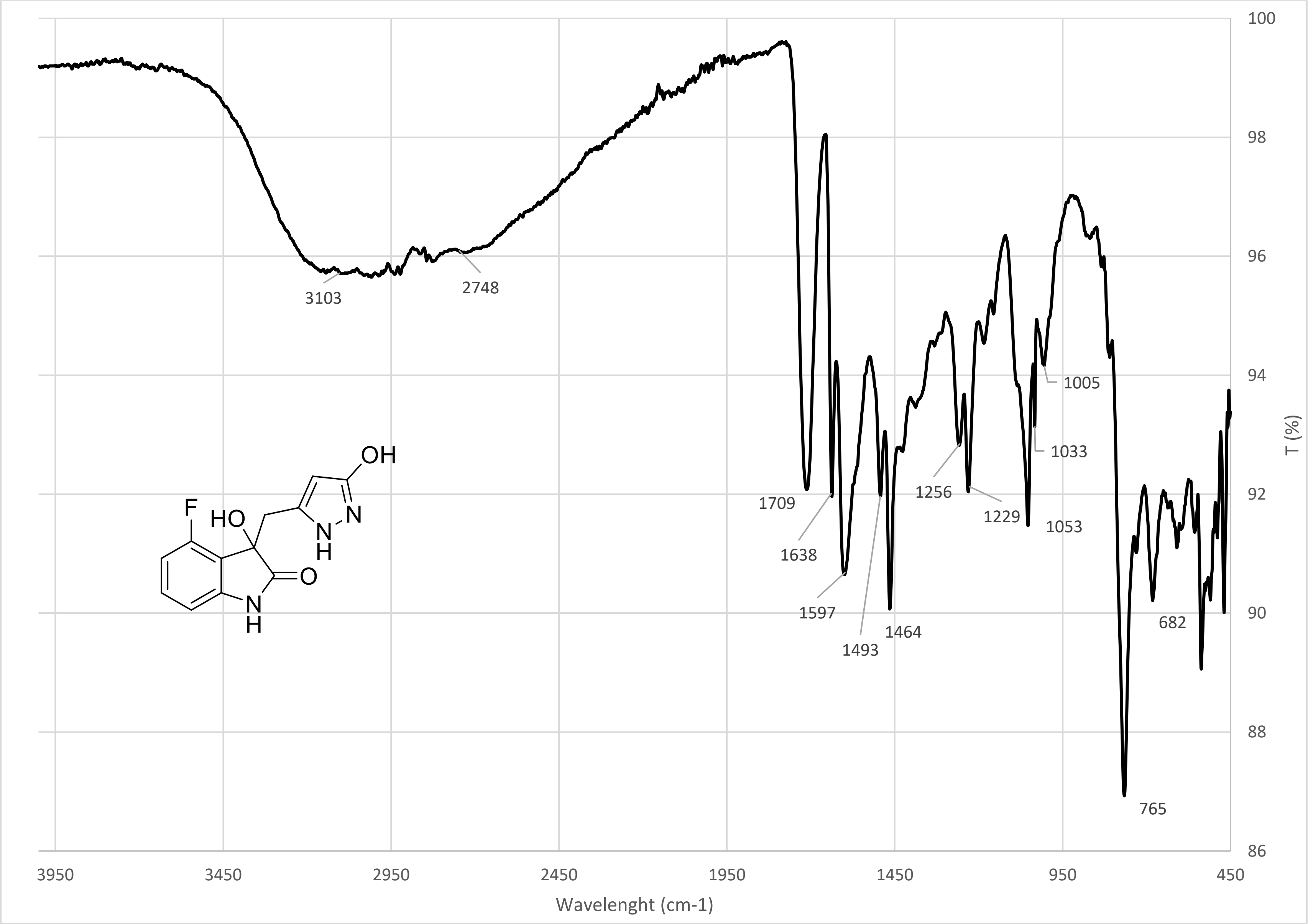
3103
2748
1709
1638
1597
1493
1464
1256
1229
1053
1033
1005
765
682
100
98
96
94
92
90
88
86
T (%)
3950
3450
2950
2450
1950
1450
950
450
Wavelenght (cm-1)
F
HO
OH
N
N
H
O
N
H

Single Mass Analysis

Tolerance = 5.0 mDa / DBE: min = -1.5, max = 100.0
Element prediction: Off
Number of isotope peaks used for i-FIT = 5

Monoisotopic Mass, Even Electron Ions
505 formula(e) evaluated with 8 results within limits (up to 500 closest results for each mass)
Elements Used:
C: 0-50 H: 0-80 N: 0-6 O: 0-4 F: 0-4

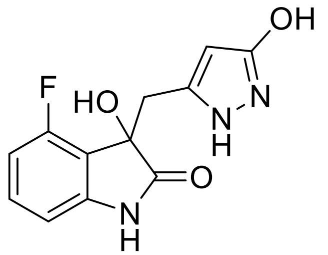

QToF Premier

15-Nov-2019
GG302A 164 (1.387) Cm (163:164)

1: TOF MS ES+
5.90e+004

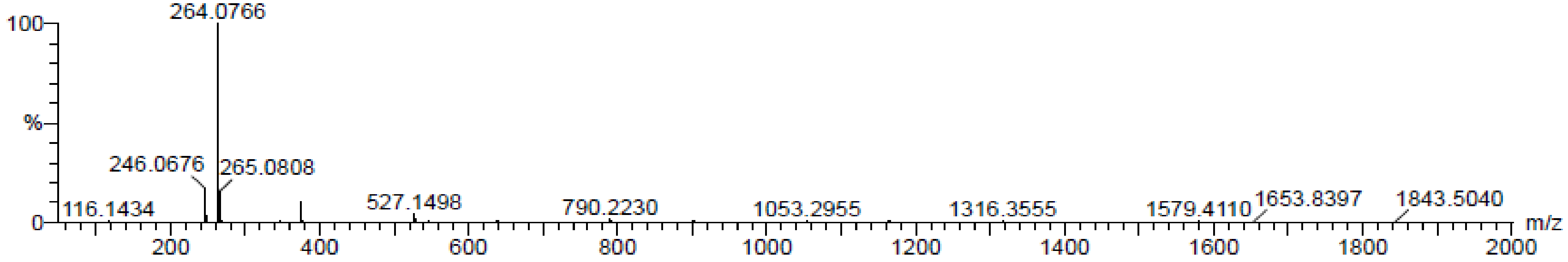

Mass	Calc. Mass	mDa	PPM	DBE	i-FIT	i-FIT (Norm)	Formula
Minimum:				-1.5			
Maximum:		5.0	5.0	100.0			
264.0766	264.0760	0.6	2.3	5.5	703.6	9.1	C10 H10 N3 O F4
	264.0773	-0.7	-2.7	12.5	700.8	6.3	C15 H10 N3 O2
	264.0749	1.7	6.4	9.5	700.1	5.6	C13 H9 N3 F3
	264.0784	-1.8	-6.8	8.5	694.5	0.0	C12 H11 N3 O3 F
	264.0796	-3.0	-11.4	4.5	703.6	9.1	C9 H12 N3 O4 F2
	264.0733	3.3	12.5	8.5	699.8	5.3	C10 H10 N5 O4
	264.0720	4.6	17.4	1.5	707.7	13.3	C5 H10 N5 O3 F4
	264.0813	-4.7	-17.8	16.5	706.2	11.7	C20 H10 N

*1.4 Data of 4-chloro-3-hydroxy-3-((3-hydroxy-1H-pyrazol-5-yl)methyl)indolin-2-one (**15b**)*

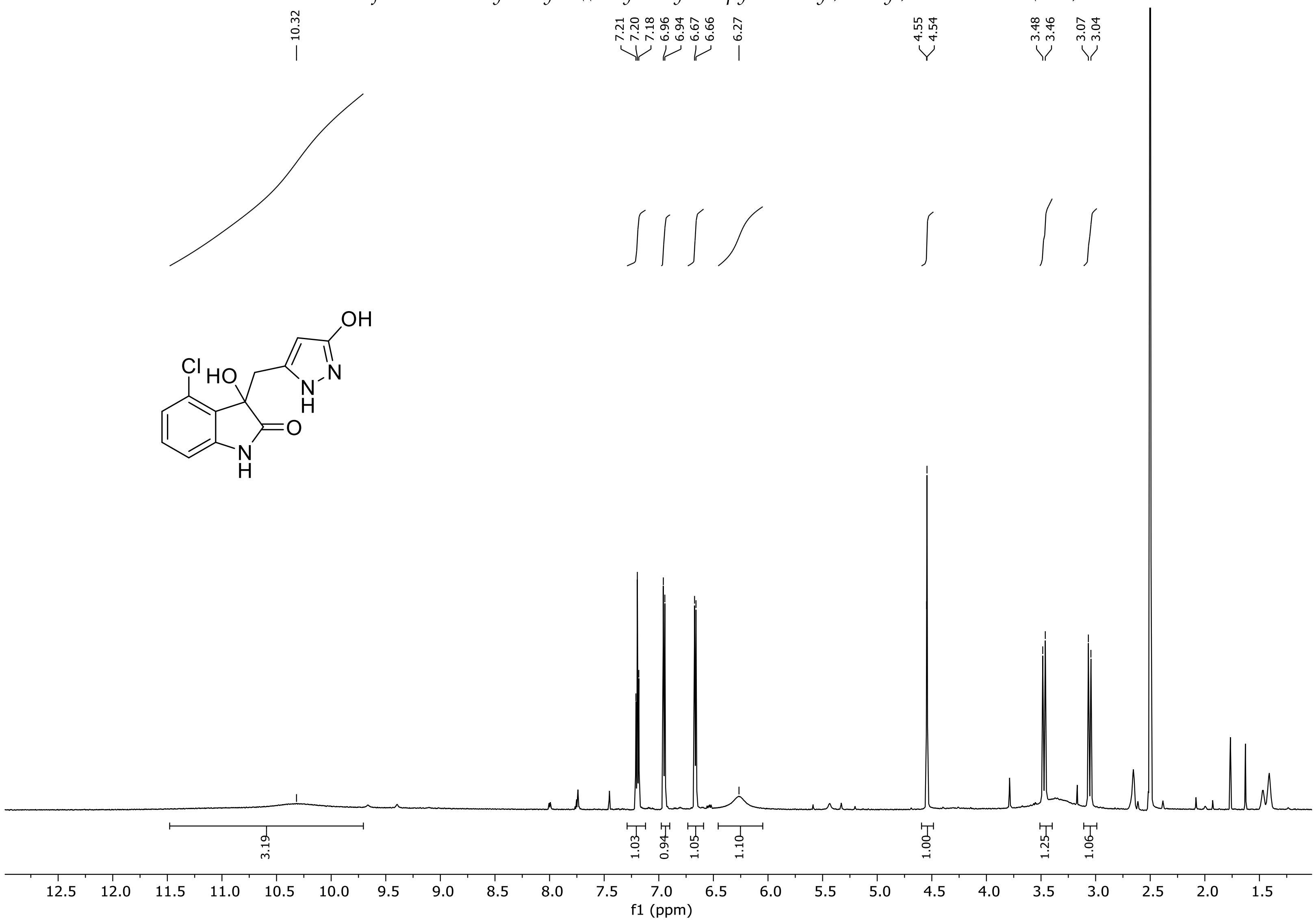

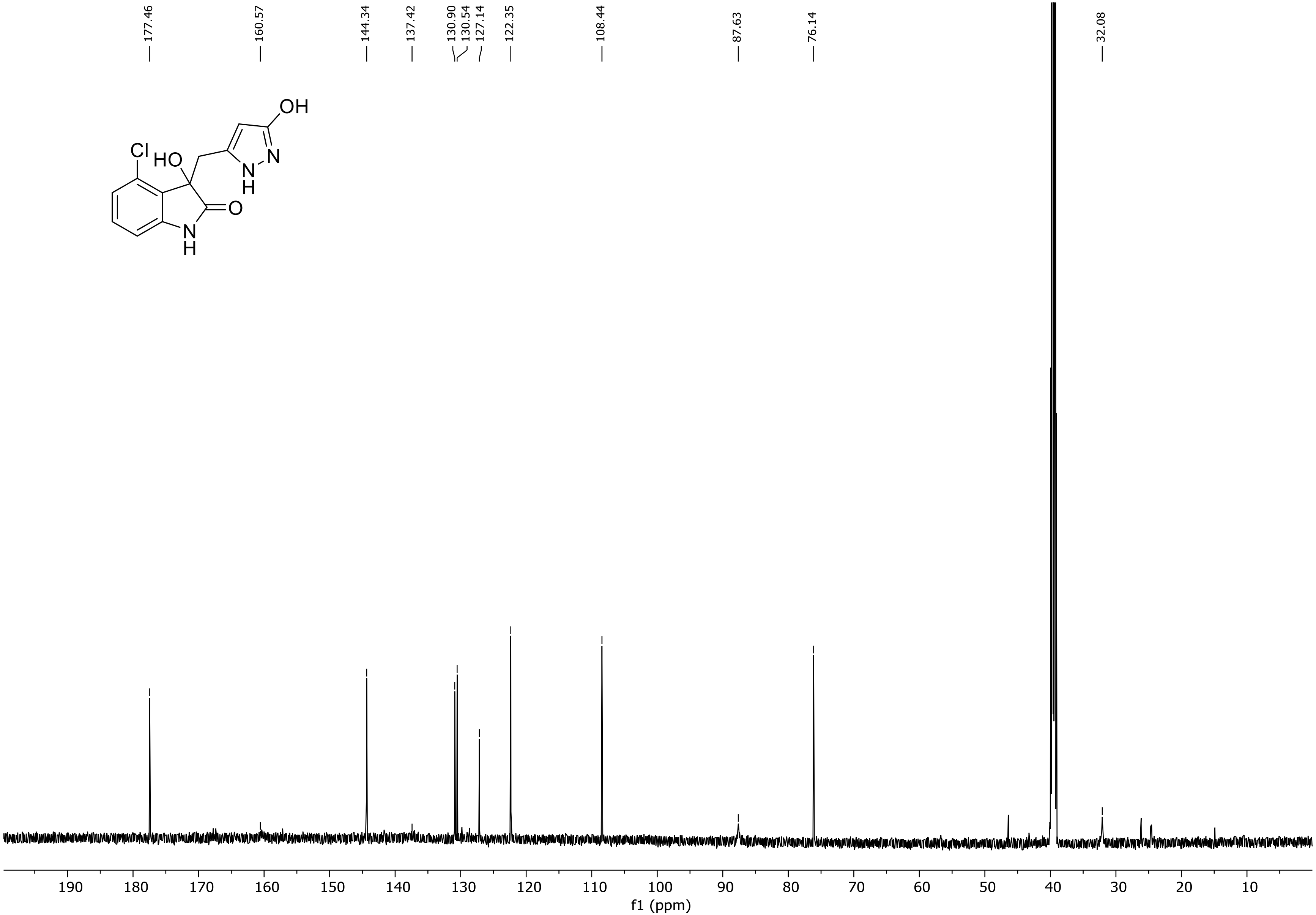
177.46
160.57
144.34
137.42
130.90
130.54
127.14
122.35
108.44
87.63
76.14
32.08
OH
Cl
HO
N
N
H
O
N
H
190
180
170
160
150
140
130
120
110
100
90
80
70
60
50
40
30
20
10
f1 (ppm)

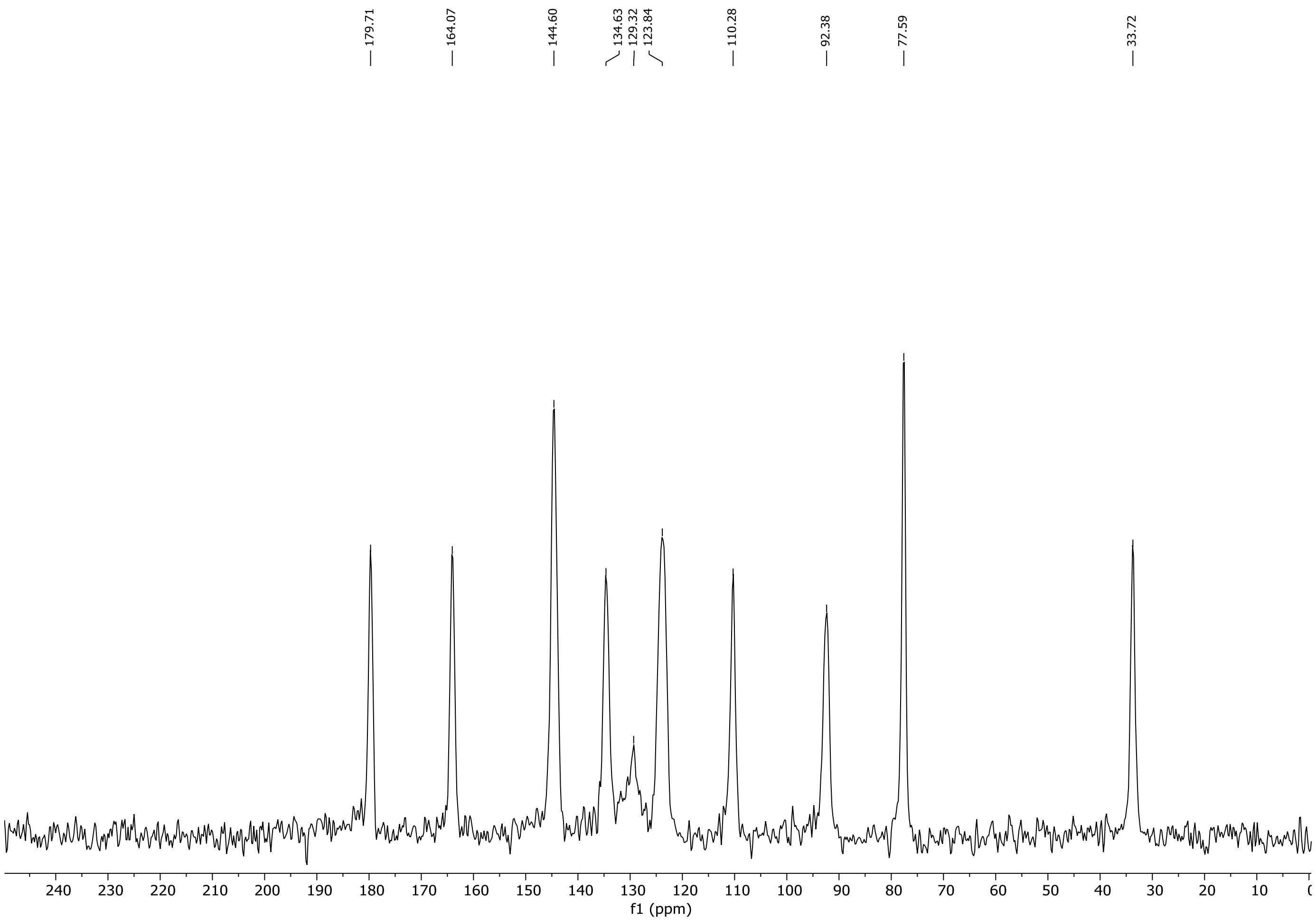

Solid-state carbon-13 NMR spectrum recorded with a 45 s recycle delay and 7 ms contact time.

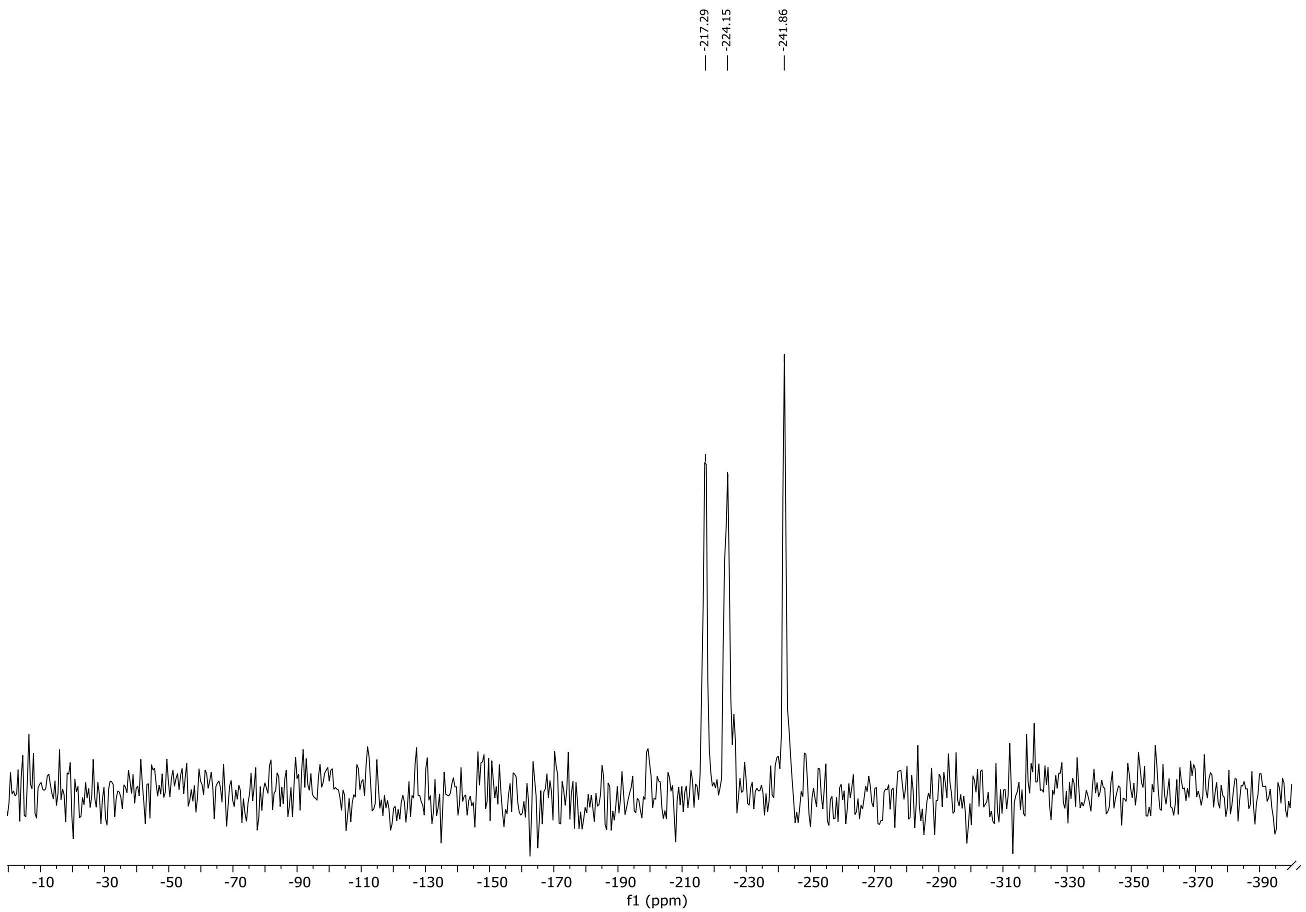

Solid-state nitrogen-15 NMR spectrum recorded with a 45 s recycle delay and 10 ms contact time.

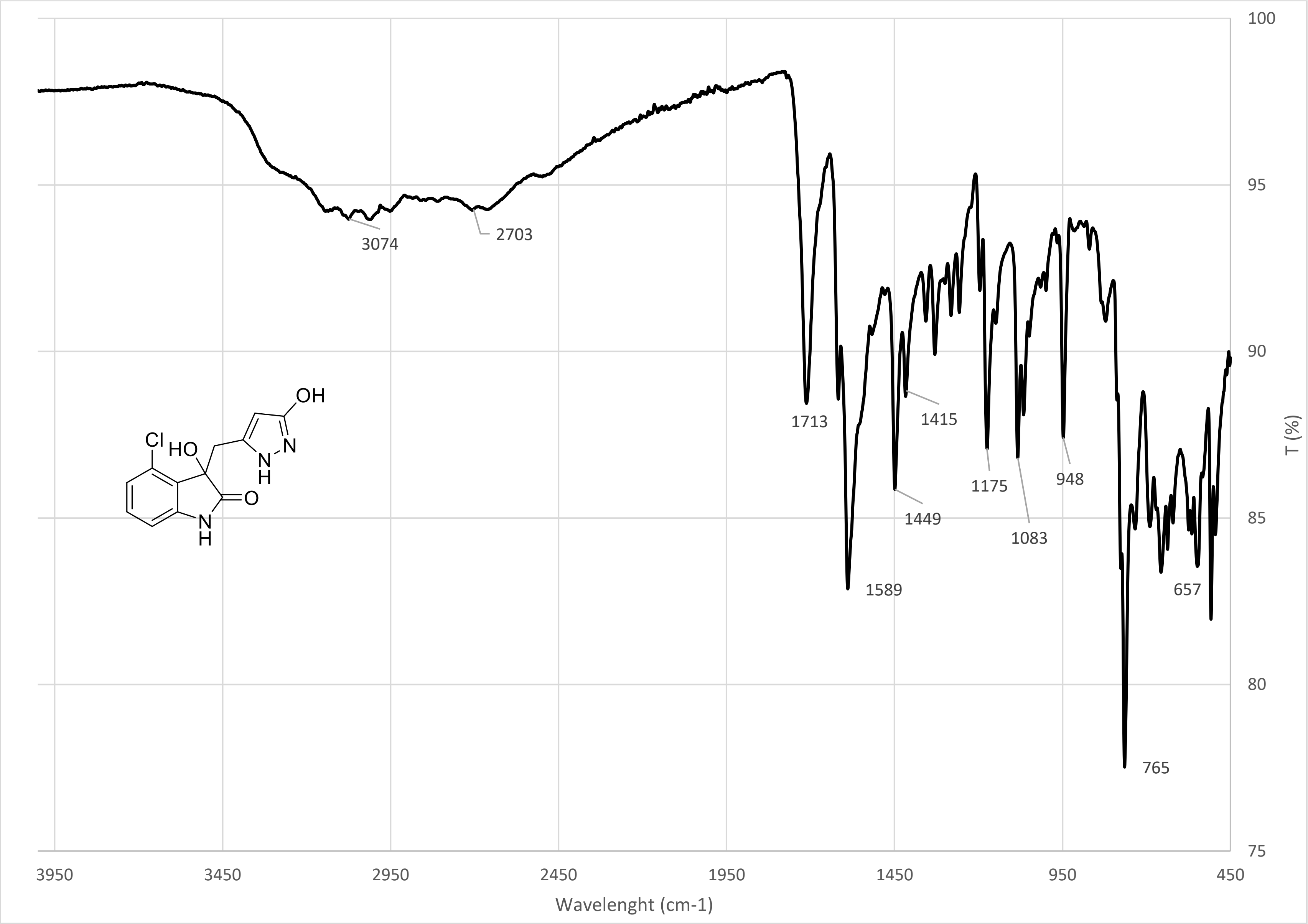

3074
2703
1713
1415
1589
1449
1175
1083
948
765
657
Cl
HO
OH
N
N
H
O
NH
100
95
90
85
80
75
3950
3450
2950
2450
1950
1450
950
450
Wavelenght (cm-1)
T (%)

Elemental Composition Report

Single Mass Analysis

Tolerance = 3.0 mDa / DBE: min = -1.5, max = 100.0
Element prediction: Off
Number of isotope peaks used for i-FIT = 5

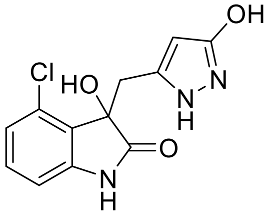

Monoisotopic Mass, Even Electron Ions
536 formula(e) evaluated with 6 results within limits (up to 500 closest results for each mass)
Elements Used:
C: 0-40 H: 0-80 N: 0-6 O: 0-6 Cl: 0-3

QToF Premier

18-Oct-2019
GG291 186 (1.570) Cm (183:190)

1: TOF MS ES+
1.70e+005

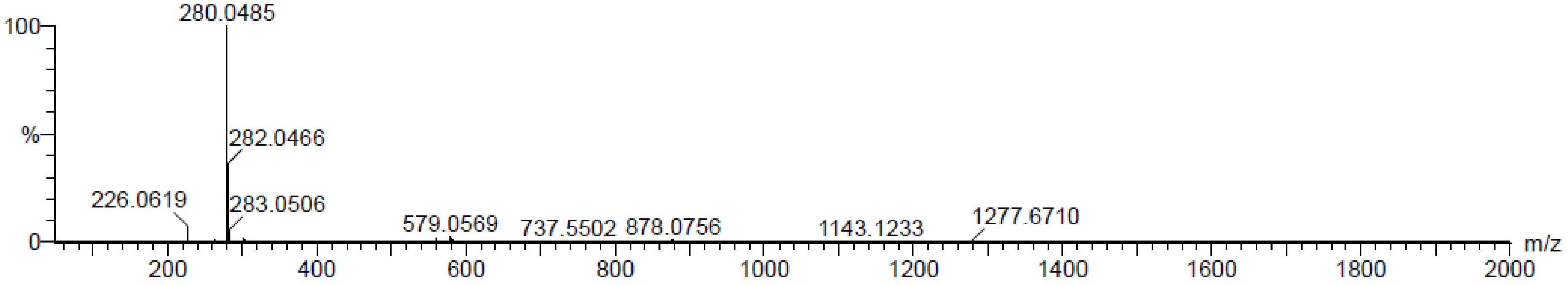

Minimum:				-1.5			
Maximum:		3.0	5.0	100.0			

Mass	Calc. Mass	mDa	PPM	DBE	i-FIT	i-FIT (Norm)	Formula
280.0485	280.0489	-0.4	-1.4	8.5	1215.6	0.0	C12 H11 N3 O3 Cl
	280.0499	-1.4	-5.0	-0.5	1239.1	23.6	C6 H17 N5 O Cl3
	280.0471	1.4	5.0	13.5	1235.6	20.0	C13 H6 N5 O3
	280.0467	1.8	6.4	-0.5	1236.7	21.2	C6 H16 N3 O5 Cl2
	280.0507	-2.2	-7.9	3.5	1236.5	20.9	C11 H16 N O3 Cl2
	280.0511	-2.6	-9.3	17.5	1236.4	20.9	C18 H6 N3 O

*1.5 Data of 4-bromo-3-hydroxy-3-((3-hydroxy-1H-pyrazol-5-yl)methyl)indolin-2-one (**15c**)*

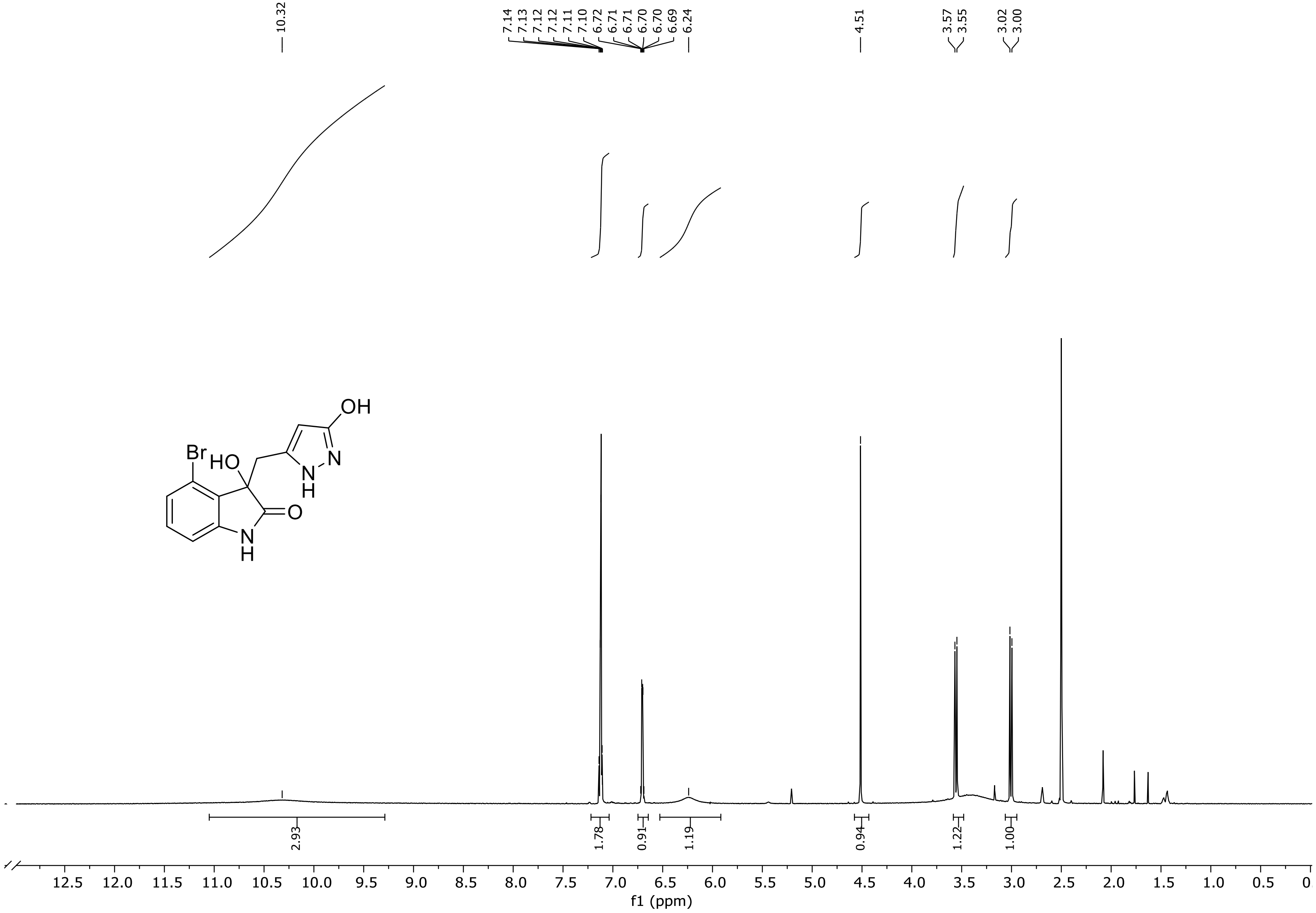

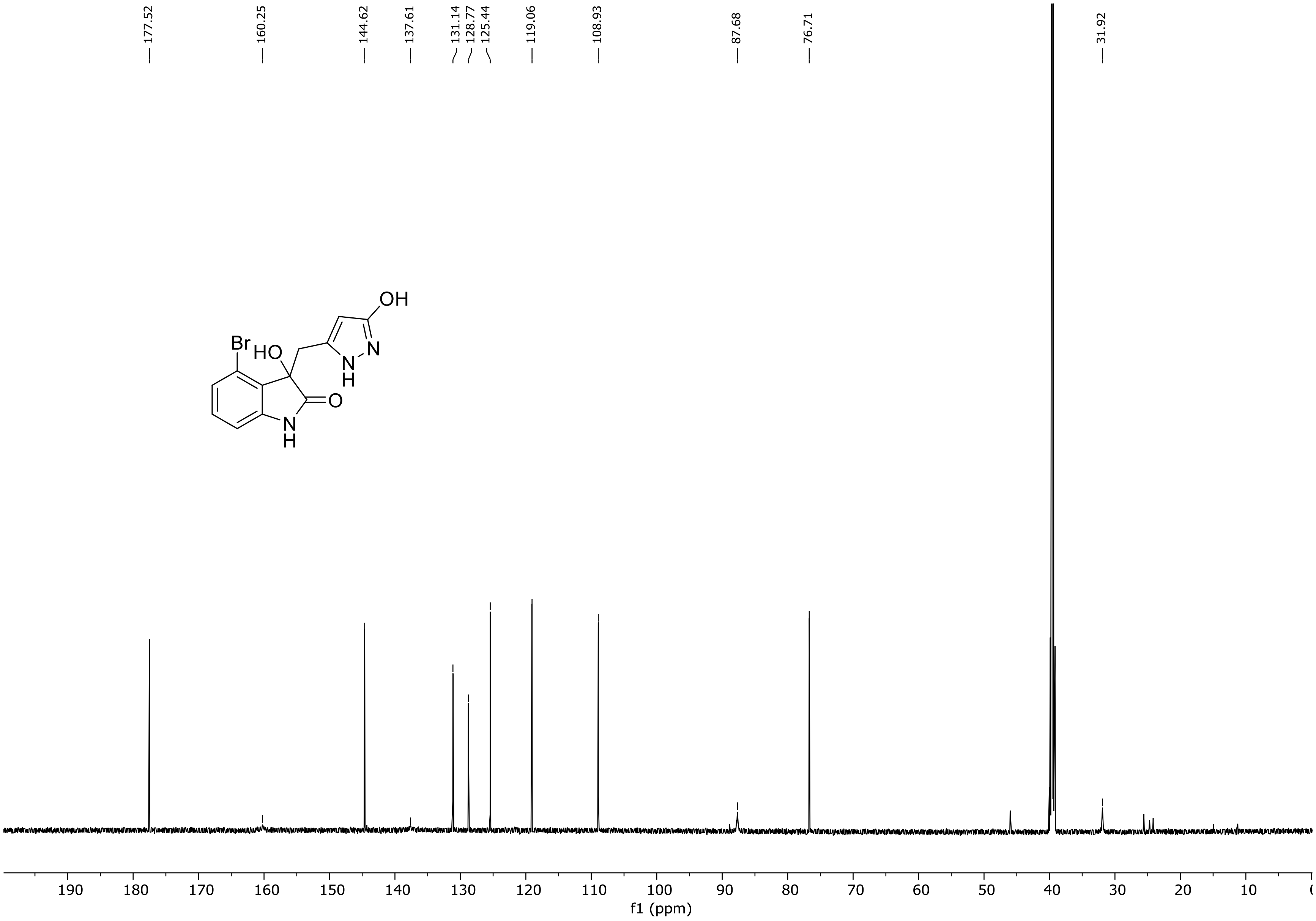
177.52
160.25
144.62
137.61
131.14
128.77
125.44
119.06
108.93
87.68
76.71
31.92
Br
HO
OH
N
N
H
N
H
O
190
180
170
160
150
140
130
120
110
100
90
80
70
60
50
40
30
20
10
f1 (ppm)

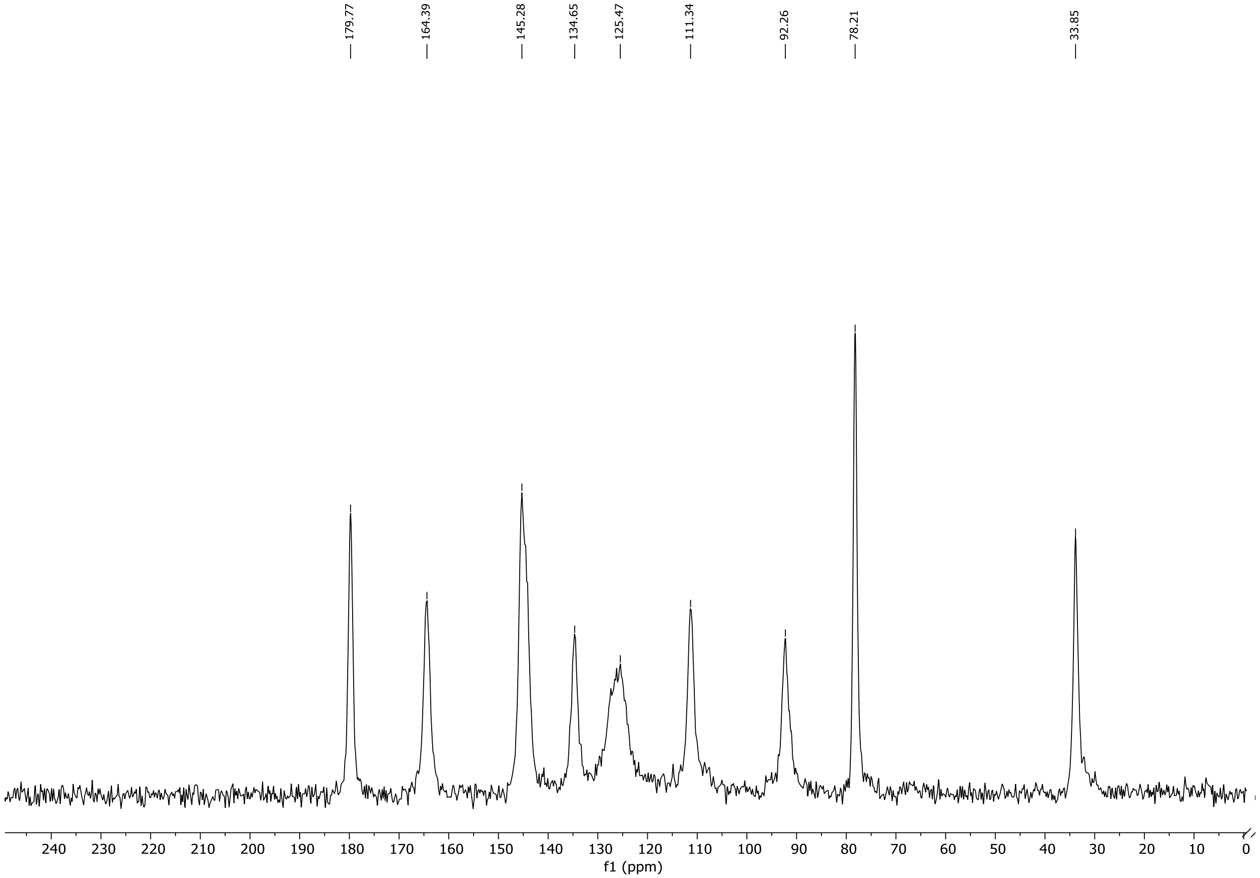

Solid-state carbon-13 NMR spectrum recorded with a 40 s recycle delay and 10 ms contact time.

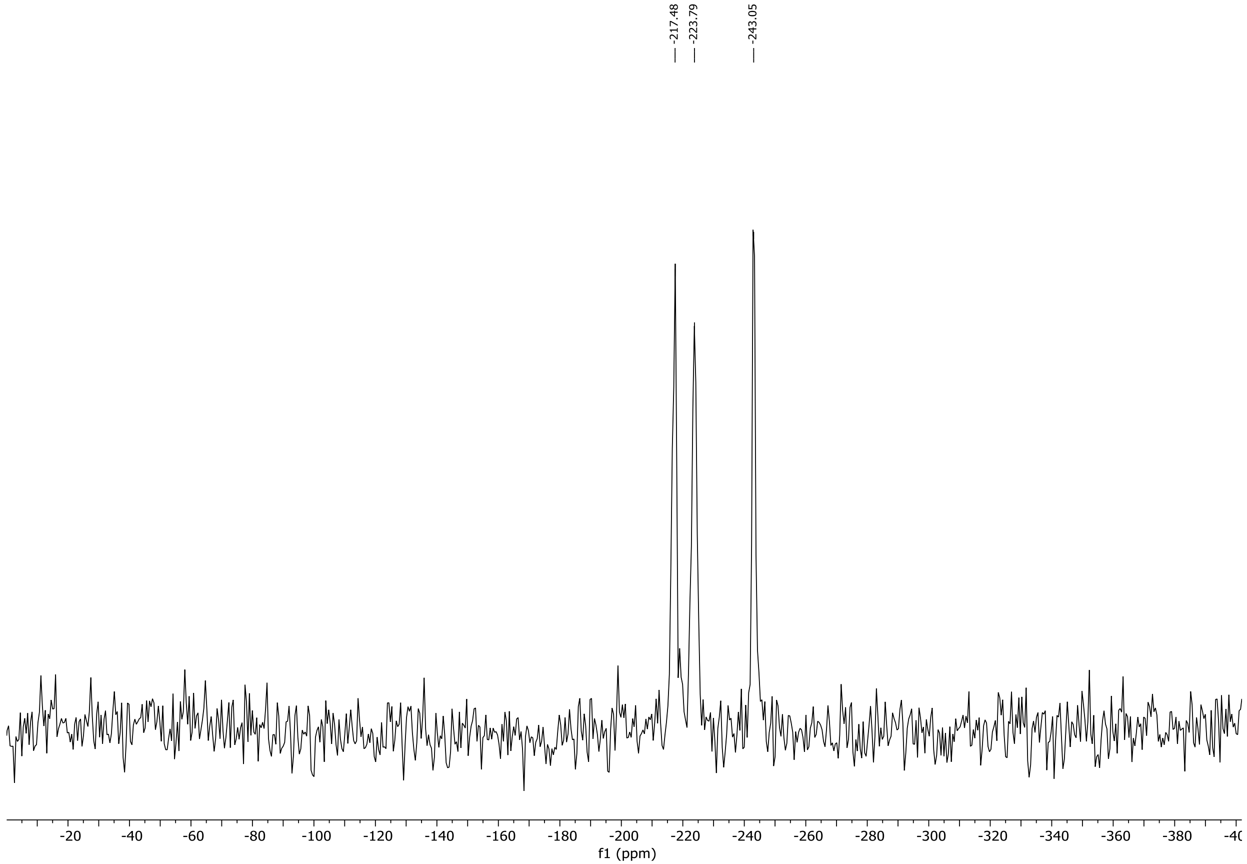

Solid-state nitrogen-15 NMR spectrum recorded with a 40 s recycle delay and 10 ms contact time.

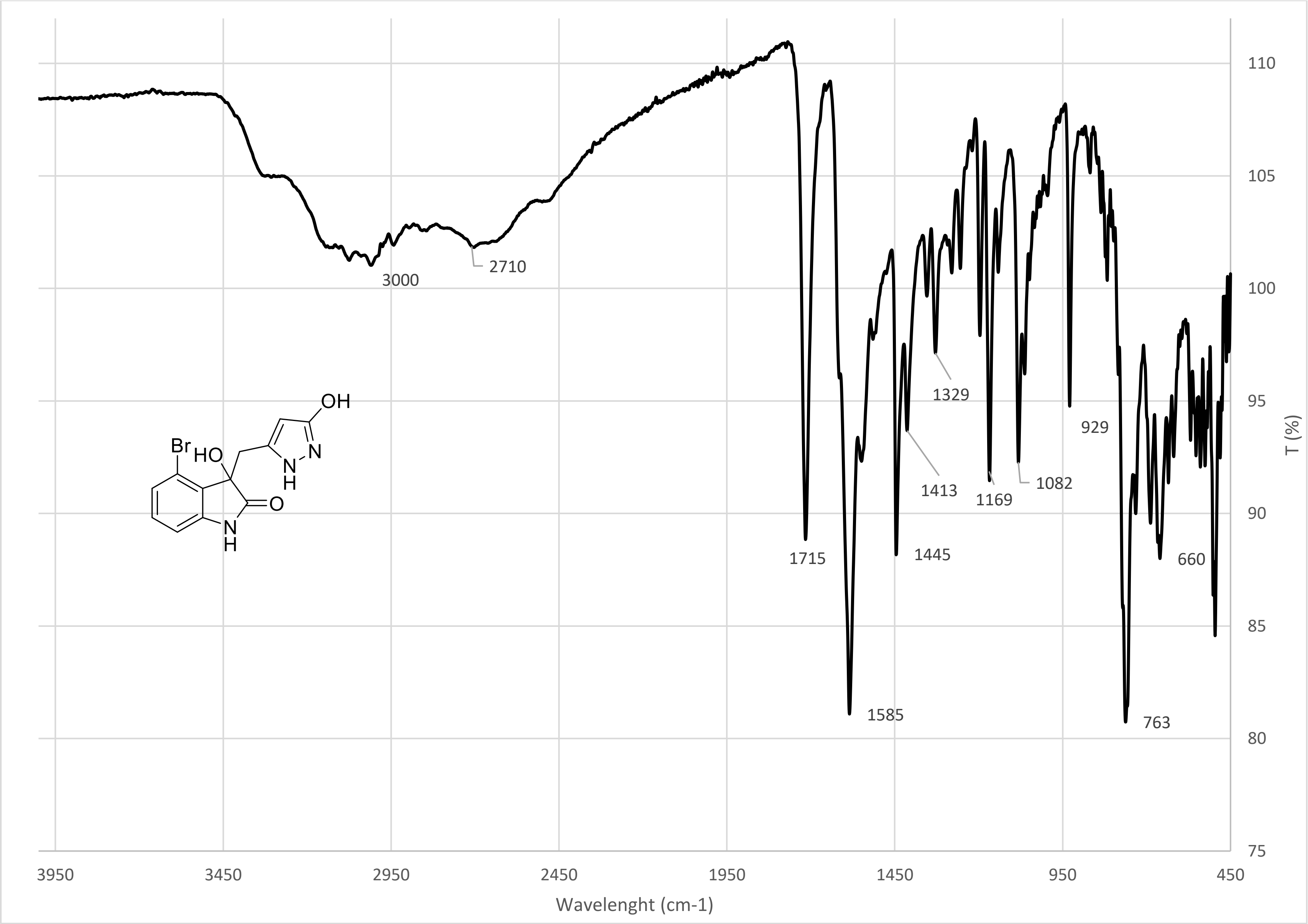

110
105
100
95
90
85
80
75
T (%)
3950
3450
2950
2450
1950
1450
950
450
Wavelenght (cm-1)
3000
2710
1715
1585
1445
1413
1329
1169
1082
929
763
660
Br
HO
OH
N
N
H
O
NH
H

Elemental Composition Report

Single Mass Analysis

Tolerance = 3.0 mDa / DBE: min = -1.5, max = 100.0
Element prediction: Off
Number of isotope peaks used for i-FIT = 5

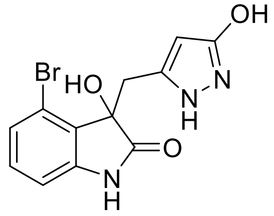

Monoisotopic Mass, Even Electron Ions
635 formula(e) evaluated with 4 results within limits (up to 500 closest results for each mass)
Elements Used:
C: 0-40 H: 0-80 N: 0-8 O: 0-8 Br: 0-2

QToF Premier

26-Nov-2019
GG310 230 (1.942) Cm (207:264)

1: TOF MS ES+
6.70e+005

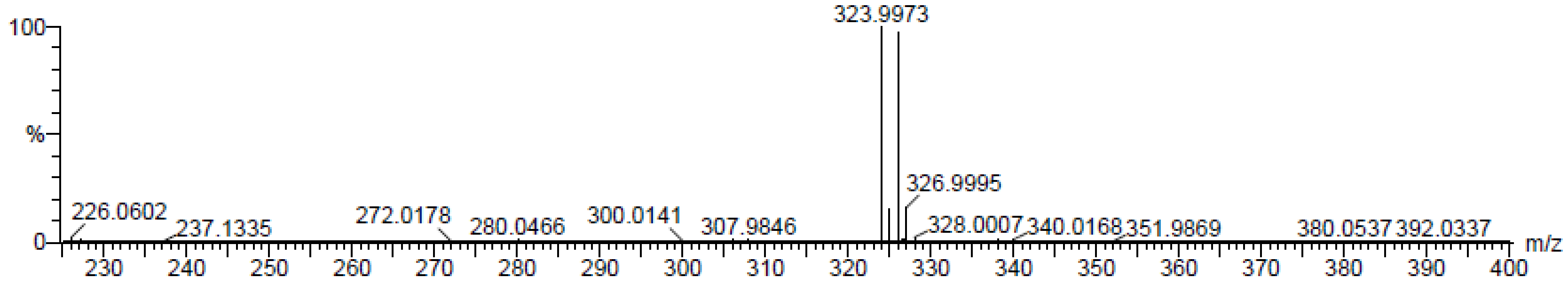

Minimum:				-1.5			
Maximum:		3.0	5.0	100.0			

Mass	Calc. Mass	mDa	PPM	DBE	i-FIT	i-FIT (Norm)	Formula
323.9973	323.9965	0.8	2.5	11.5	1635.3	29.3	C8 H2 N7 O8
	323.9962	1.1	3.4	1.5	1637.0	31.1	C11 H20 N Br2
	323.9984	-1.1	-3.4	8.5	1605.9	0.0	C12 H11 N3 O3 Br
	323.9944	2.9	9.0	4.5	1617.9	11.9	C7 H11 N5 O5 Br

Table 1 Crystal data and structure refinement for 1986565.	
Identification code	1986565
Empirical formula	$C_{12}H_{10}BrN_3O_3$
Formula weight	324.14
Temperature/K	120
Crystal system	orthorhombic
Space group	$P2_12_12_1$
a/Å	6.9156(3)
b/Å	13.1025(6)
c/Å	13.3565(6)
α/°	90
β/°	90
γ/°	90
Volume/Å^3	1210.25(9)
Z	4
ρ_{calc}g/cm^3	1.779
μ/mm^{-1}	3.404
F(000)	648.0
Crystal size/mm^3	0.243 × 0.102 × 0.071

Radiation	MoKα (λ = 0.71073)
2Θ range for data collection/°	4.354 to 66.514
Index ranges	-10 ≤ h ≤ 10, -20 ≤ k ≤ 20, -20 ≤ l ≤ 20
Reflections collected	32088
Independent reflections	4654 [R_{int} = 0.0355, R_{sigma} = 0.0268]
Data/restraints/parameters	4654/0/188
Goodness-of-fit on F^2	1.055
Final R indexes [I>=2σ (I)]	R_1 = 0.0249, wR_2 = 0.0561
Final R indexes [all data]	R_1 = 0.0308, wR_2 = 0.0576
Largest diff. peak/hole / e $Å^{-3}$	0.92/-0.65
Flack parameter	0.000(2)

Table 2 Fractional Atomic Coordinates (×10⁴) and Equivalent Isotropic Displacement Parameters (Å²×10³) for 1986565. U_{eq} is defined as 1/3 of of the trace of the orthogonalised U_{IJ} tensor.

Atom	*x*	*y*	*z*	U(eq)
Br	9479.2(3)	7691.3(2)	3241.3(2)	18.84(6)
O1	7612(2)	8746.1(12)	1112.2(11)	11.3(3)
O2	3672(2)	9025.3(13)	335.7(11)	15.8(3)
O3	639(2)	5187.4(11)	2892.2(10)	13.3(3)

N1	3085(3)	9224.4(14)	2023.4(13)	12.6(3)
N2	2692(3)	6498.0(15)	919.3(13)	13.4(3)
N3	1296(3)	5899.5(14)	1342.7(13)	13.1(3)
C1	5957(3)	8354.9(14)	1597.6(13)	9.2(3)
C2	5838(3)	8542.5(14)	2710.0(13)	9.3(3)
C3	7075(3)	8322.0(16)	3491.6(15)	12.6(4)
C4	6586(3)	8592.1(18)	4468.9(15)	17.6(4)
C5	4846(3)	9077.4(18)	4649.8(15)	18.8(4)
C6	3560(3)	9307.3(17)	3880.2(15)	15.8(4)
C7	4088(3)	9032.7(15)	2914.9(14)	10.4(3)
C8	4103(3)	8907.3(15)	1218.1(14)	10.9(3)
C9	5876(3)	7209.3(16)	1314.5(15)	12.2(3)
C10	4072(3)	6663.0(14)	1610.3(14)	10.0(3)
C11	3495(3)	6230.0(15)	2503.6(14)	10.9(3)
C12	1703(3)	5719.5(15)	2321.9(14)	10.4(3)

Table 3 Anisotropic Displacement Parameters (Å^2×10^3) for 1986565. The Anisotropic displacement factor exponent takes the form: -2π^2[h^{2}a*^{2}U$_{11}$+2hka*b*U$_{12}$+…].

Atom	U_{11}	U_{22}	U_{33}	U_{23}	U_{13}	U_{12}
Br	15.00(9)	24.16(10)	17.35(9)	4.74(8)	-1.91(8)	6.98(8)
O1	10.7(6)	14.7(7)	8.5(6)	-1.8(5)	3.0(5)	-4.3(5)
O2	15.6(7)	22.3(8)	9.4(6)	2.0(6)	-2.0(5)	1.2(6)
O3	12.5(6)	13.3(6)	13.9(6)	4.0(5)	2.5(6)	-1.4(6)
N1	10.9(7)	15.8(8)	11.0(7)	0.3(6)	1.0(6)	4.1(6)
N2	15.8(8)	16.9(8)	7.5(7)	1.0(6)	-0.8(6)	-4.5(7)
N3	11.8(8)	15.9(8)	11.7(7)	0.9(6)	-2.7(6)	-5.1(6)
C1	8.8(8)	11.4(8)	7.4(8)	-1.0(6)	1.7(6)	-0.5(6)
C2	11.6(8)	9.4(8)	7.0(7)	0.3(6)	2.6(6)	-0.5(6)
C3	13.4(8)	13.2(9)	11.1(8)	1.3(6)	0.9(7)	0.0(7)
C4	23.6(11)	21.7(11)	7.6(8)	1.0(7)	-1.8(8)	-1.2(9)
C5	26.7(11)	20.6(10)	9.1(8)	-2.0(7)	4.0(8)	-0.4(8)
C6	18.4(10)	17.5(10)	11.6(8)	-2.1(7)	5.7(8)	2.4(8)
C7	11.3(9)	11.3(8)	8.6(7)	0.5(6)	1.7(6)	0.2(6)
C8	11.4(9)	10.9(8)	10.3(8)	-0.3(6)	0.8(6)	-1.5(6)
C9	12.3(8)	11.4(9)	12.9(8)	-3.3(7)	4.3(6)	-1.3(7)
C10	11.9(8)	9.6(8)	8.5(8)	-1.7(6)	0.6(6)	0.5(6)
C11	10.3(8)	13.8(9)	8.5(8)	0.3(7)	-1.4(7)	-1.0(7)

C12	11.7(8)	10.1(8)	9.4(8)	-0.2(6)	0.0(7)	1.6(7)

Table 4 Bond Lengths for 1986565.						
**Atom**	**Atom**	**Length/Å**		**Atom**	**Atom**	**Length/Å**
Br	C3	1.887(2)		C1	C9	1.549(3)
O1	C1	1.412(2)		C2	C3	1.380(3)
O2	C8	1.226(2)		C2	C7	1.397(3)
O3	C12	1.268(2)		C3	C4	1.394(3)
N1	C7	1.401(3)		C4	C5	1.382(3)
N1	C8	1.351(3)		C5	C6	1.392(3)
N2	N3	1.367(3)		C6	C7	1.388(3)
N2	C10	1.345(3)		C9	C10	1.492(3)
N3	C12	1.358(3)		C10	C11	1.380(3)
C1	C2	1.508(2)		C11	C12	1.429(3)
C1	C8	1.557(3)				

Table 5 Bond Angles for 1986565.								
**Atom**	**Atom**	**Atom**	**Angle/˚**		**Atom**	**Atom**	**Atom**	**Angle/˚**

C8	N1	C7	111.33(17)		C4	C5	C6	121.77(19)
C10	N2	N3	108.03(16)		C7	C6	C5	117.51(19)
C12	N3	N2	110.58(18)		C2	C7	N1	110.17(16)
O1	C1	C2	115.96(15)		C6	C7	N1	127.82(18)
O1	C1	C8	110.44(15)		C6	C7	C2	121.95(18)
O1	C1	C9	105.60(15)		O2	C8	N1	126.87(19)
C2	C1	C8	101.54(15)		O2	C8	C1	124.91(17)
C2	C1	C9	113.35(15)		N1	C8	C1	108.22(16)
C9	C1	C8	109.95(15)		C10	C9	C1	115.50(16)
C3	C2	C1	132.62(18)		N2	C10	C9	119.26(17)
C3	C2	C7	119.00(17)		N2	C10	C11	108.80(17)
C7	C2	C1	108.38(16)		C11	C10	C9	131.91(18)
C2	C3	Br	120.25(15)		C10	C11	C12	107.23(17)
C2	C3	C4	120.32(19)		O3	C12	N3	123.59(19)
C4	C3	Br	119.39(16)		O3	C12	C11	131.20(18)
C5	C4	C3	119.4(2)		N3	C12	C11	105.20(18)

**Table 6 Hydrogen Bonds for 1986565.**						
**D**	**H**	**A**	**d(D-H)/Å**	**d(H-A)/Å**	**d(D-A)/Å**	**D-H-A/°**

O1	H0	O3[1]	0.82(4)	1.79(4)	2.608(2)	172(4)
N1	H1	O3[2]	0.85(3)	2.03(3)	2.870(2)	168(3)
N2	H2	O1[3]	0.92(3)	1.83(3)	2.733(2)	170(3)
N3	H3	O2[3]	0.77(3)	2.19(3)	2.886(2)	150(3)

[1]1-X,1/2+Y,1/2-Z; [2]-X,1/2+Y,1/2-Z; [3]-1/2+X,3/2-Y,-Z

Table 7 Torsion Angles for 1986565.

A	B	C	D	Angle/°		A	B	C	D	Angle/°
Br	C3	C4	C5	178.38(18)		C3	C2	C7	C6	0.2(3)
O1	C1	C2	C3	-55.4(3)		C3	C4	C5	C6	-0.1(4)
O1	C1	C2	C7	124.82(18)		C4	C5	C6	C7	-0.1(3)
O1	C1	C8	O2	50.4(3)		C5	C6	C7	N1	-176.8(2)
O1	C1	C8	N1	-129.59(17)		C5	C6	C7	C2	0.1(3)
O1	C1	C9	C10	-170.80(16)		C7	N1	C8	O2	-175.1(2)
N2	N3	C12	O3	179.78(18)		C7	N1	C8	C1	4.9(2)
N2	N3	C12	C11	-1.1(2)		C7	C2	C3	Br	-178.41(15)
N2	C10	C11	C12	3.6(2)		C7	C2	C3	C4	-0.4(3)
N3	N2	C10	C9	173.83(17)		C8	N1	C7	C2	-1.5(2)

N3	N2	C10	C11	-4.2(2)		C8	N1	C7	C6	175.6(2)
C1	C2	C3	Br	1.9(3)		C8	C1	C2	C3	-175.2(2)
C1	C2	C3	C4	179.8(2)		C8	C1	C2	C7	5.1(2)
C1	C2	C7	N1	-2.7(2)		C8	C1	C9	C10	-51.7(2)
C1	C2	C7	C6	179.99(19)		C9	C1	C2	C3	67.0(3)
C1	C9	C10	N2	100.1(2)		C9	C1	C2	C7	-112.77(18)
C1	C9	C10	C11	-82.3(3)		C9	C1	C8	O2	-65.7(2)
C2	C1	C8	O2	174.0(2)		C9	C1	C8	N1	114.26(17)
C2	C1	C8	N1	-6.0(2)		C9	C10	C11	C12	-174.17(19)
C2	C1	C9	C10	61.2(2)		C10	N2	N3	C12	3.3(2)
C2	C3	C4	C5	0.4(3)		C10	C11	C12	O3	177.5(2)
C3	C2	C7	N1	177.56(18)		C10	C11	C12	N3	-1.5(2)

Table 8 Hydrogen Atom Coordinates (Å×10^4) and Isotropic Displacement Parameters (Å^2×10^3) for 1986565.

Atom	*x*	*y*	*z*	U(eq)
H0	8230(50)	9150(30)	1450(20)	36(10)
H1	2060(50)	9590(30)	2010(20)	25(8)
H2	2810(40)	6450(20)	240(20)	22(7)
H3	410(50)	5750(20)	1020(20)	24(8)

H4	7441.52	8443.88	5006.07	21
H5	4518.79	9258.92	5317.21	23
H6	2365.95	9639.71	4011.12	19
H9A	6033.36	7148.65	580.2	15
H9B	6989.2	6860.09	1630.62	15
H11	4164.33	6264.92	3123.81	13

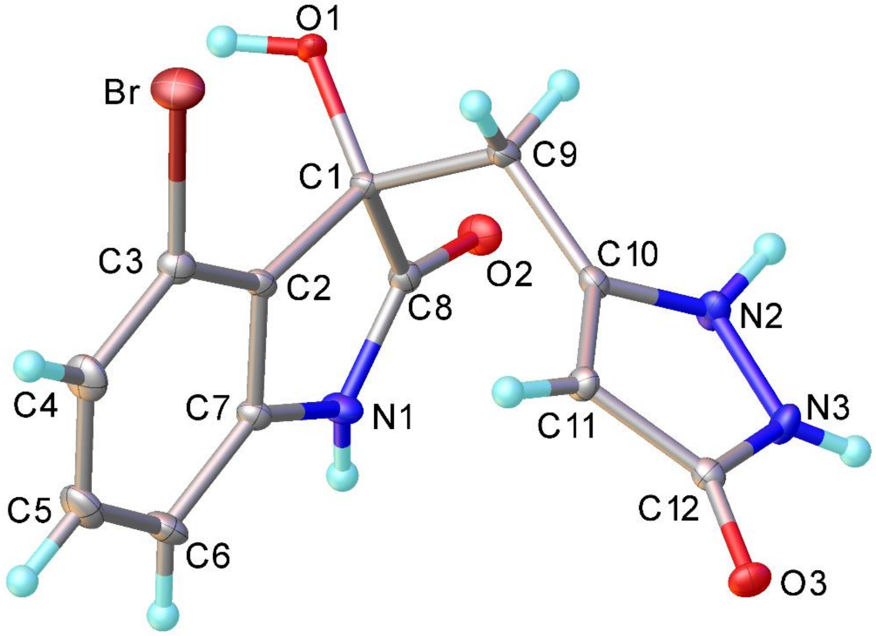

1.6 *Data of 5-chloro-3-hydroxy-3-((3-hydroxy-1H-pyrazol-5-yl)methyl)indolin-2-one (**15d**)*

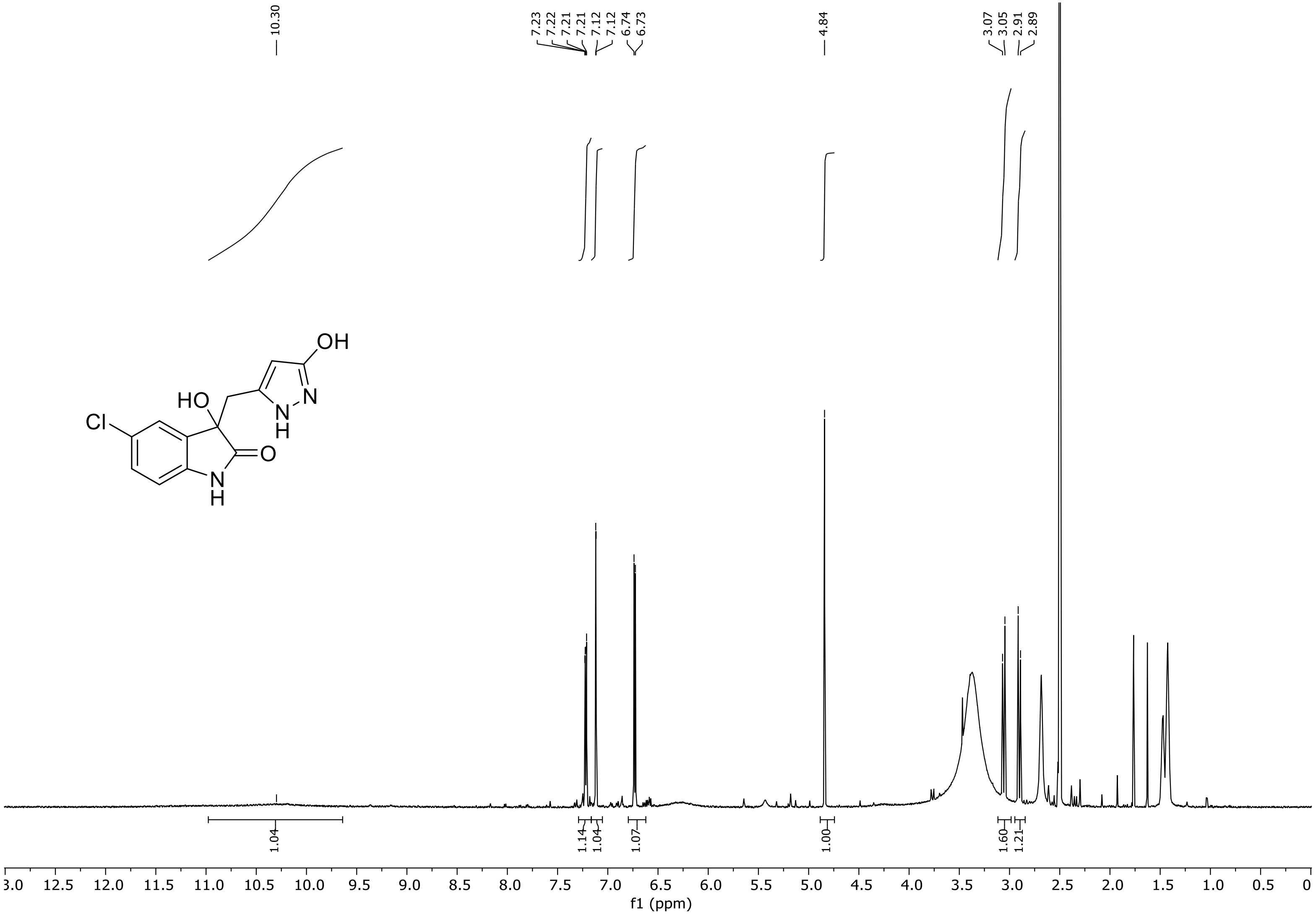

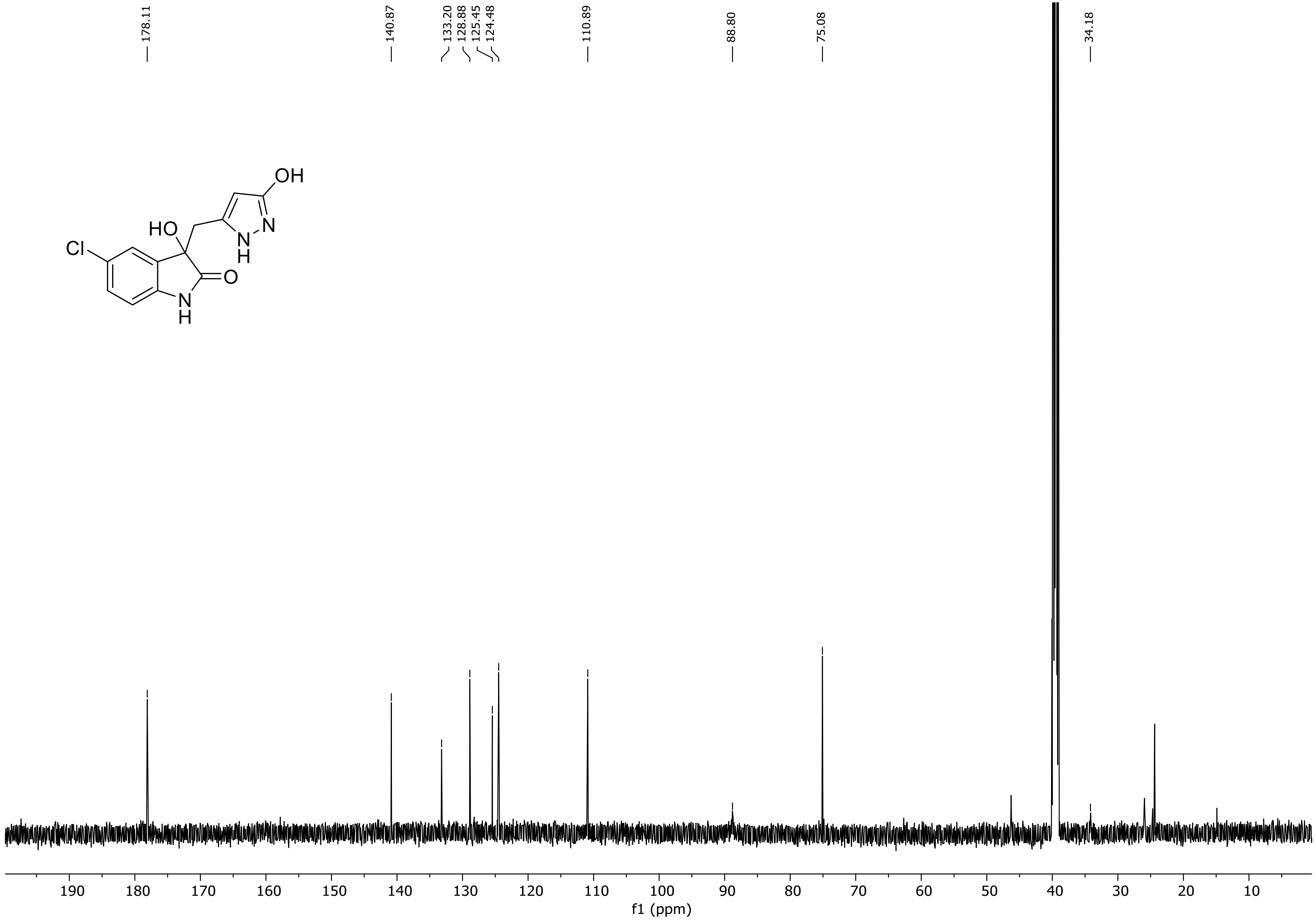
178.11
140.87
133.20
128.88
125.45
124.48
110.89
88.80
75.08
34.18
OH
HO
Cl
N
H
O
190 180 170 160 150 140 130 120 110 100 90 80 70 60 50 40 30 20 10
f1 (ppm)

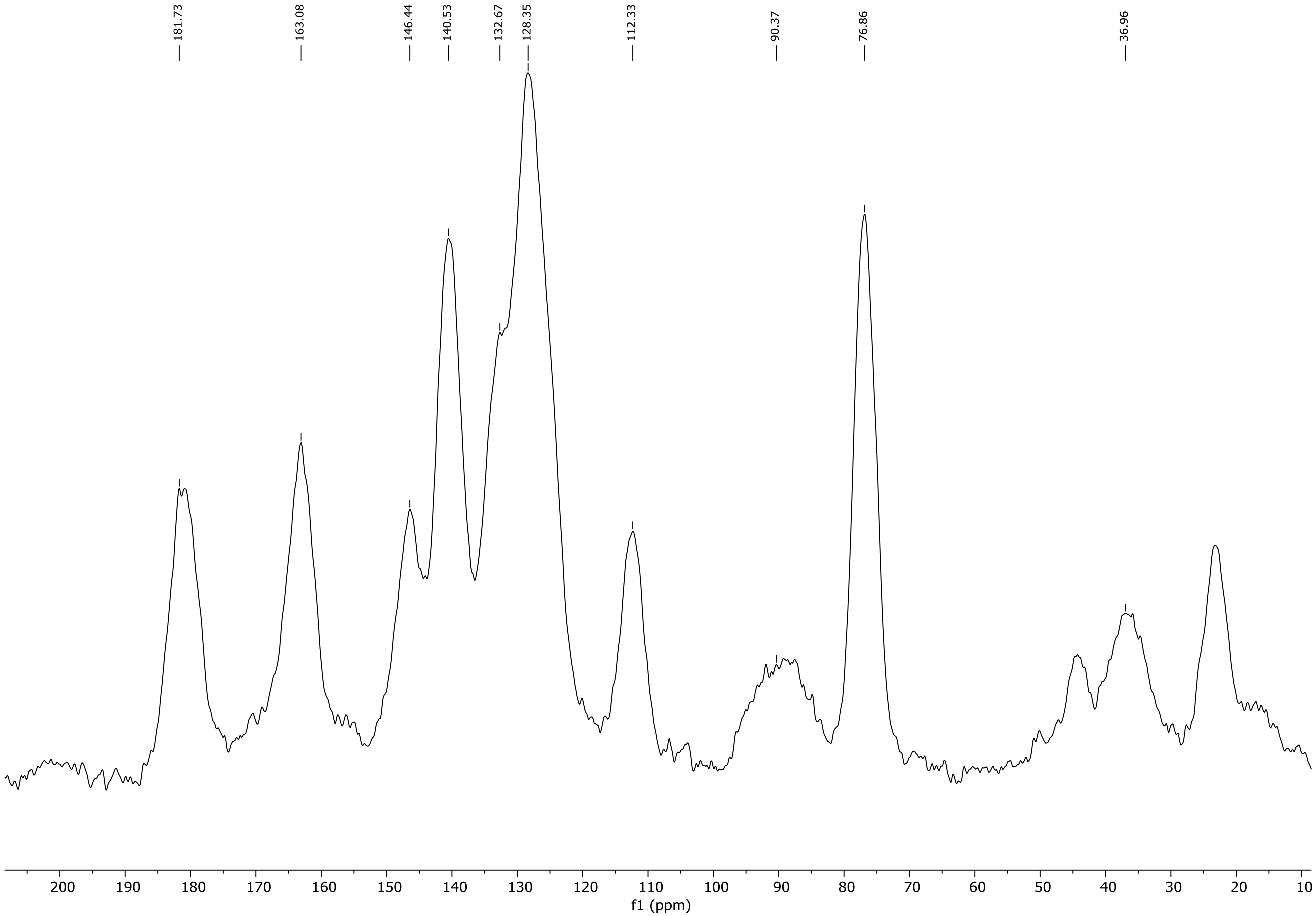

Solid-state carbon-13 NMR spectrum recorded with a 4 s recycle delay and 1 ms contact time.

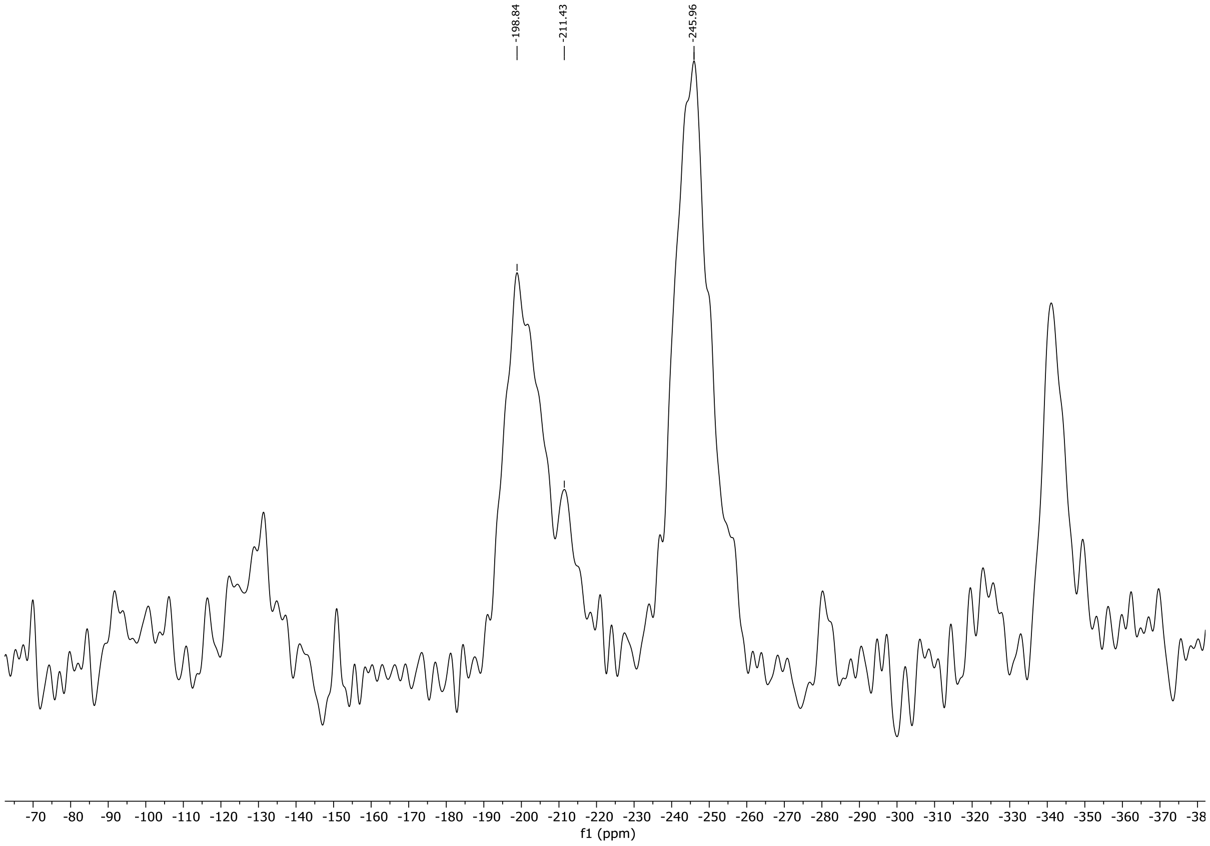

Solid-state nitrogen-15 NMR spectrum recorded with a 3 s recycle delay and 1 ms contact time.

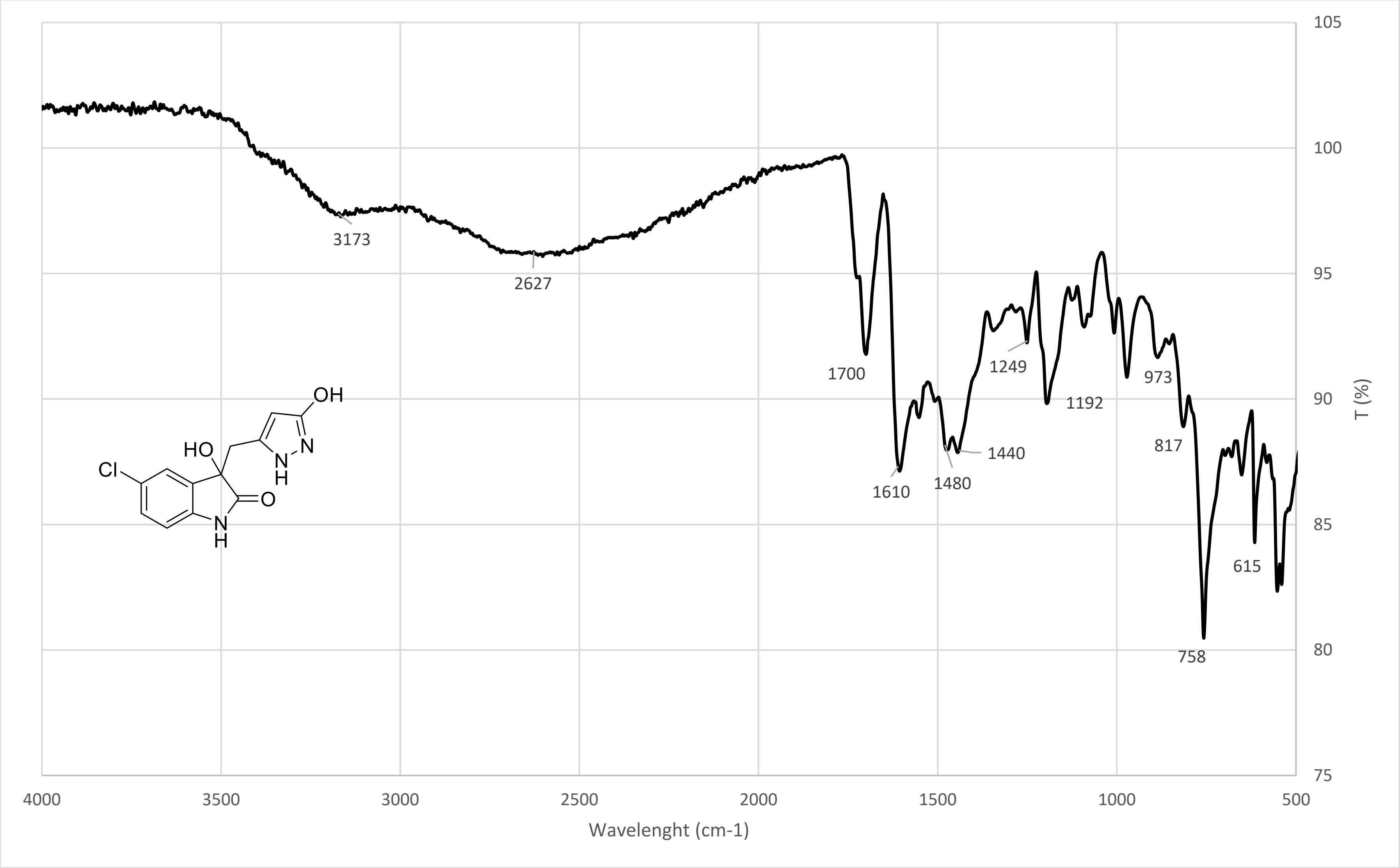

3173
2627
1700
1610
1480
1440
1249
1192
973
817
758
615
OH
HO
Cl
N
N
H
O
N
H
105
100
95
90
85
80
75
T (%)
4000
3500
3000
2500
2000
1500
1000
500
Wavelenght (cm-1)

Elemental Composition Report

Single Mass Analysis

Tolerance = 3.0 mDa / DBE: min = -1.5, max = 100.0
Element prediction: Off
Number of isotope peaks used for i-FIT = 5

Monoisotopic Mass, Even Electron Ions
369 formula(e) evaluated with 4 results within limits (up to 500 closest results for each mass)
Elements Used:
C: 0-60 H: 0-80 N: 0-4 O: 0-4 Cl: 0-2 I: 0-3

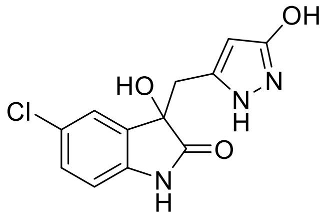

QToF Premier

05-Jun-2019
GG234A 235 (2.006) Cm (233:246)

1: TOF MS ES+
1.25e+005

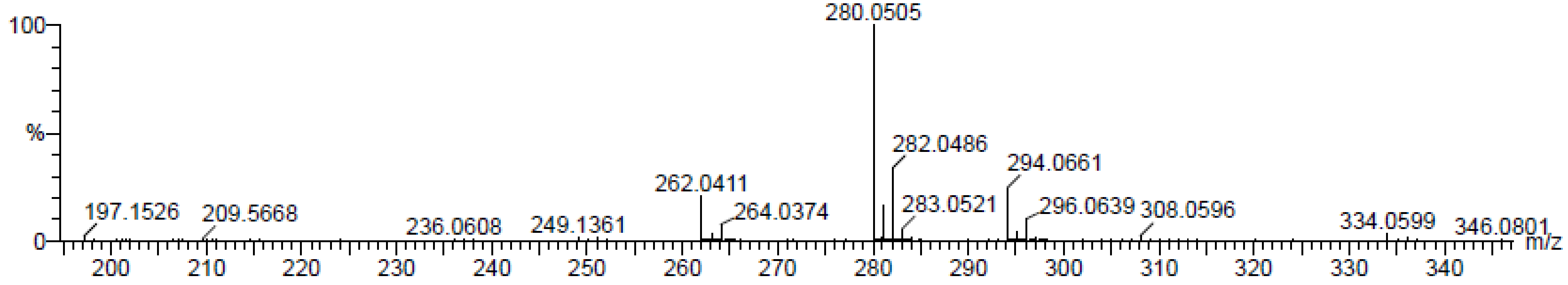

Minimum:				-1.5			
Maximum:		3.0	5.0	100.0			

Mass	Calc. Mass	mDa	PPM	DBE	i-FIT	i-FIT (Norm)	Formula
280.0505	280.0507	-0.2	-0.7	3.5	985.2	23.1	C11 H16 N O3 Cl2
	280.0511	-0.6	-2.1	17.5	988.0	26.0	C18 H6 N3 O
	280.0489	1.6	5.7	8.5	962.1	0.1	C12 H11 N3 O3 Cl
	280.0529	-2.4	-8.6	12.5	964.9	2.8	C17 H11 N O Cl

*1.7 Data of 3-hydroxy-3-((3-hydroxy-1H-pyrazol-5-yl)methyl)-5-methoxyindolin-2-one (**15e**)*

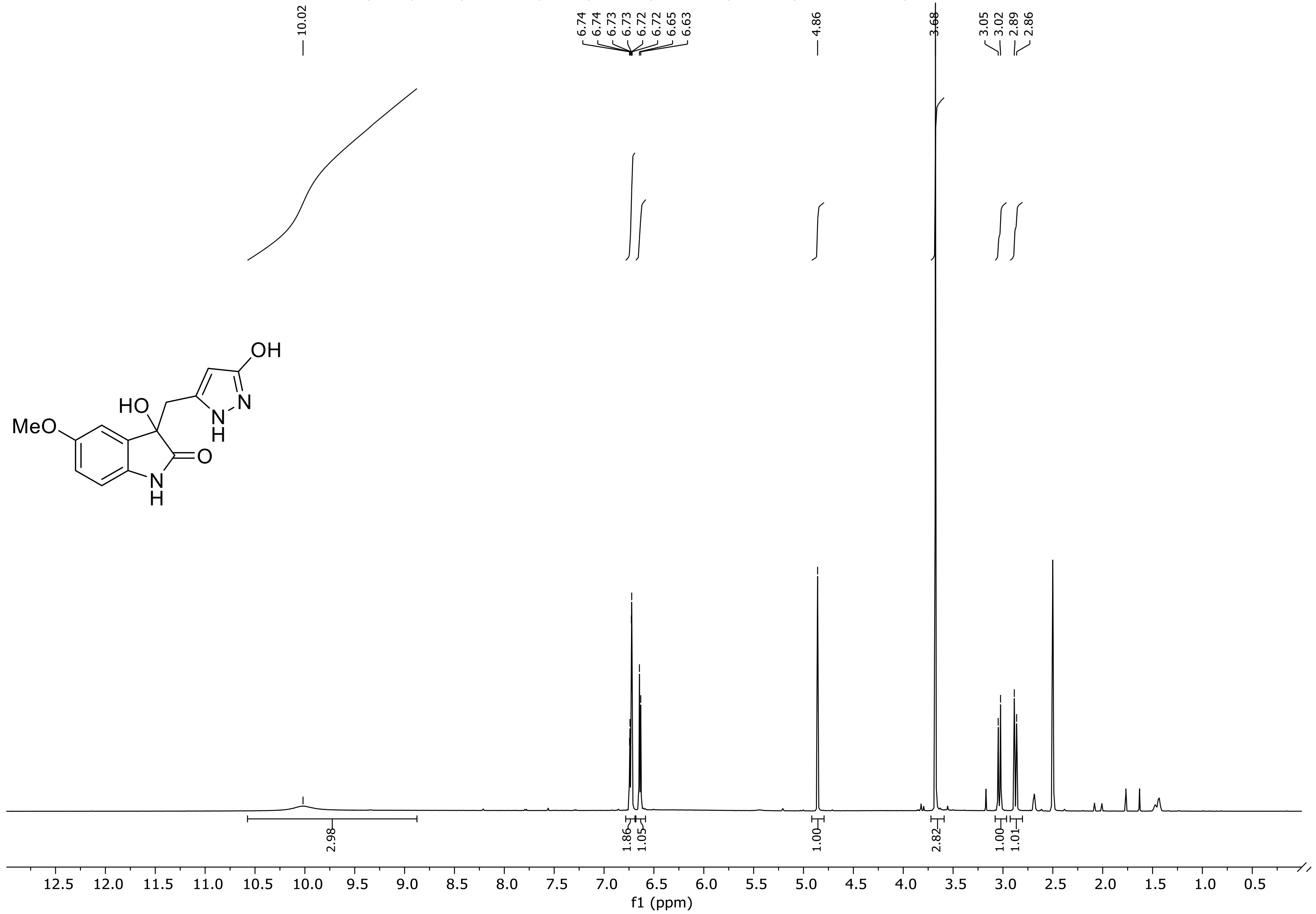

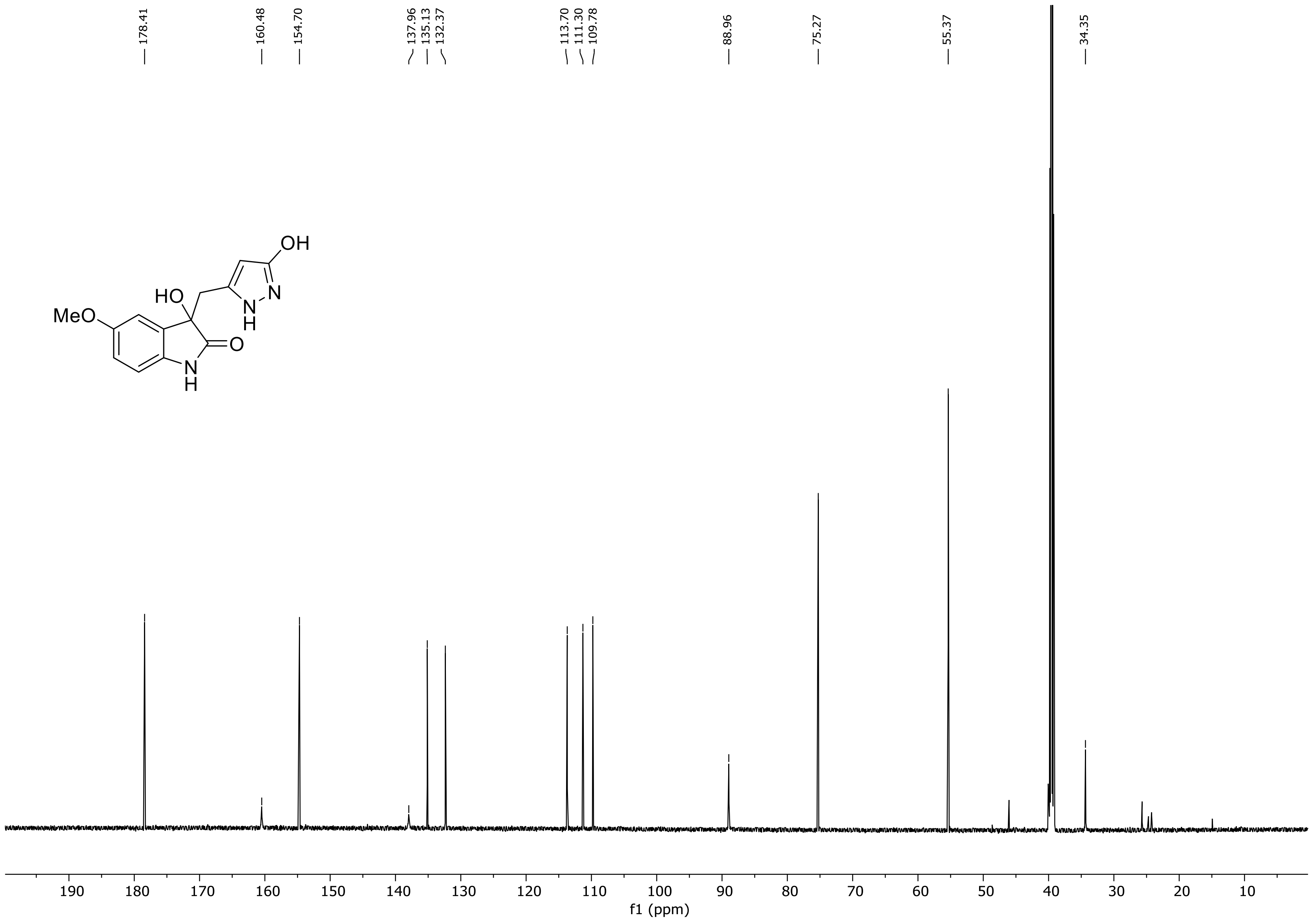

178.41
160.48
154.70
137.96
135.13
132.37
113.70
111.30
109.78
88.96
75.27
55.37
34.35
OH
HO
MeO
N
N
H
O
N
H
190
180
170
160
150
140
130
120
110
100
90
80
70
60
50
40
30
20
10
f1 (ppm)

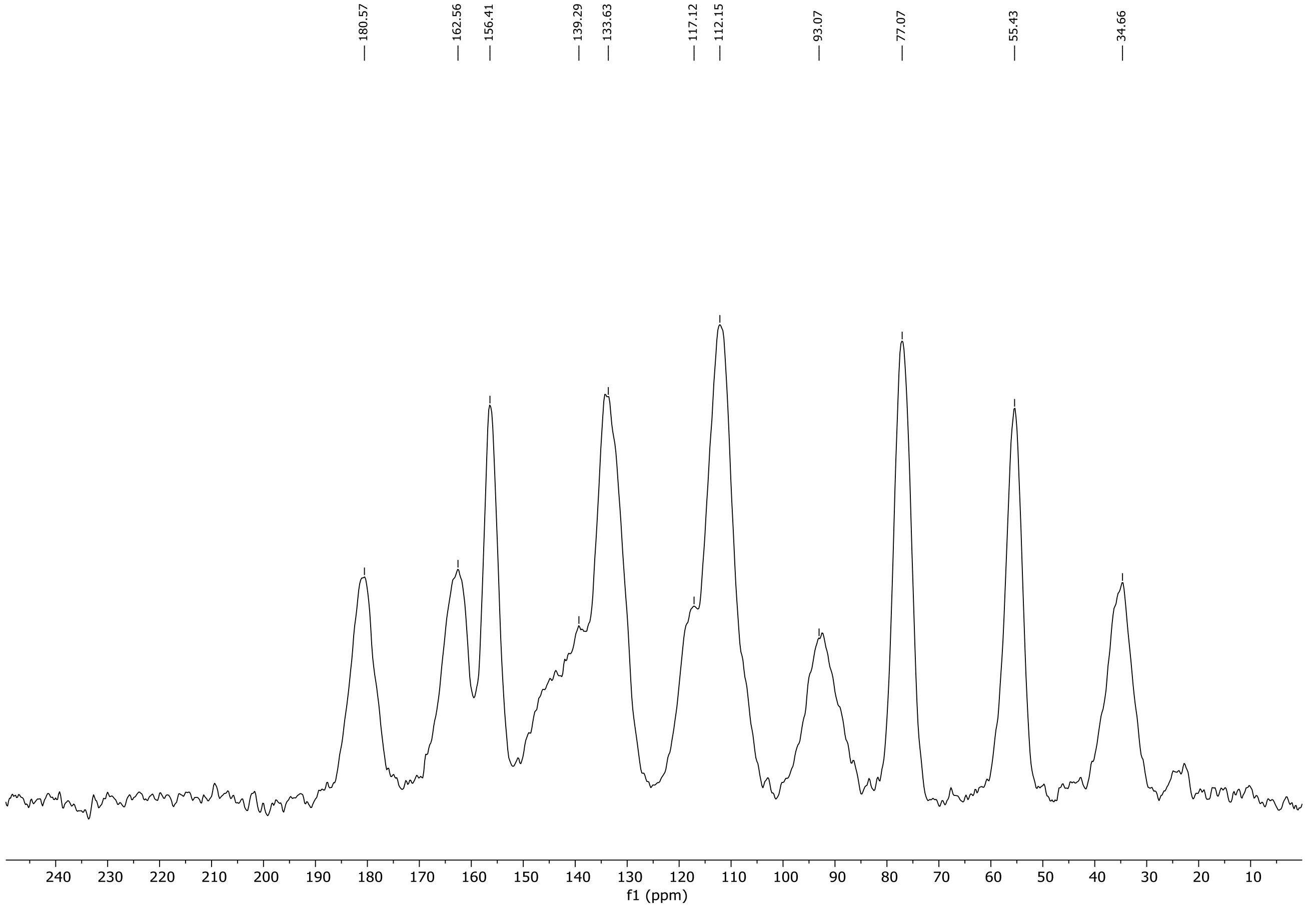

Solid-state carbon-13 NMR spectrum recorded with a 4 s recycle delay and 1 ms contact time.

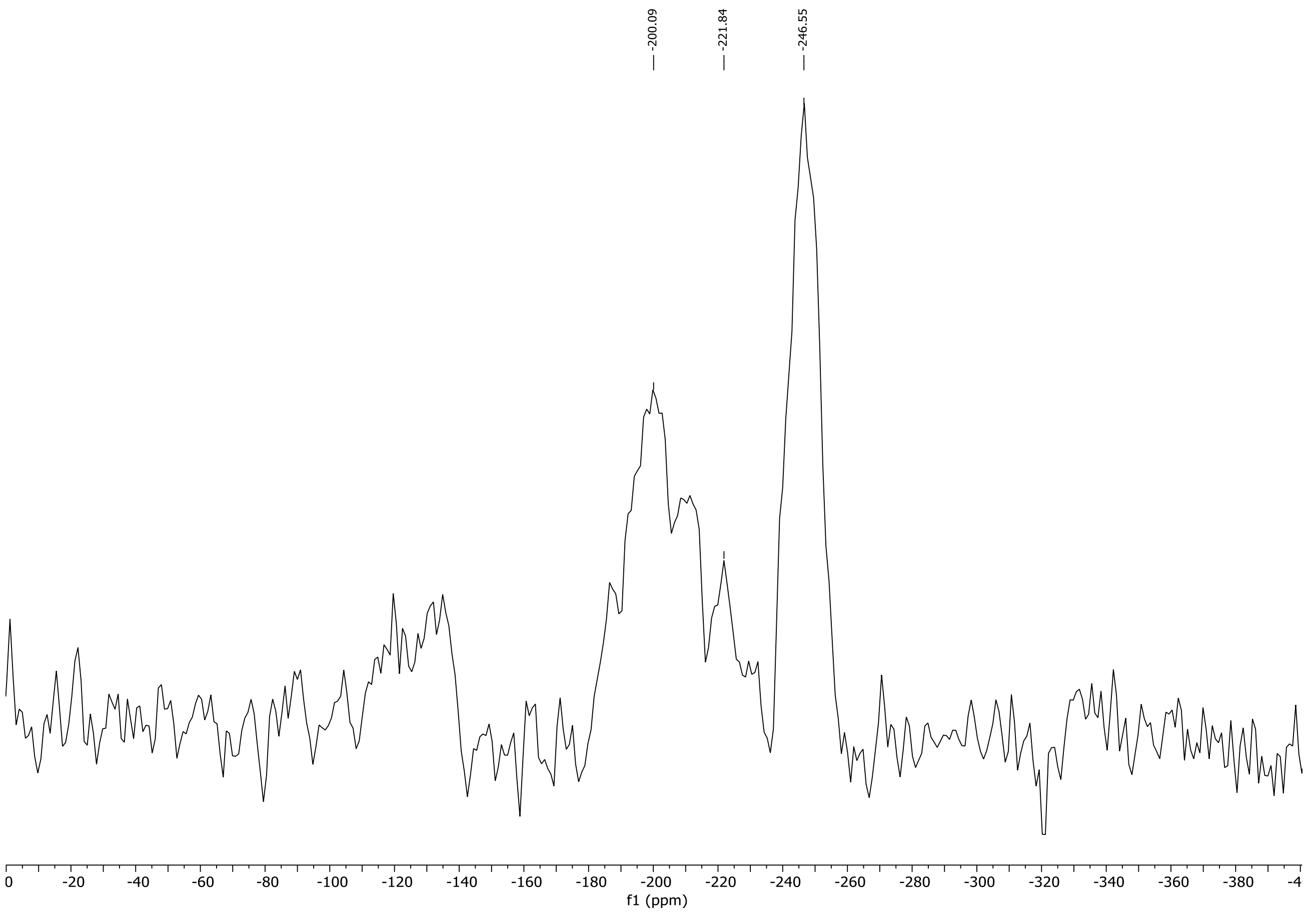

Solid-state nitrogen-15 NMR spectrum recorded with a 4 s recycle delay and 4 ms contact time.

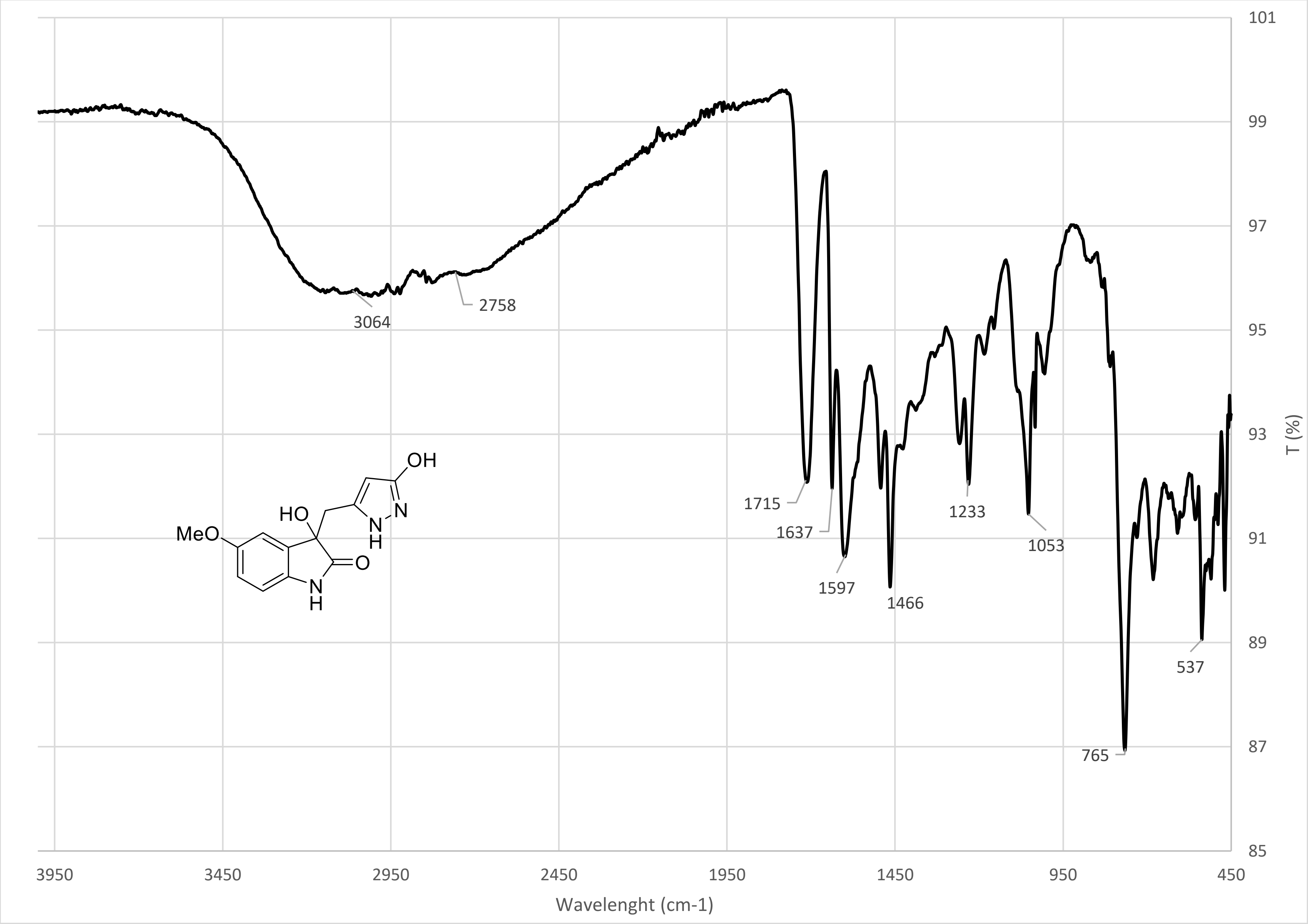

101
99
97
95
93
91
89
87
85
T (%)
3950
3450
2950
2450
1950
1450
950
450
Wavelenght (cm-1)
3064
2758
1715
1637
1597
1466
1233
1053
765
537
OH
HO
MeO
N
H
O

Elemental Composition Report

Single Mass Analysis

Tolerance = 3.0 mDa / DBE: min = -1.5, max = 100.0
Element prediction: Off
Number of isotope peaks used for i-FIT = 5

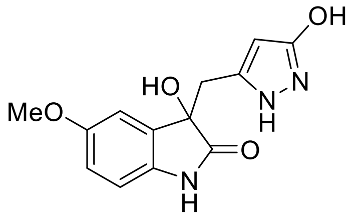

Monoisotopic Mass, Even Electron Ions
554 formula(e) evaluated with 4 results within limits (up to 500 closest results for each mass)
Elements Used:
C: 0-40 H: 0-80 N: 0-6 O: 0-6 S: 0-3

QToF Premier

24-Oct-2019
GG295A 195 (1.647) Cm (180:231)

1: TOF MS ES+
3.74e+005

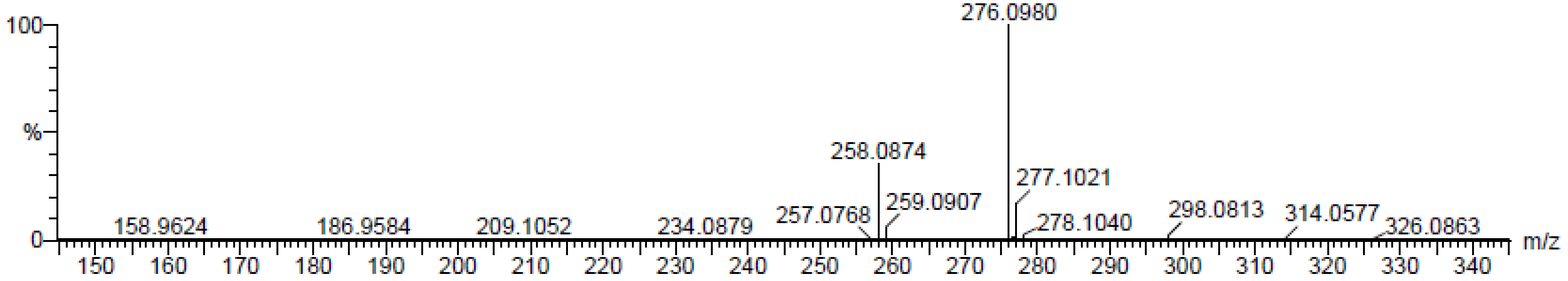

Minimum:				-1.5			
Maximum:		3.0	5.0	100.0			
Mass	Calc. Mass	mDa	PPM	DBE	i-FIT	i-FIT (Norm)	Formula
276.0980	276.0978	0.2	0.7	-0.5	1328.3	19.1	C5 H18 N5 O6 S
	276.0984	-0.4	-1.4	8.5	1309.2	0.0	C13 H14 N3 O4
	276.0987	-0.7	-2.5	-1.5	1334.5	25.3	C6 H22 N5 O S3
	276.0953	2.7	9.8	3.5	1332.0	22.8	C9 H18 N5 O S2

*1.8 Data of 5-fluoro-3-hydroxy-3-((3-hydroxy-1H-pyrazol-5-yl)methyl)indolin-2-one (**15f**)*

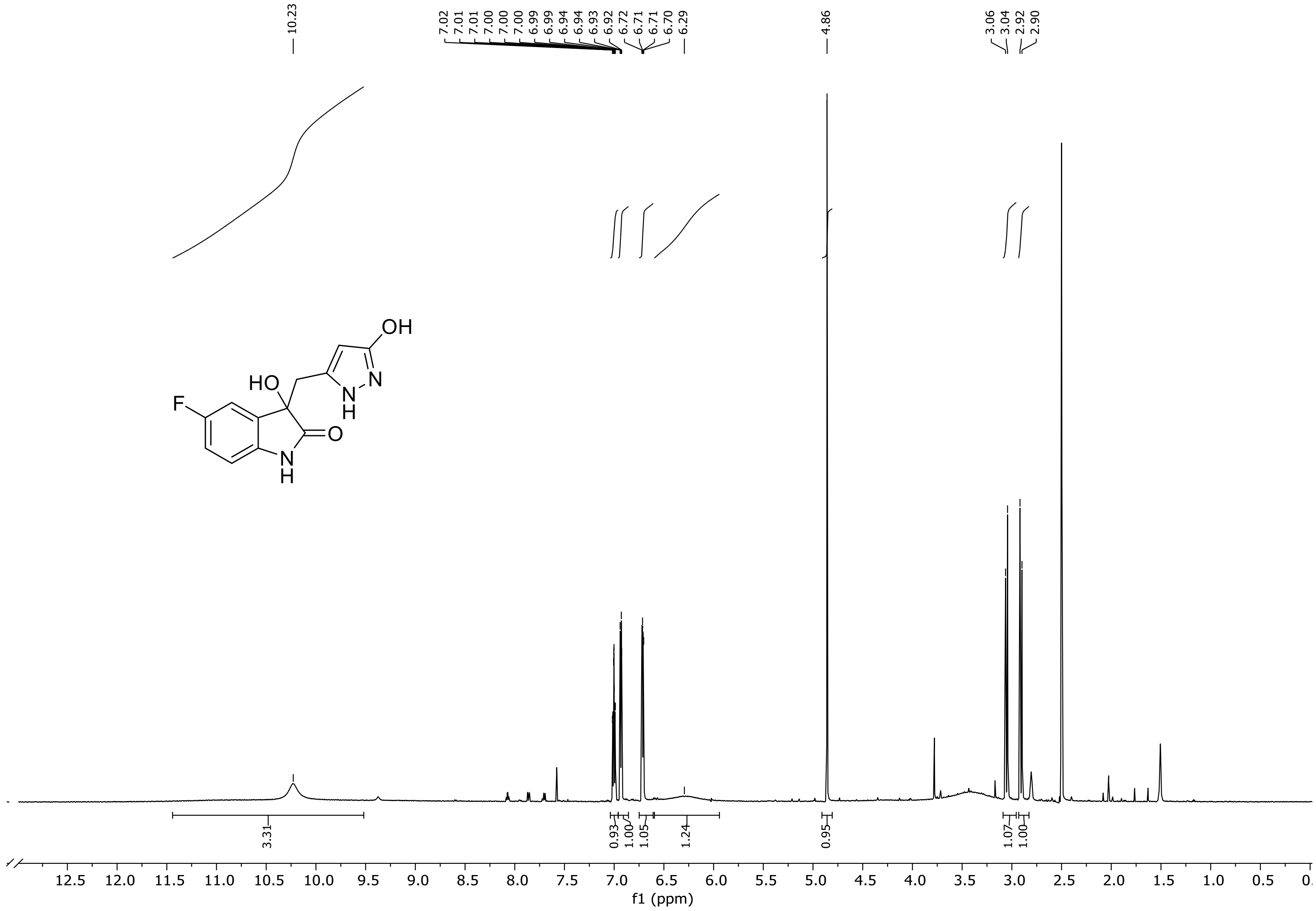

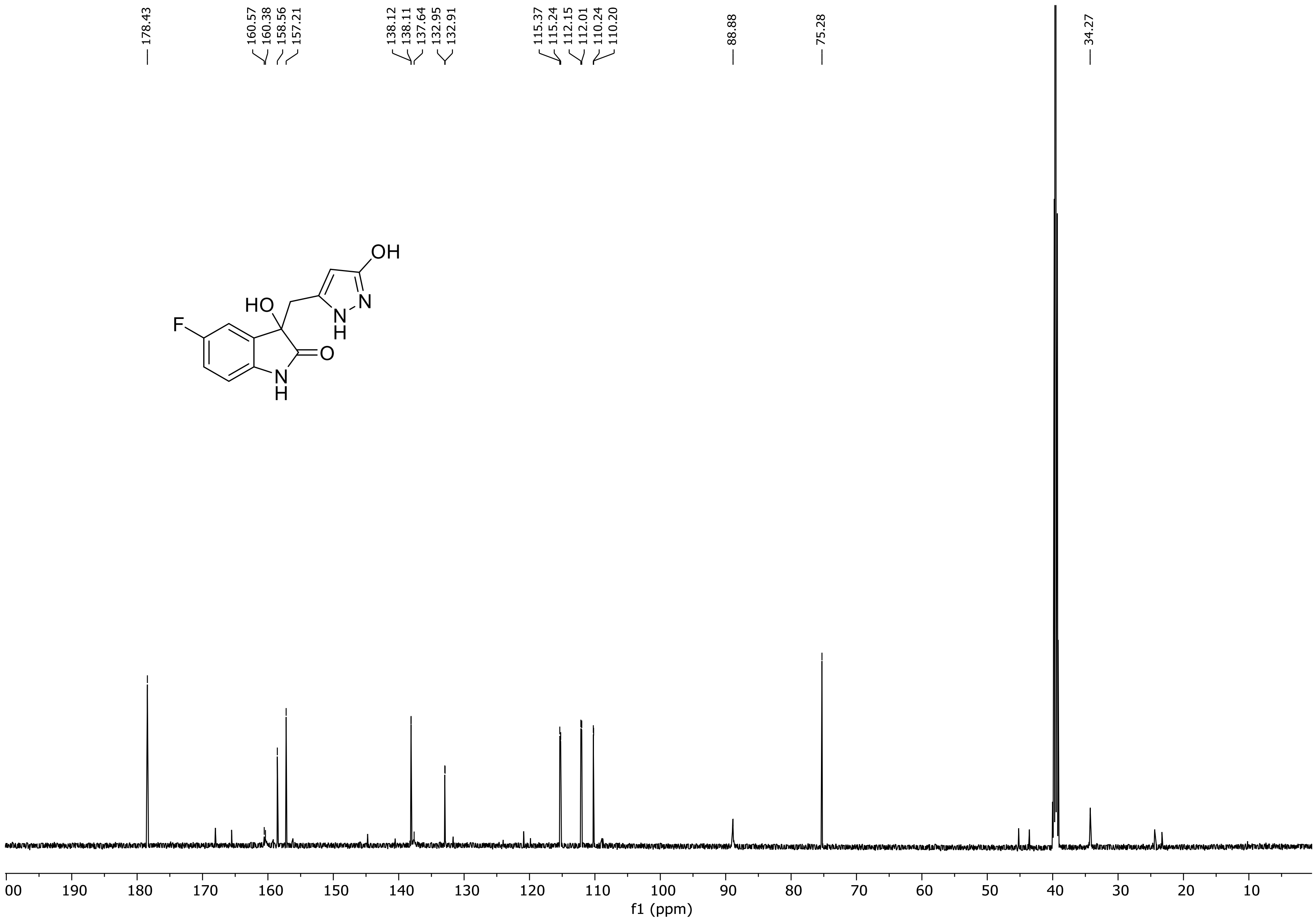
178.43
160.57
160.38
158.56
157.21
138.12
138.11
137.64
132.95
132.91
115.37
115.24
112.15
112.01
110.24
110.20
88.88
75.28
34.27
OH
HO
F
N
N
H
O
N
H
00
190
180
170
160
150
140
130
120
110
100
90
80
70
60
50
40
30
20
10
f1 (ppm)

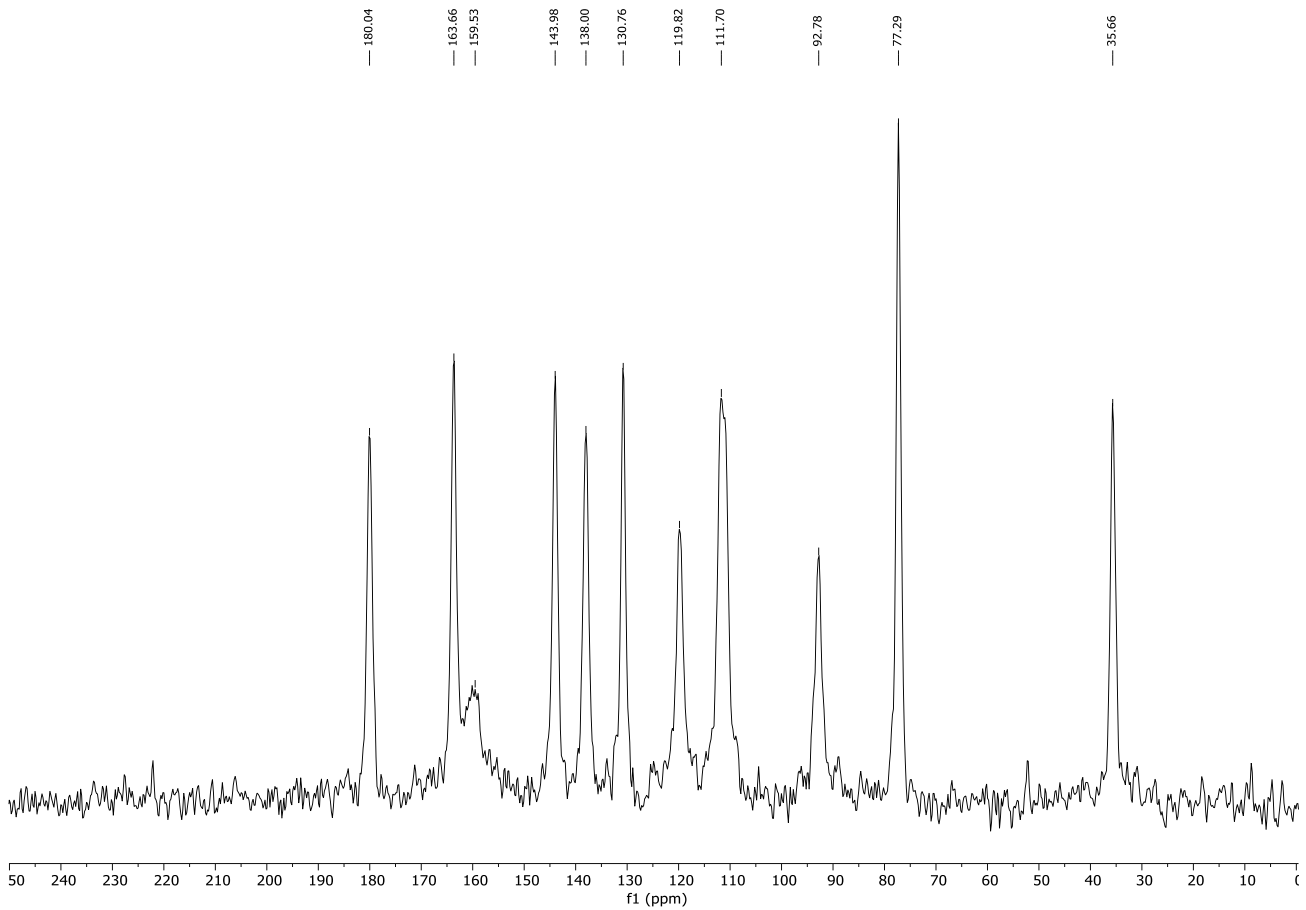

Solid-state carbon-13 NMR spectrum recorded with a 60 s recycle delay and 4 ms contact time .

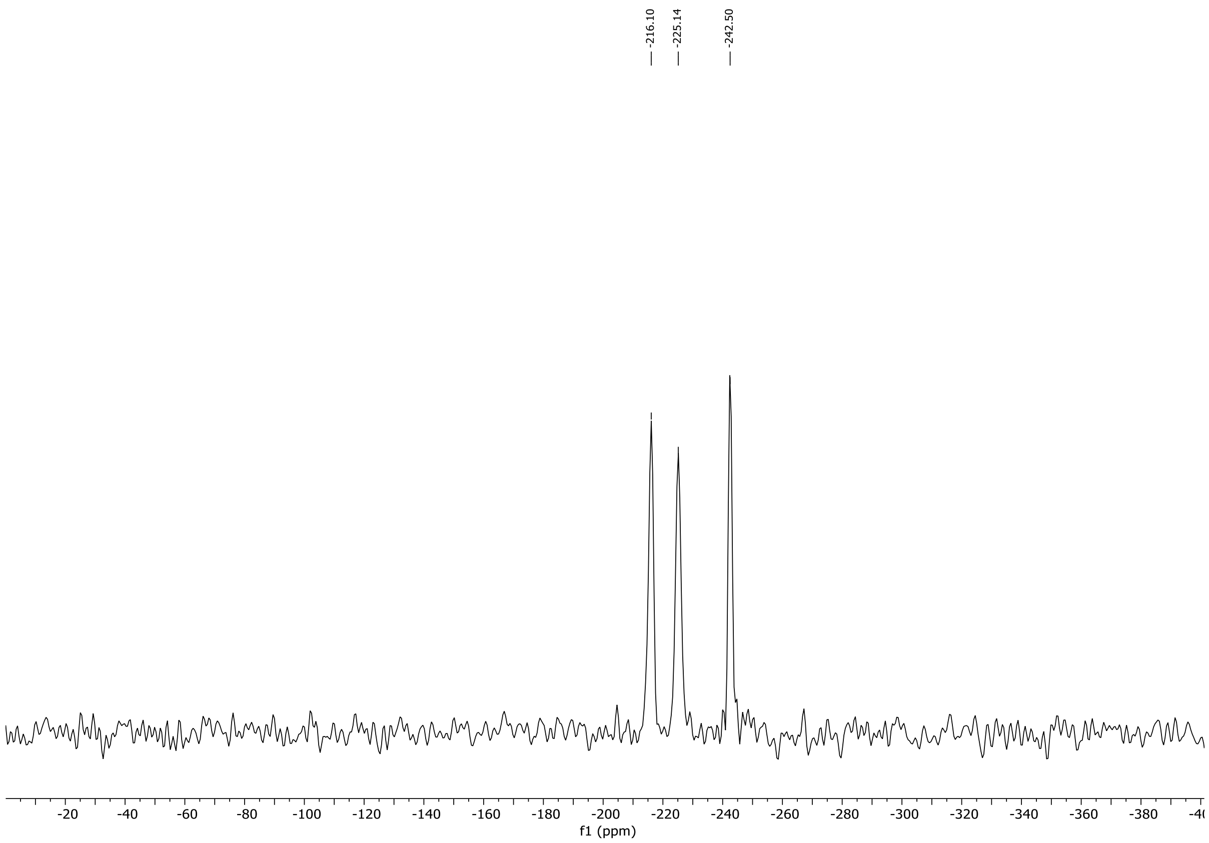

Solid-state nitrogen-15 NMR spectrum recorded with a 60 s recycle delay and 10 ms contact time.

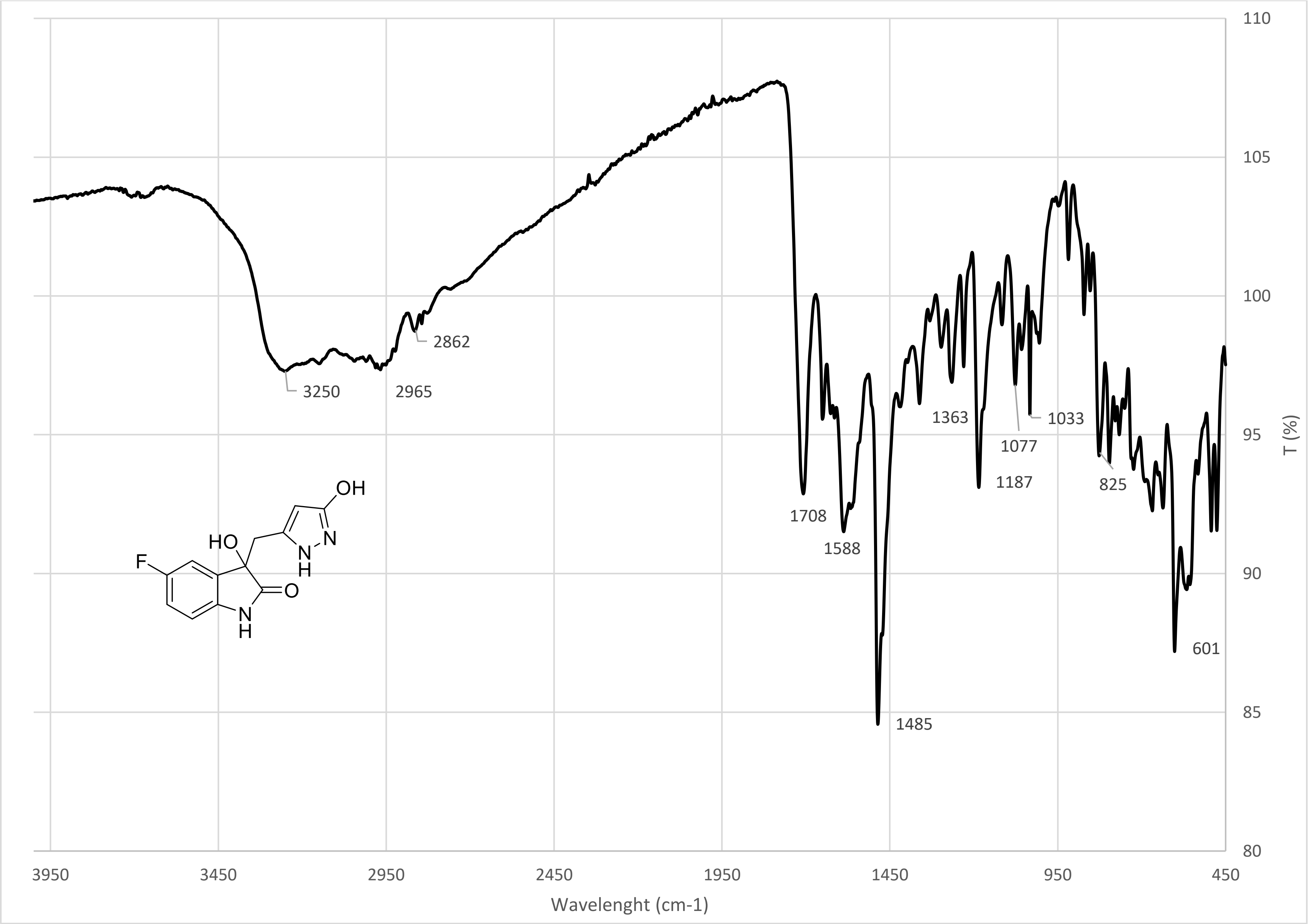

110
105
100
95
90
85
80
T (%)
3950
3450
2950
2450
1950
1450
950
450
Wavelenght (cm-1)
3250
2965
2862
1708
1588
1485
1363
1187
1077
1033
825
601
OH
HO
N
N
H
F
O
N
H

Elemental Composition Report

Single Mass Analysis

Tolerance = 3.0 mDa / DBE: min = -1.5, max = 100.0
Element prediction: Off
Number of isotope peaks used for i-FIT = 5

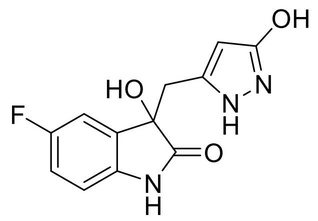

Monoisotopic Mass, Even Electron Ions
821 formula(e) evaluated with 5 results within limits (up to 500 closest results for each mass)
Elements Used:
C: 0-40 H: 0-80 N: 0-8 O: 0-8 F: 0-3

QToF Premier

27-Nov-2019
GG312A 202 (1.709) Cm (202:207)

1: TOF MS ES+
2.77e+004

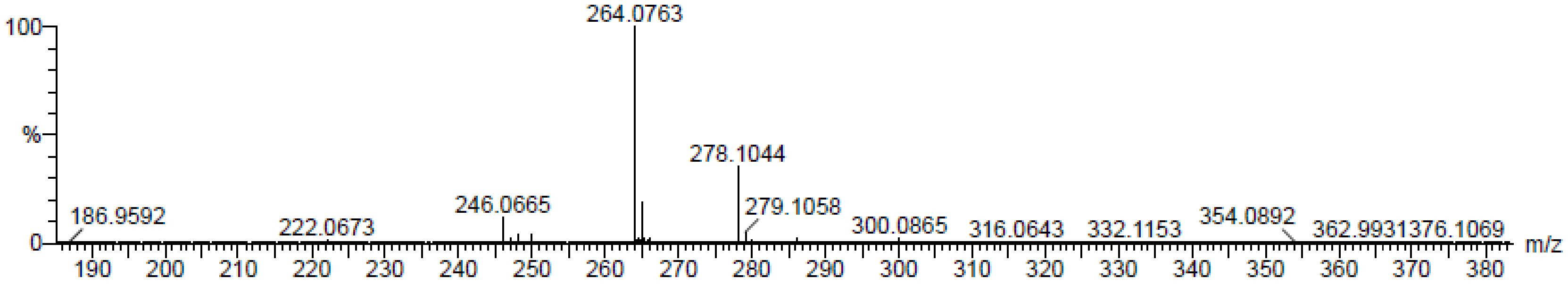

Minimum:				-1.5			
Maximum:		3.0	5.0	100.0			

Mass	Calc. Mass	mDa	PPM	DBE	i-FIT	i-FIT (Norm)	Formula
264.0763	264.0756	0.7	2.7	0.5	640.7	15.3	C4 H12 N5 O6 F2
	264.0773	-1.0	-3.8	12.5	625.4	0.0	C15 H10 N3 O2
	264.0749	1.4	5.3	9.5	633.0	7.6	C13 H9 N3 F3
	264.0744	1.9	7.2	4.5	638.3	12.9	C7 H11 N5 O5 F
	264.0784	-2.1	-8.0	8.5	632.6	7.2	C12 H11 N3 O3 F

1.9 *Data of 6-chloro-3-hydroxy-3-((3-hydroxy-1H-pyrazol-5-yl)methyl)indolin-2-one (**15g**)*

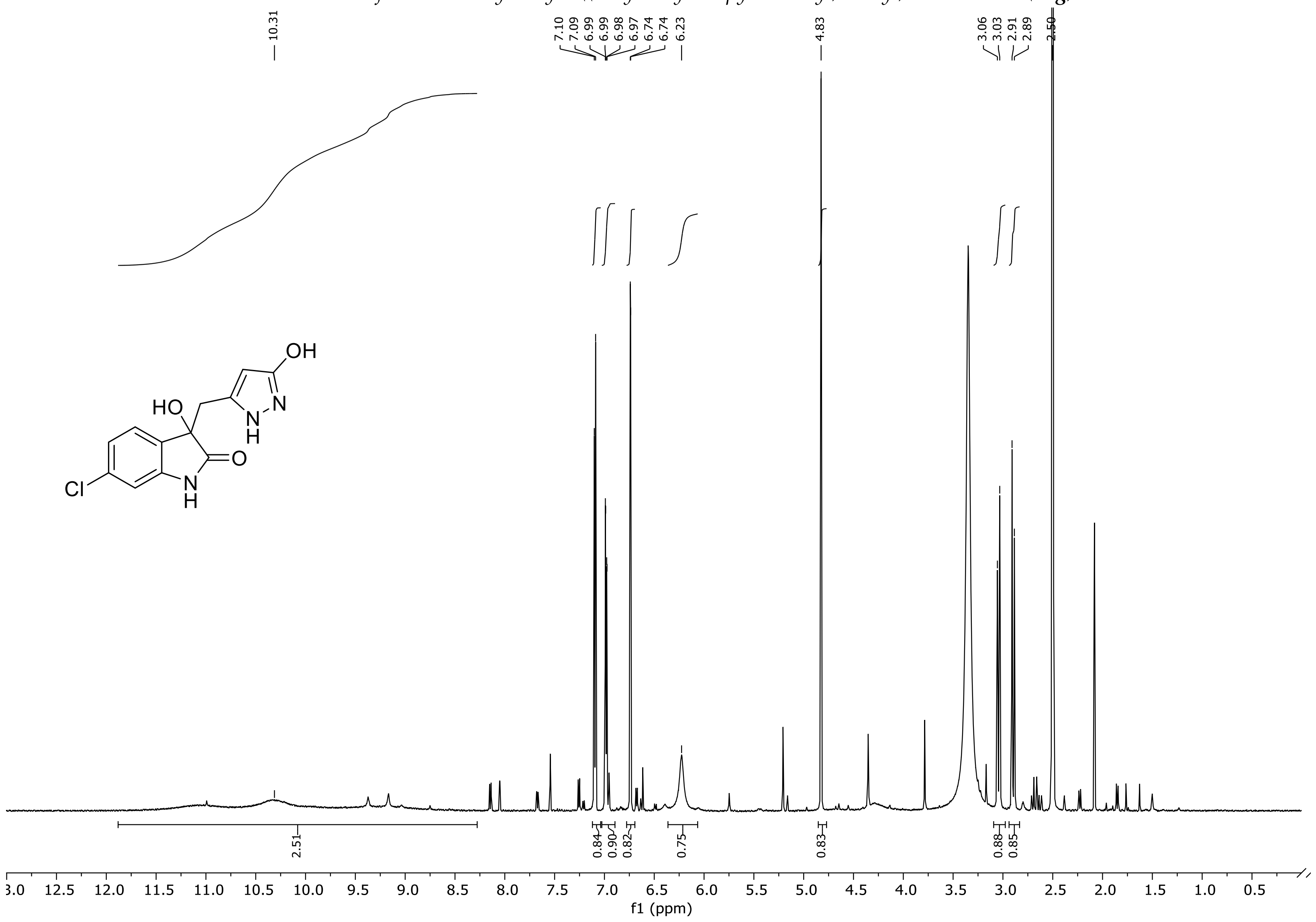

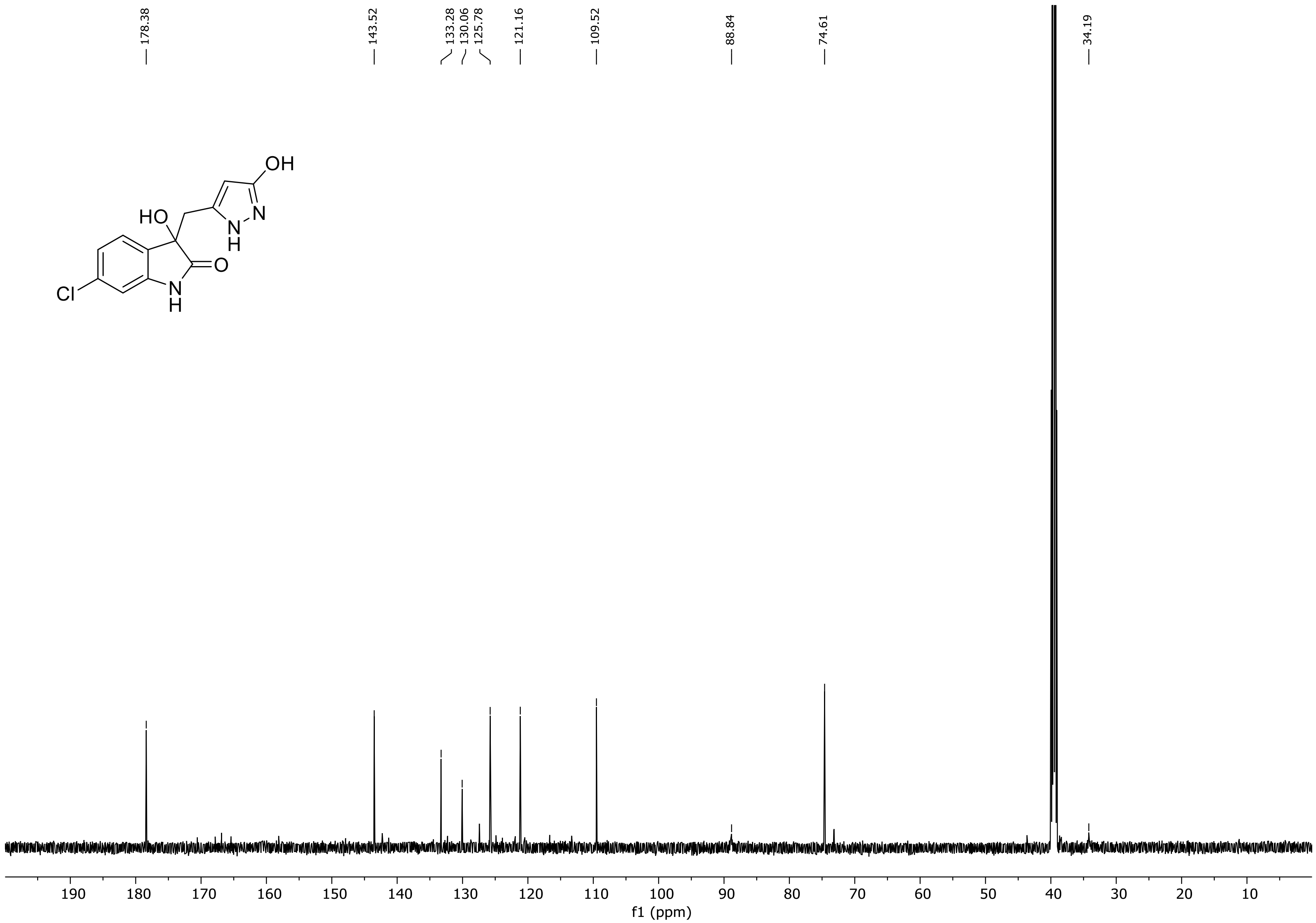
178.38
143.52
133.28
130.06
125.78
121.16
109.52
88.84
74.61
34.19
OH
HO
N
N
H
O
N
H
Cl
190
180
170
160
150
140
130
120
110
100
90
80
70
60
50
40
30
20
10
f1 (ppm)

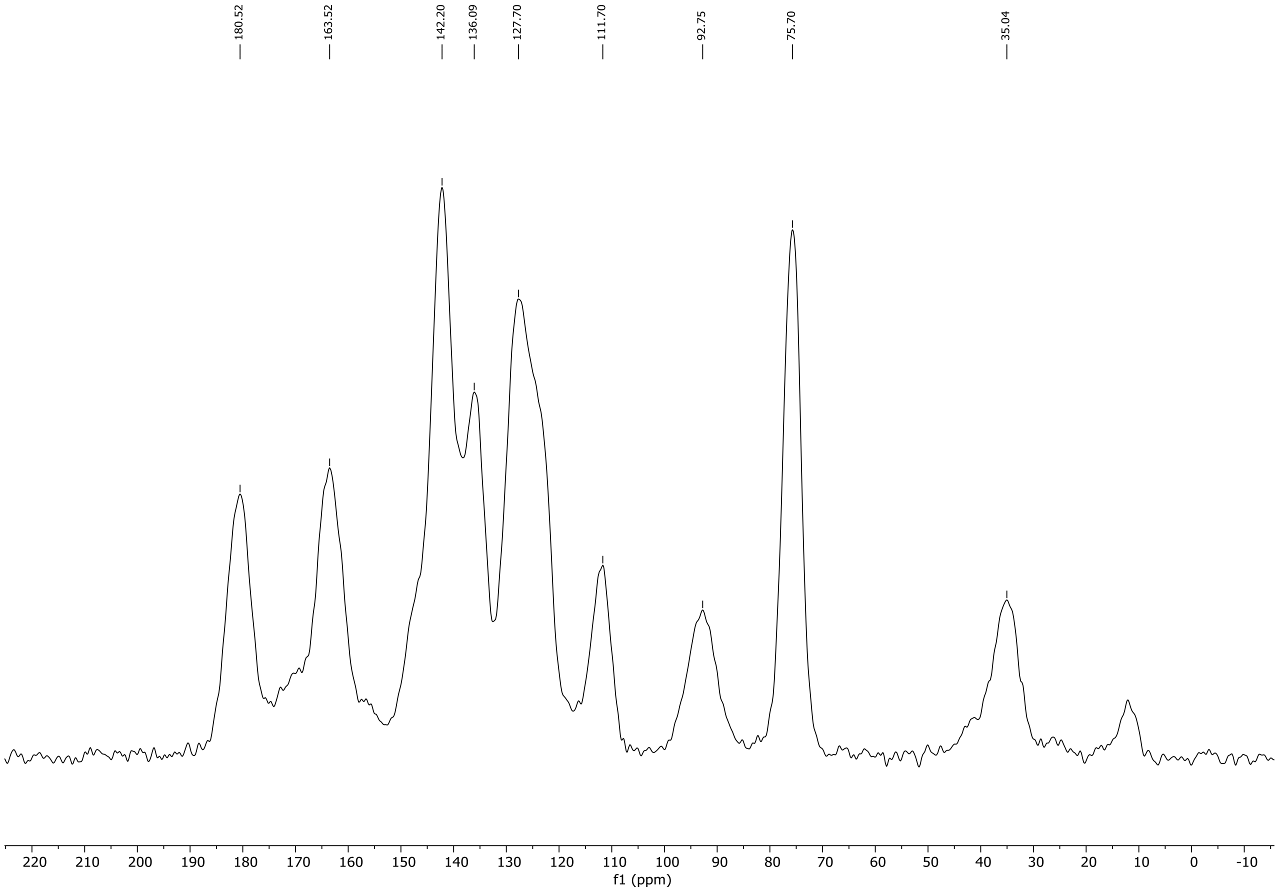

Solid-state carbon-13 NMR spectrum recorded with a 4 s recycle delay and 1 ms contact time.

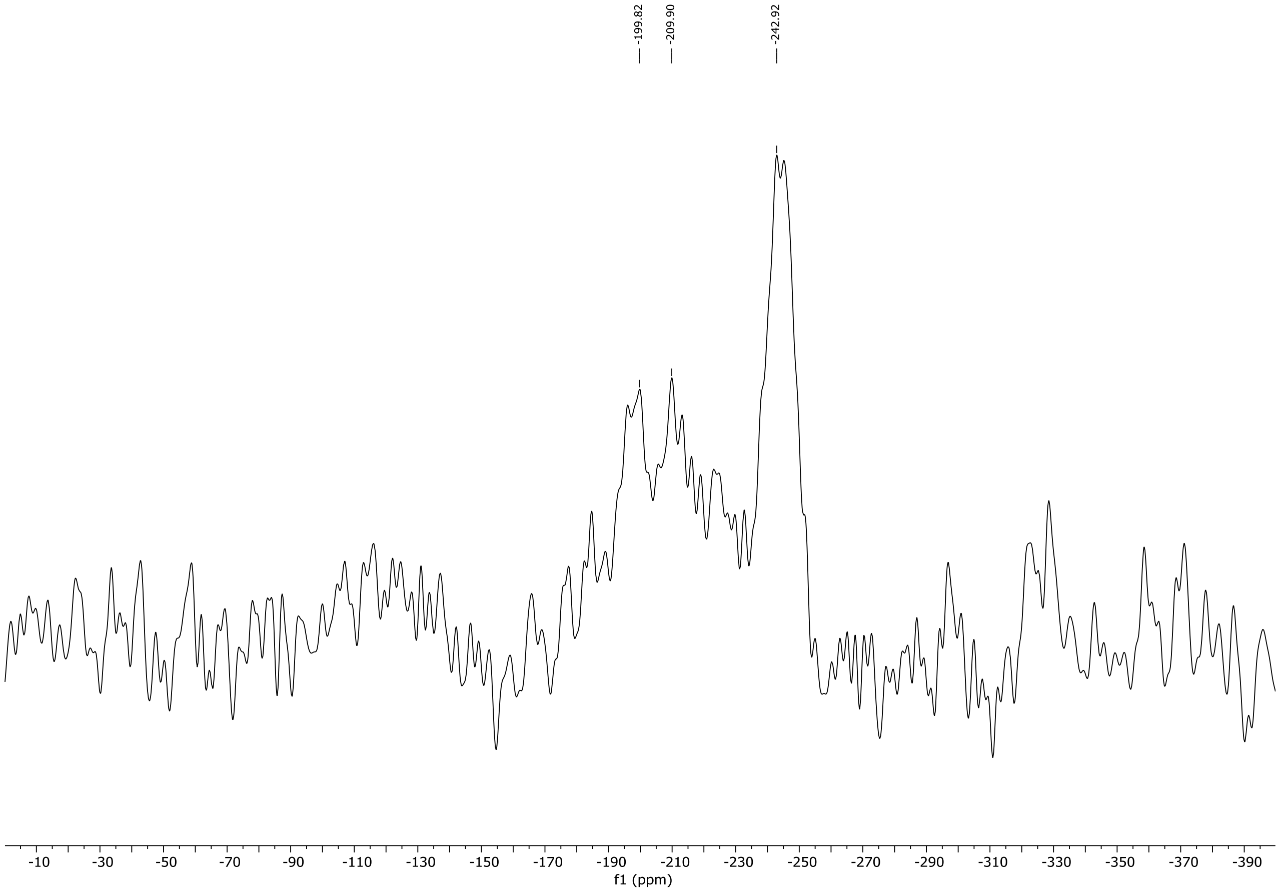

Solid-state nitrogen-15 NMR spectrum recorded with a 3 s recycle delay and 2 ms contact time.

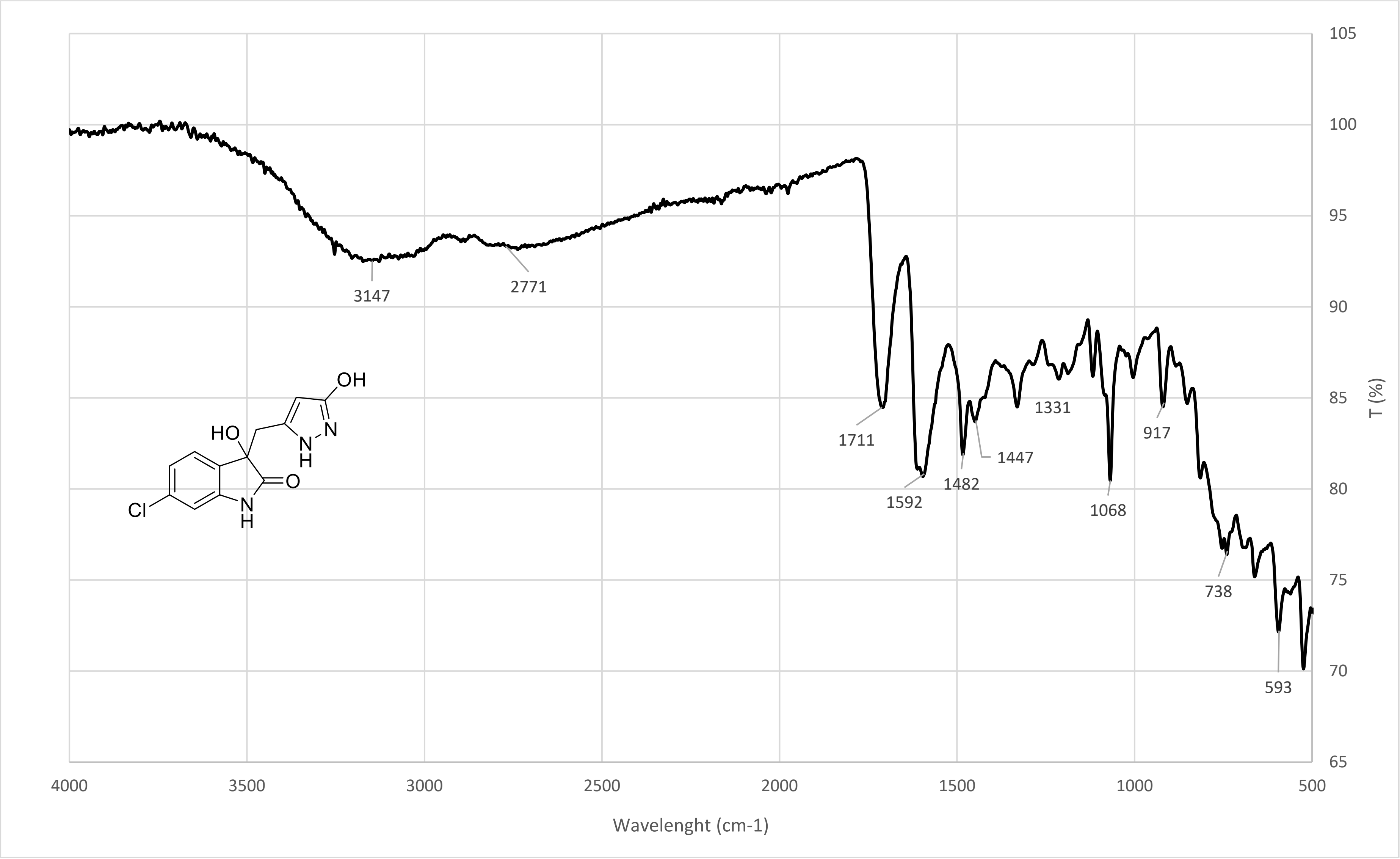
3147
2771
1711
1592
1482
1447
1331
1068
917
738
593
T (%)
Wavelenght (cm-1)
4000
3500
3000
2500
2000
1500
1000
500
105
100
95
90
85
80
75
70
65
OH
HO
N
N
H
Cl
O
N
H

Elemental Composition Report

Single Mass Analysis

Tolerance = 3.0 mDa / DBE: min = -1.5, max = 100.0
Element prediction: Off
Number of isotope peaks used for i-FIT = 5

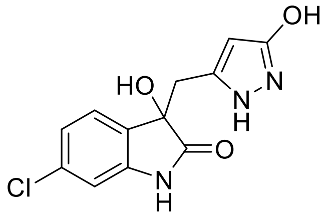

Monoisotopic Mass, Even Electron Ions
615 formula(e) evaluated with 4 results within limits (up to 500 closest results for each mass)
Elements Used:
C: 0-60 H: 0-80 N: 0-7 O: 0-10 Cl: 0-1 151Eu: 0-1

QToF Premier

29-May-2019
GG227B 230 (1.939) Cm (222:241)

1: TOF MS ES+
3.61e+005

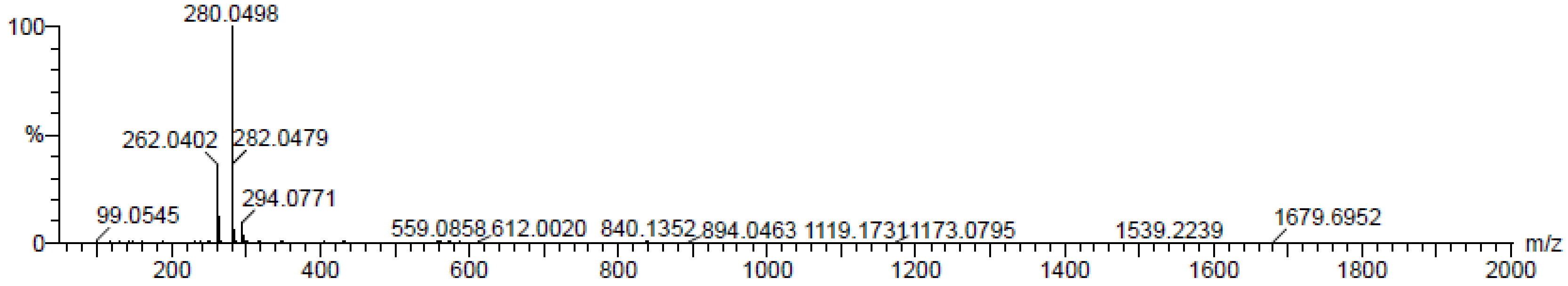

Mass	Calc. Mass	mDa	PPM	DBE	i-FIT	i-FIT (Norm)	Formula
Minimum:				-1.5			
Maximum:		3.0	5.0	100.0			
280.0498	280.0489	0.9	3.2	0.5	1230.9	26.2	C H10 N7 O10
	280.0489	0.9	3.2	8.5	1204.7	0.0	C12 H11 N3 O3 Cl
	280.0511	-1.3	-4.6	17.5	1230.9	26.1	C18 H6 N3 O
	280.0471	2.7	9.6	13.5	1230.0	25.3	C13 H6 N5 O3

*1.10 Data of 7-chloro-3-hydroxy-3-((3-hydroxy-1H-pyrazol-5-yl)methyl)indolin-2-one (**15h**)*

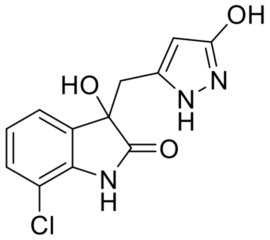

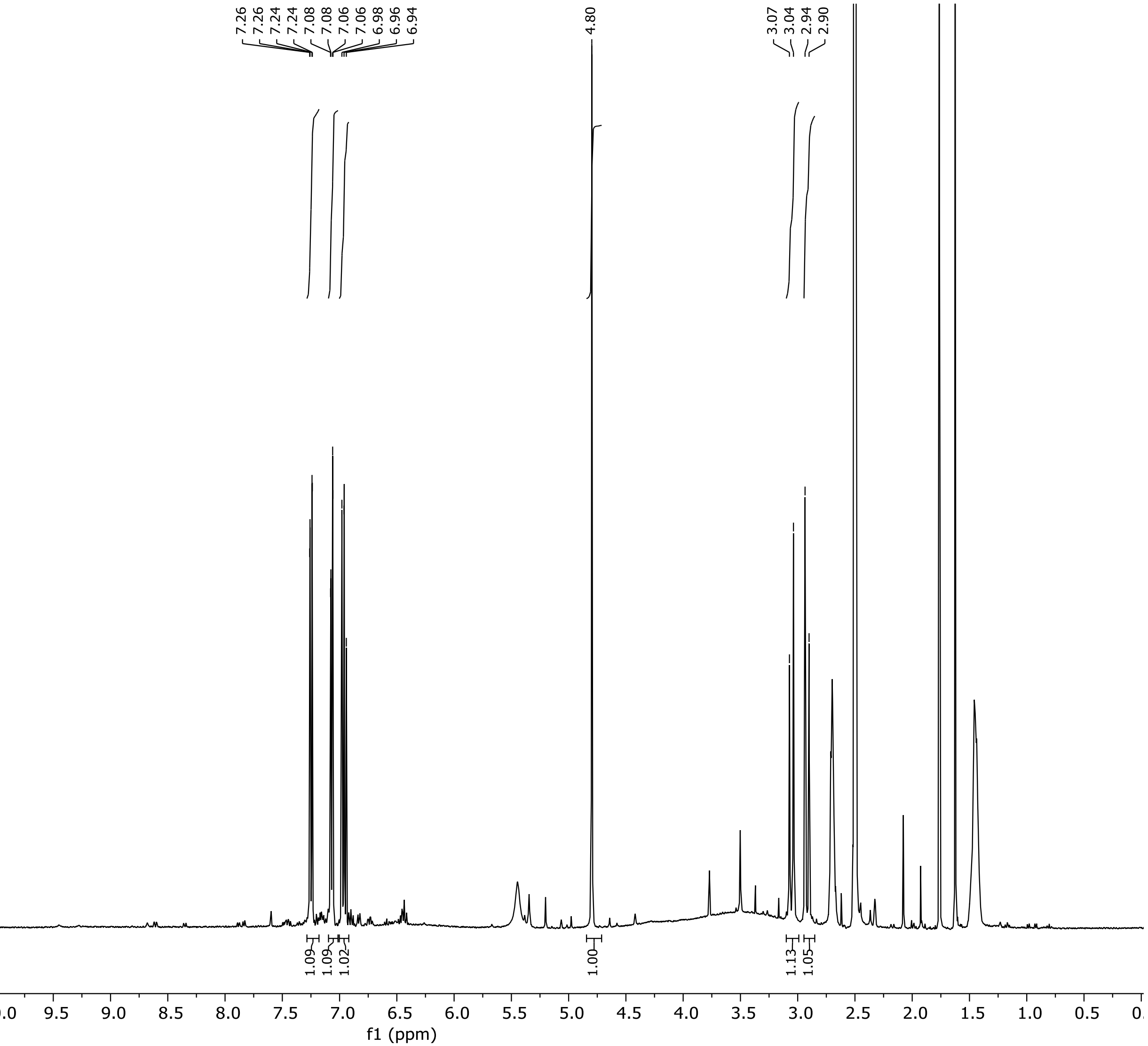

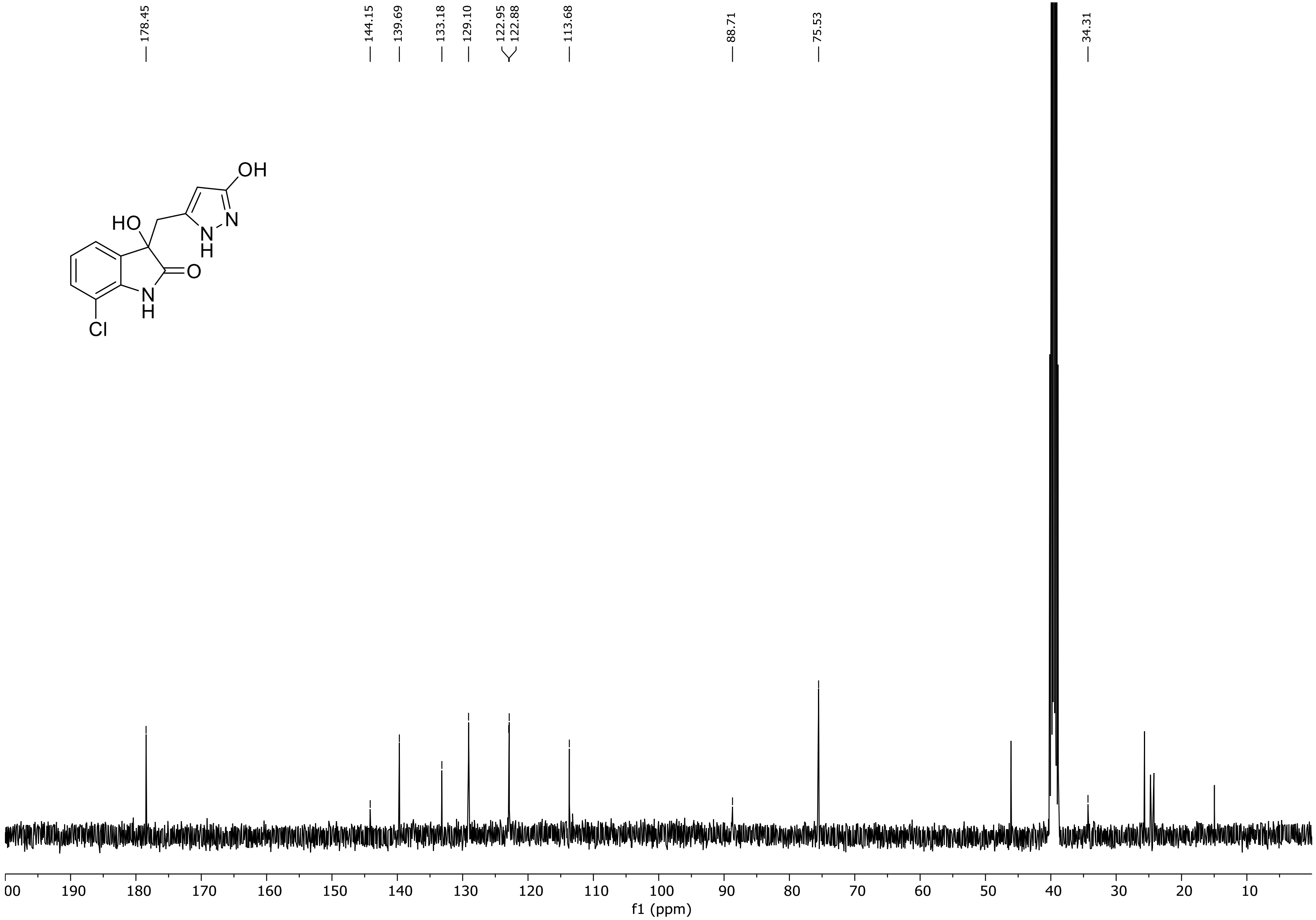
178.45
144.15
139.69
133.18
129.10
122.95
122.88
113.68
88.71
75.53
34.31
OH
HO
N
N
H
O
N
H
Cl
00
190
180
170
160
150
140
130
120
110
100
90
80
70
60
50
40
30
20
10
f1 (ppm)

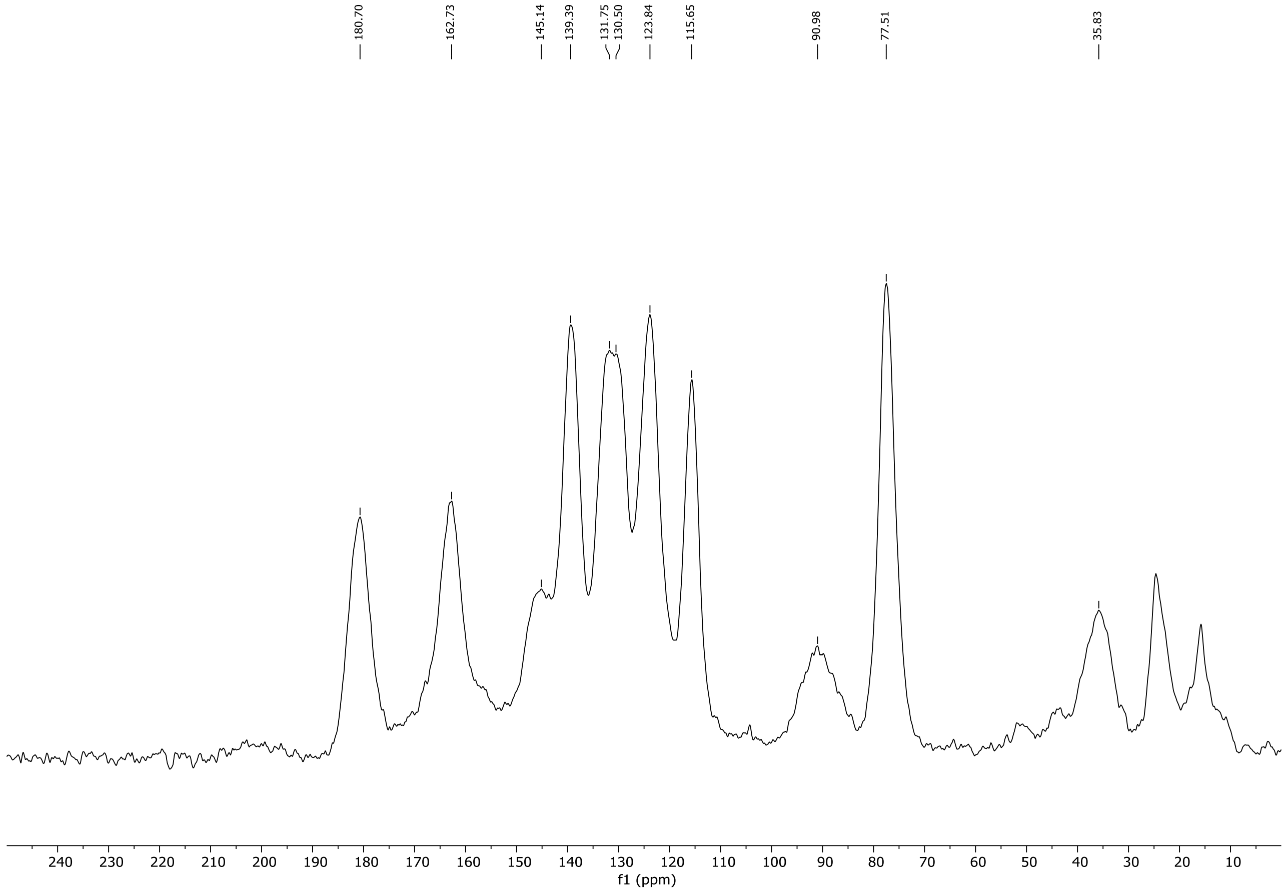

Solid-state carbon-13 NMR spectrum recorded with a 1 s recycle delay and 1 ms contact time.

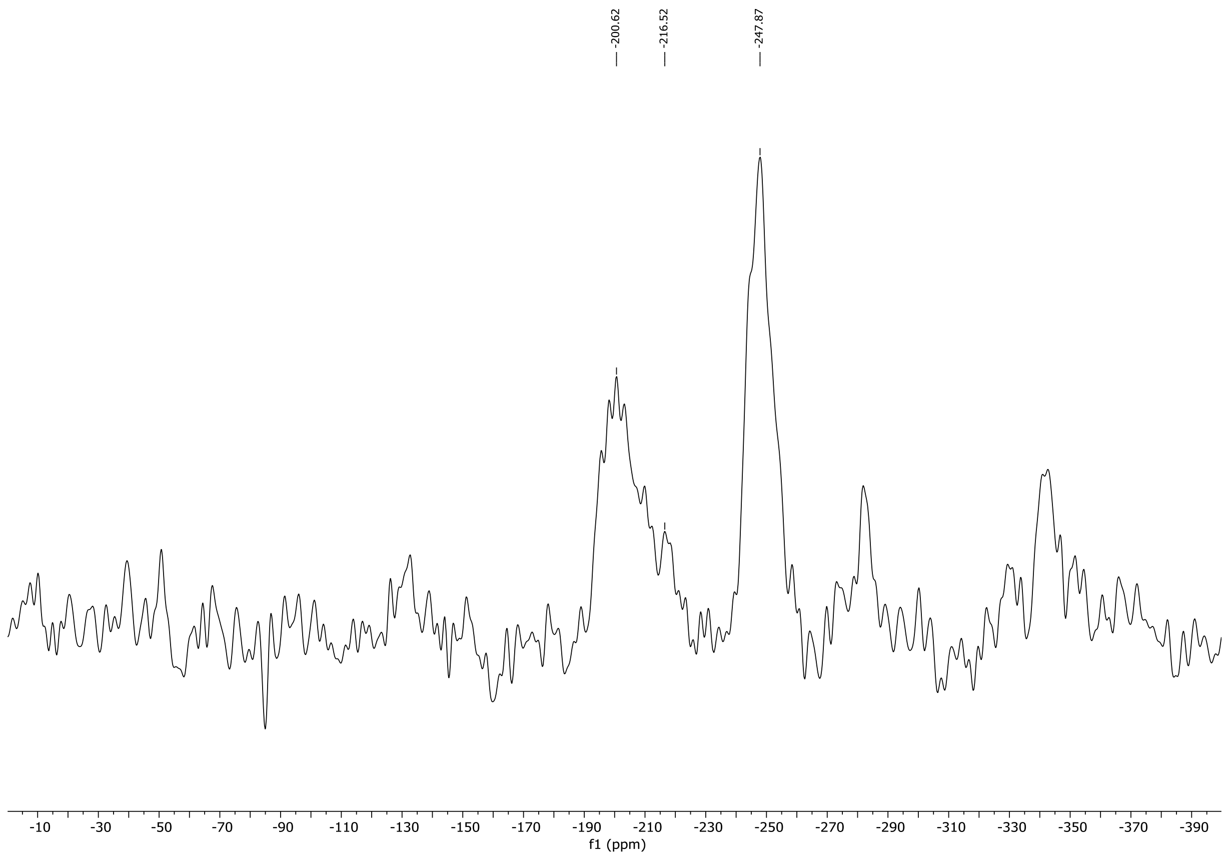

Solid-state nitrogen-15 NMR spectrum recorded with a 1s recycle delay and 1 ms contact time.

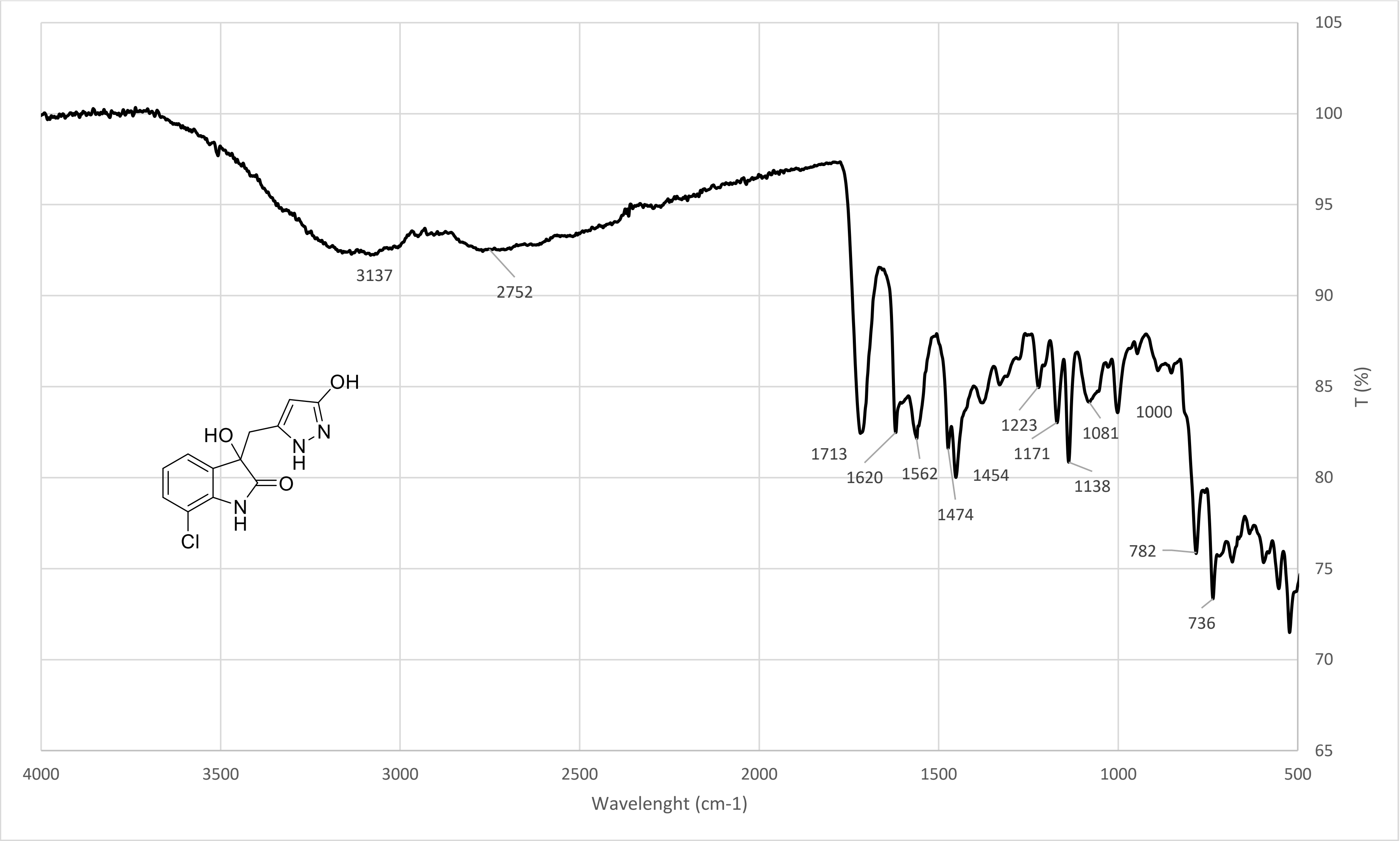

HO
OH
N
N
H
O
N
H
Cl
3137
2752
1713
1620
1562
1474
1454
1223
1171
1138
1081
1000
782
736
T (%)
Wavelenght (cm-1)

Elemental Composition Report

Single Mass Analysis

Tolerance = 3.0 mDa / DBE: min = -1.5, max = 100.0
Element prediction: Off
Number of isotope peaks used for i-FIT = 5

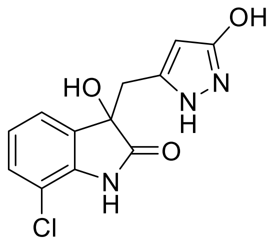

Monoisotopic Mass, Even Electron Ions
477 formula(e) evaluated with 5 results within limits (up to 500 closest results for each mass)
Elements Used:
C: 0-60 H: 0-90 N: 0-6 O: 0-5 Cl: 0-3

QToF Premier

23-Aug-2019
GG265A 223 (1.884) Cm (223)

1: TOF MS ES+
1.43e+004

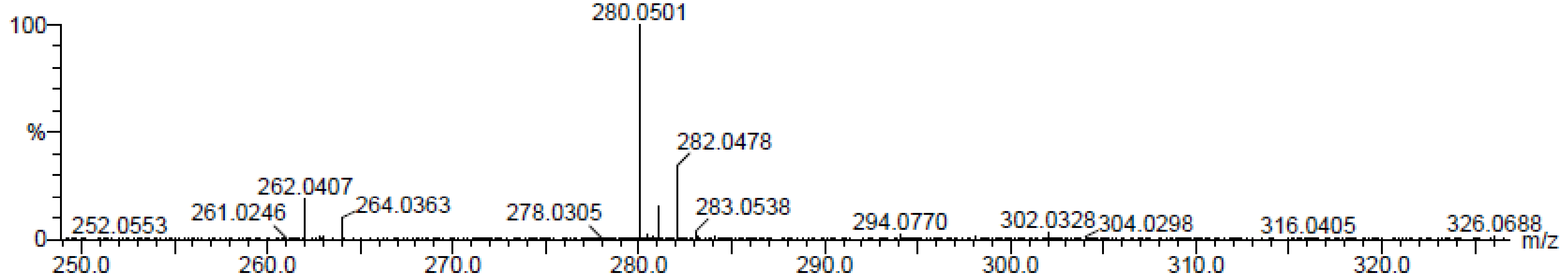

Minimum:				-1.5			
Maximum:		3.0	5.0	100.0			

Mass	Calc. Mass	mDa	PPM	DBE	i-FIT	i-FIT (Norm)	Formula
280.0501	280.0499	0.2	0.7	-0.5	395.6	18.5	C6 H17 N5 O Cl3
	280.0507	-0.6	-2.1	3.5	391.6	14.4	C11 H16 N O3 Cl2
	280.0511	-1.0	-3.6	17.5	397.0	19.9	C18 H6 N3 O
	280.0489	1.2	4.3	8.5	377.2	0.0	C12 H11 N3 O3 Cl
	280.0529	-2.8	-10.0	12.5	380.9	3.8	C17 H11 N O Cl

*1.11 Data of 3-hydroxy-3-((3-hydroxy-1H-pyrazol-5-yl)methyl)-7-(trifluoromethyl)indolin-2-one (**15i**)*

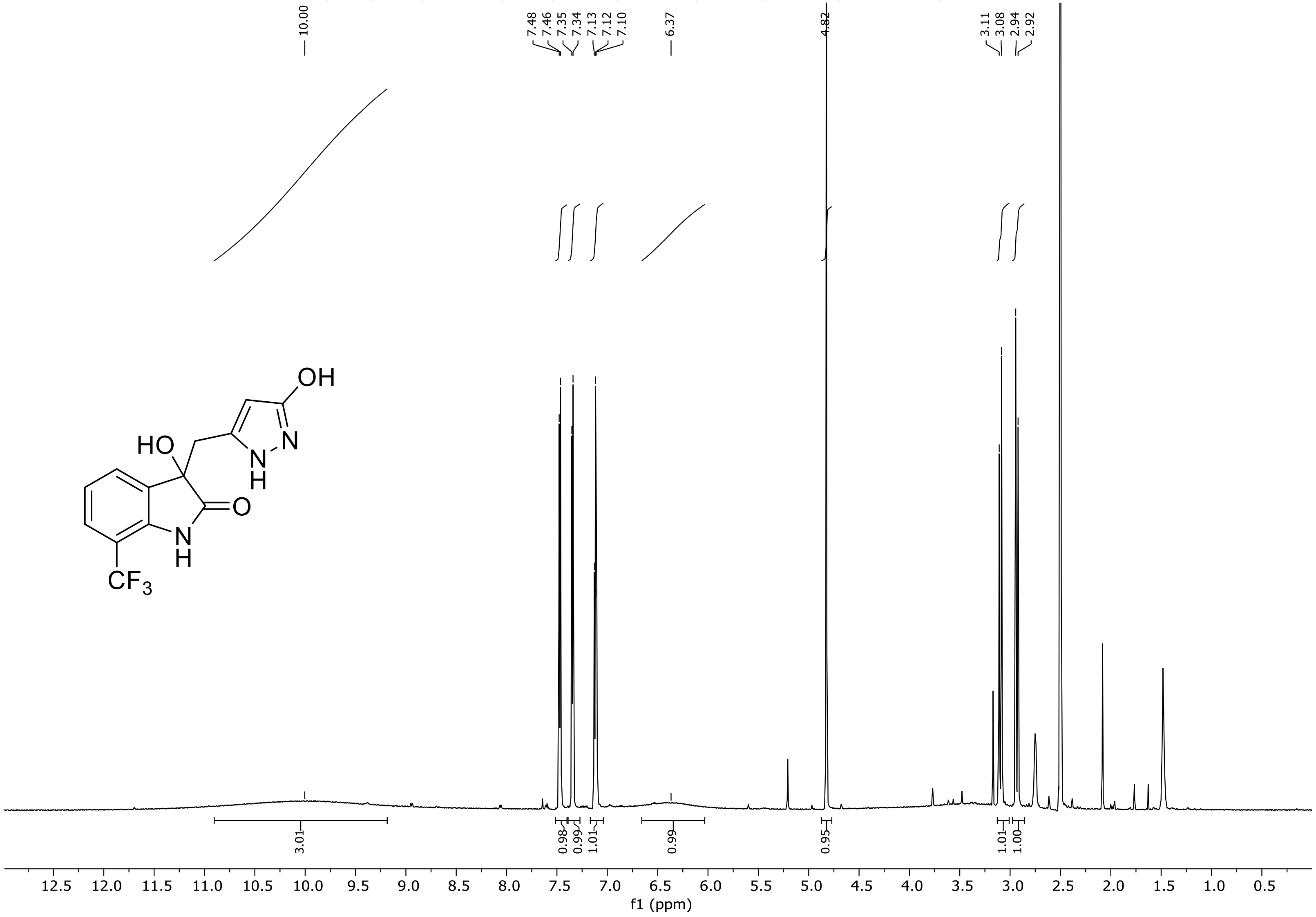

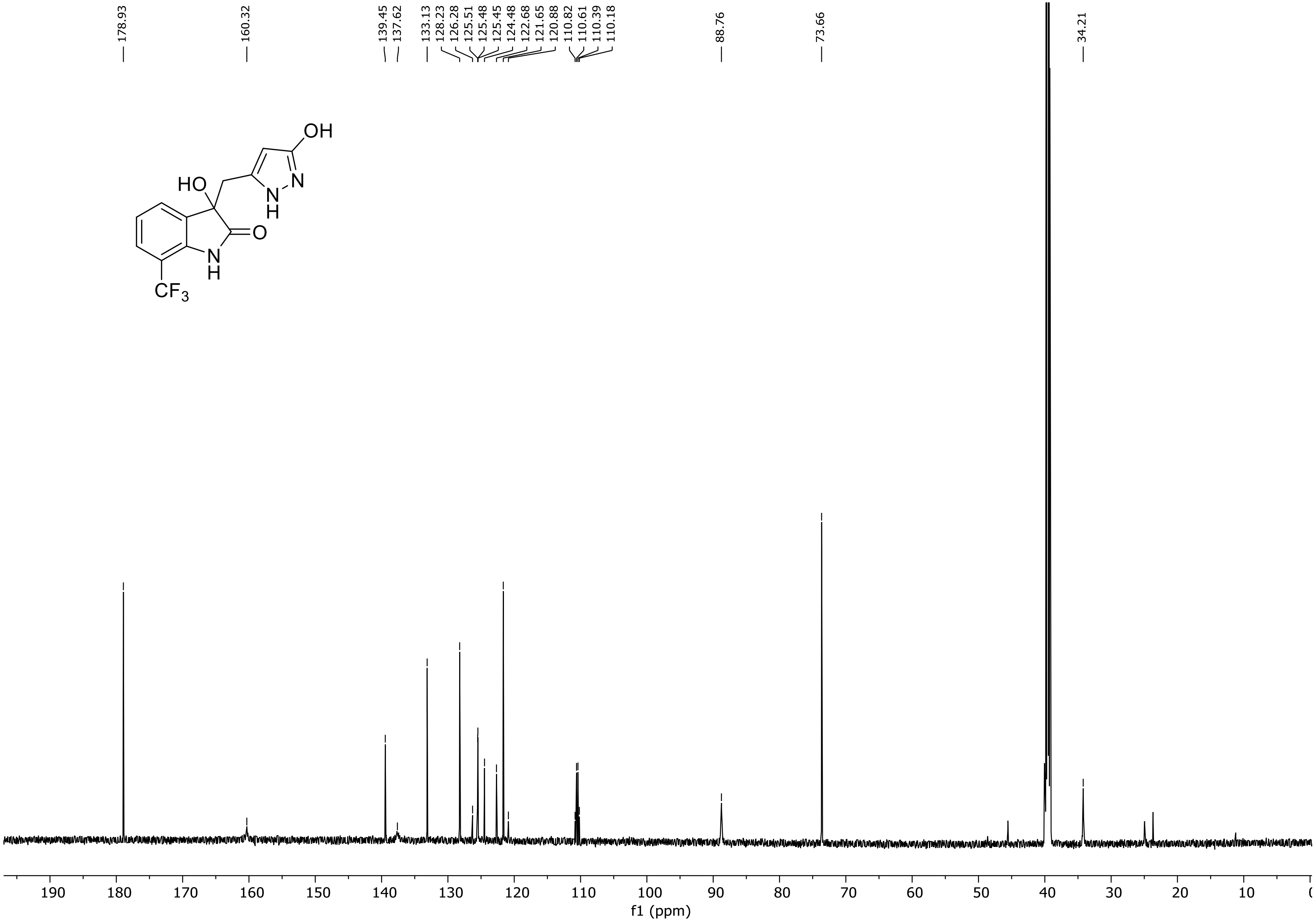
178.93
160.32
139.45
137.62
133.13
128.23
126.28
125.51
125.48
125.45
124.48
122.68
121.65
120.88
110.82
110.61
110.39
110.18
88.76
73.66
34.21
OH
HO
N
N
H
O
N
H
CF3
190 180 170 160 150 140 130 120 110 100 90 80 70 60 50 40 30 20 10
f1 (ppm)

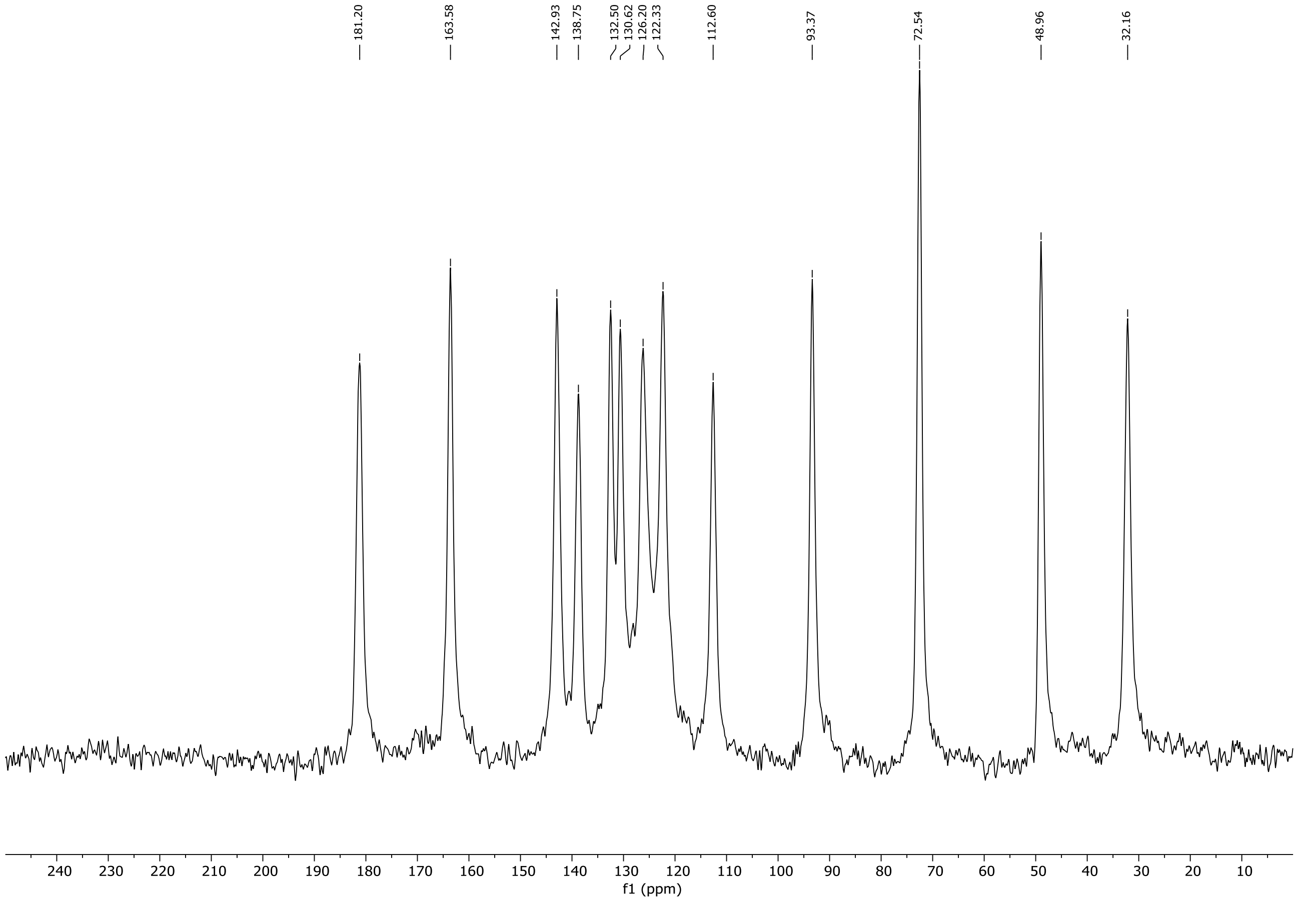

Solid-state carbon-13 NMR spectrum recorded with a 3 s recycle delay and 4 ms contact time.

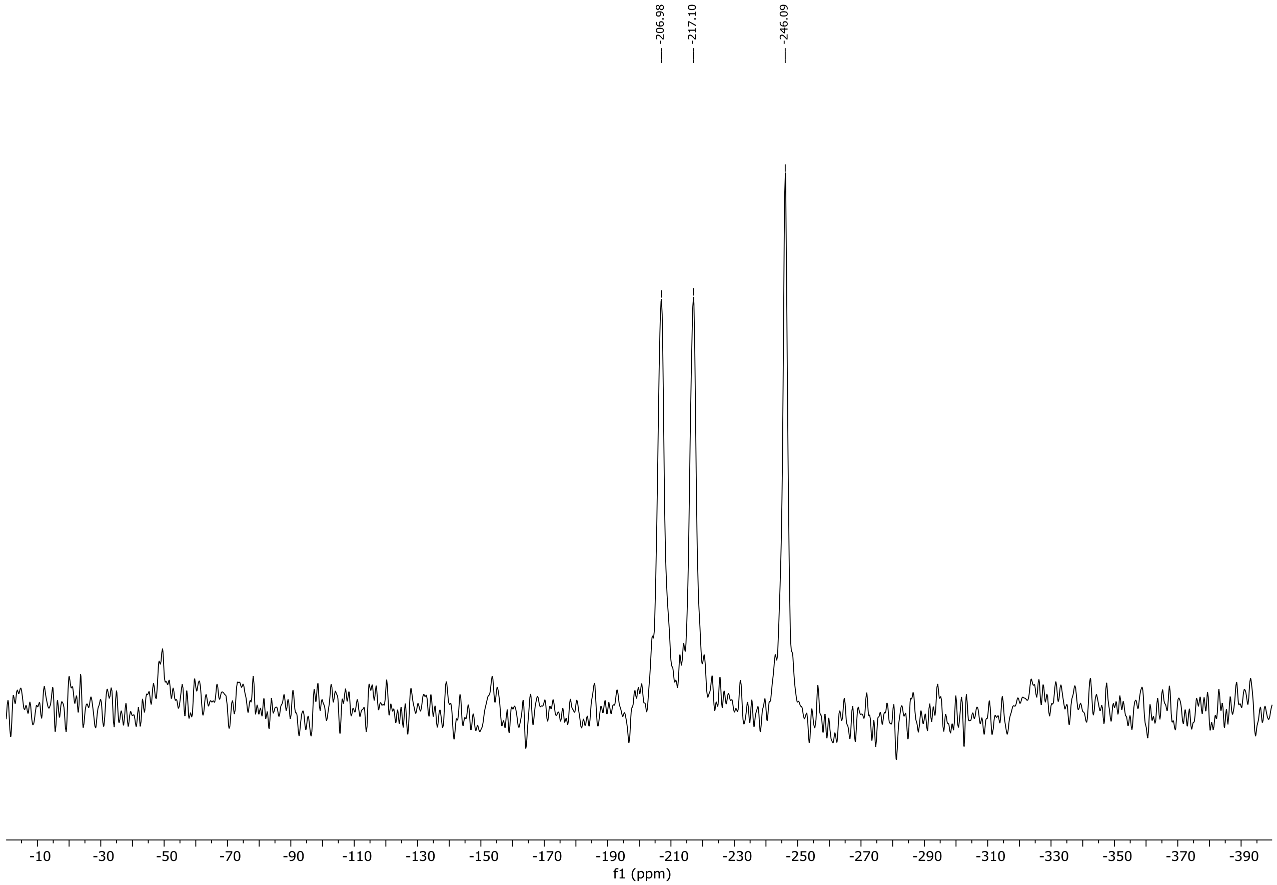

Solid-state nitrogen-15 NMR spectrum recorded with a 3 s recycle delay and 10 ms contact time.

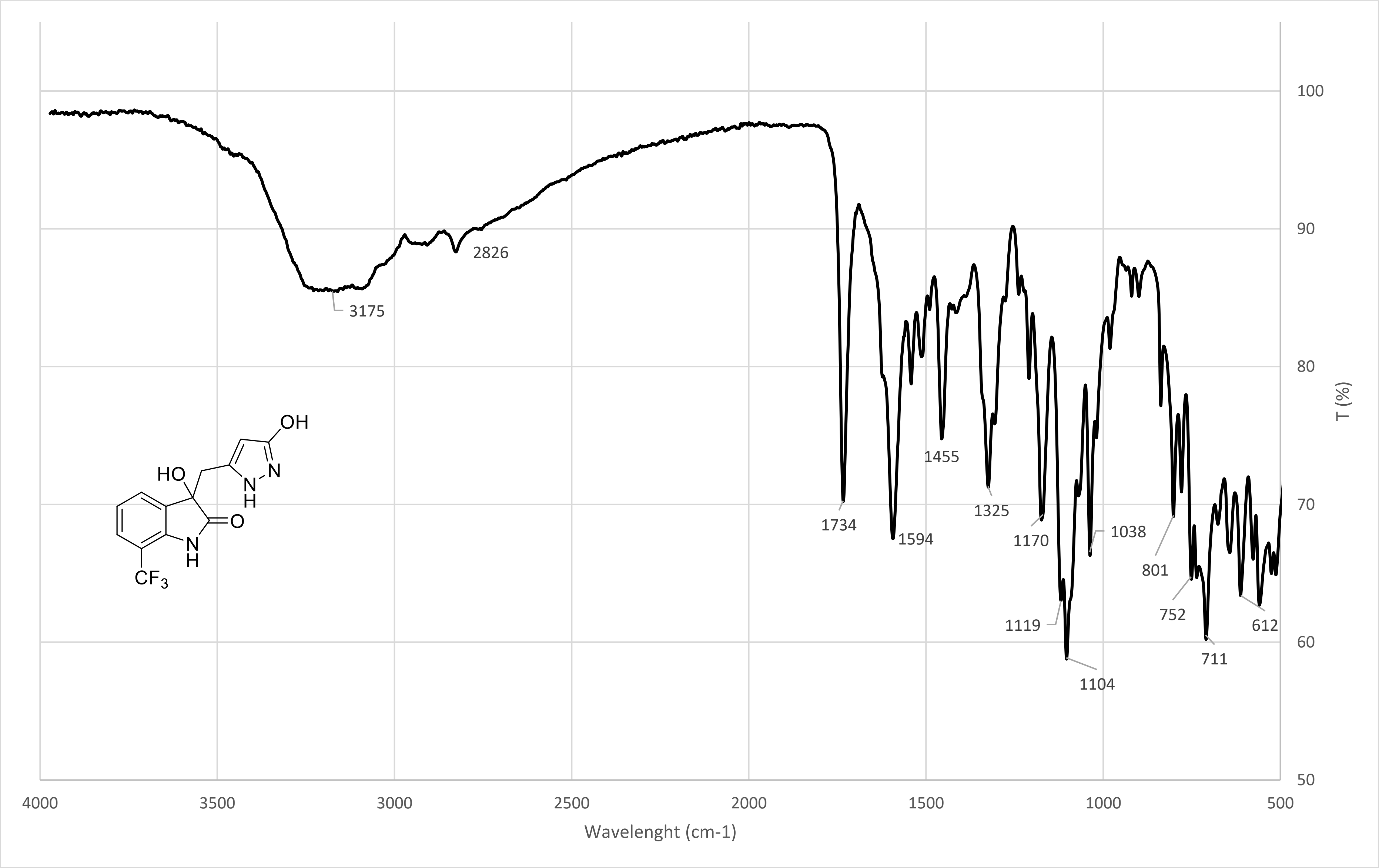
3175
2826
1734
1594
1455
1325
1170
1119
1104
1038
801
752
711
612
100
90
80
70
60
50
T (%)
4000
3500
3000
2500
2000
1500
1000
500
Wavelenght (cm-1)
HO
OH
N
N
H
O
H
CF_3

Single Mass Analysis
Tolerance = 5.0 PPM / DBE: min = -1.5, max = 100.0
Element prediction: Off
Number of isotope peaks used for i-FIT = 5

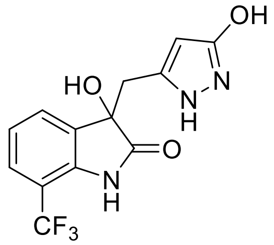

Monoisotopic Mass, Even Electron Ions
518 formula(e) evaluated with 1 results within limits (up to 500 closest results for each mass)
Elements Used:
C: 0-80 H: 0-60 N: 0-5 O: 0-5 F: 0-3

22-Jan-2019
GG150A 241 (2.054) Cm (232:253)

1: TOF MS ES+
5.51e+005

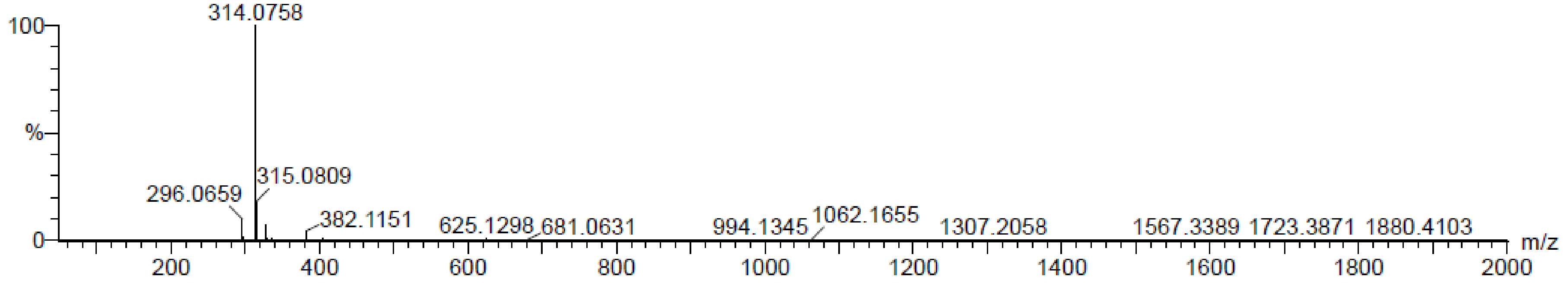

	Mass	Calc. Mass	mDa	PPM	DBE	i-FIT	i-FIT (Norm)	Formula
Minimum:					-1.5			
Maximum:			3.0	5.0	100.0			
	314.0758	314.0753	0.5	1.6	8.5	1101.5	0.0	C13 H11 N3 O3 F3

*1.12 Data of 7-fluoro-3-hydroxy-3-((3-hydroxy-1H-pyrazol-5-yl)methyl)indolin-2-one (**15j**)*

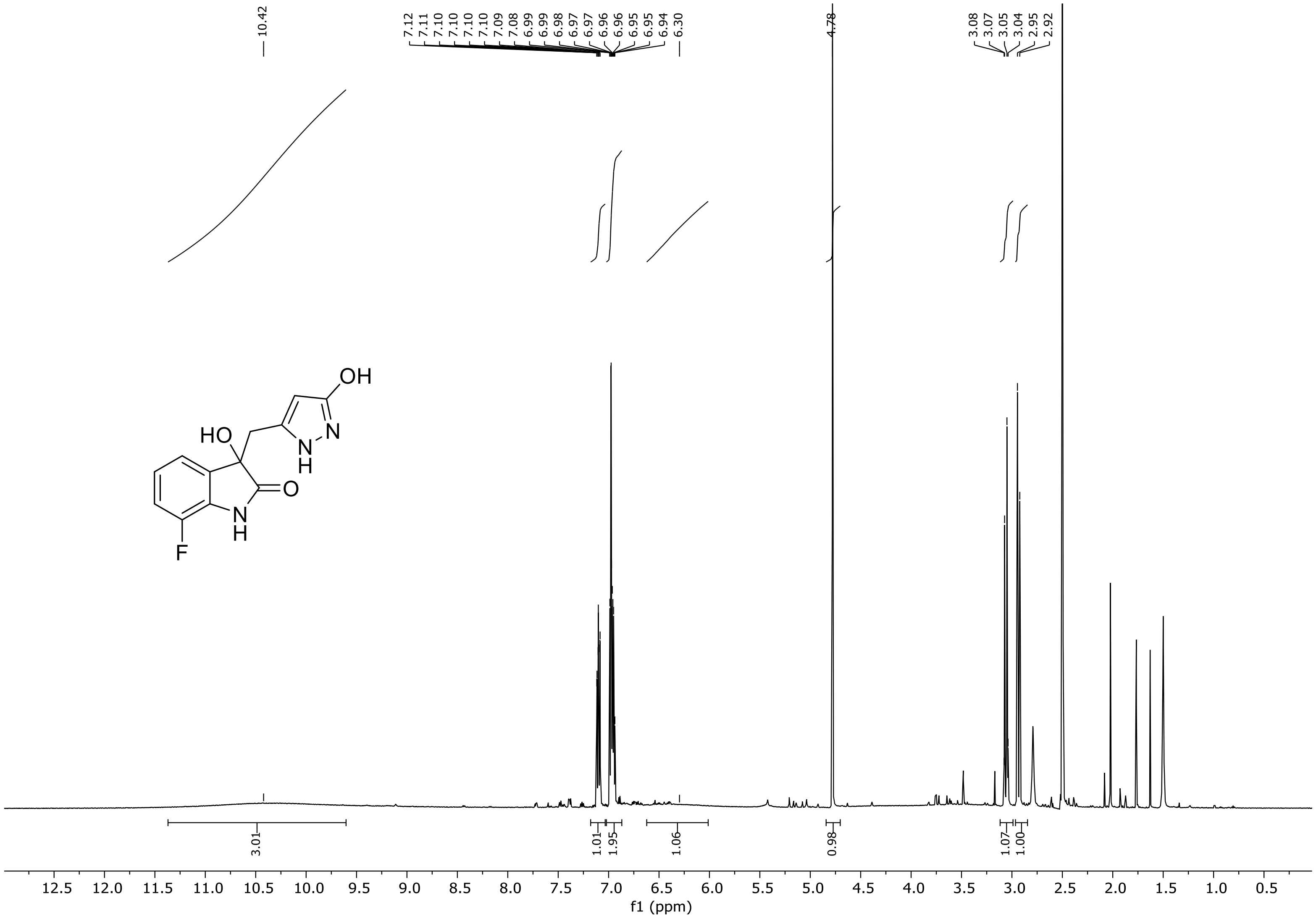

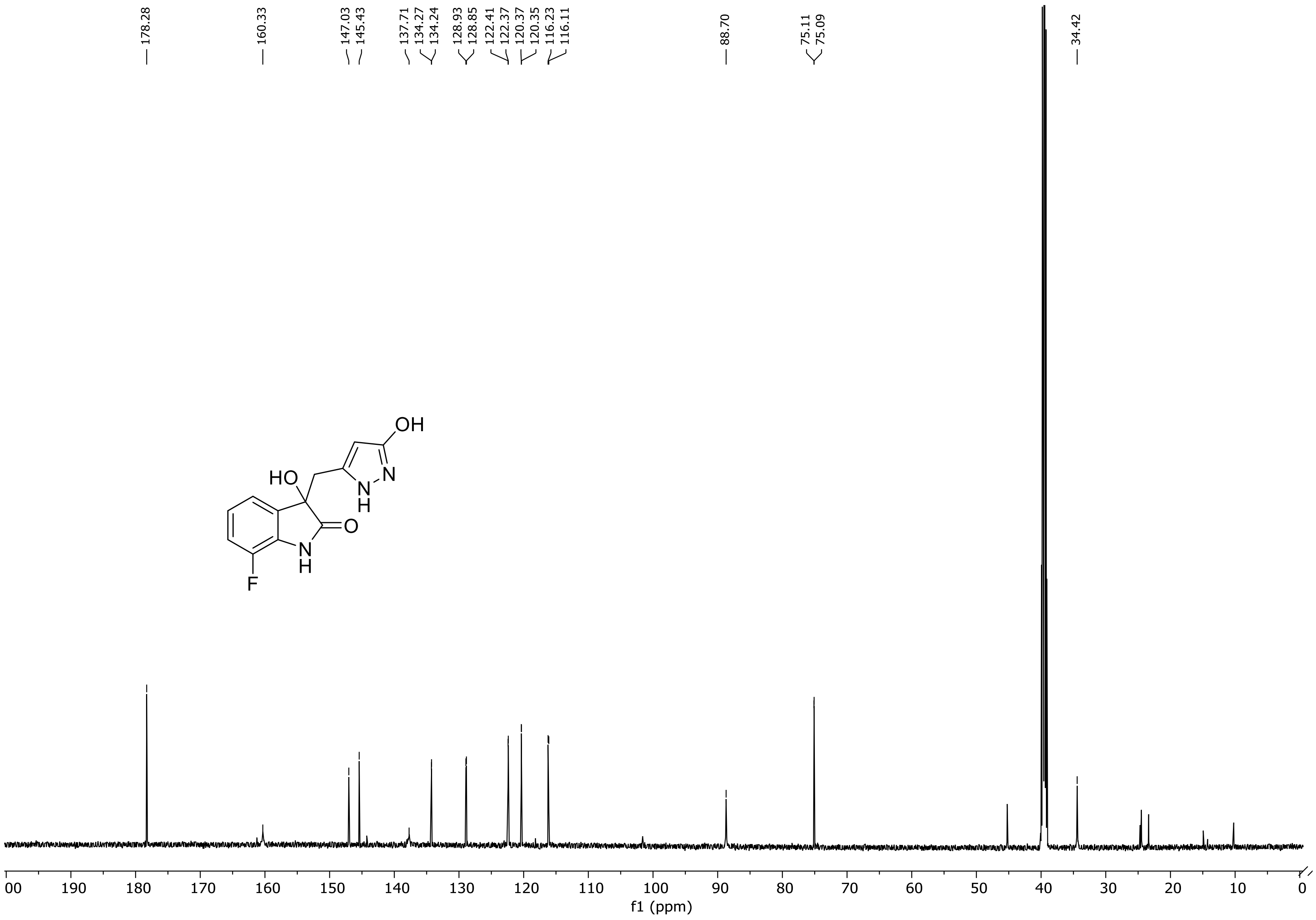
178.28
160.33
147.03
145.43
137.71
134.27
134.24
128.93
128.85
122.41
122.37
120.37
120.35
116.23
116.11
88.70
75.11
75.09
34.42
HO
OH
N
N
H
O
N
H
F
00
190
180
170
160
150
140
130
120
110
100
90
80
70
60
50
40
30
20
10
0
f1 (ppm)

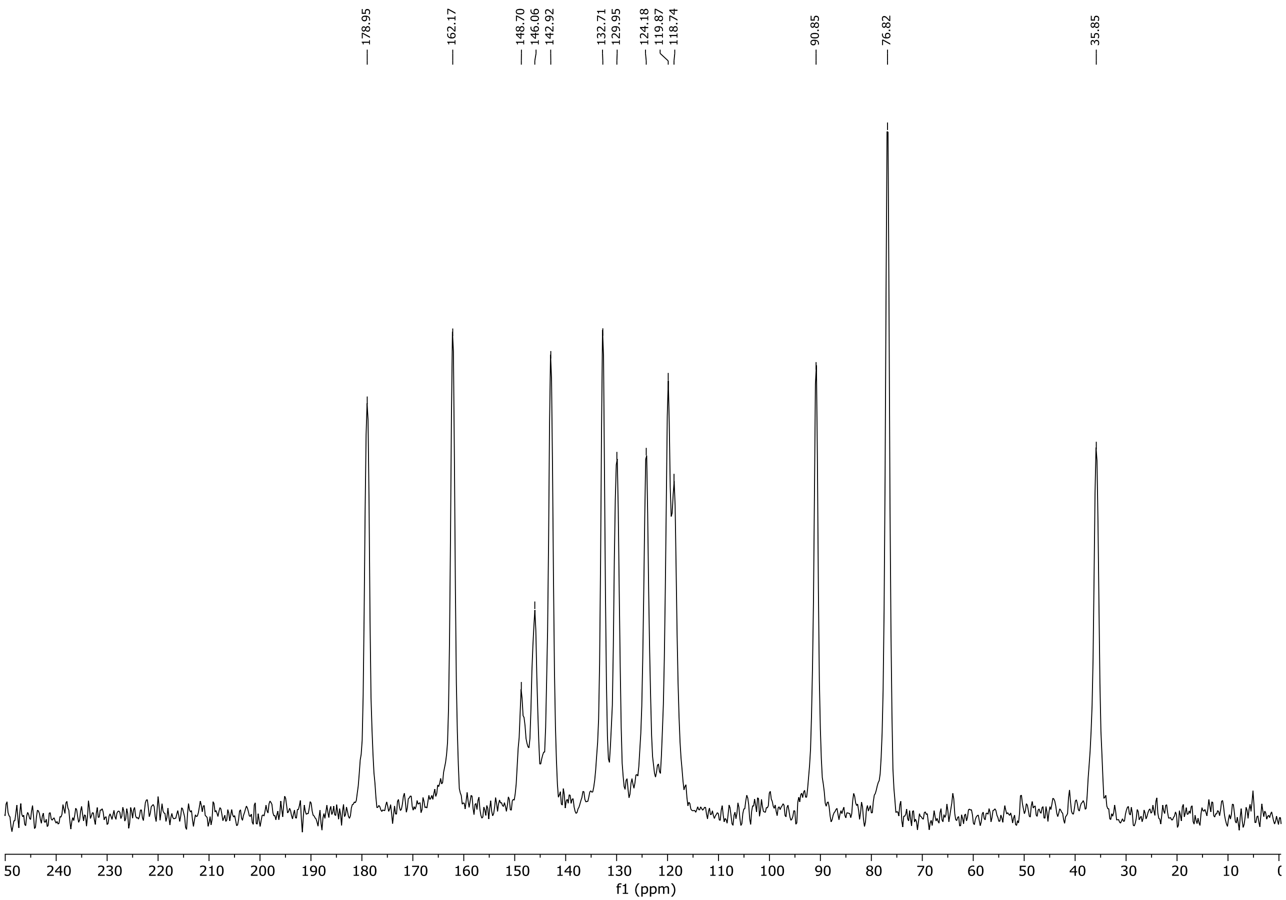

Solid-state carbon-13 NMR spectrum recorded with a 30 s recycle delay and 10 ms contact time.

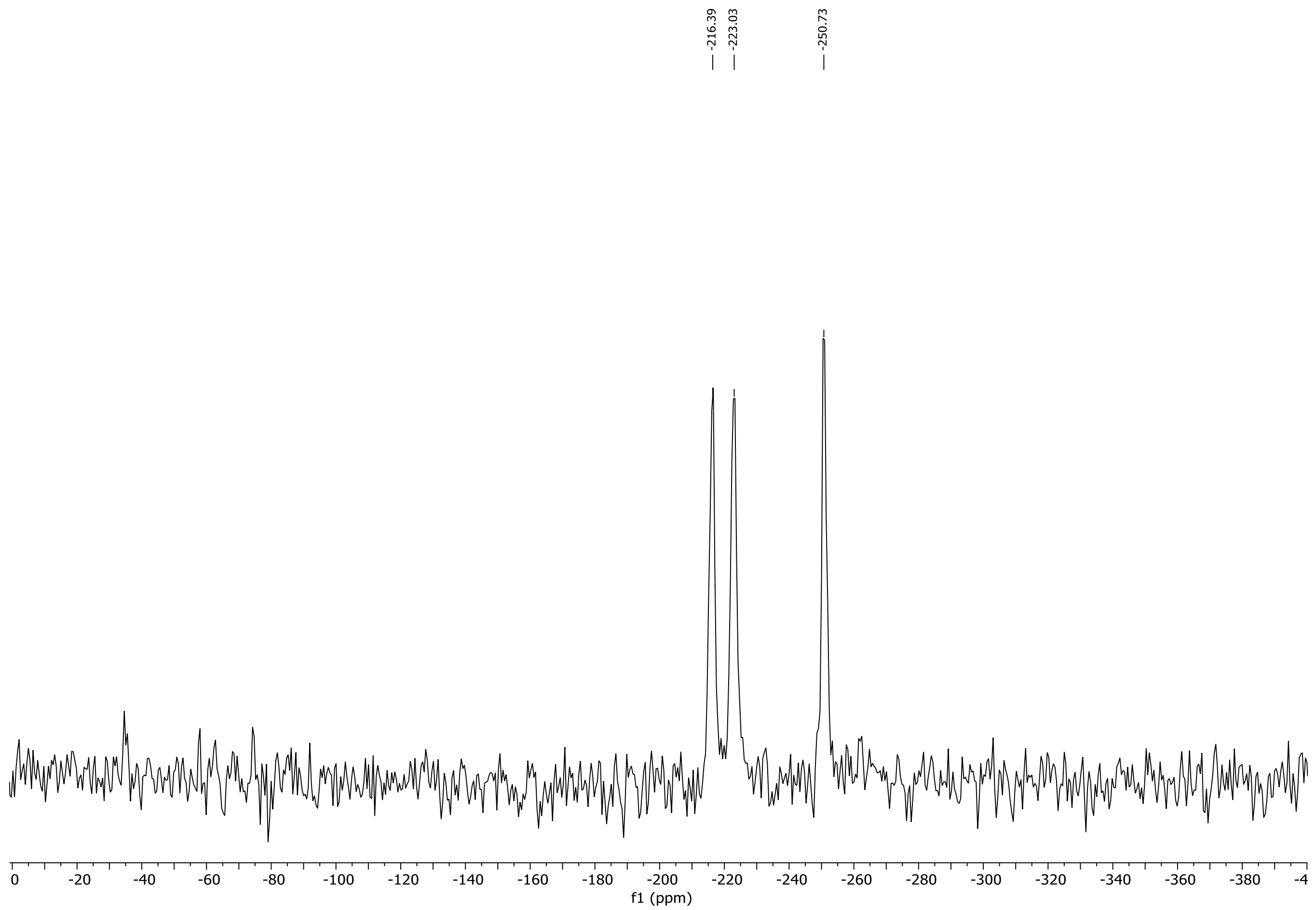

Solid-state nitrogen-15 NMR spectrum recorded with a 30 s recycle delay and 10 ms contact time.

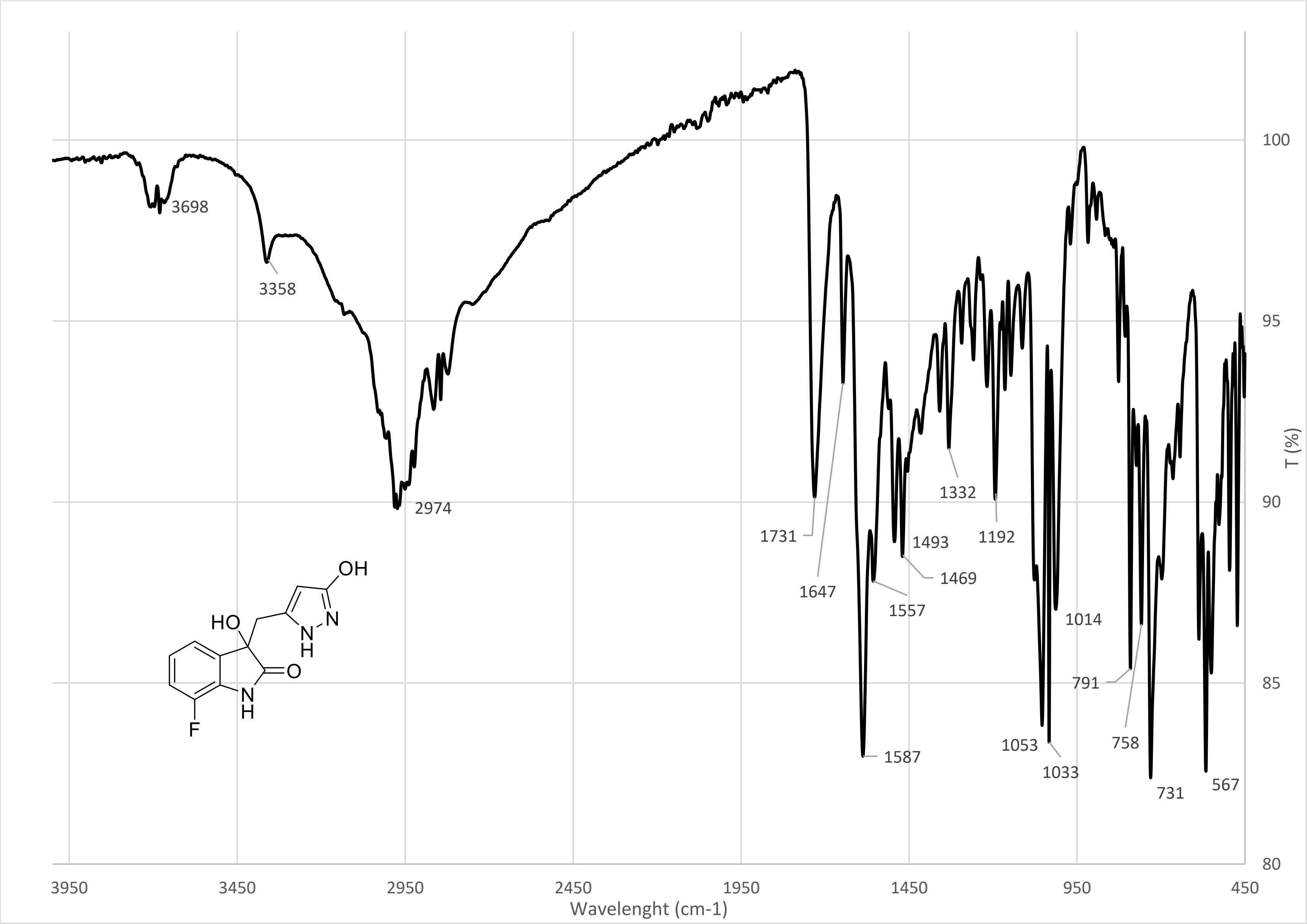
3698
3358
2974
1731
1647
1587
1557
1493
1469
1332
1192
1053
1033
1014
791
758
731
567
OH
HO
N
N
H
O
NH
H
F
3950
3450
2950
2450
1950
1450
950
450
Wavelenght (cm-1)
100
95
90
85
80
T (%)

Single Mass Analysis

Tolerance = 3.0 mDa / DBE: min = -1.5, max = 100.0
Element prediction: Off
Number of isotope peaks used for i-FIT = 5

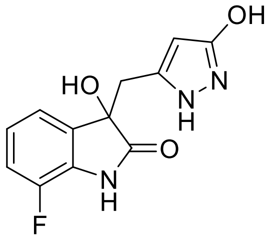

Monoisotopic Mass, Even Electron Ions
316 formula(e) evaluated with 3 results within limits (up to 500 closest results for each mass)
Elements Used:
C: 0-40 H: 0-80 N: 0-4 O: 0-4 F: 0-3

QToF Premier

21-Nov-2019
GG301A 199 (1.687) Cm (199:223)

1: TOF MS ES+
3.68e+004

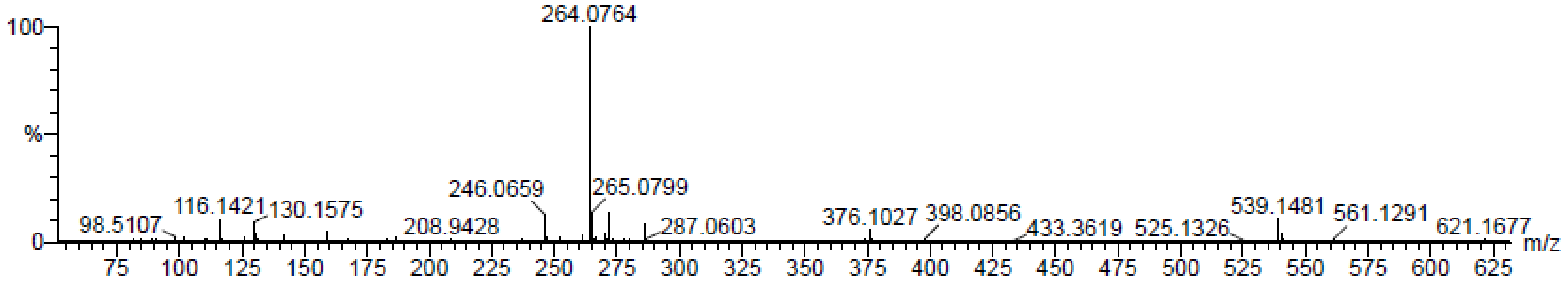

Minimum:				-1.5			
Maximum:		3.0	5.0	100.0			

Mass	Calc. Mass	mDa	PPM	DBE	i-FIT	i-FIT (Norm)	Formula
264.0764	264.0773	-0.9	-3.4	12.5	794.2	2.8	C15 H10 N3 O2
	264.0749	1.5	5.7	9.5	794.1	2.8	C13 H9 N3 F3
	264.0784	-2.0	-7.6	8.5	791.5	0.1	C12 H11 N3 O3 F

*1.13 Data of 1-benzyl-3-hydroxy-3-((3-hydroxy-1H-pyrazol-5-yl)methyl)indolin-2-one (**15l**)*

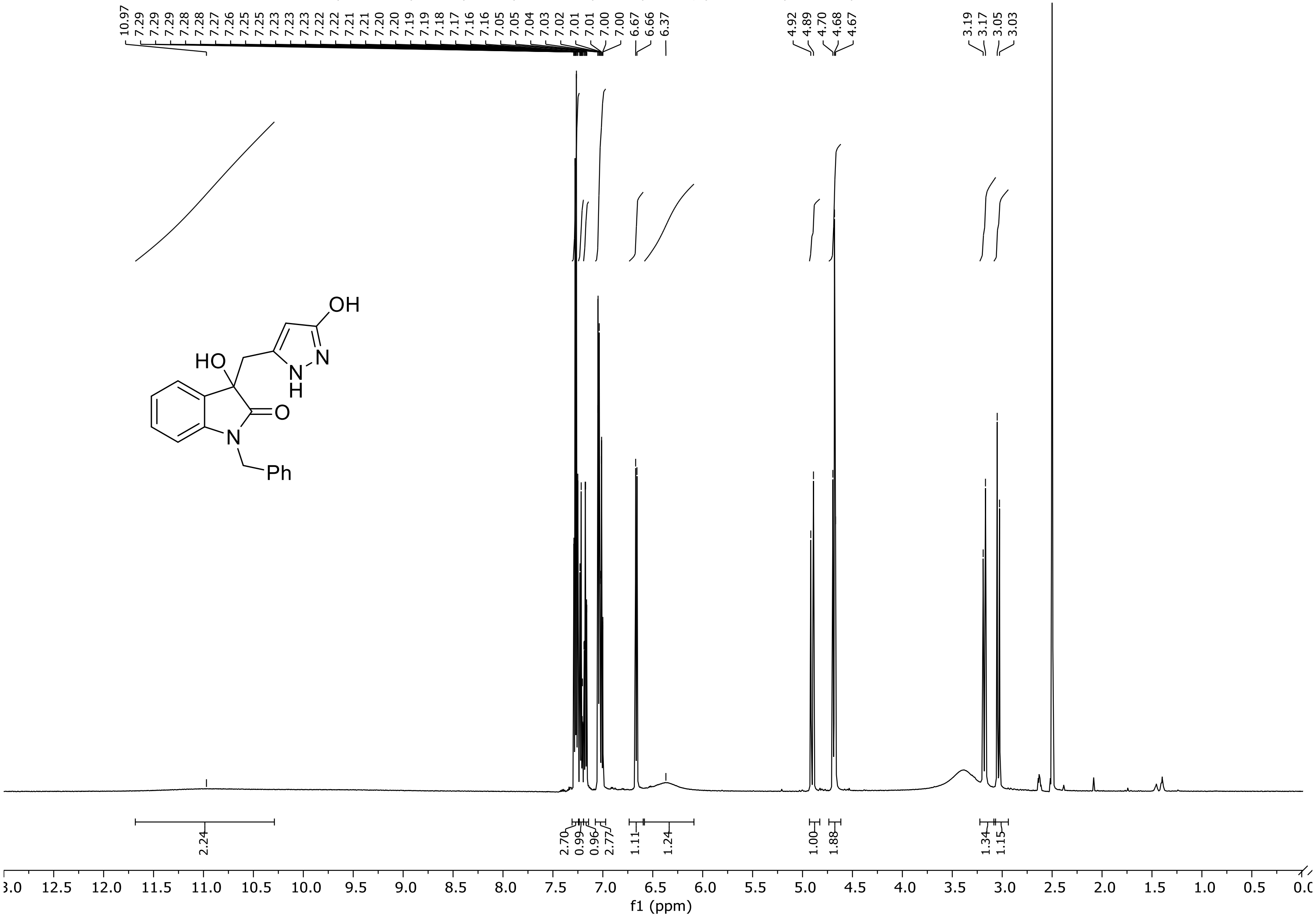

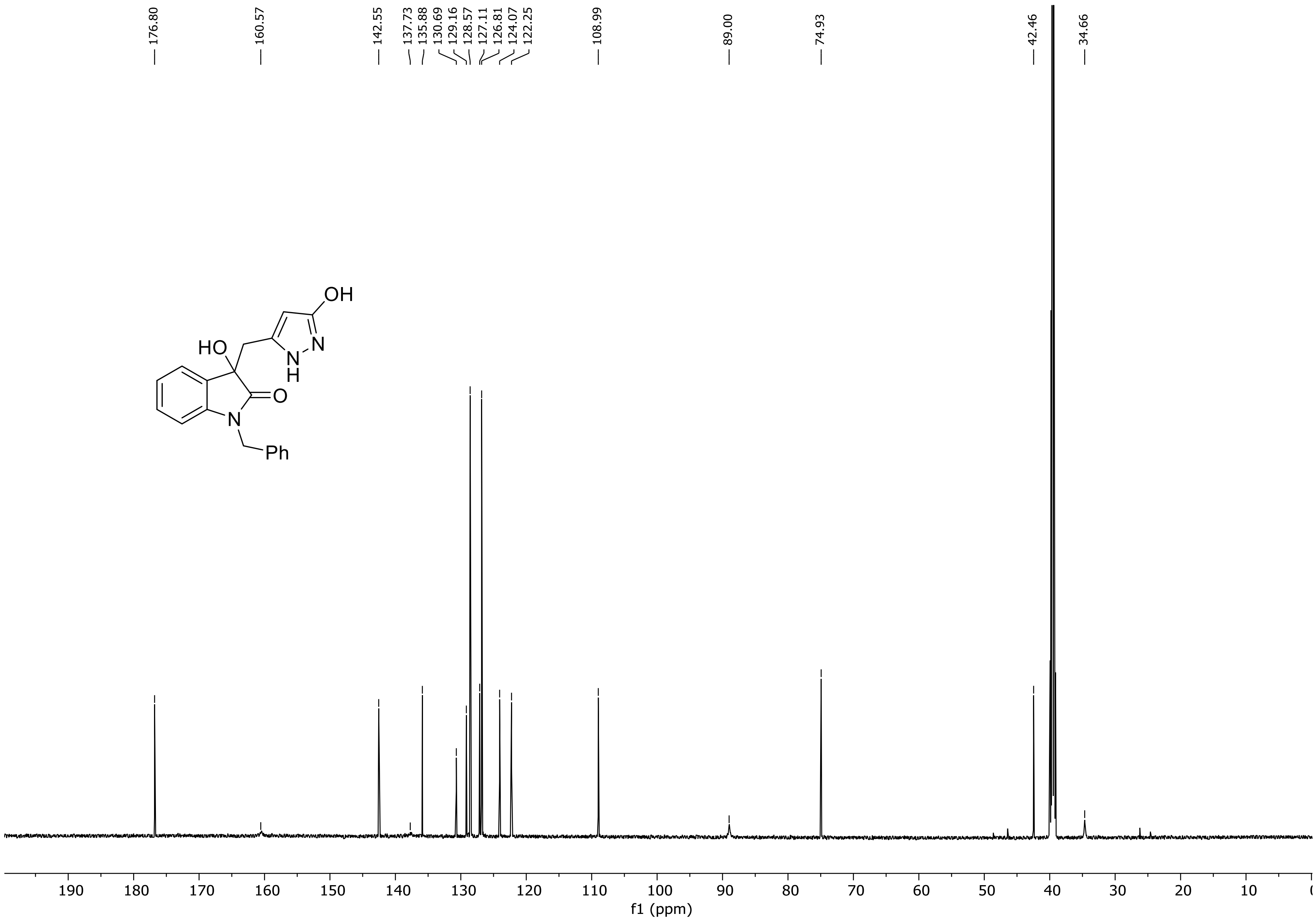
176.80
160.57
142.55
137.73
135.88
130.69
129.16
128.57
127.11
126.81
124.07
122.25
108.99
89.00
74.93
42.46
34.66
OH
HO
N
N
H
O
N
Ph
190 180 170 160 150 140 130 120 110 100 90 80 70 60 50 40 30 20 10
f1 (ppm)

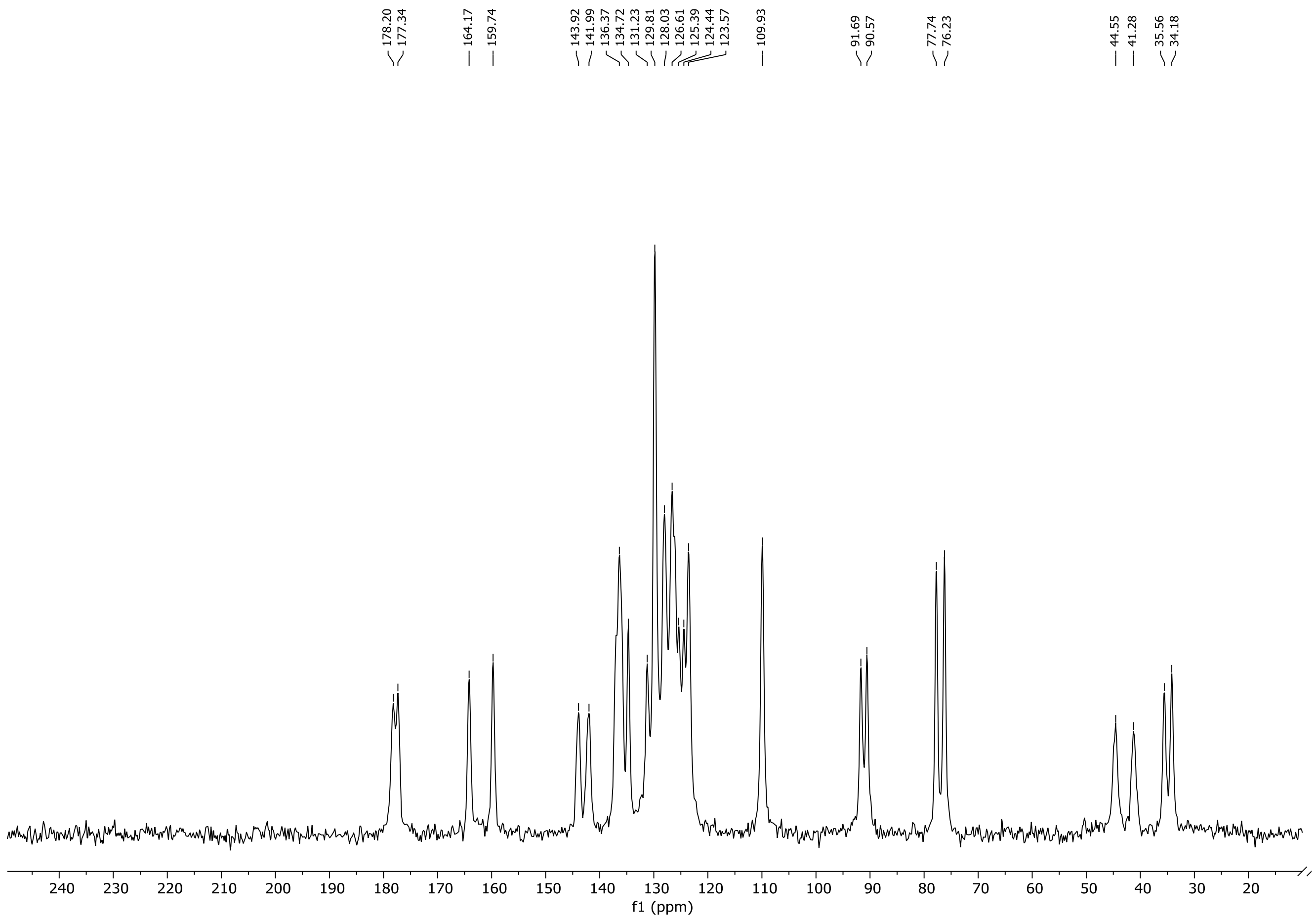

Solid-state carbon-13 NMR spectrum recorded with a 4 s recycle delay and 10 ms contact time.

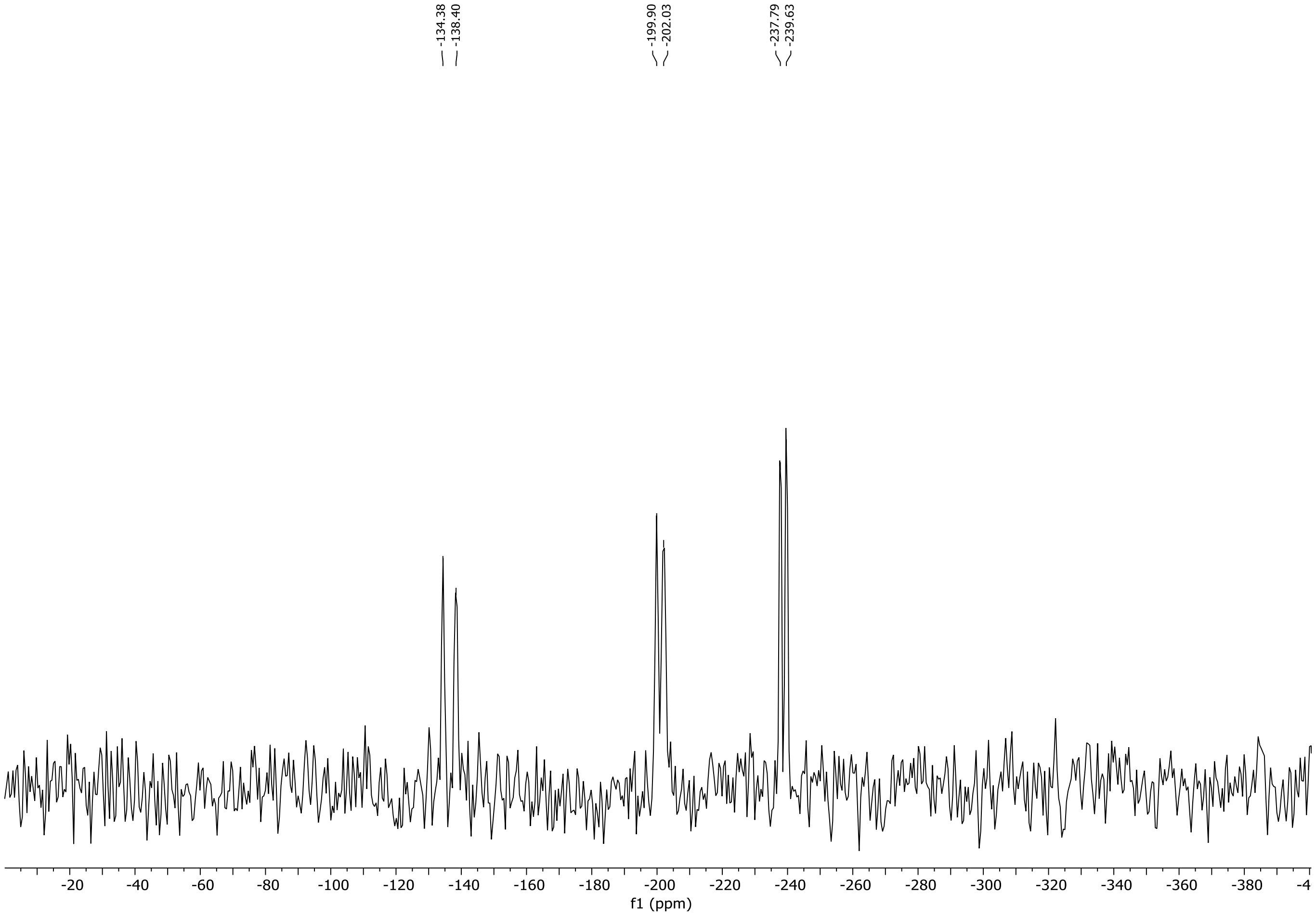

Solid-state nitrogen-15 NMR spectrum recorded with a 30 s recycle delay and 10 ms contact time.

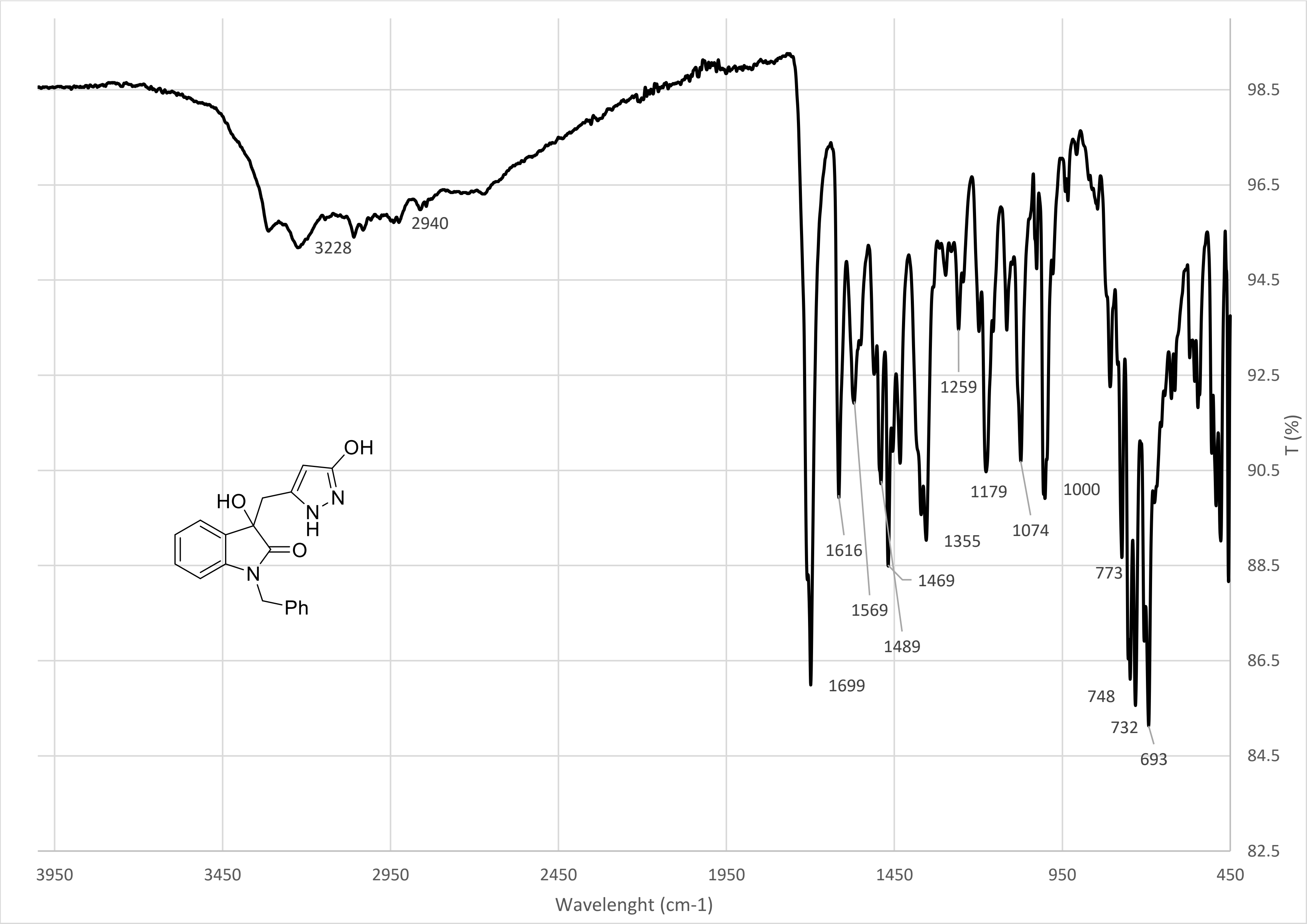
3228
2940
1699
1616
1569
1489
1469
1355
1259
1179
1074
1000
773
748
732
693
T (%)
Wavelenght (cm-1)
OH
HO
N
N
H
O
N
Ph

Elemental Composition Report

Single Mass Analysis

Tolerance = 3.0 mDa / DBE: min = -1.5, max = 100.0
Element prediction: Off
Number of isotope peaks used for i-FIT = 5

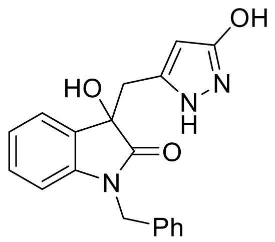

Monoisotopic Mass, Even Electron Ions
214 formula(e) evaluated with 1 results within limits (up to 500 closest results for each mass)
Elements Used:
C: 0-40 H: 0-80 N: 0-4 O: 0-4 I: 0-2

QToF Premier

22-Nov-2019
GG304 324 (2.735) Cm (323:332)

1: TOF MS ES+
2.21e+004

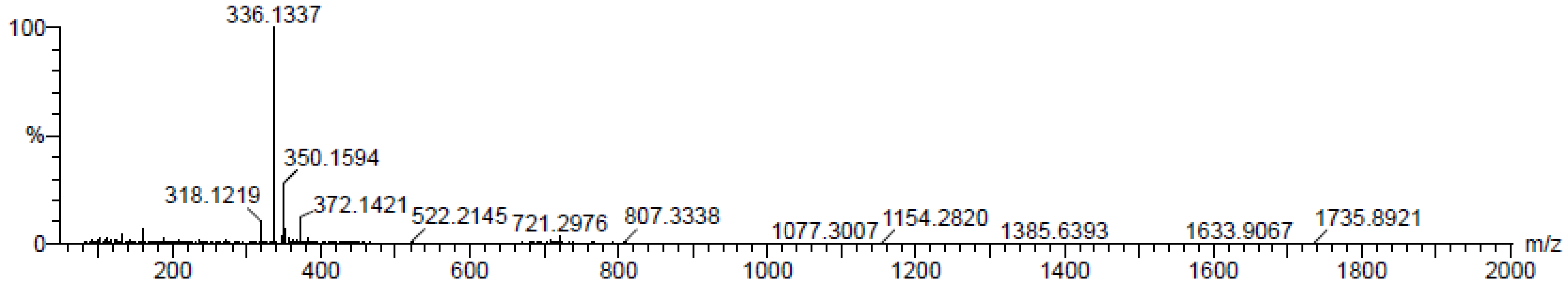

Minimum:				-1.5			
Maximum:		3.0	5.0	100.0			

Mass	Calc. Mass	mDa	PPM	DBE	i-FIT	i-FIT (Norm)	Formula
336.1337	336.1348	-1.1	-3.3	12.5	585.0	0.0	C19 H18 N3 O3

Table 1 Crystal data and structure refinement for 1986567.	
Identification code	1986567
Empirical formula	$C_{19}H_{17}N_3O_3$
Formula weight	335.35
Temperature/K	120
Crystal system	monoclinic
Space group	$P2_1/n$
a/Å	8.5554(6)
b/Å	29.118(2)
c/Å	13.1745(9)
α/°	90
β/°	92.172(3)
γ/°	90
Volume/Å^3	3279.6(4)
Z	8
ρ_{calc}g/cm^3	1.358
μ/mm^{-1}	0.094
F(000)	1408.0
Crystal size/mm^3	0.16 × 0.08 × 0.077
Radiation	MoKα (λ = 0.71073)
2Θ range for data collection/°	4.172 to 50.054
Index ranges	$-10 \le h \le 10$, $-34 \le k \le 34$, $-15 \le l \le 15$
Reflections collected	48703
Independent reflections	5792 [R_{int} = 0.0673, R_{sigma} = 0.0494]
Data/restraints/parameters	5792/0/484
Goodness-of-fit on F^2	1.016
Final R indexes [I>=2σ (I)]	R_1 = 0.0409, wR_2 = 0.0813
Final R indexes [all data]	R_1 = 0.0713, wR_2 = 0.0911
Largest diff. peak/hole / e Å^{-3}	0.26/-0.22

Table 2 Fractional Atomic Coordinates (×10⁴) and Equivalent Isotropic Displacement Parameters (Å²×10³) for 1986567. U_{eq} is defined as 1/3 of of the trace of the orthogonalised U_{IJ} tensor.				
**Atom**	***x***	***y***	***z***	**U(eq)**
O1	-285.6(15)	3896.9(4)	8593.8(10)	20.6(3)
O2	3010.6(15)	4258.5(4)	8893.9(10)	24.1(3)
O3	3056.9(18)	5371.9(5)	4949.8(10)	30.1(4)
N1	1527.0(17)	4914.5(5)	8892.6(11)	17.5(3)
N2	3341.8(18)	4392.2(6)	6368.4(12)	21.5(4)
N3	4023.4(18)	4722.3(5)	5797.2(11)	22.4(4)
C1	372(2)	4274.7(6)	8066.3(13)	17.7(4)
C2	-734(2)	4674.9(6)	8063.2(13)	17.6(4)
C3	-2240(2)	4715.6(7)	7669.8(14)	23.6(5)
C4	-3028(2)	5125.8(7)	7807.2(16)	28.6(5)
C5	-2312(2)	5484.5(7)	8331.8(16)	29.4(5)
C6	-774(2)	5451.1(6)	8714.0(15)	24.9(5)
C7	-13(2)	5040.9(6)	8567.4(13)	18.1(4)
C8	1810(2)	4468.9(6)	8665.3(13)	17.8(4)
C9	826(2)	4122.4(6)	7001.7(14)	20.5(4)
C10	1796(2)	4457.4(6)	6447.9(13)	18.9(4)
C11	1433(2)	4852.0(7)	5922.0(14)	21.8(4)
C12	2844(2)	4998.2(6)	5527.0(13)	21.4(4)
C13	2627(2)	5210.3(6)	9450.8(14)	22.2(4)
C14	3135(2)	5623.4(6)	8857.2(14)	19.2(4)
C15	3030(2)	5640.9(7)	7810.9(15)	25.9(5)
C16	3533(2)	6021.9(7)	7293.1(16)	32.1(5)
C17	4158(3)	6390.2(7)	7812.8(17)	36.3(5)
C18	4310(3)	6373.4(8)	8859.2(18)	45.4(6)
C19	3797(3)	5991.6(7)	9375.7(16)	36.7(6)
O1'	8315.3(15)	3126.0(5)	7680.0(11)	26.4(3)
O2'	5772.3(15)	3754.2(4)	6967.9(10)	26.6(3)
O3'	528.6(16)	2493.2(5)	8024.5(11)	28.9(4)
N1'	5353.9(18)	3099.1(5)	6018.4(12)	22.8(4)

N2'	3276.6(18)	3307.2(6)	8646.8(12)	23.1(4)
N3'	1754.4(18)	3184.2(5)	8460.8(12)	23.2(4)
C1'	6700(2)	3006.4(6)	7596.6(14)	20.5(4)
C2'	6485(2)	2554.4(6)	7068.4(14)	20.2(4)
C3'	6847(2)	2116.3(7)	7388.2(16)	27.0(5)
C4'	6380(3)	1747.3(7)	6775.6(16)	33.7(5)
C5'	5565(3)	1823.1(7)	5868.1(16)	36.4(6)
C6'	5185(2)	2264.8(7)	5540.0(15)	30.4(5)
C7'	5664(2)	2623.0(6)	6151.8(14)	22.3(4)
C8'	5877(2)	3339.1(6)	6842.4(15)	20.4(4)
C9'	5980(2)	3024.0(7)	8639.0(14)	22.9(5)
C10'	4255(2)	2952.3(6)	8562.0(14)	20.8(4)
C11'	3355(2)	2573.8(7)	8314.5(15)	22.3(4)
C12'	1835(2)	2738.9(6)	8257.9(14)	21.6(4)
C13'	4711(2)	3314.7(7)	5096.0(15)	29.4(5)
C14'	2947(2)	3357.0(7)	5028.0(15)	29.6(5)
C15'	1976(3)	3050.4(8)	5505.9(16)	33.9(5)
C16'	370(3)	3089.2(9)	5386.1(18)	44.0(6)
C17'	-284(3)	3433.3(10)	4795(2)	53.2(7)
C18'	662(3)	3742.4(9)	4322.2(19)	52.6(8)
C19'	2277(3)	3705.7(8)	4437.5(16)	39.4(6)

Table 3 Anisotropic Displacement Parameters (Å^2×10^3) for 1986567. The Anisotropic displacement factor exponent takes the form: -2π^2[$h^2a^{*2}U_{11}$+2hka*b*U_{12}+…].

Atom	U_{11}	U_{22}	U_{33}	U_{23}	U_{13}	U_{12}
O1	20.0(7)	14.4(7)	27.6(8)	0.1(6)	4.4(6)	0.0(6)
O2	21.6(7)	19.5(7)	30.9(8)	-0.9(6)	-2.6(6)	1.8(6)
O3	33.7(9)	29.4(8)	27.7(8)	12.7(6)	9.3(7)	9.1(7)
N1	20.2(8)	13.9(8)	18.2(8)	-2.3(6)	0.5(7)	-3.3(7)
N2	22.4(9)	19.8(9)	22.4(9)	5.0(7)	1.5(7)	3.3(8)

N3	25.3(9)	21.7(9)	20.4(8)	4.8(7)	3.5(7)	1.5(7)
C1	20.9(10)	13.1(9)	19.4(10)	-0.2(8)	3.5(8)	-2.0(8)
C2	18.9(10)	17.5(10)	16.6(10)	0.7(8)	3.6(8)	-0.6(8)
C3	23.4(11)	21.7(11)	25.8(11)	0.9(9)	2.5(9)	-1.5(9)
C4	22.5(11)	23.7(12)	39.5(12)	4.0(10)	0.6(9)	3.4(9)
C5	28.2(12)	16.5(11)	44.1(13)	4.9(9)	8.6(10)	7.3(9)
C6	29.8(12)	14.3(10)	31.2(12)	-1.7(9)	7.3(9)	-2.6(9)
C7	20.6(10)	15.9(10)	18.0(10)	1.0(8)	5.3(8)	-1.1(8)
C8	21.9(10)	16.9(10)	15.0(10)	1.5(8)	4.7(8)	-0.9(8)
C9	20.8(10)	18.6(10)	22.3(10)	-4.3(8)	1.0(8)	0.8(8)
C10	19.8(10)	21.8(11)	15.2(10)	-5.3(8)	1.0(8)	0.8(8)
C11	21.4(11)	26.2(12)	17.6(10)	-1.7(8)	-1.8(9)	8.2(9)
C12	26.9(11)	22.3(11)	15.0(10)	2.1(8)	1.4(8)	5.5(9)
C13	27.8(11)	20.0(11)	18.7(10)	-3.1(8)	-1.0(8)	-4.5(9)
C14	18.6(10)	16.2(10)	23.1(10)	-2.6(8)	2.2(8)	-0.4(8)
C15	27.6(11)	25.5(11)	24.7(11)	-2.4(9)	0.4(9)	-8.2(9)
C16	32.9(12)	38.0(13)	25.6(11)	8.5(10)	1.6(10)	-6.8(10)
C17	44.9(14)	22.9(12)	41.9(14)	9.2(10)	11.4(11)	-7.2(10)
C18	69.0(17)	26.8(13)	41.0(14)	-8.5(11)	11.3(12)	-22.1(12)
C19	57.7(15)	26.9(12)	25.9(12)	-5.6(10)	7.1(11)	-13.4(11)
O1'	16.9(7)	20.1(8)	42.0(9)	-2.2(7)	-2.2(6)	0.0(6)
O2'	23.9(7)	15.8(8)	39.8(9)	1.0(6)	-3.8(6)	0.8(6)
O3'	20.0(8)	18.5(8)	48.1(9)	-0.9(7)	-0.7(7)	0.8(6)
N1'	26.3(9)	19.3(9)	22.5(9)	2.1(7)	-2.2(7)	1.0(7)
N2'	21.5(9)	16.6(9)	31.2(10)	-2.2(8)	1.5(7)	0.0(7)
N3'	20.2(9)	17.6(9)	32.0(9)	1.5(7)	3.7(7)	0.4(7)
C1'	14.5(10)	17.7(10)	28.8(11)	-1.6(8)	-3.1(8)	-0.3(8)
C2'	17.2(10)	17.3(10)	26.2(11)	-1.8(8)	3.6(8)	0.5(8)
C3'	29.9(12)	21.8(11)	29.4(12)	-0.5(9)	1.2(9)	4.7(9)
C4'	46.6(14)	16.4(11)	38.2(13)	-3.2(10)	3.2(11)	4.9(10)
C5'	55.1(15)	23.3(12)	31.0(12)	-10.9(10)	2.7(11)	-0.2(11)
C6'	40.8(13)	28.8(12)	21.5(11)	-4.1(9)	-0.1(10)	1.5(10)

C7'	24.5(11)	18.5(10)	24.3(11)	-1.2(9)	4.7(9)	1.4(9)
C8'	13.9(10)	17.7(11)	29.9(11)	2.0(9)	4.3(8)	-0.6(8)
C9'	24.3(11)	18.0(10)	26.1(11)	-2.7(8)	-4.4(9)	1.1(8)
C10'	23.2(11)	20.0(10)	19.2(10)	-0.6(8)	1.3(8)	3.4(9)
C11'	22.3(11)	15.8(11)	28.9(11)	0.5(9)	2.5(9)	2.7(9)
C12'	21.7(11)	17.2(11)	26.1(11)	1.2(8)	2.7(9)	-3.1(8)
C13'	38.6(13)	26.8(12)	22.7(11)	2.8(9)	-1.2(9)	2.7(10)
C14'	38.2(13)	28.8(12)	21.1(11)	-8.9(9)	-6.8(9)	6.8(10)
C15'	37.4(13)	38.3(13)	25.7(12)	-9.0(10)	-2.4(10)	5.1(11)
C16'	34.0(14)	59.9(17)	37.7(14)	-19.7(13)	-4.2(11)	3.2(12)
C17'	39.3(15)	71(2)	47.5(16)	-30.7(15)	-15.4(13)	17.5(15)
C18'	62.2(18)	49.7(17)	43.6(15)	-18.8(13)	-28.3(14)	29.9(15)
C19'	55.0(16)	29.7(13)	32.4(13)	-7.7(10)	-13.1(11)	11.5(11)

Table 4 Bond Lengths for 1986567.

Atom	Atom	Length/Å		Atom	Atom	Length/Å
O1	C1	1.428(2)		O1'	C1'	1.426(2)
O2	C8	1.223(2)		O2'	C8'	1.224(2)
O3	C12	1.344(2)		O3'	C12'	1.352(2)
N1	C7	1.419(2)		N1'	C7'	1.421(2)
N1	C8	1.356(2)		N1'	C8'	1.353(2)
N1	C13	1.455(2)		N1'	C13'	1.457(2)
N2	N3	1.365(2)		N2'	N3'	1.364(2)
N2	C10	1.344(2)		N2'	C10'	1.337(2)
N3	C12	1.328(2)		N3'	C12'	1.326(2)
C1	C2	1.501(2)		C1'	C2'	1.497(3)
C1	C8	1.544(3)		C1'	C8'	1.539(3)
C1	C9	1.535(2)		C1'	C9'	1.527(3)
C2	C3	1.376(3)		C2'	C3'	1.375(3)
C2	C7	1.388(2)		C2'	C7'	1.388(3)

C3	C4	1.387(3)		C3'	C4'	1.393(3)
C4	C5	1.383(3)		C4'	C5'	1.379(3)
C5	C6	1.395(3)		C5'	C6'	1.391(3)
C6	C7	1.377(3)		C6'	C7'	1.371(3)
C9	C10	1.489(3)		C9'	C10'	1.490(3)
C10	C11	1.371(3)		C10'	C11'	1.377(3)
C11	C12	1.399(3)		C11'	C12'	1.386(3)
C13	C14	1.508(3)		C13'	C14'	1.513(3)
C14	C15	1.379(3)		C14'	C15'	1.387(3)
C14	C19	1.381(3)		C14'	C19'	1.390(3)
C15	C16	1.380(3)		C15'	C16'	1.382(3)
C16	C17	1.370(3)		C16'	C17'	1.375(4)
C17	C18	1.381(3)		C17'	C18'	1.375(4)
C18	C19	1.383(3)		C18'	C19'	1.389(3)

**Table 5 Bond Angles for 1986567.**								
**Atom**	**Atom**	**Atom**	**Angle/˚**		**Atom**	**Atom**	**Atom**	**Angle/˚**
C7	N1	C13	125.07(15)		C7'	N1'	C13'	125.89(16)
C8	N1	C7	110.71(14)		C8'	N1'	C7'	110.50(15)
C8	N1	C13	124.11(15)		C8'	N1'	C13'	123.35(16)
C10	N2	N3	112.68(16)		C10'	N2'	N3'	112.25(16)
C12	N3	N2	103.73(15)		C12'	N3'	N2'	103.68(15)
O1	C1	C2	109.85(14)		O1'	C1'	C2'	110.61(15)
O1	C1	C8	110.75(14)		O1'	C1'	C8'	108.32(15)
O1	C1	C9	109.88(14)		O1'	C1'	C9'	110.21(15)
C2	C1	C8	101.97(14)		C2'	C1'	C8'	102.03(15)
C2	C1	C9	113.78(15)		C2'	C1'	C9'	113.65(15)
C9	C1	C8	110.39(15)		C9'	C1'	C8'	111.66(15)
C3	C2	C1	130.49(17)		C3'	C2'	C1'	130.62(18)
C3	C2	C7	120.47(17)		C3'	C2'	C7'	120.04(17)

C7	C2	C1	109.04(15)		C7'	C2'	C1'	109.09(16)
C2	C3	C4	118.55(19)		C2'	C3'	C4'	118.78(19)
C5	C4	C3	120.51(19)		C5'	C4'	C3'	120.23(19)
C4	C5	C6	121.46(18)		C4'	C5'	C6'	121.46(19)
C7	C6	C5	117.01(18)		C7'	C6'	C5'	117.34(19)
C2	C7	N1	109.63(15)		C2'	C7'	N1'	109.50(16)
C6	C7	N1	128.39(17)		C6'	C7'	N1'	128.24(18)
C6	C7	C2	121.96(17)		C6'	C7'	C2'	122.14(18)
O2	C8	N1	125.48(17)		O2'	C8'	N1'	126.52(18)
O2	C8	C1	126.00(16)		O2'	C8'	C1'	124.65(17)
N1	C8	C1	108.52(15)		N1'	C8'	C1'	108.79(15)
C10	C9	C1	115.05(15)		C10'	C9'	C1'	111.49(15)
N2	C10	C9	120.92(16)		N2'	C10'	C9'	120.49(17)
N2	C10	C11	106.49(17)		N2'	C10'	C11'	106.97(17)
C11	C10	C9	132.56(17)		C11'	C10'	C9'	132.24(18)
C10	C11	C12	105.11(17)		C10'	C11'	C12'	104.52(17)
O3	C12	C11	126.48(17)		O3'	C12'	C11'	126.47(17)
N3	C12	O3	121.55(17)		N3'	C12'	O3'	120.95(16)
N3	C12	C11	111.97(16)		N3'	C12'	C11'	112.58(17)
N1	C13	C14	113.83(15)		N1'	C13'	C14'	115.38(17)
C15	C14	C13	122.60(17)		C15'	C14'	C13'	122.26(19)
C15	C14	C19	118.41(18)		C15'	C14'	C19'	118.9(2)
C19	C14	C13	118.93(17)		C19'	C14'	C13'	118.8(2)
C14	C15	C16	120.94(19)		C16'	C15'	C14'	120.4(2)
C17	C16	C15	120.4(2)		C17'	C16'	C15'	120.4(3)
C16	C17	C18	119.4(2)		C16'	C17'	C18'	120.0(2)
C17	C18	C19	120.0(2)		C17'	C18'	C19'	120.0(2)
C14	C19	C18	120.8(2)		C18'	C19'	C14'	120.3(2)

Table 6 Hydrogen Bonds for 1986567.						
**D**	**H**	**A**	**d(D-H)/Å**	**d(H-A)/Å**	**d(D-A)/Å**	**D-H-A/°**
O1	H1	N3'	0.91(2)	1.81(2)	2.722(2)	175(2)
O3	H3	N3[1]	0.93(3)	1.80(3)	2.733(2)	176(2)
N2	H2	O2'	0.89(2)	2.02(2)	2.877(2)	161.7(18)
O1'	H1'	O1[2]	0.88(3)	1.97(3)	2.7950(19)	154(2)
O3'	H3'	O1'[3]	0.90(3)	1.77(3)	2.6687(19)	171(2)
N2'	H2'	O2	0.91(2)	1.94(2)	2.799(2)	156(2)

[1]1-X,1-Y,1-Z; [2]1+X,+Y,+Z; [3]-1+X,+Y,+Z

Table 7 Torsion Angles for 1986567.										
**A**	**B**	**C**	**D**	**Angle/˚**		**A**	**B**	**C**	**D**	**Angle/˚**
O1	C1	C2	C3	-60.6(2)		O1'	C1'	C2'	C3'	70.9(3)
O1	C1	C2	C7	118.48(16)		O1'	C1'	C2'	C7'	-115.02(17)
O1	C1	C8	O2	60.6(2)		O1'	C1'	C8'	O2'	-63.0(2)
O1	C1	C8	N1	-119.78(15)		O1'	C1'	C8'	N1'	114.81(16)
O1	C1	C9	C10	-168.89(15)		O1'	C1'	C9'	C10'	173.61(15)
N1	C13	C14	C15	-21.4(3)		N1'	C13'	C14'	C15'	-28.5(3)
N1	C13	C14	C19	161.60(18)		N1'	C13'	C14'	C19'	153.73(18)
N2	N3	C12	O3	179.83(16)		N2'	N3'	C12'	O3'	-179.51(16)
N2	N3	C12	C11	0.5(2)		N2'	N3'	C12'	C11'	0.4(2)
N2	C10	C11	C12	1.3(2)		N2'	C10'	C11'	C12'	0.5(2)
N3	N2	C10	C9	177.22(15)		N3'	N2'	C10'	C9'	174.15(16)
N3	N2	C10	C11	-1.0(2)		N3'	N2'	C10'	C11'	-0.4(2)
C1	C2	C3	C4	177.65(18)		C1'	C2'	C3'	C4'	173.75(19)
C1	C2	C7	N1	1.2(2)		C1'	C2'	C7'	N1'	1.8(2)
C1	C2	C7	C6	-177.71(16)		C1'	C2'	C7'	C6'	-174.65(18)
C1	C9	C10	N2	103.3(2)		C1'	C9'	C10'	N2'	-103.7(2)
C1	C9	C10	C11	-79.0(3)		C1'	C9'	C10'	C11'	69.2(3)

C2	C1	C8	O2	177.43(17)		C2'	C1'	C8'	O2'	-179.70(17)
C2	C1	C8	N1	-2.94(18)		C2'	C1'	C8'	N1'	-1.92(19)
C2	C1	C9	C10	67.5(2)		C2'	C1'	C9'	C10'	-61.6(2)
C2	C3	C4	C5	-0.2(3)		C2'	C3'	C4'	C5'	-0.2(3)
C3	C2	C7	N1	-179.62(16)		C3'	C2'	C7'	N1'	176.59(17)
C3	C2	C7	C6	1.5(3)		C3'	C2'	C7'	C6'	0.2(3)
C3	C4	C5	C6	1.7(3)		C3'	C4'	C5'	C6'	-0.1(3)
C4	C5	C6	C7	-1.6(3)		C4'	C5'	C6'	C7'	0.5(3)
C5	C6	C7	N1	-178.69(17)		C5'	C6'	C7'	N1'	-176.22(19)
C5	C6	C7	C2	0.0(3)		C5'	C6'	C7'	C2'	-0.5(3)
C7	N1	C8	O2	-176.51(17)		C7'	N1'	C8'	O2'	-179.16(18)
C7	N1	C8	C1	3.86(19)		C7'	N1'	C8'	C1'	3.1(2)
C7	N1	C13	C14	-65.3(2)		C7'	N1'	C13'	C14'	91.3(2)
C7	C2	C3	C4	-1.3(3)		C7'	C2'	C3'	C4'	0.2(3)
C8	N1	C7	C2	-3.3(2)		C8'	N1'	C7'	C2'	-3.1(2)
C8	N1	C7	C6	175.54(18)		C8'	N1'	C7'	C6'	173.0(2)
C8	N1	C13	C14	118.81(18)		C8'	N1'	C13'	C14'	-95.2(2)
C8	C1	C2	C3	-178.08(18)		C8'	C1'	C2'	C3'	-174.05(19)
C8	C1	C2	C7	0.99(18)		C8'	C1'	C2'	C7'	0.03(19)
C8	C1	C9	C10	-46.5(2)		C8'	C1'	C9'	C10'	53.2(2)
C9	C1	C2	C3	63.1(3)		C9'	C1'	C2'	C3'	-53.7(3)
C9	C1	C2	C7	-117.87(17)		C9'	C1'	C2'	C7'	120.39(17)
C9	C1	C8	O2	-61.3(2)		C9'	C1'	C8'	O2'	58.6(2)
C9	C1	C8	N1	118.30(16)		C9'	C1'	C8'	N1'	-123.65(16)
C9	C10	C11	C12	-176.72(19)		C9'	C10'	C11'	C12'	-173.06(19)
C10	N2	N3	C12	0.3(2)		C10'	N2'	N3'	C12'	0.0(2)
C10	C11	C12	O3	179.59(17)		C10'	C11'	C12'	O3'	179.28(18)
C10	C11	C12	N3	-1.1(2)		C10'	C11'	C12'	N3'	-0.6(2)
C13	N1	C7	C2	-179.65(16)		C13'	N1'	C7'	C2'	171.06(17)
C13	N1	C7	C6	-0.8(3)		C13'	N1'	C7'	C6'	-12.8(3)
C13	N1	C8	O2	-0.1(3)		C13'	N1'	C8'	O2'	6.5(3)
C13	N1	C8	C1	-179.73(15)		C13'	N1'	C8'	C1'	-171.27(16)

C13	C14	C15	C16	-178.85(18)		C13'	C14'	C15'	C16'	-177.03(19)
C13	C14	C19	C18	178.7(2)		C13'	C14'	C19'	C18'	177.18(19)
C14	C15	C16	C17	0.4(3)		C14'	C15'	C16'	C17'	-0.3(3)
C15	C14	C19	C18	1.5(3)		C15'	C14'	C19'	C18'	-0.6(3)
C15	C16	C17	C18	1.3(3)		C15'	C16'	C17'	C18'	-0.2(3)
C16	C17	C18	C19	-1.6(4)		C16'	C17'	C18'	C19'	0.3(4)
C17	C18	C19	C14	0.2(4)		C17'	C18'	C19'	C14'	0.1(3)
C19	C14	C15	C16	-1.8(3)		C19'	C14'	C15'	C16'	0.7(3)

Table 8 Hydrogen Atom Coordinates (Å×10⁴) and Isotropic Displacement Parameters (Å²×10³) for 1986567.

Atom	*x*	*y*	*z*	U(eq)
H1	440(30)	3667(8)	8578(17)	48(7)
H3	4050(30)	5354(9)	4690(20)	70(9)
H2	3930(20)	4163(7)	6627(15)	29(6)
H3A	-2719.85	4473.25	7319.37	28
H4	-4045.69	5159.86	7544.38	34
H5	-2869.08	5753.96	8432.11	35
H6	-283.53	5694.97	9052.62	30
H9A	1396.33	3835.18	7062.72	25
H9B	-123.53	4063.39	6597.16	25
H11	470(20)	4992(7)	5858(14)	24(5)
H13A	3544.84	5031.9	9651.5	27
H13B	2148.11	5314.76	10065.42	27
H15	2614.66	5392.23	7449.11	31
H16	3447.21	6028.84	6587.4	39
H17	4477.69	6649.31	7463.82	44
H18	4756.12	6618.77	9216.94	54
H19	3900.23	5982.79	10080.71	44
H1'	8460(30)	3404(9)	7930(20)	66(9)
H3'	-290(30)	2683(9)	7913(18)	58(8)

H2'	3480(30)	3611(8)	8762(16)	43(7)
H3'A	7394.09	2067.17	8002.2	32
H4'	6618.35	1448.83	6979.45	40
H5'	5262.46	1573.43	5466.49	44
H6'	4628.06	2315.05	4930.08	36
H9'A	6452.62	2788.62	9071.57	28
H9'B	6201.63	3319.93	8950.4	28
H11'	3700(20)	2278(7)	8215(15)	34(6)
H13C	5041.23	3138.61	4516.99	35
H13D	5157.73	3619.52	5043.56	35
H15'	2408.79	2817.4	5909.36	41
H16'	-272.93	2881.38	5707.17	53
H17'	-1365.58	3457.03	4715.09	64
H18'	219.23	3976.02	3924.99	63
H19'	2914.19	3915.61	4117.86	47

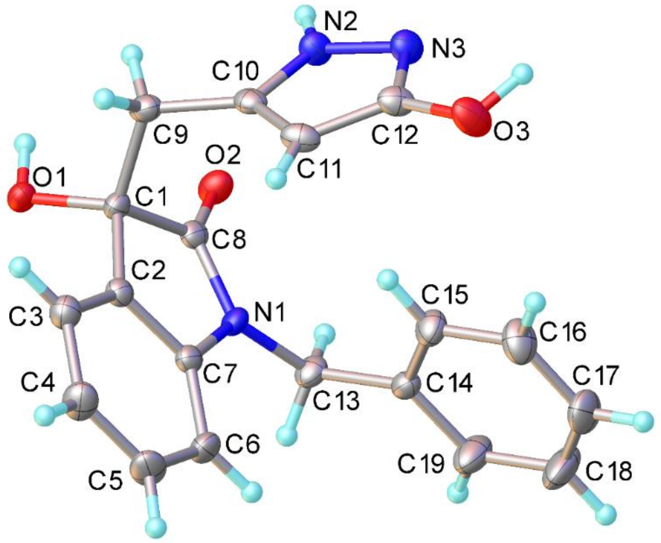

*1.14 Data of 2-(2-hydrazinyl-2-oxoethyl)quinoline-4-carbohydrazide (**16**)*

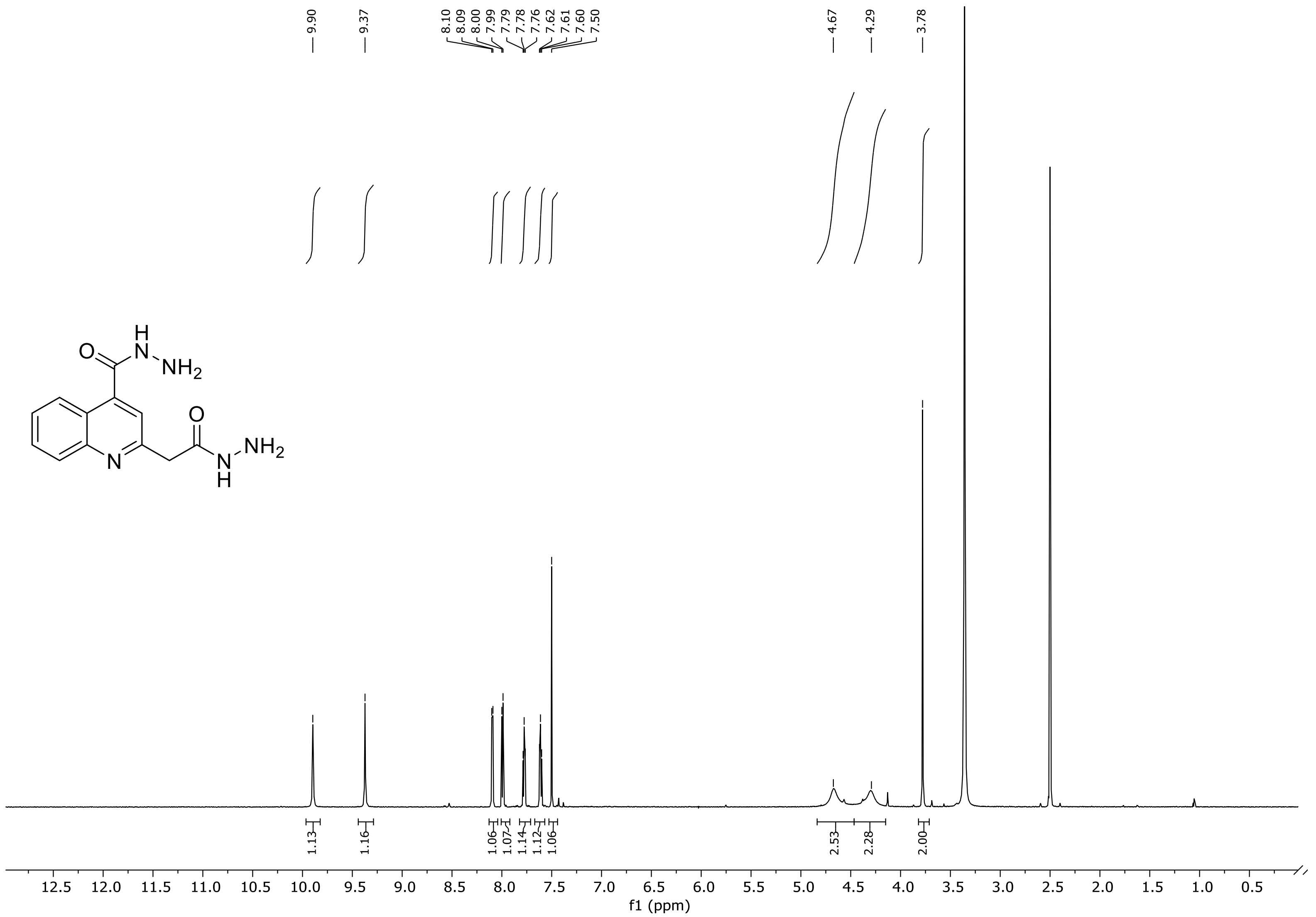

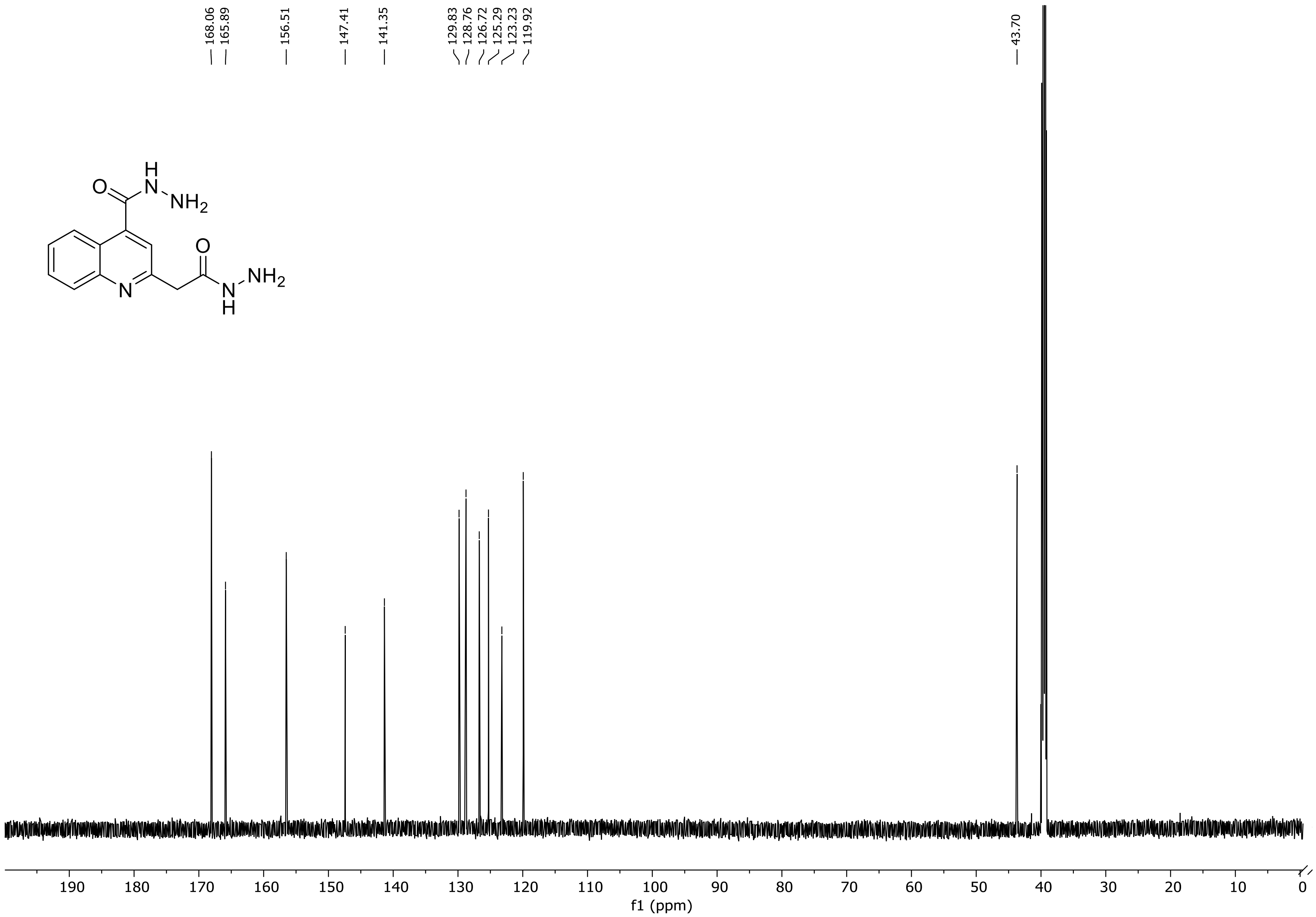

168.06
165.89
156.51
147.41
141.35
129.83
128.76
126.72
125.29
123.23
119.92
43.70
190 180 170 160 150 140 130 120 110 100 90 80 70 60 50 40 30 20 10 0
f1 (ppm)
O
H
N
NH2
N

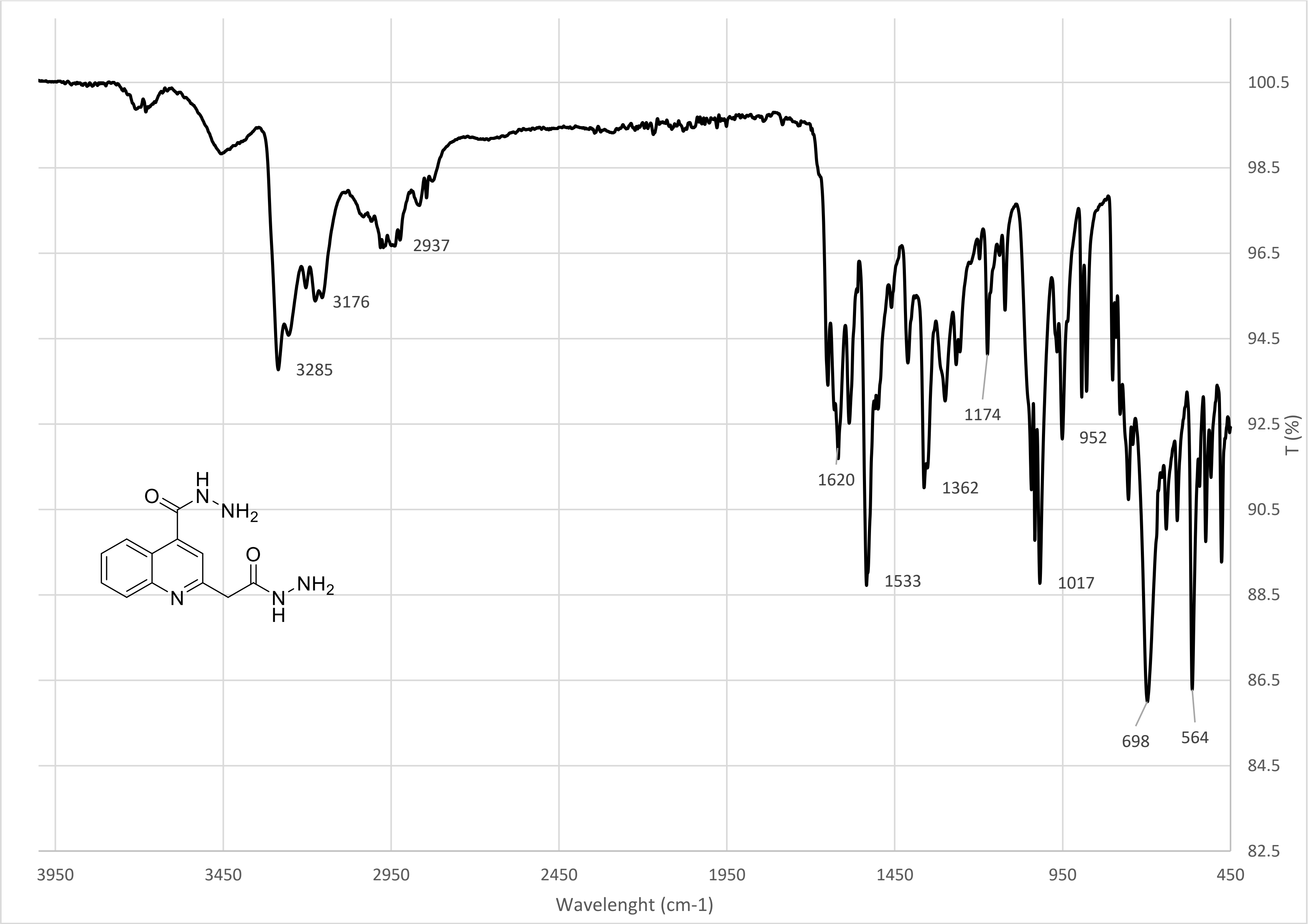
3285
3176
2937
1620
1533
1362
1174
1017
952
698
564
100.5
98.5
96.5
94.5
92.5
90.5
88.5
86.5
84.5
82.5
T (%)
3950
3450
2950
2450
1950
1450
950
450
Wavelenght (cm-1)

Elemental Composition Report

Single Mass Analysis

Tolerance = 3.0 mDa / DBE: min = -1.5, max = 50.0
Element prediction: Off
Number of isotope peaks used for i-FIT = 3

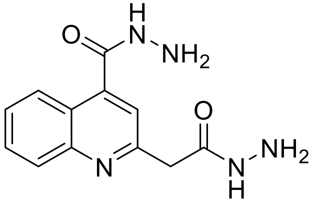

Monoisotopic Mass, Even Electron Ions
190 formula(e) evaluated with 2 results within limits (up to 500 closest results for each mass)
Elements Used:
C: 0-70 H: 0-100 N: 0-6 O: 0-8

01-Aug-2017
GGI27S 61 (0.524) Cm (61:62)

1: TOF MS ES+
1.73e+003

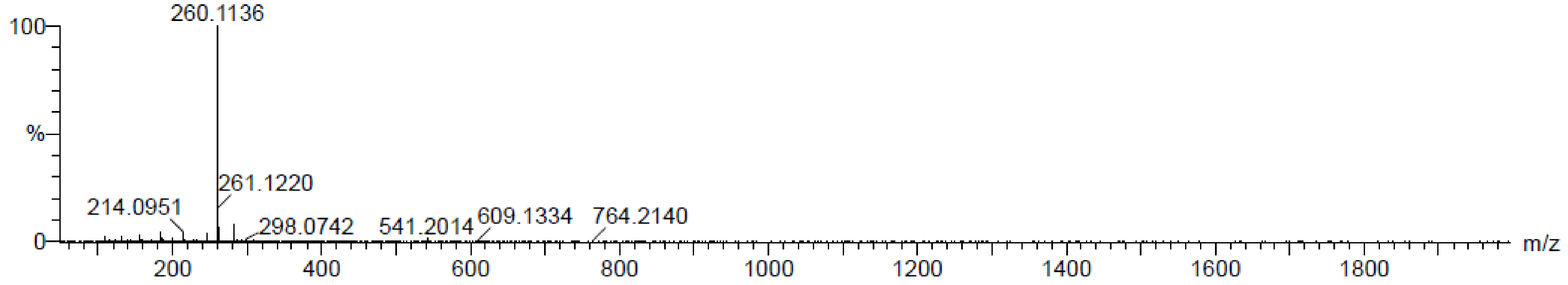

Minimum:				-1.5			
Maximum:		3.0	5.0	50.0			

Mass	Calc. Mass	mDa	PPM	DBE	i-FIT	i-FIT (Norm)	Formula
260.1136	260.1134	0.2	0.8	3.5	170.6	0.6	C11 H18 N O6
	260.1147	-1.1	-4.2	8.5	170.9	0.8	C12 H14 N5 O2

Table 1 Crystal data and structure refinement for 1986566.	
Identification code	1986566
Empirical formula	$C_{12}H_{15}N_5O_3$
Formula weight	277.29
Temperature/K	120
Crystal system	monoclinic
Space group	$P2_1/n$
a/Å	4.9407(5)
b/Å	14.0587(13)
c/Å	18.2746(16)
α/°	90
β/°	97.040(5)
γ/°	90
Volume/Å^3	1259.8(2)
Z	4
ρ_{calc}g/cm^3	1.462
μ/mm^{-1}	0.910
F(000)	584.0
Crystal size/mm^3	0.286 × 0.023 × 0.022
Radiation	CuKα (λ = 1.54184)
2Θ range for data collection/°	7.956 to 116.974
Index ranges	-5 ≤ h ≤ 5, -14 ≤ k ≤ 14, -19 ≤ l ≤ 20
Reflections collected	7750
Independent reflections	1719 [R_{int} = 0.0857, R_{sigma} = 0.0802]
Data/restraints/parameters	1719/0/214
Goodness-of-fit on F^2	1.041
Final R indexes [I>=2σ (I)]	R_1 = 0.0494, wR_2 = 0.0932
Final R indexes [all data]	R_1 = 0.0881, wR_2 = 0.1061

Largest diff. peak/hole / e Å^{-3}	0.20/-0.24

Table 2 Fractional Atomic Coordinates (×10^4) and Equivalent Isotropic Displacement Parameters (Å^2×10^3) for 1986566 . U_{eq} is defined as 1/3 of of the trace of the orthogonalised U_{IJ} tensor.

Atom	*x*	*y*	*z*	U(eq)
O(1)	6342(4)	4872.4(14)	8897(1)	26.2(6)
O(2)	1522(4)	4226.1(15)	5747.6(11)	31.0(6)
N(1)	2276(5)	6511.4(18)	6540.0(13)	23.6(7)
N(2)	1836(6)	4783.9(18)	8942.1(14)	23.1(7)
N(3)	2099(6)	4217(2)	9588.0(15)	25.4(7)
N(4)	-3012(6)	4038.4(19)	5483.3(14)	23.6(7)
N(5)	-2865(6)	3141(2)	5132.2(18)	26.8(7)
C(1)	4336(6)	6837(2)	7061.7(16)	21.1(8)
C(2)	5866(6)	7619(2)	6869.2(17)	26.3(8)
C(3)	7938(6)	7979(2)	7357.7(17)	28.8(9)
C(4)	8518(6)	7571(2)	8061.9(17)	27.2(9)
C(5)	7071(6)	6815(2)	8264.8(16)	22.4(8)
C(6)	4949(6)	6406(2)	7766.1(15)	19.6(8)
C(7)	3368(6)	5603(2)	7917.4(15)	19.5(8)
C(8)	1345(6)	5298(2)	7392.8(16)	22.7(8)
C(9)	841(6)	5780(2)	6708.9(16)	21.2(8)
C(10)	3967(6)	5071(2)	8627.3(16)	21.0(8)
C(11)	-1443(6)	5441(2)	6140.0(16)	25.6(8)
C(12)	-818(7)	4515(2)	5781.1(15)	22.5(8)
O(3)	-7623(5)	1867(2)	5092.9(13)	34.6(7)

Table 3 Anisotropic Displacement Parameters (Å^2×10^3) for 1986566. The Anisotropic displacement factor exponent takes the form: -2π^2[h^{2}a*^{2}U$_{11}$+2hka*b*U$_{12}$+…].						
**Atom**	**U_{11}**	**U_{22}**	**U_{33}**	**U_{23}**	**U_{13}**	**U_{12}**
O(1)	16.9(13)	37.0(14)	23.2(12)	6.9(10)	-4(1)	1.5(10)
O(2)	18.1(13)	40.2(15)	33.5(14)	-9.1(11)	-1.8(10)	2.6(11)
N(1)	24.9(16)	25.9(17)	18.3(15)	-3.6(13)	-4.4(12)	2.4(13)
N(2)	18.8(16)	31.6(18)	17.3(15)	5.4(13)	-3.6(14)	-0.4(13)
N(3)	25.8(17)	31.5(18)	17.8(17)	5.6(14)	-2.1(13)	0.0(15)
N(4)	21.2(18)	27.4(19)	21.0(16)	-3.7(13)	-2.8(13)	1.3(14)
N(5)	31.2(19)	28.7(19)	19.7(18)	-4.6(15)	-0.5(15)	0.6(15)
C(1)	21.8(18)	19.5(19)	21.7(18)	-2.0(15)	1.9(15)	1.1(15)
C(2)	31(2)	27(2)	20.3(19)	0.9(15)	0.8(16)	2.8(17)
C(3)	32(2)	25(2)	30(2)	1.9(16)	4.8(17)	-2.5(16)
C(4)	22.5(19)	27(2)	31(2)	-7.1(16)	-3.4(16)	0.4(16)
C(5)	24.4(18)	22(2)	19.4(18)	-1.9(15)	-2.2(15)	1.1(16)
C(6)	21.1(18)	20.1(19)	17.5(18)	1.3(14)	1.0(15)	2.7(15)
C(7)	14.6(17)	26(2)	17.5(18)	-2.4(14)	1.6(14)	3.1(15)
C(8)	18.8(18)	25(2)	24.5(19)	-0.3(16)	2.5(15)	-2.4(15)
C(9)	22.0(18)	23(2)	17.8(18)	-2.6(15)	-1.5(14)	5.7(16)
C(10)	21(2)	22.7(19)	18.0(18)	-2.9(15)	-2.9(16)	-2.4(15)
C(11)	23.5(19)	31(2)	20.9(19)	-0.2(15)	-4.1(15)	2.0(16)
C(12)	23(2)	30(2)	12.5(18)	1.1(15)	-3.6(15)	1.3(17)
O(3)	24.6(14)	34.5(16)	44.2(16)	-7.6(12)	2.6(12)	0.0(14)

Table 4 Bond Lengths for 1986566.						
**Atom**	**Atom**	**Length/Å**		**Atom**	**Atom**	**Length/Å**
O(1)	C(10)	1.247(3)		C(2)	C(3)	1.370(4)
O(2)	C(12)	1.234(3)		C(3)	C(4)	1.406(4)
N(1)	C(1)	1.385(4)		C(4)	C(5)	1.357(4)
N(1)	C(9)	1.308(4)		C(5)	C(6)	1.423(4)
N(2)	N(3)	1.417(3)		C(6)	C(7)	1.420(4)
N(2)	C(10)	1.323(4)		C(7)	C(8)	1.366(4)
N(4)	N(5)	1.422(4)		C(7)	C(10)	1.495(4)
N(4)	C(12)	1.332(4)		C(8)	C(9)	1.416(4)
C(1)	C(2)	1.403(4)		C(9)	C(11)	1.515(4)
C(1)	C(6)	1.421(4)		C(11)	C(12)	1.507(4)

Table 5 Bond Angles for 1986566.								
**Atom**	**Atom**	**Atom**	**Angle/˚**		**Atom**	**Atom**	**Atom**	**Angle/˚**
C(9)	N(1)	C(1)	118.2(3)		C(8)	C(7)	C(6)	119.0(3)
C(10)	N(2)	N(3)	122.5(3)		C(8)	C(7)	C(10)	120.1(3)
C(12)	N(4)	N(5)	123.1(3)		C(7)	C(8)	C(9)	120.3(3)
N(1)	C(1)	C(2)	117.4(3)		N(1)	C(9)	C(8)	122.8(3)
N(1)	C(1)	C(6)	122.7(3)		N(1)	C(9)	C(11)	117.7(3)
C(2)	C(1)	C(6)	120.0(3)		C(8)	C(9)	C(11)	119.4(3)
C(3)	C(2)	C(1)	120.5(3)		O(1)	C(10)	N(2)	121.4(3)
C(2)	C(3)	C(4)	119.9(3)		O(1)	C(10)	C(7)	122.1(3)
C(5)	C(4)	C(3)	120.9(3)		N(2)	C(10)	C(7)	116.5(3)
C(4)	C(5)	C(6)	120.8(3)		C(12)	C(11)	C(9)	113.3(2)
C(1)	C(6)	C(5)	117.8(3)		O(2)	C(12)	N(4)	122.4(3)
C(7)	C(6)	C(1)	117.0(3)		O(2)	C(12)	C(11)	123.3(3)

C(7)	C(6)	C(5)	125.2(3)		N(4)	C(12)	C(11)	114.3(3)
C(6)	C(7)	C(10)	120.8(3)					

**Table 6 Hydrogen Bonds for 1986566.**						
**D**	**H**	**A**	**d(D-H)/Å**	**d(H-A)/Å**	**d(D-A)/Å**	**D-H-A/°**
N(2)	H(2)	O(1)[1]	0.92(3)	1.91(4)	2.708(3)	144(3)
N(3)	H(3A)	O(1)[2]	0.95(3)	2.16(4)	3.062(4)	157(3)
N(3)	H(3B)	O(3)[3]	1.03(4)	2.15(4)	3.063(4)	147(3)
N(4)	H(4)	O(2)[1]	0.86(4)	1.97(4)	2.813(4)	166(4)
N(5)	H(5A)	N(1)[4]	0.87(3)	2.29(4)	3.143(4)	168(3)
N(5)	H(5B)	O(3)[5]	0.91(4)	2.38(4)	3.157(4)	144(3)
O(3)	H(3C)	N(3)[6]	0.93(5)	2.14(5)	3.023(4)	159(4)
O(3)	H(3D)	N(5)	0.92(5)	2.05(5)	2.949(4)	164(4)

[1]-1+X,+Y,+Z; [2]1-X,1-Y,2-Z; [3]3/2+X,1/2-Y,1/2+Z; [4]-X,1-Y,1-Z; [5]1+X,+Y,+Z; [6]-1/2+X,1/2-Y,-1/2+Z

**Table 7 Hydrogen Atom Coordinates (Å×10[4]) and Isotropic Displacement Parameters (Å[2]×10[3]) for 1986566.**				
**Atom**	***x***	***y***	***z***	**U(eq)**
H(2)	80(70)	4890(20)	8729(18)	42(10)
H(3A)	2610(60)	4650(20)	9978(19)	41(11)
H(3B)	3690(80)	3750(30)	9551(18)	57(12)
H(4)	-4620(80)	4190(30)	5570(20)	57(13)
H(5A)	-2450(60)	3240(20)	4691(19)	39(11)
H(5B)	-1380(80)	2820(30)	5346(19)	60(13)
H(2A)	5461.02	7901.5	6396.12	32

H(3)	8981.15	8502.79	7220.79	35
H(4A)	9943.06	7828.08	8400.57	33
H(5)	7480.09	6555.99	8745.75	27
H(8)	271.83	4761.81	7487.23	27
H(11A)	-3120.08	5362.55	6380.1	31
H(11B)	-1808.03	5934.03	5753.83	31
H(3C)	-6530(90)	1430(30)	4880(20)	88(17)
H(3D)	-6370(90)	2360(30)	5130(20)	83(16)

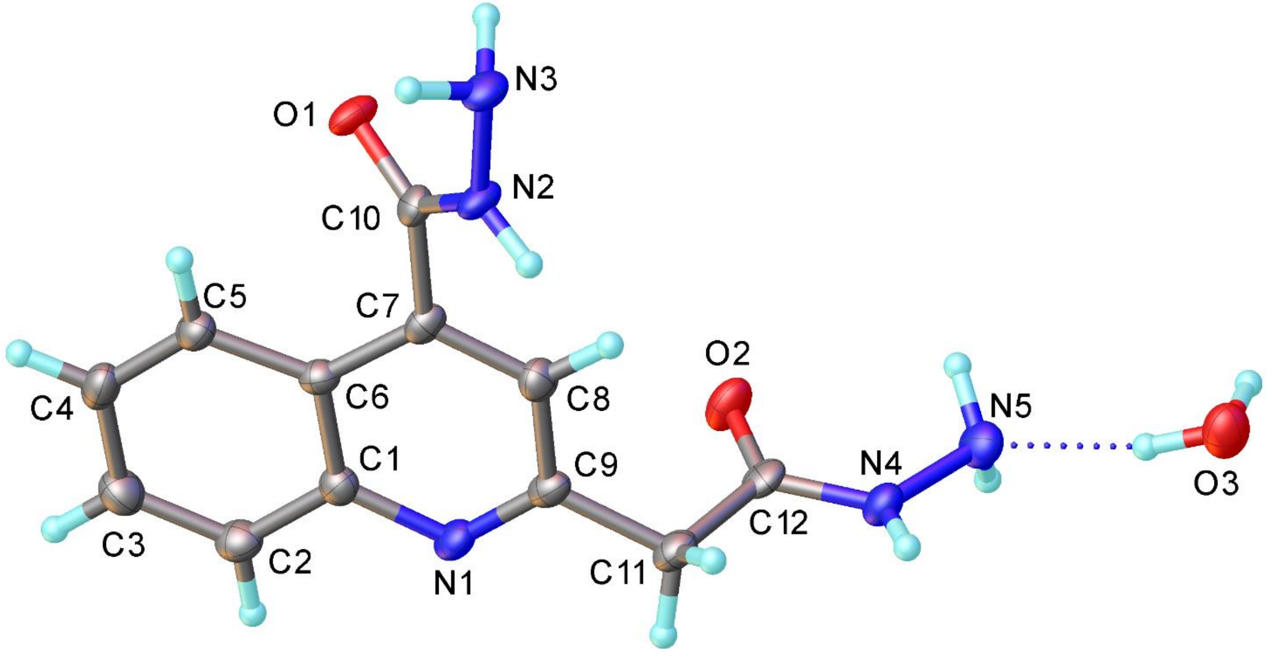

*1.15 Data of 6-chloro-2-(2-hydrazinyl-2-oxoethyl)quinoline-4-carbohydrazide (**16d**)*

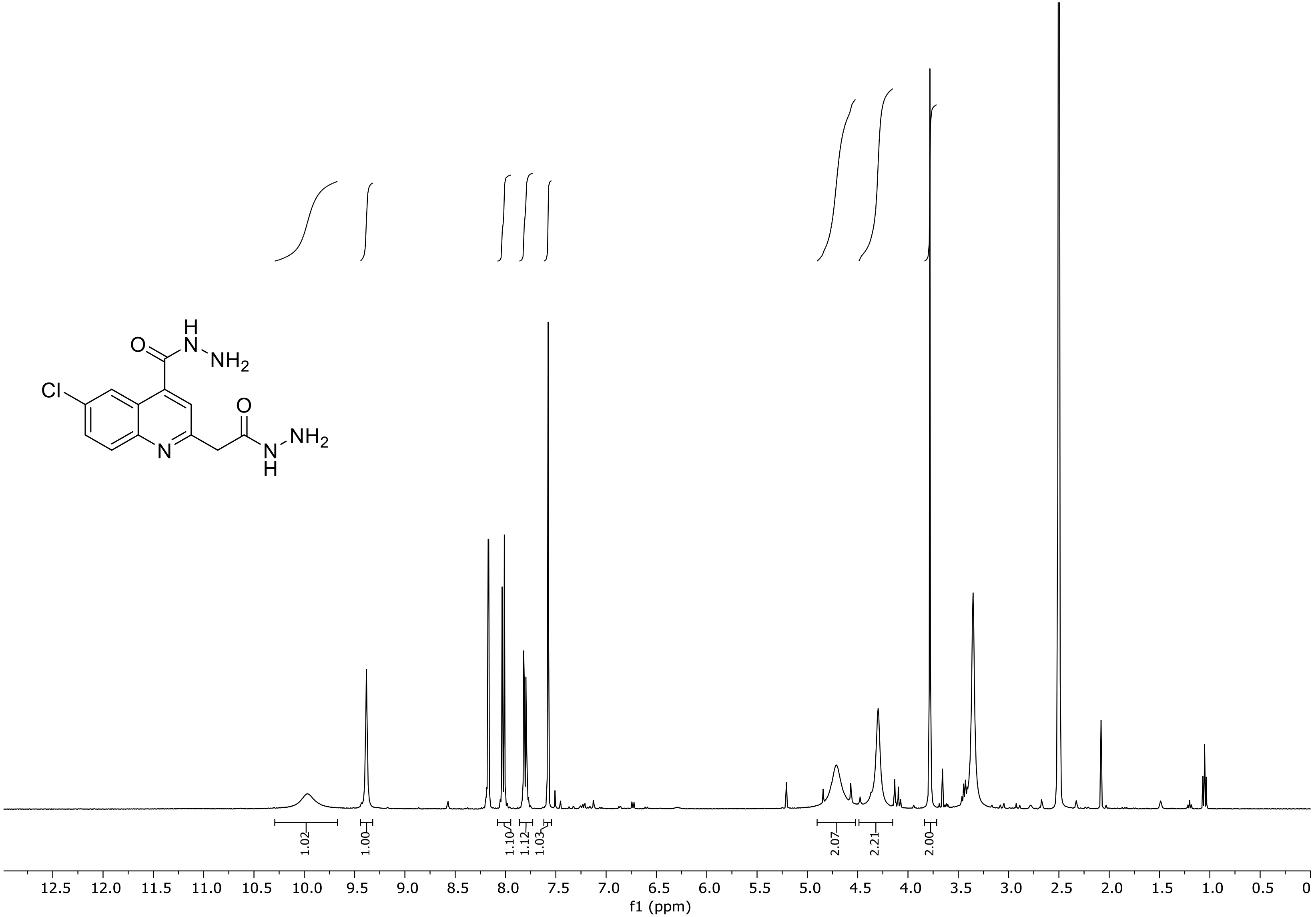

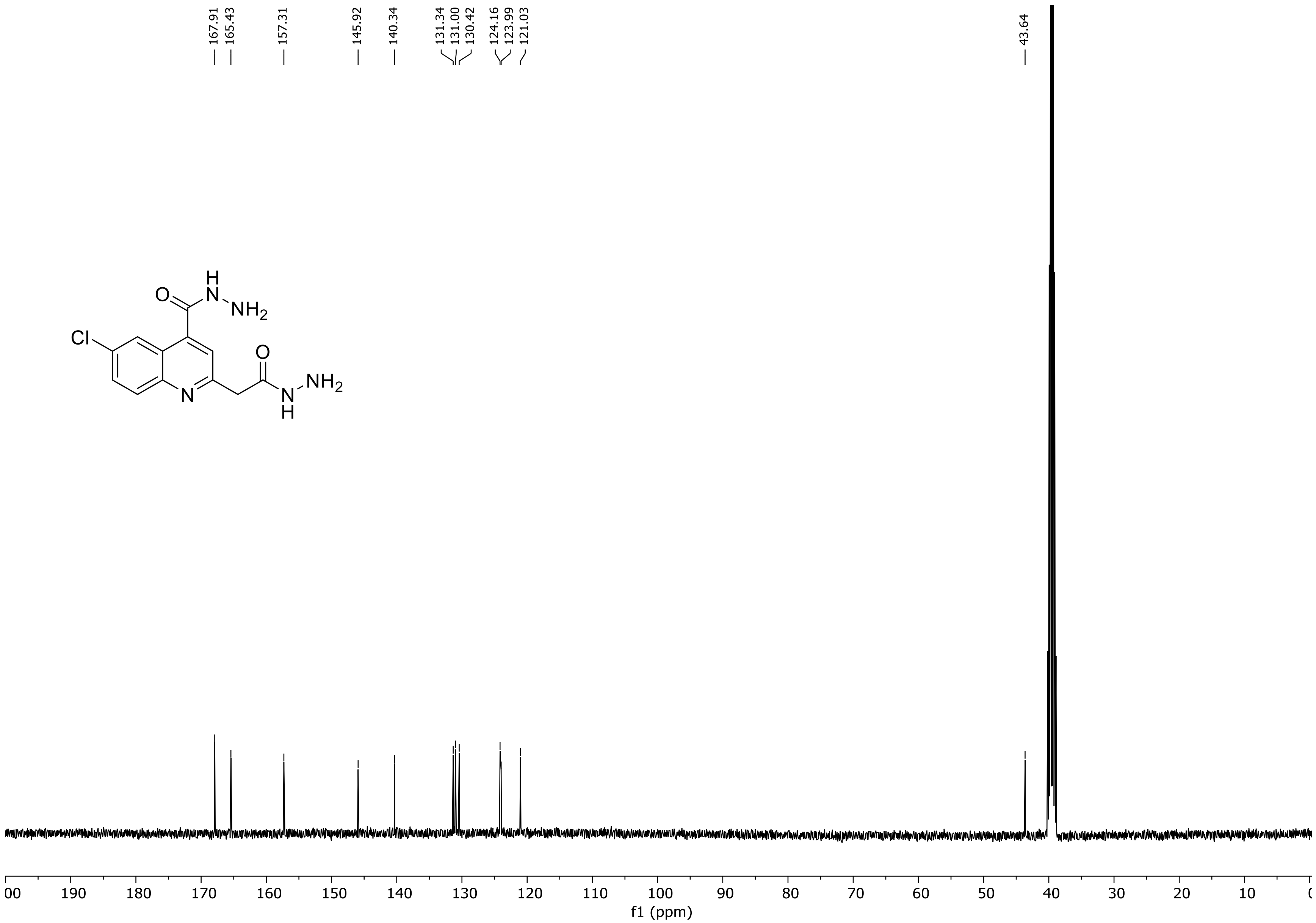

167.91
165.43
157.31
145.92
140.34
131.34
131.00
130.42
124.16
123.99
121.03
43.64
Cl
O
H
N
NH2
N
O
N
NH2
H
00
190
180
170
160
150
140
130
120
110
100
90
80
70
60
50
40
30
20
10
f1 (ppm)

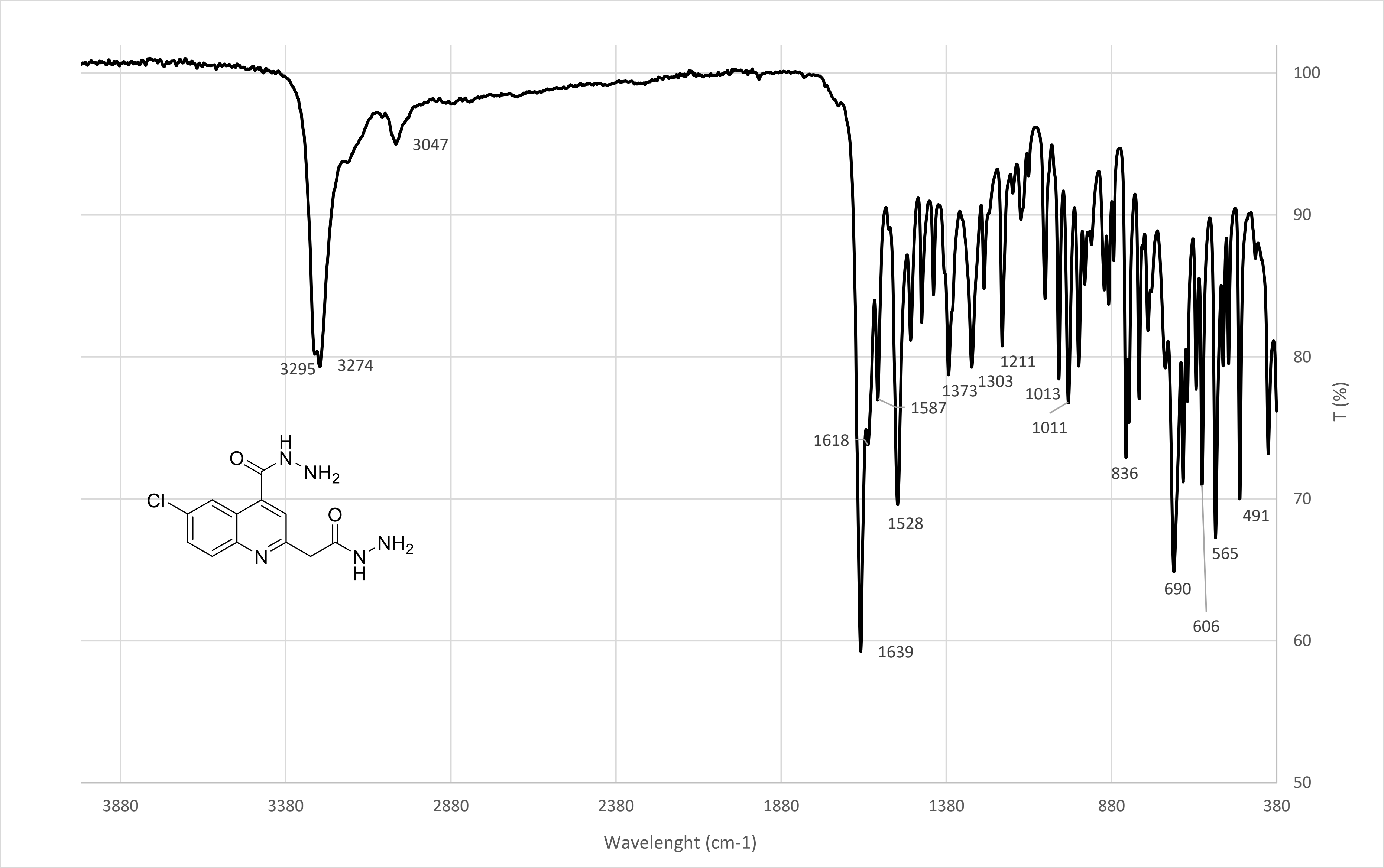
3295
3274
3047
1618
1639
1587
1528
1373
1303
1211
1013
1011
836
690
606
565
491
100
90
80
70
60
50
T (%)
3880
3380
2880
2380
1880
1380
880
380
Wavelenght (cm-1)
O
H
N
NH2
Cl
N
O
N
NH2
H

Single Mass Analysis

Tolerance = 5.0 PPM / DBE: min = -1.5, max = 100.0
Element prediction: Off
Number of isotope peaks used for i-FIT = 5

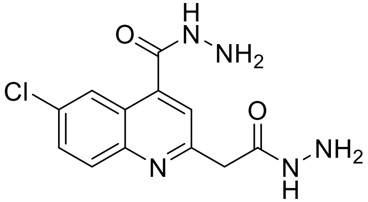

Monoisotopic Mass, Even Electron Ions
377 formula(e) evaluated with 3 results within limits (up to 500 closest results for each mass)
Elements Used:
C: 0-50 H: 0-50 N: 0-7 O: 0-6 Cl: 0-1

QToF Premier

21-May-2019
GG221C 215 (1.815) Cm (213:220) 1: TOF MS ES+
5.78e+004

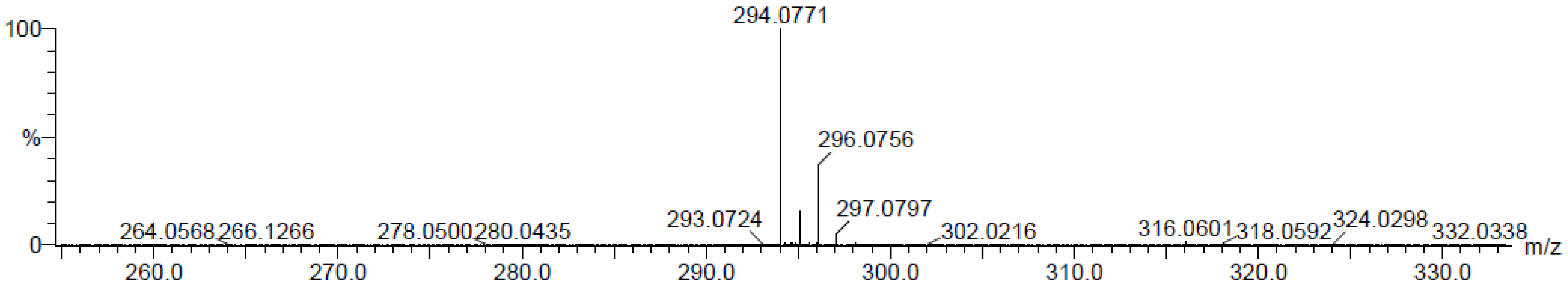

Minimum:				-1.5			
Maximum:		3.0	5.0	100.0			
Mass	Calc. Mass	mDa	PPM	DBE	i-FIT	i-FIT (Norm)	Formula
294.0771	294.0766	0.5	1.7	12.5	749.0	14.3	C17 H12 N O4
	294.0780	-0.9	-3.1	17.5	749.3	14.6	C18 H8 N5
	294.0758	1.3	4.4	8.5	734.7	0.0	C12 H13 N5 O2 Cl

*1.16 Data of 6-fluoro-2-(2-hydrazinyl-2-oxoethyl)quinoline-4-carbohydrazide (**16f**)*

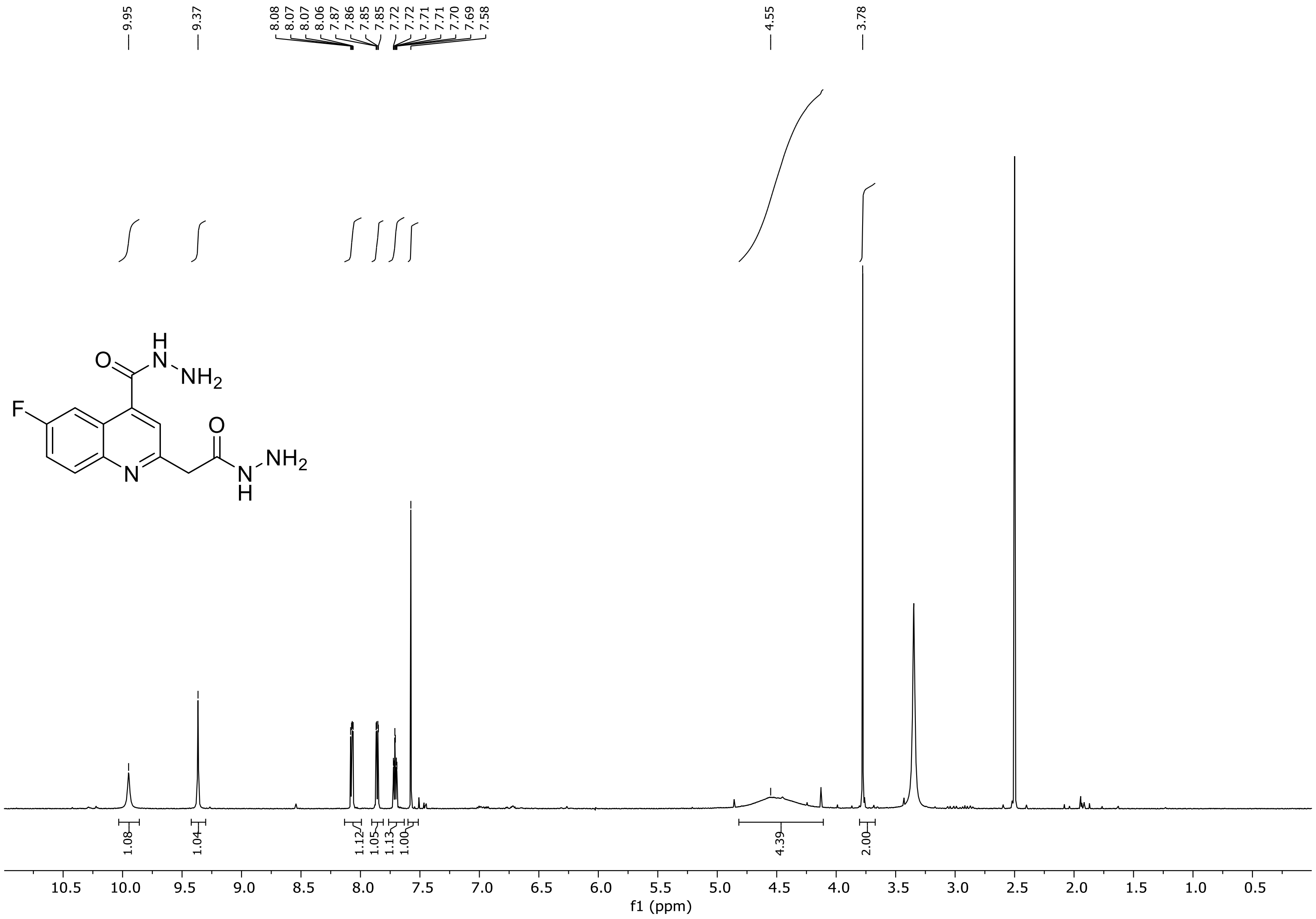

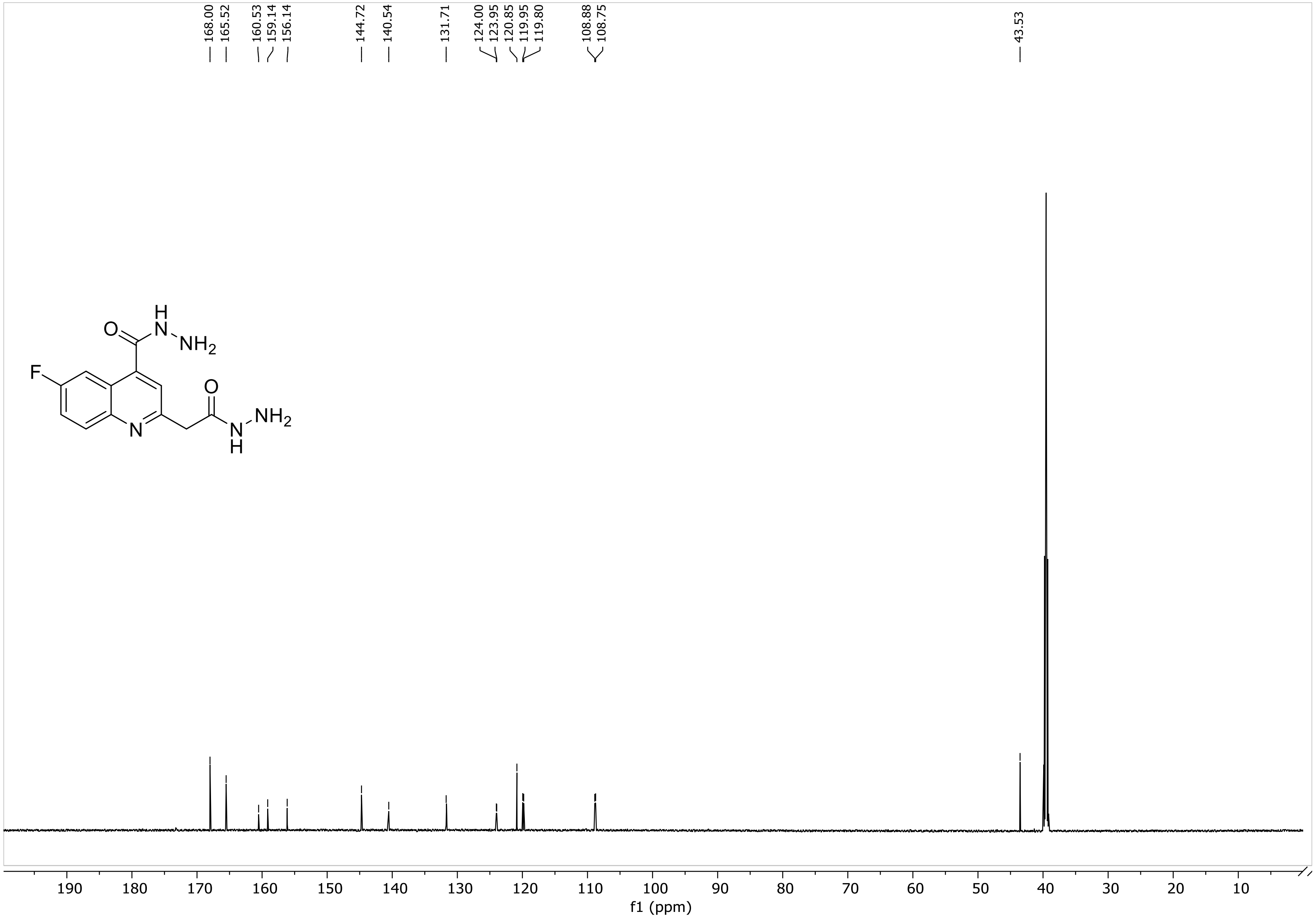
168.00
165.52
160.53
159.14
156.14
144.72
140.54
131.71
124.00
123.95
120.85
119.95
119.80
108.88
108.75
43.53
190 180 170 160 150 140 130 120 110 100 90 80 70 60 50 40 30 20 10
f1 (ppm)

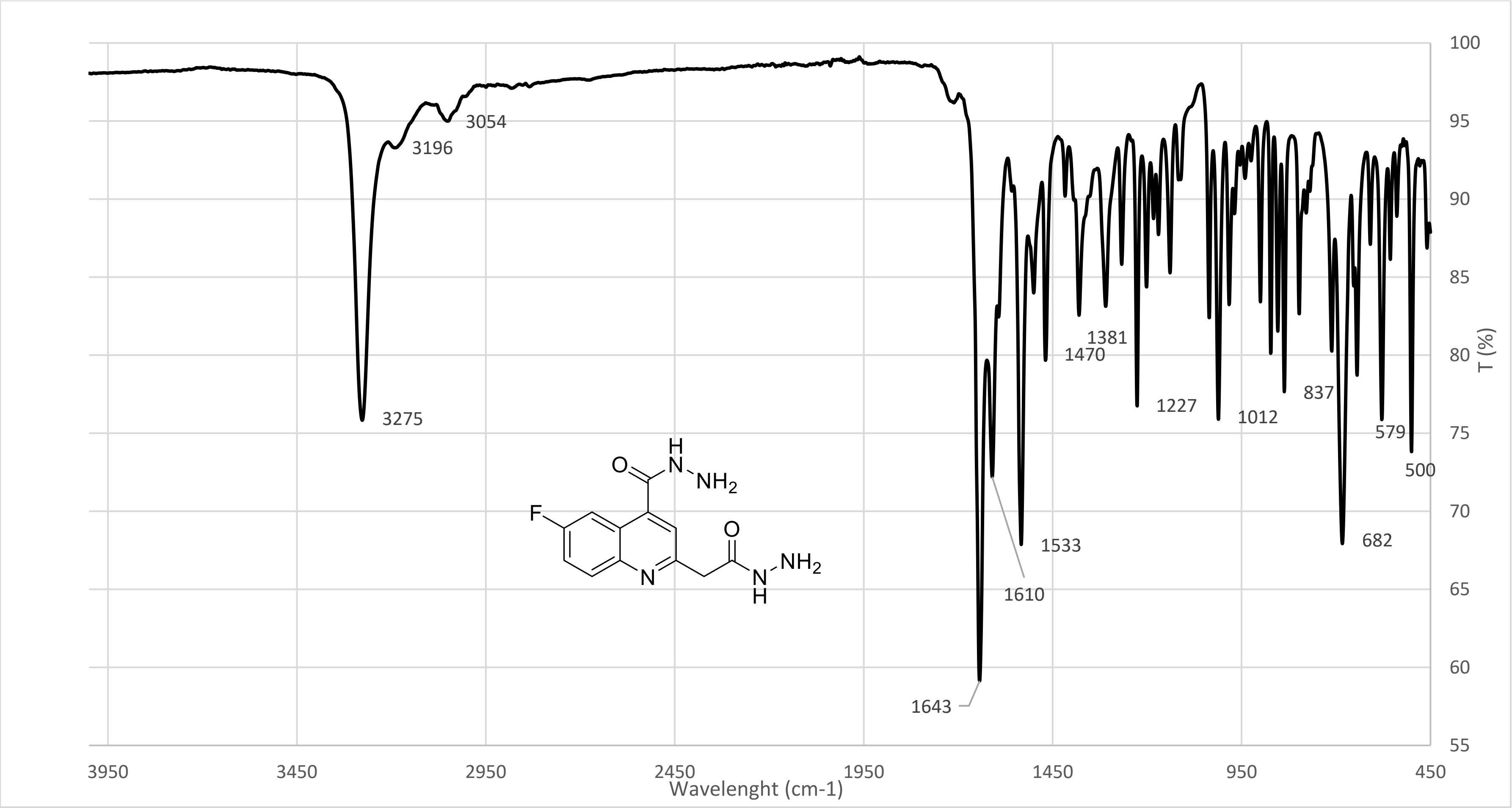

100
95
90
85
80
75
70
65
60
55
T (%)
3950
3450
2950
2450
1950
1450
950
450
Wavelenght (cm-1)
3275
3196
3054
1643
1610
1533
1470
1381
1227
1012
837
682
579
500
F
N
O
H
N
NH2
O
N
H
NH2

Elemental Composition Report

Single Mass Analysis

Tolerance = 3.0 mDa / DBE: min = -1.5, max = 100.0
Element prediction: Off
Number of isotope peaks used for i-FIT = 5

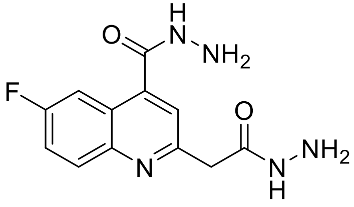

Monoisotopic Mass, Even Electron Ions
879 formula(e) evaluated with 9 results within limits (up to 500 closest results for each mass)
Elements Used:
C: 0-40 H: 0-80 N: 0-8 O: 0-8 F: 0-3

QToF Premier

27-Nov-2019
GG312B 161 (1.364)

1: TOF MS ES+
1.68e+004

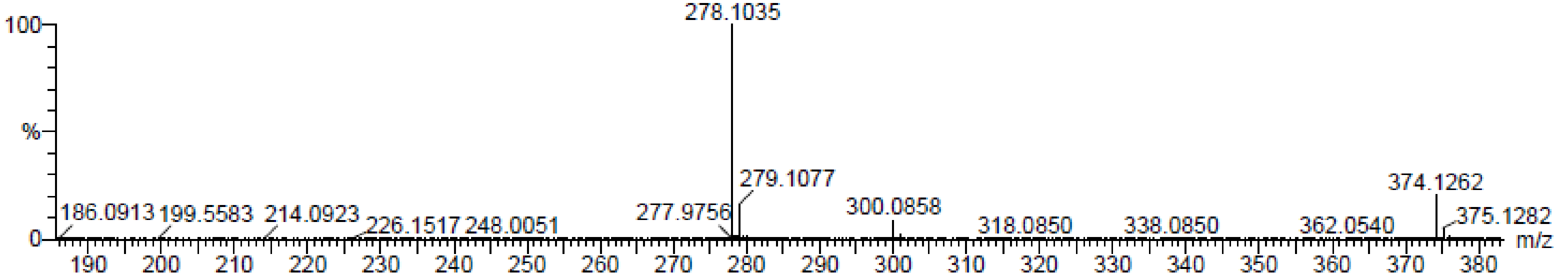

Mass	Calc. Mass	mDa	PPM	DBE	i-FIT	i-FIT (Norm)	Formula
Minimum:				-1.5			
Maximum:		3.0	5.0	100.0			
278.1035	278.1040	-0.5	-1.8	3.5	613.2	4.0	C11 H17 N O6 F
	278.1053	-1.8	-6.5	8.5	610.5	1.3	C12 H13 N5 O2 F
	278.1028	0.7	2.5	7.5	609.6	0.4	C14 H16 N O5
	278.1042	-0.7	-2.5	12.5	612.2	3.0	C15 H12 N5 O
	278.1060	-2.5	-9.0	-0.5	624.1	14.9	C3 H16 N7 O8
	278.1024	1.1	4.0	0.5	623.2	14.1	C4 H14 N7 O5 F2
	278.1013	2.2	7.9	4.5	619.9	10.7	C7 H13 N7 O4 F
	278.1051	-1.6	-5.8	-0.5	617.5	8.3	C8 H18 N O7 F2
	278.1065	-3.0	-10.8	4.5	617.1	7.9	C9 H14 N5 O3 F2

*1.17 Data of 7-chloro-2-(2-hydrazinyl-2-oxoethyl)quinoline-4-carbohydrazide (**16g**)*

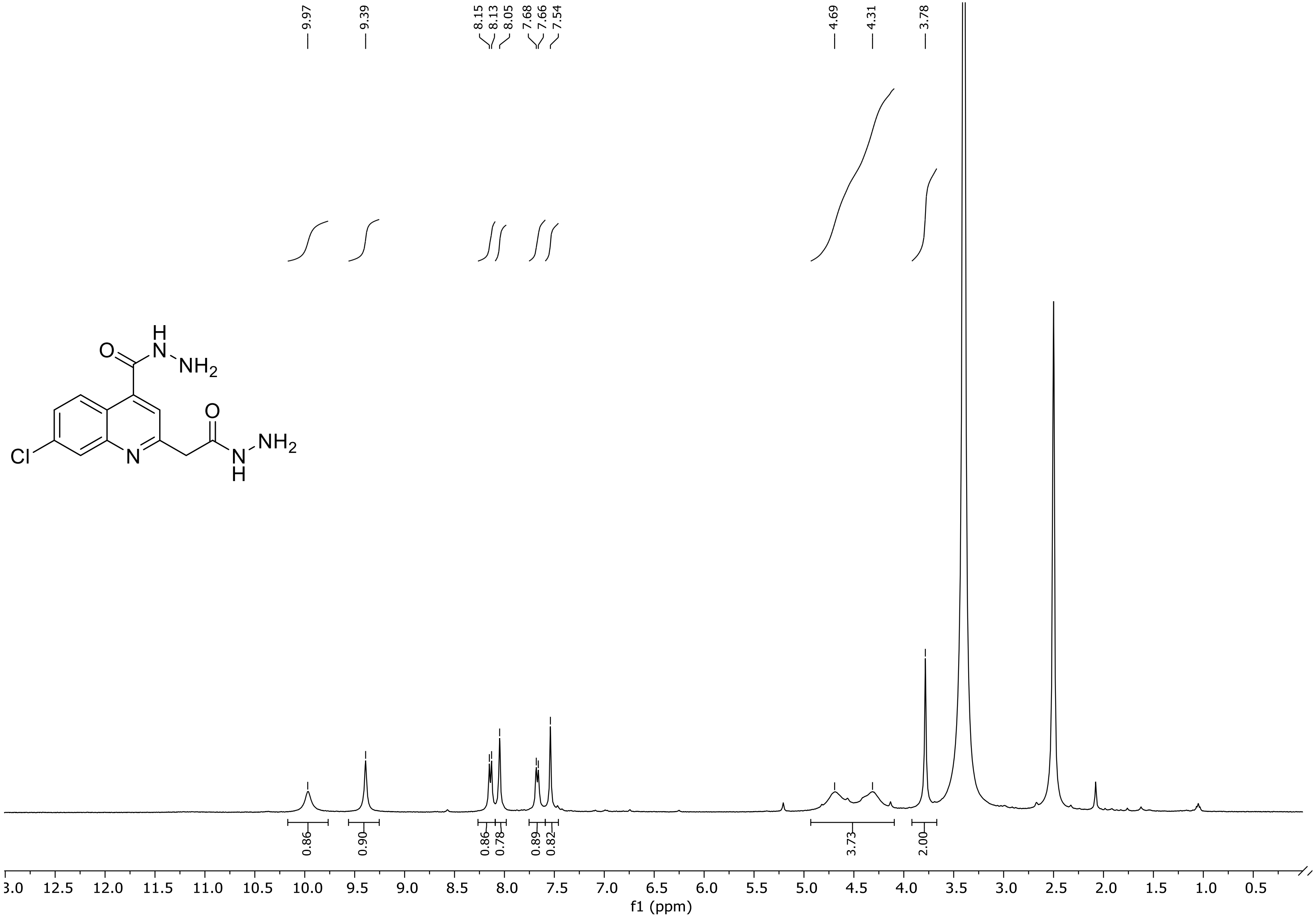

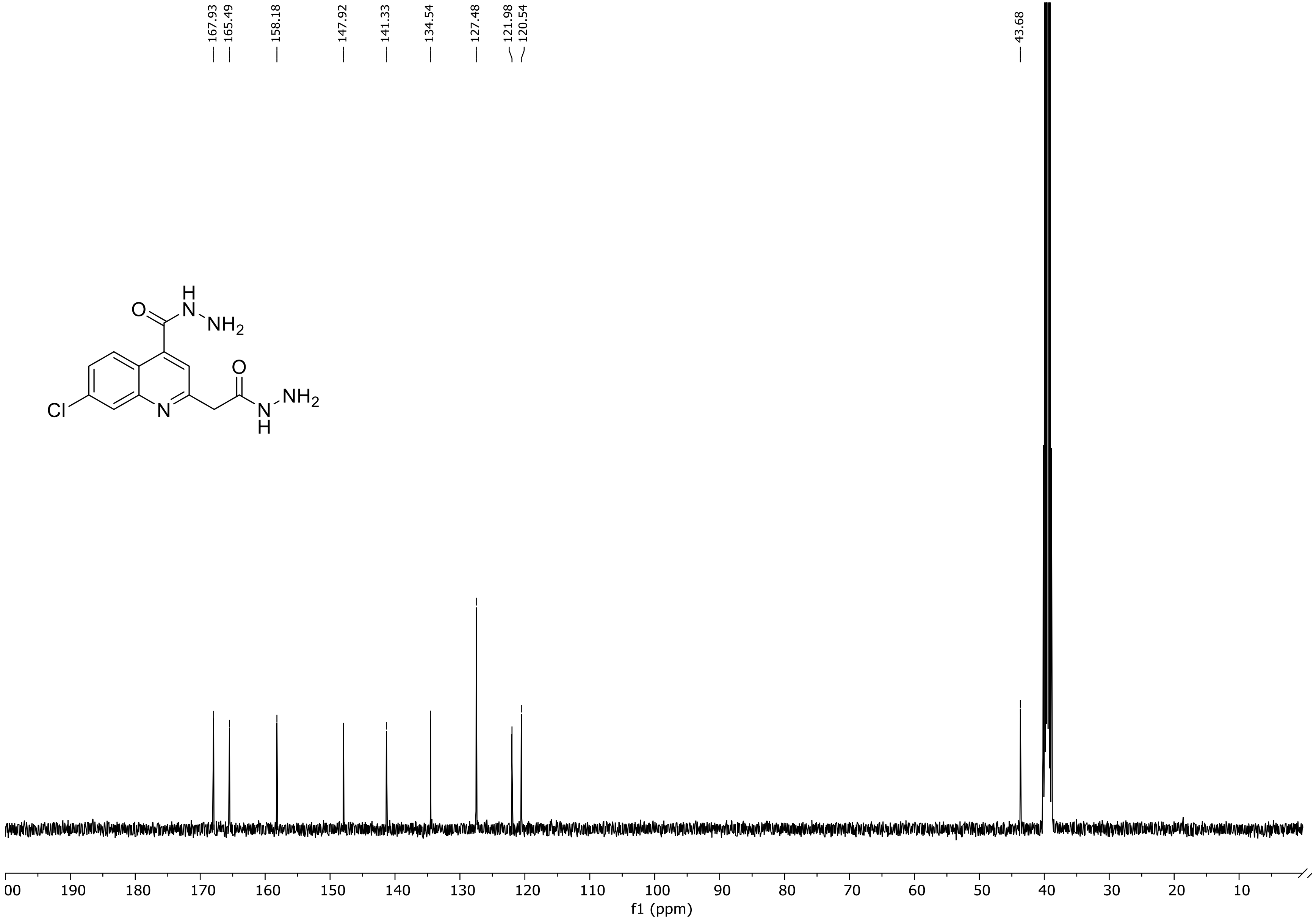
167.93
165.49
158.18
147.92
141.33
134.54
127.48
121.98
120.54
43.68
Cl
N
O
H
N
NH2
O
N
H
NH2
00
190
180
170
160
150
140
130
120
110
100
90
80
70
60
50
40
30
20
10
f1 (ppm)

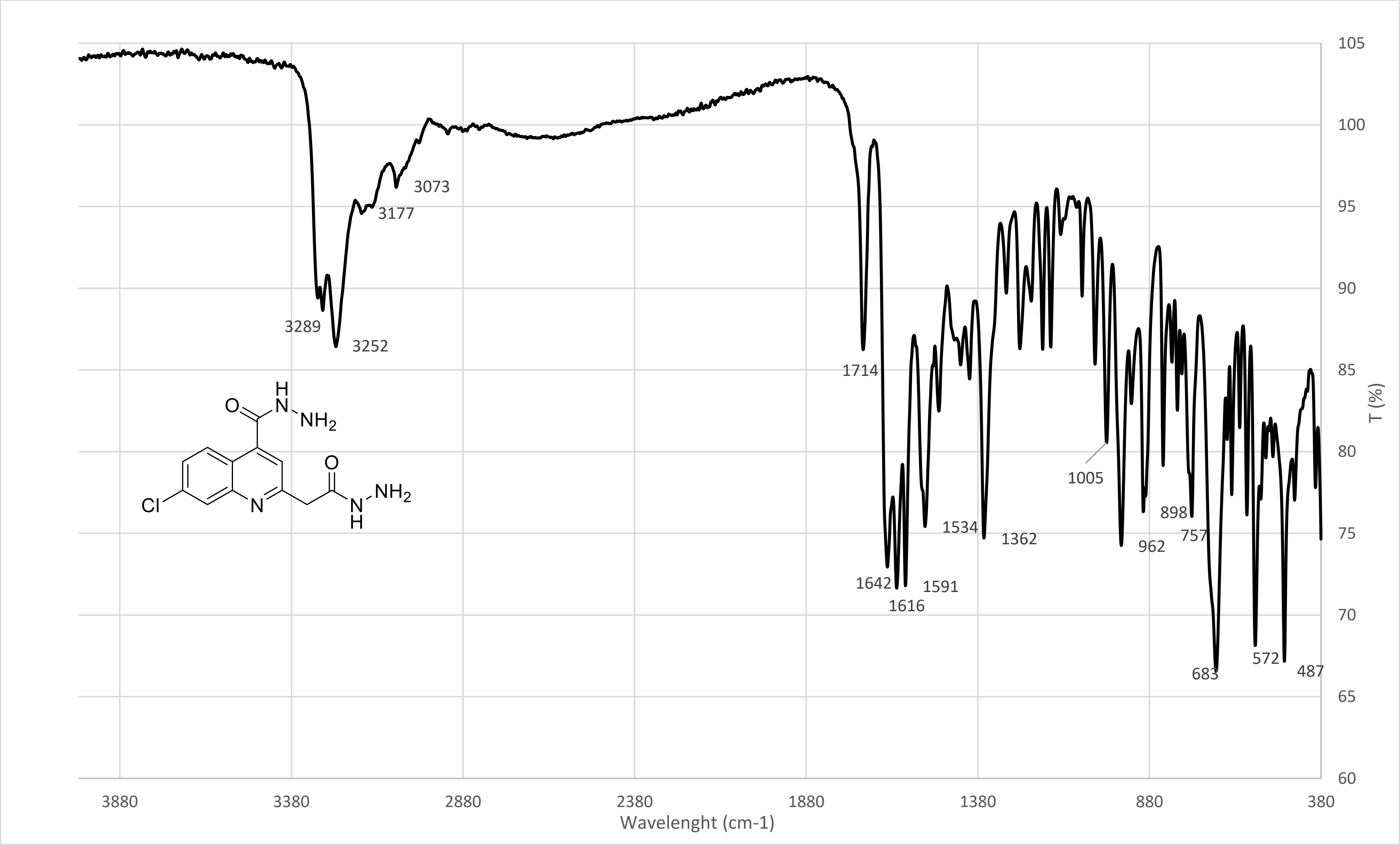
3289
3252
3177
3073
1714
1642
1616
1591
1534
1362
1005
962
898
757
683
572
487
105
100
95
90
85
80
75
70
65
60
T (%)
3880
3380
2880
2380
1880
1380
880
380
Wavelenght (cm-1)
O
H
N
NH2
Cl
N
O
NH2
N
H

Elemental Composition Report

Single Mass Analysis

Tolerance = 5.0 PPM / DBE: min = -1.5, max = 100.0
Element prediction: Off
Number of isotope peaks used for i-FIT = 5

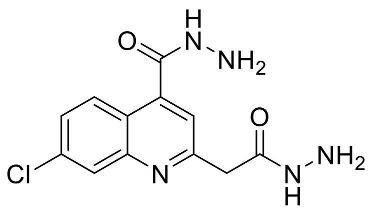

Monoisotopic Mass, Even Electron Ions
377 formula(e) evaluated with 3 results within limits (up to 500 closest results for each mass)
Elements Used:
C: 0-50 H: 0-50 N: 0-7 O: 0-6 Cl: 0-1

QToF Premier

21-May-2019
GG221C 215 (1.815) Cm (213:220)

1: TOF MS ES+
5.78e+004

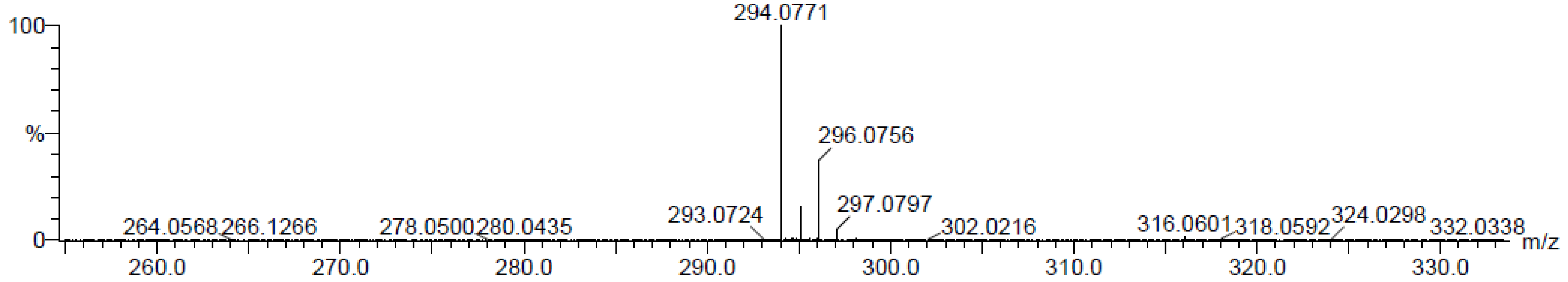

Mass	Calc. Mass	mDa	PPM	DBE	i-FIT	i-FIT (Norm)	Formula
Minimum:				-1.5			
Maximum:		3.0	5.0	100.0			
294.0771	294.0766	0.5	1.7	12.5	749.0	14.3	C17 H12 N O4
	294.0780	-0.9	-3.1	17.5	749.3	14.6	C18 H8 N5
	294.0758	1.3	4.4	8.5	734.7	0.0	C12 H13 N5 O2 Cl

*1.18 Data of 2-(2-hydrazinyl-2-oxoethyl)-8-(trifluoromethyl)quinoline-4-carbohydrazide (**16i**)*

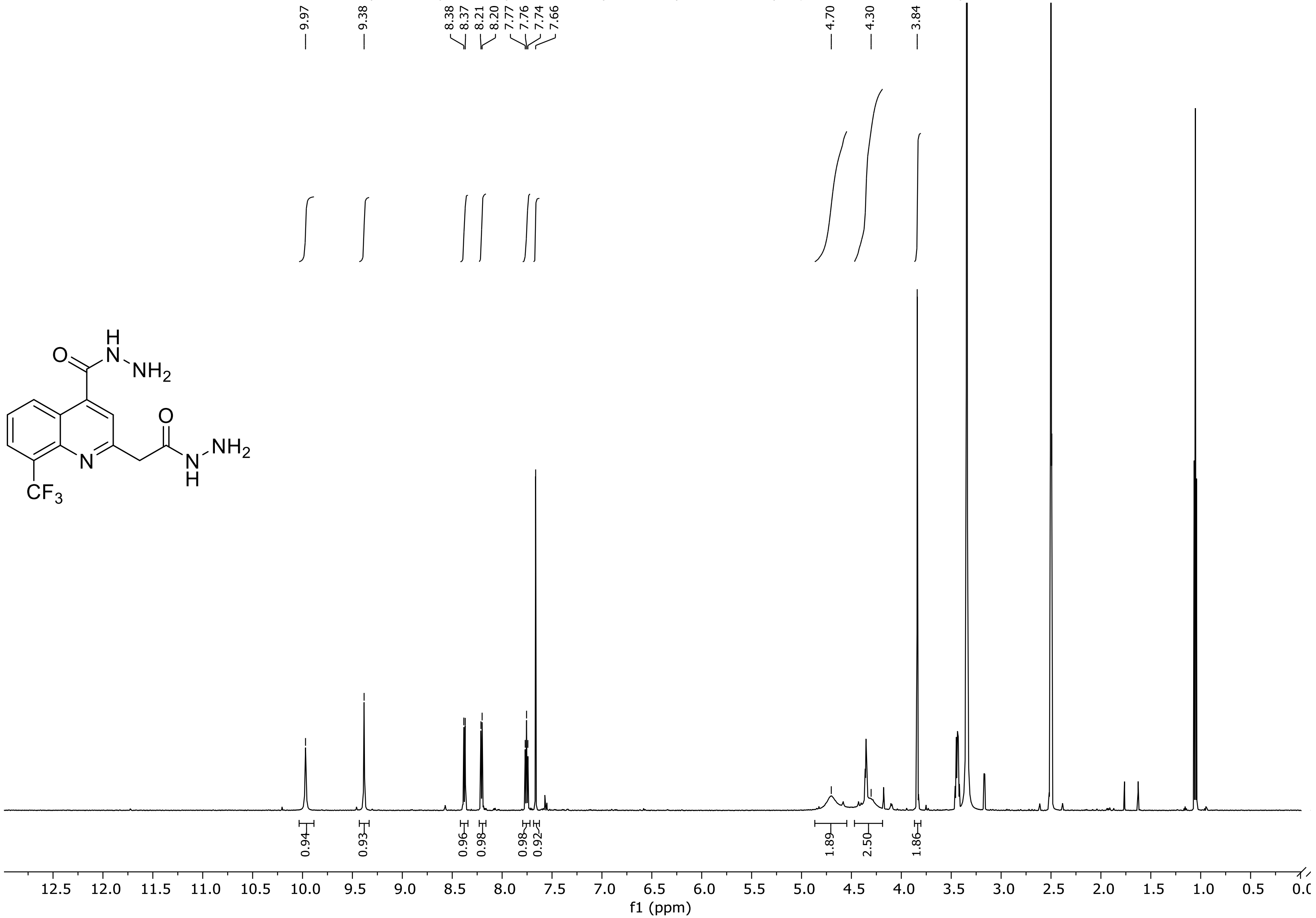

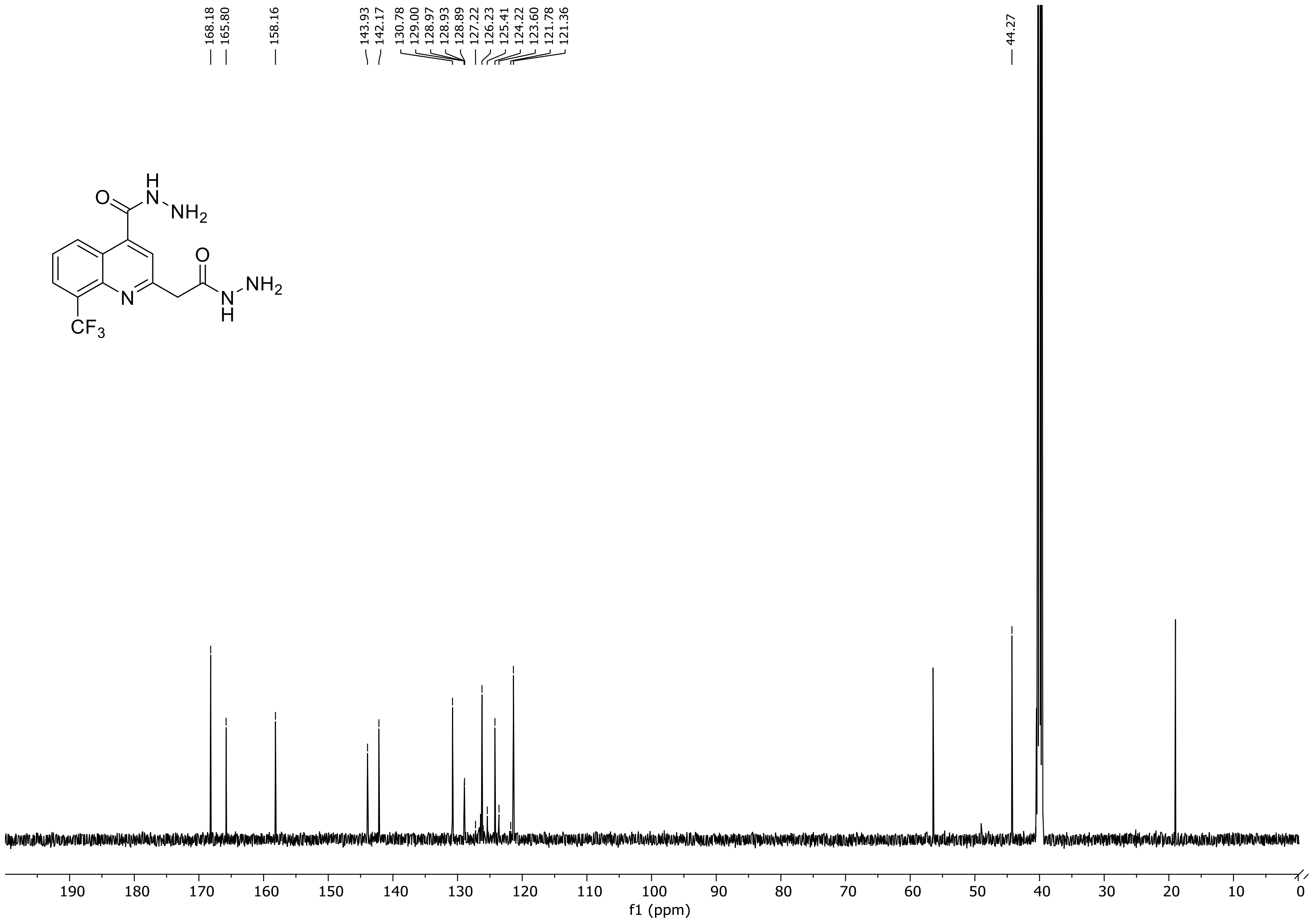
168.18
165.80
158.16
143.93
142.17
130.78
129.00
128.97
128.93
128.89
127.22
126.23
125.41
124.22
123.60
121.78
121.36
44.27
O
H
N
NH2
O
NH2
N
H
N
CF3
190
180
170
160
150
140
130
120
110
100
90
80
70
60
50
40
30
20
10
0
f1 (ppm)

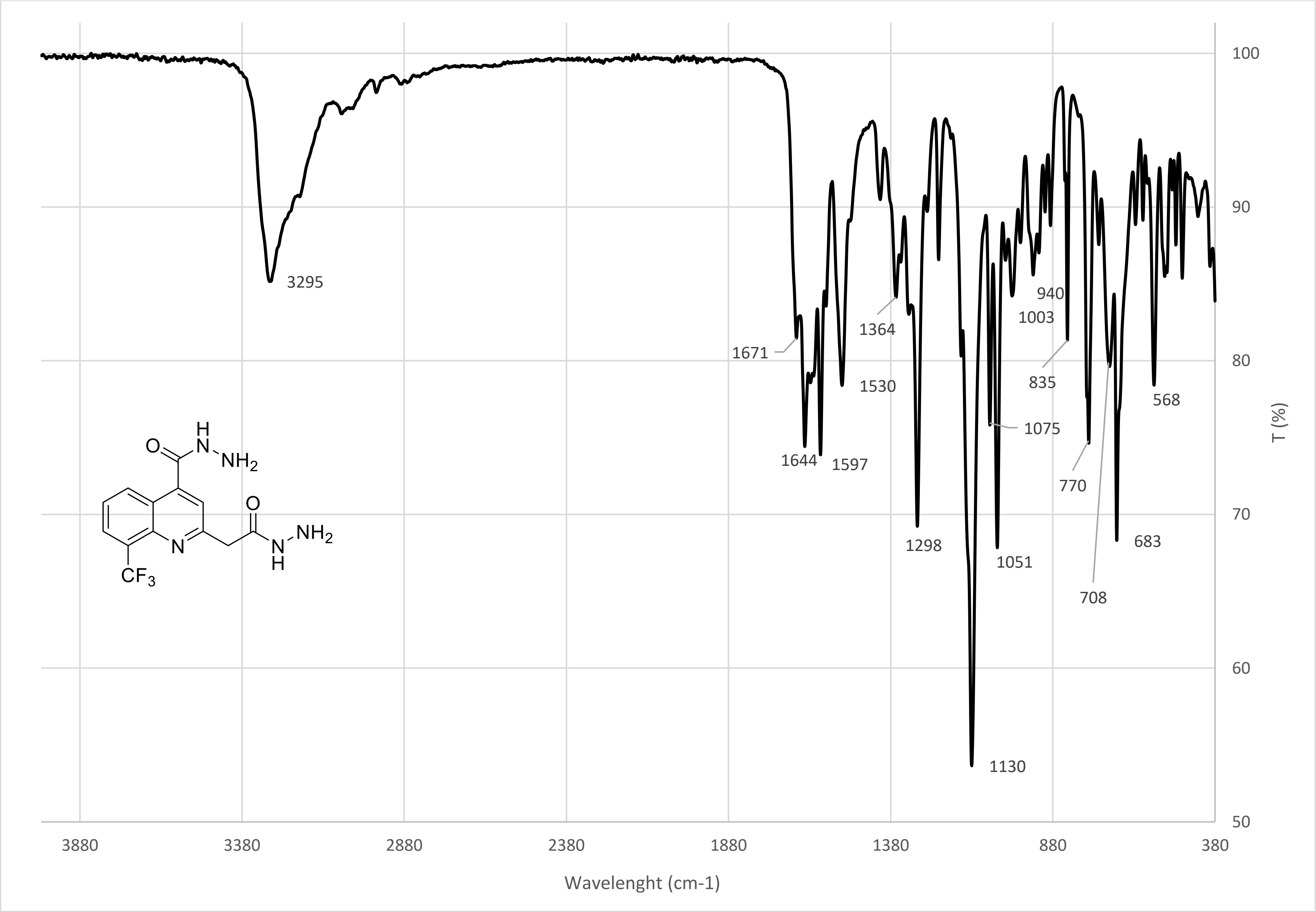

3295
1671
1644
1597
1530
1364
1298
1130
1075
1051
1003
940
835
770
708
683
568
100
90
80
70
60
50
T (%)
3880
3380
2880
2380
1880
1380
880
380
Wavelenght (cm-1)

Elemental Composition Report

Single Mass Analysis

Tolerance = 5.0 PPM / DBE: min = -1.5, max = 100.0
Element prediction: Off
Number of isotope peaks used for i-FIT = 5

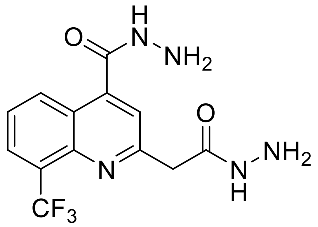

Monoisotopic Mass, Even Electron Ions
542 formula(e) evaluated with 1 results within limits (up to 500 closest results for each mass)
Elements Used:
C: 0-80 H: 0-60 N: 0-5 O: 0-5 F: 0-3

22-Jan-2019
GG150S 248 (2.092) Cm (246:250)

1: TOF MS ES+
6.70e+004

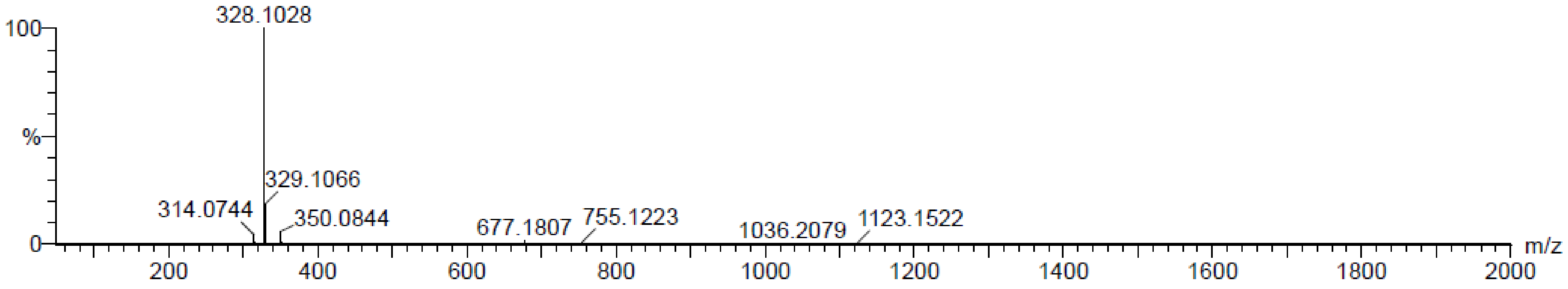

Minimum:				-1.5			
Maximum:		3.0	5.0	100.0			

Mass	Calc. Mass	mDa	PPM	DBE	i-FIT	i-FIT (Norm)	Formula
328.1028	328.1021	0.7	2.1	8.5	602.8	0.0	C13 H13 N5 O2 F3

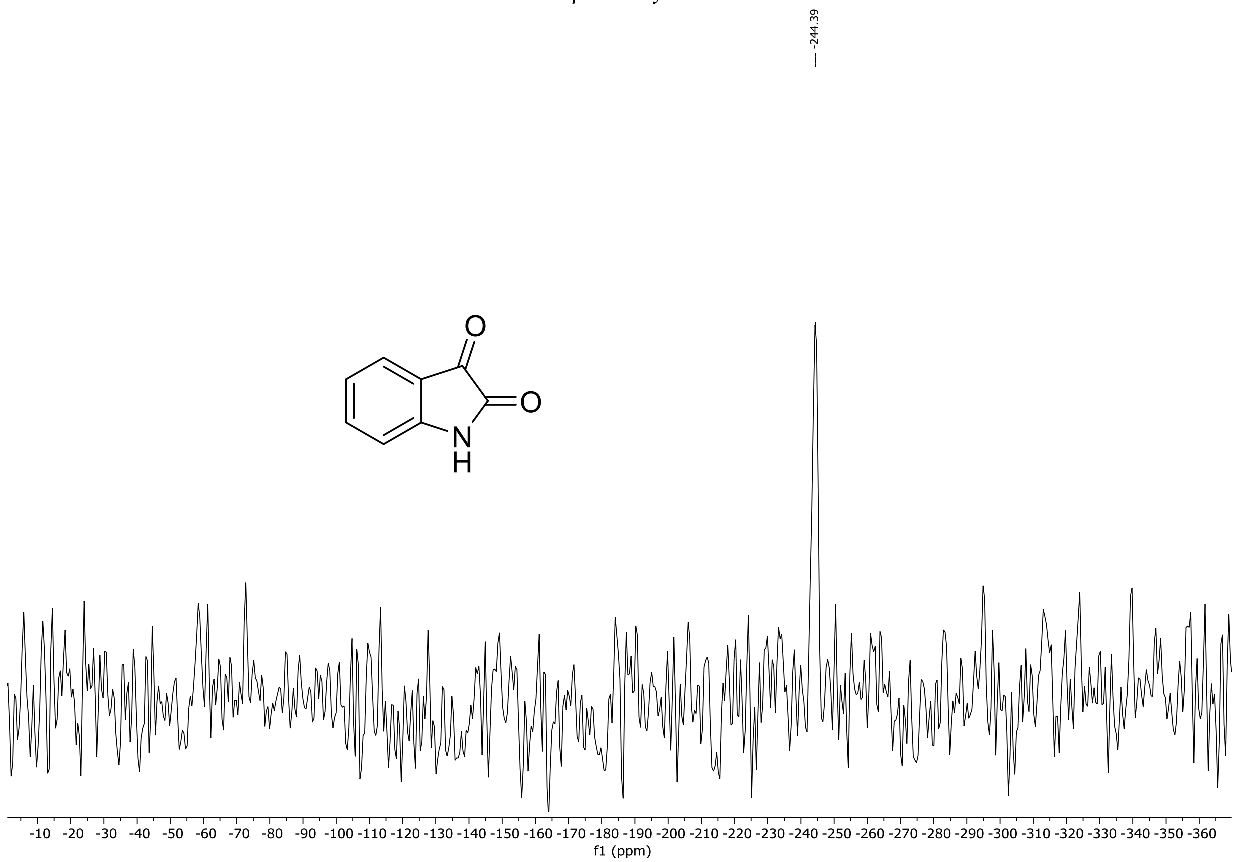

Solid-state nitrogen-15 NMR spectrum recorded with a 900 s recycle delay and 10 ms contact time.

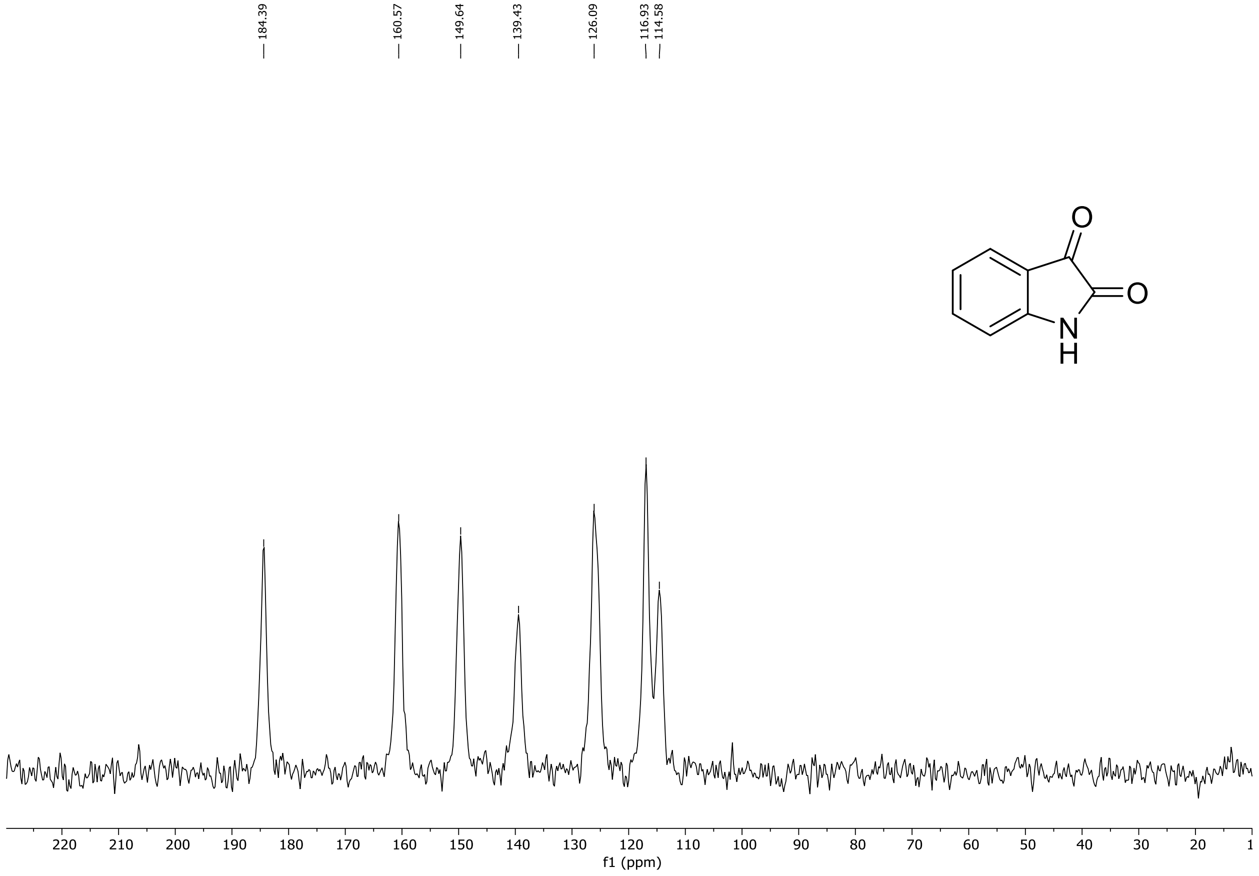

Solid-state carbon-13 NMR spectrum recorded with a 900 s recycle delay and 4 ms contact time.

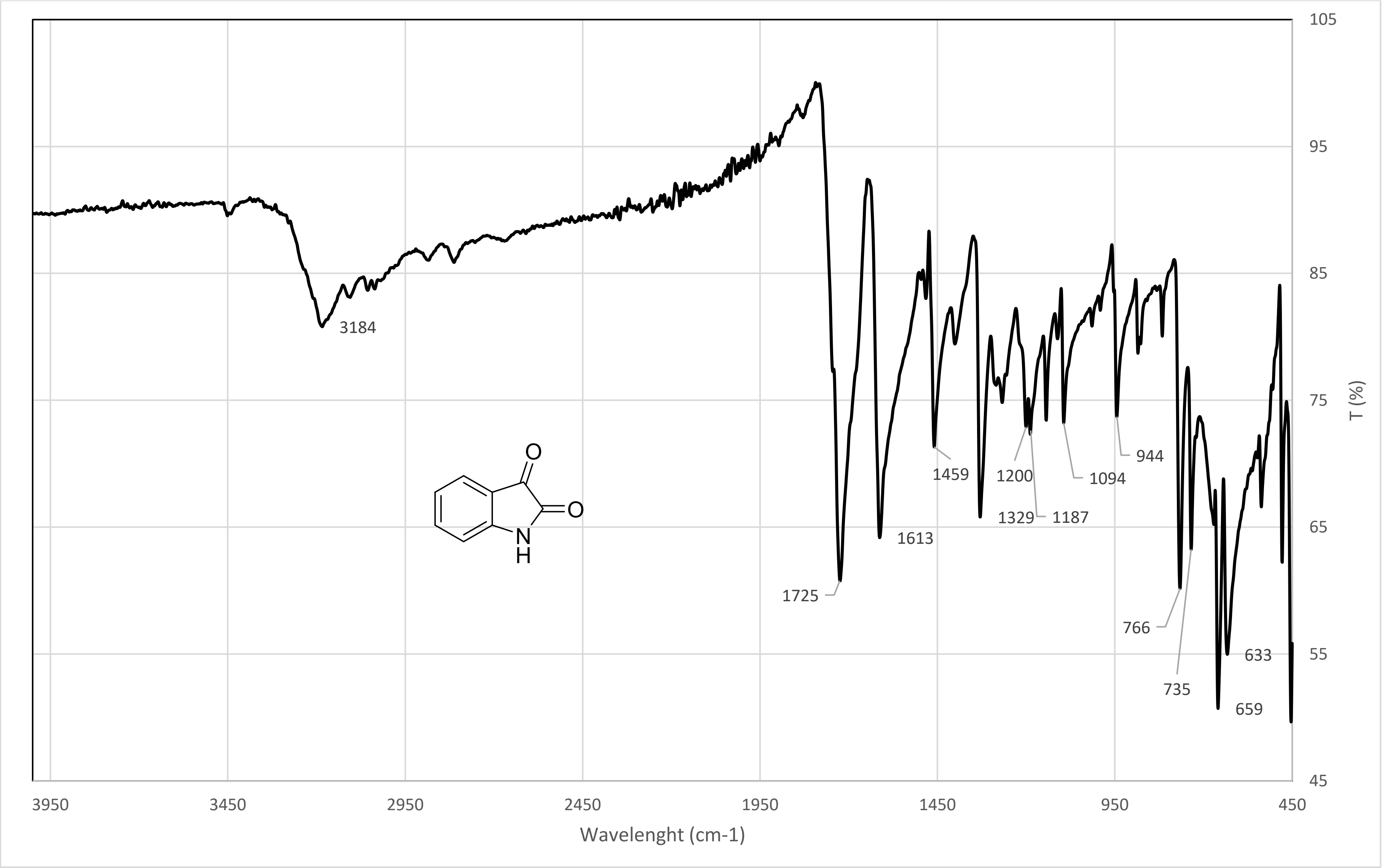

3184
1725
1613
1459
1200
1329
1187
1094
944
766
735
659
633
O
O
N
H
105
95
85
75
65
55
45
T (%)
3950
3450
2950
2450
1950
1450
950
450
Wavelenght (cm-1)

*1.20 Spectra of 4-fluoroisatin (**13a**)*

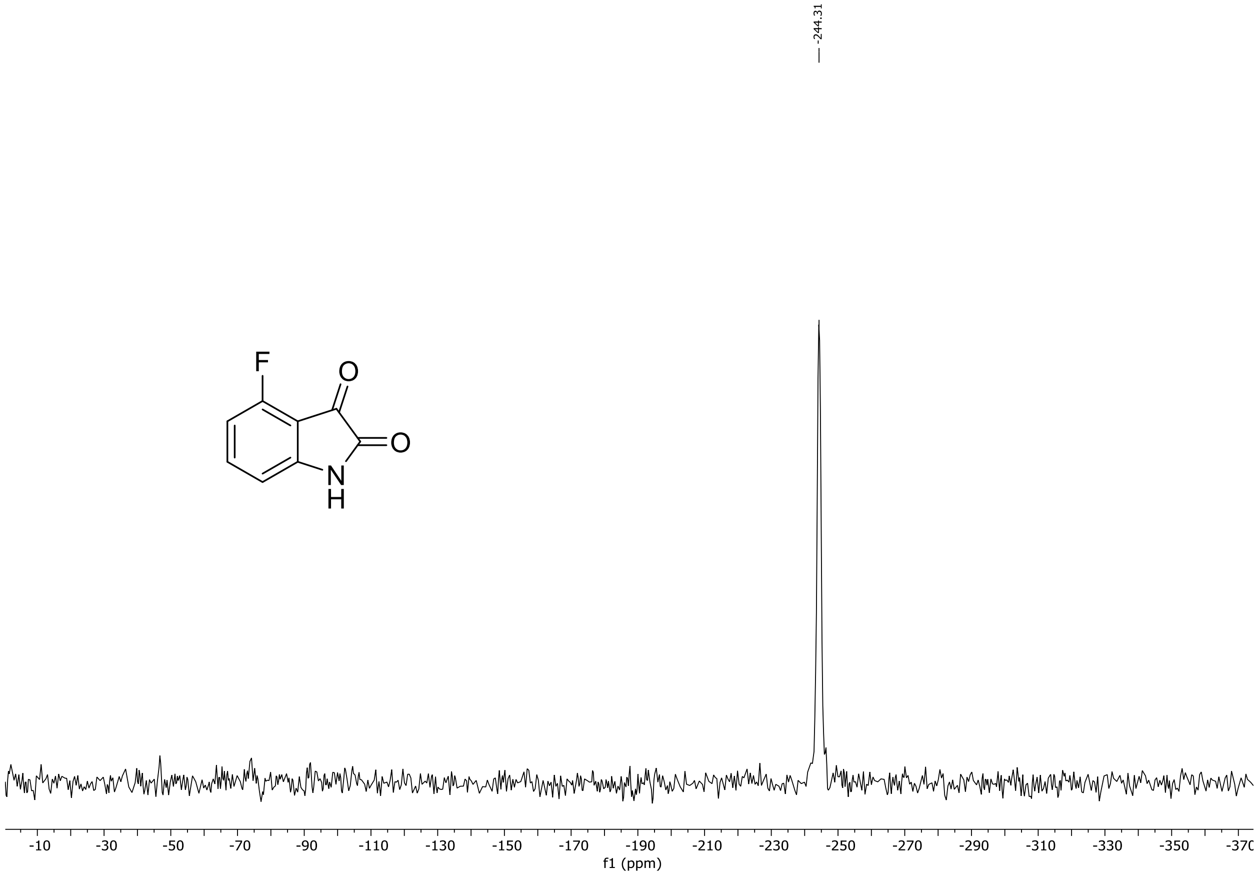

Solid-state nitrogen-15 NMR spectrum recorded with a 4 s recycle delay and 10 ms contact time.

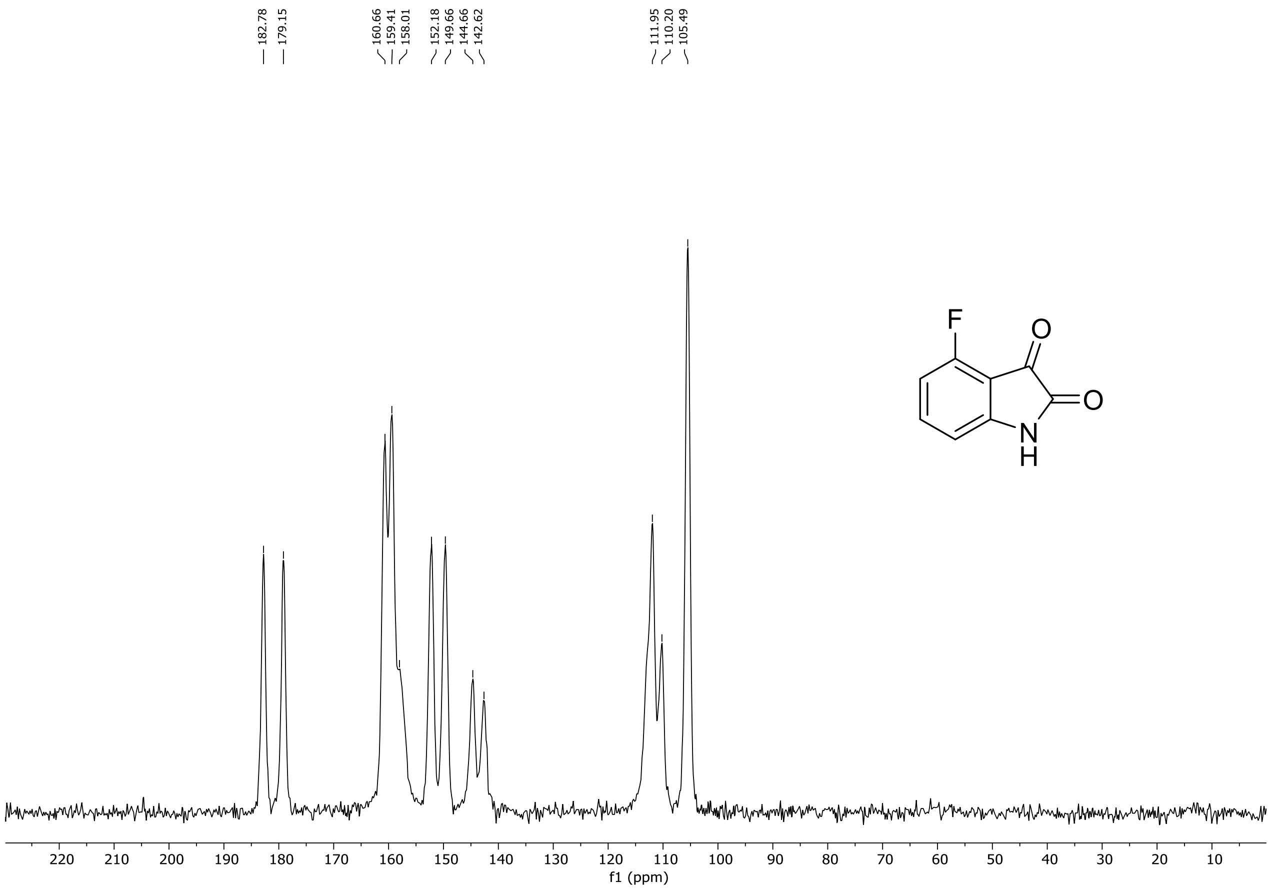

Solid-state carbon-13 NMR spectrum recorded with a 4 s recycle delay and 4 ms contact time.

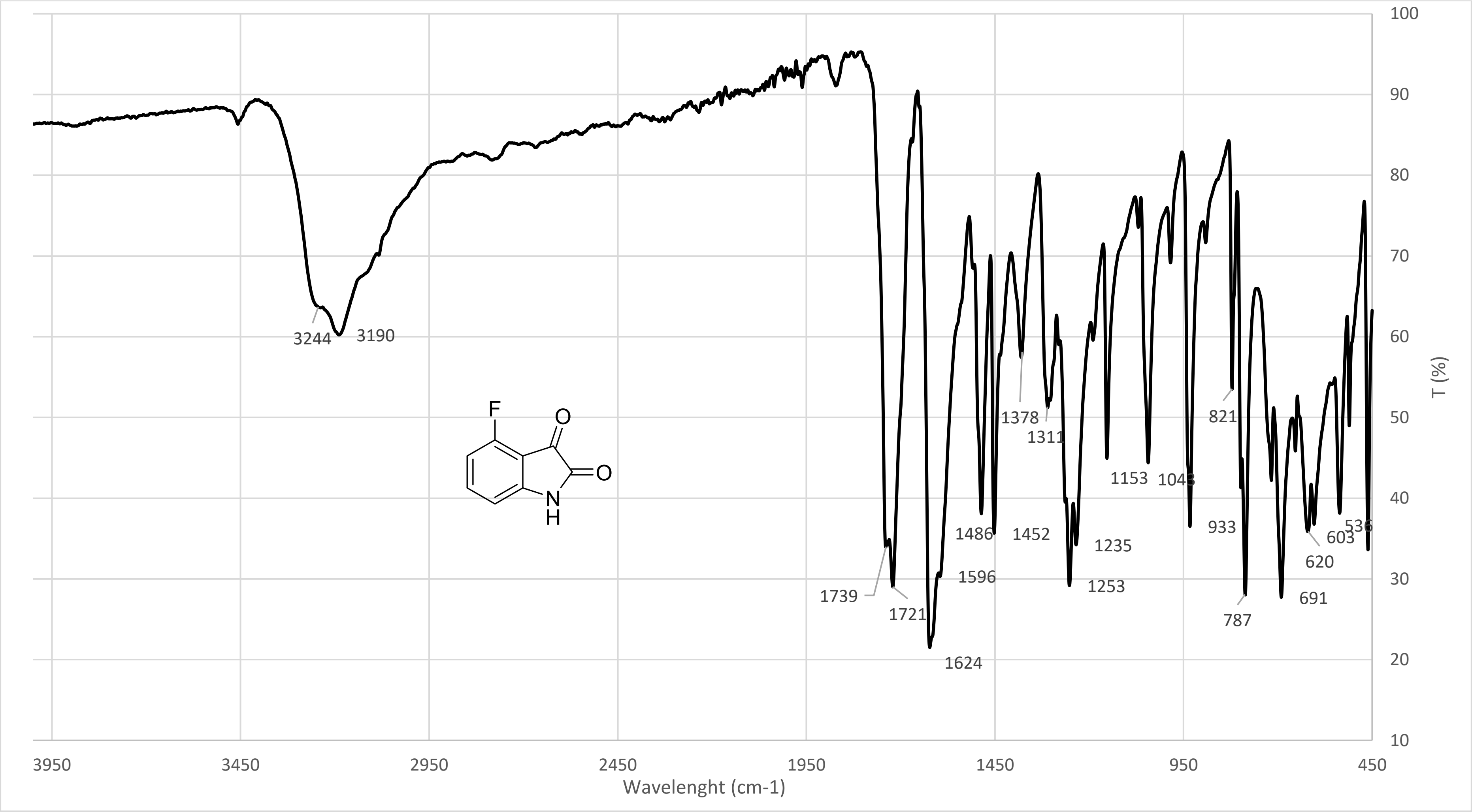
3244
3190
1739
1721
1624
1596
1486
1452
1378
1311
1253
1235
1153
1048
933
821
787
691
620
603
536
100
90
80
70
60
50
40
30
20
10
T (%)
3950
3450
2950
2450
1950
1450
950
450
Wavelenght (cm-1)
F
O
O
N
H

*1.21 Spectra of 5-chloroisatin (**13d**)*

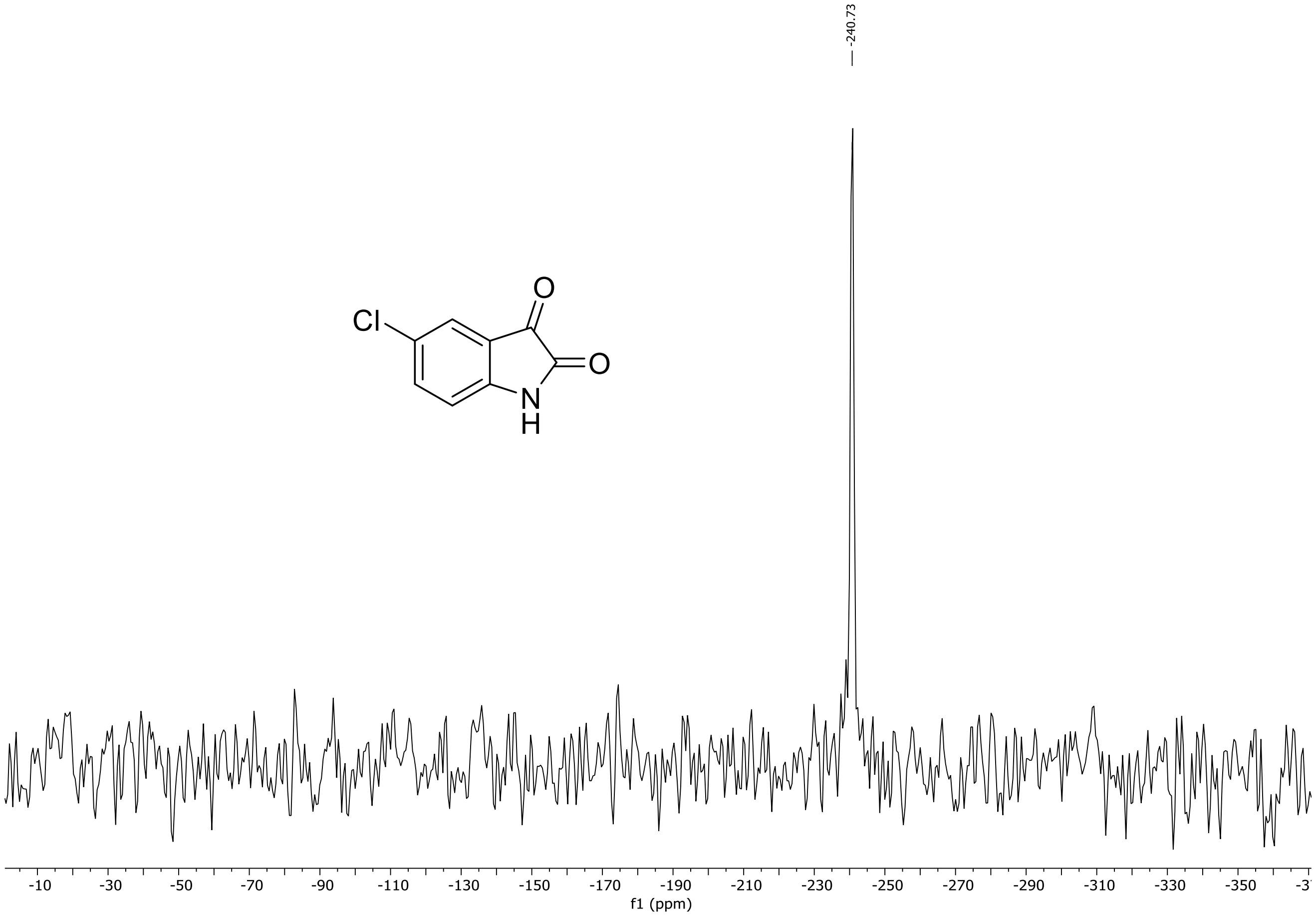

Solid-state nitrogen-15 NMR spectrum recorded with a 20 s recycle delay and 10 ms contact time.

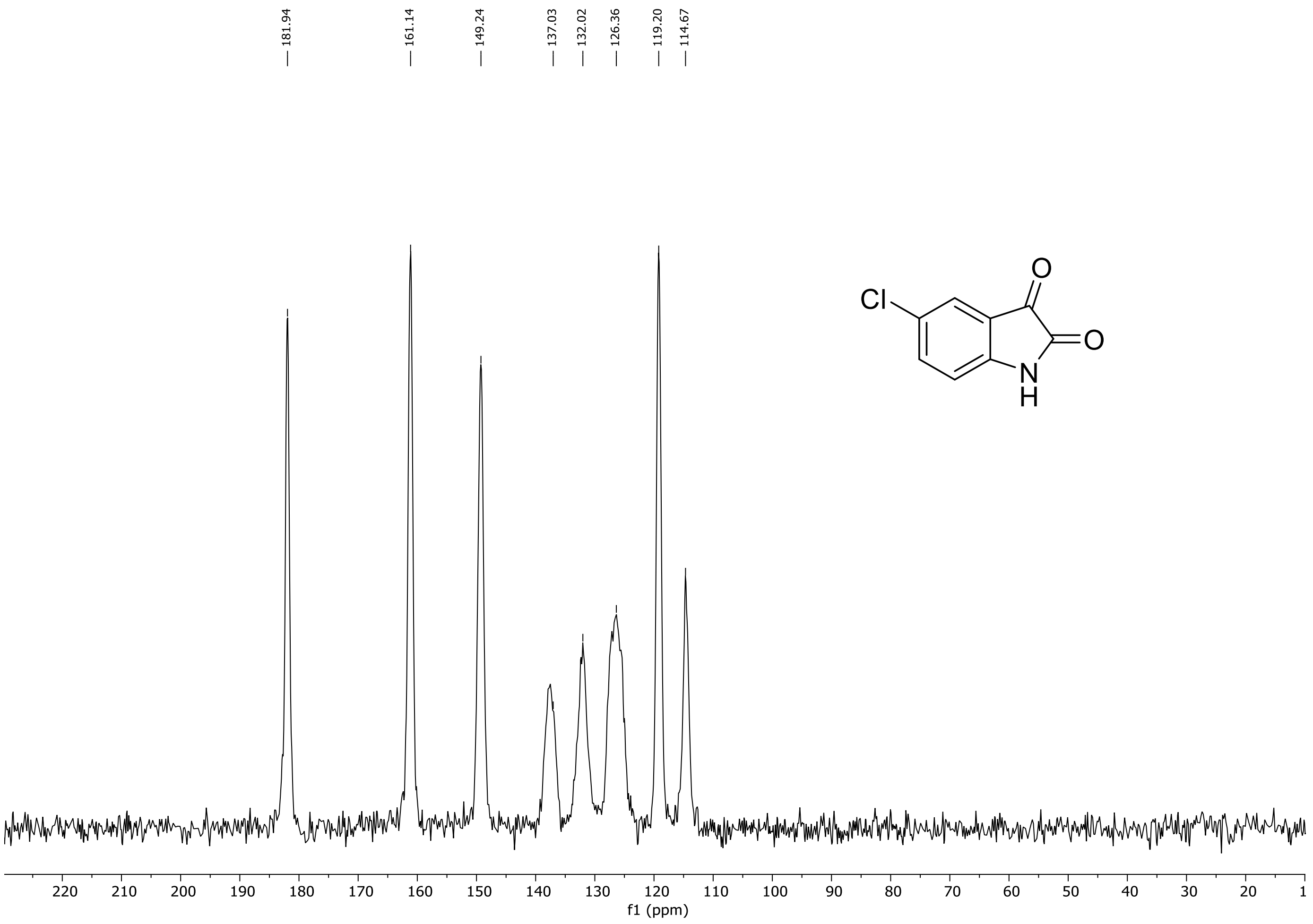

Solid-state carbon-13 NMR spectrum recorded with a 20 s recycle delay and 4 ms contact time.

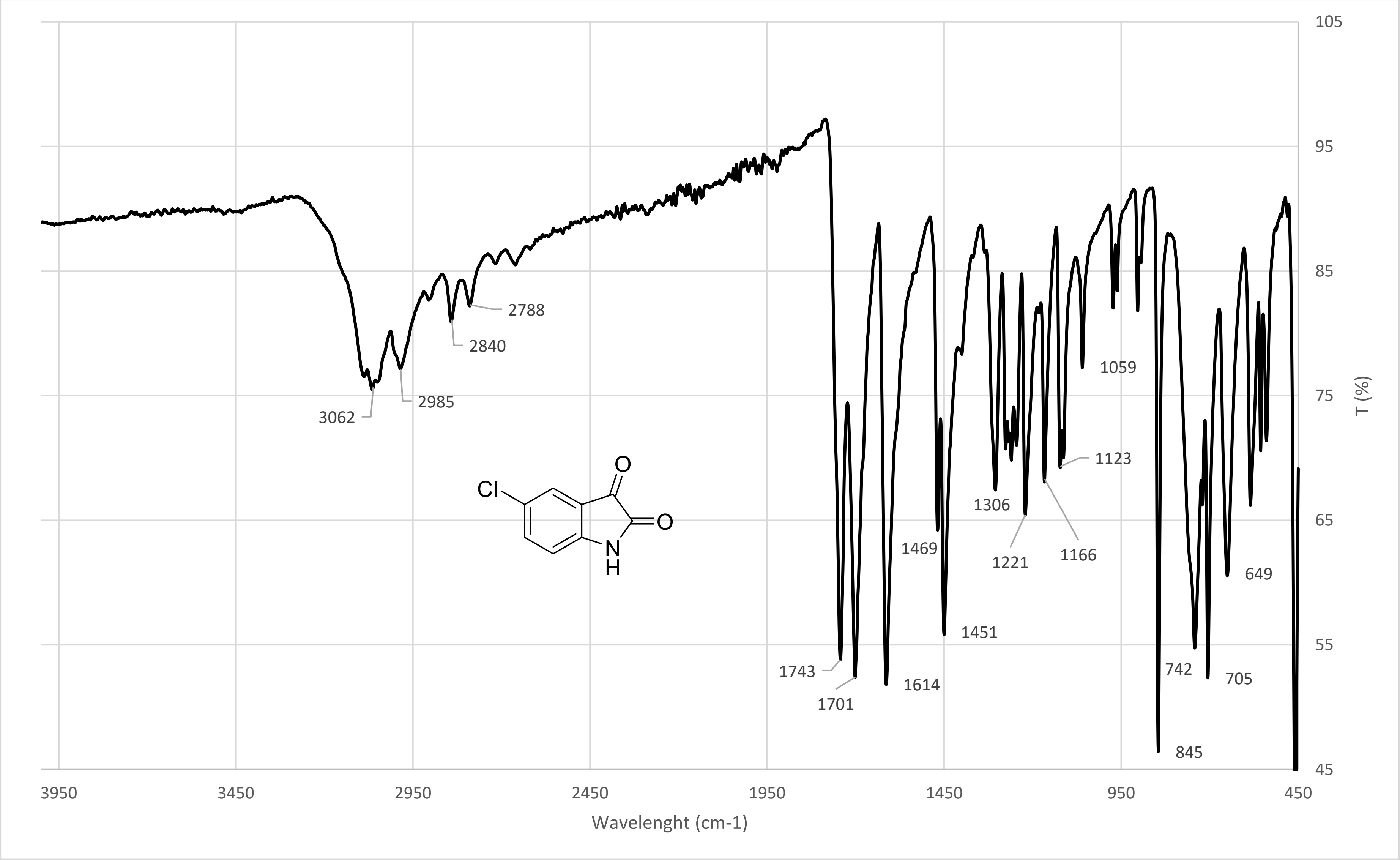

105
95
85
75
65
55
45
T (%)
3950
3450
2950
2450
1950
1450
950
450
Wavelenght (cm-1)
3062
2985
2840
2788
1743
1701
1614
1469
1451
1306
1221
1166
1123
1059
845
742
705
649
Cl
O
O
N
H

*1.22 Spectra of 5-methoxyisatin (**13e**)*

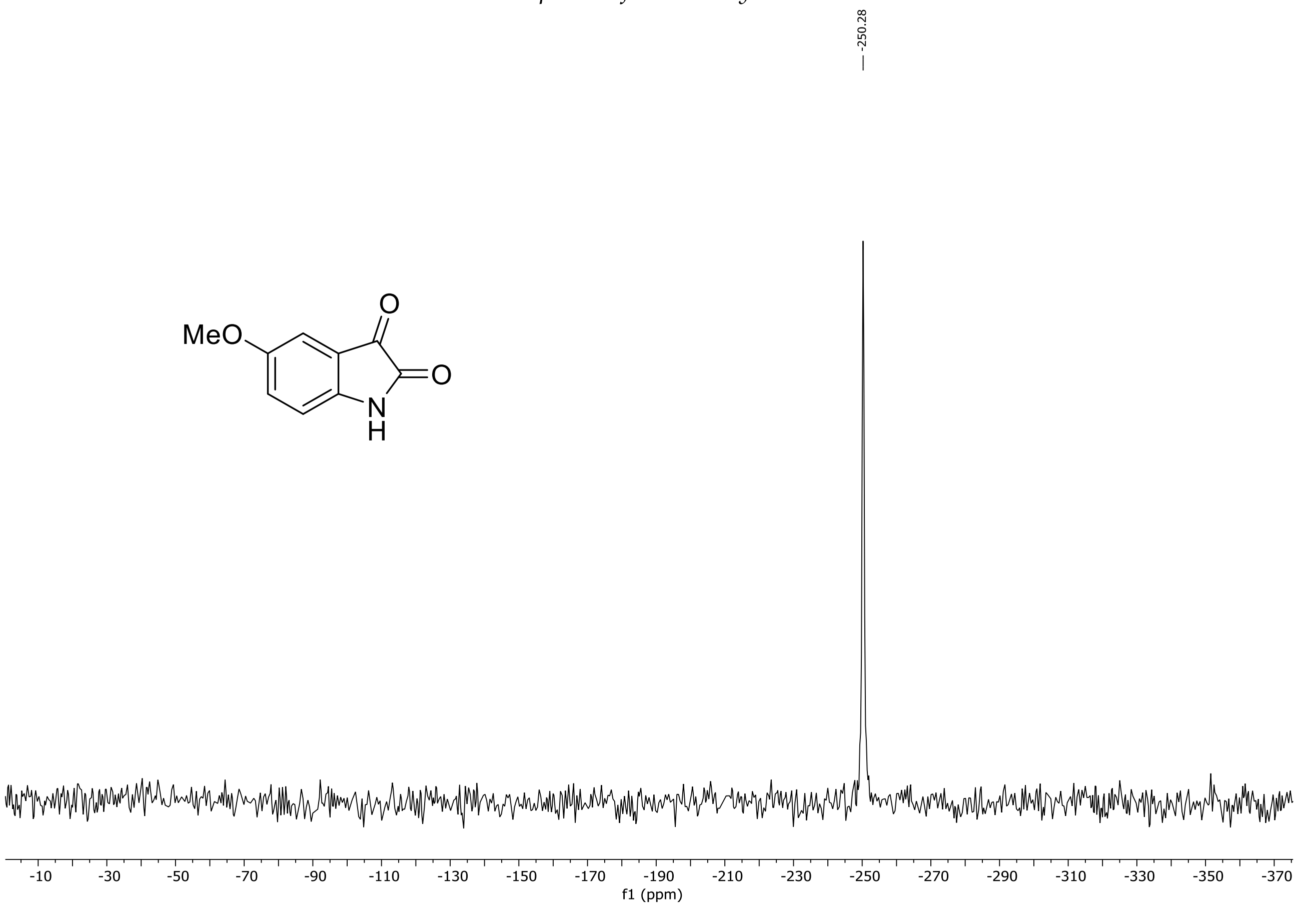

Solid-state nitrogen-15 NMR spectrum recorded with a 1 s recycle delay and 10 ms contact time.

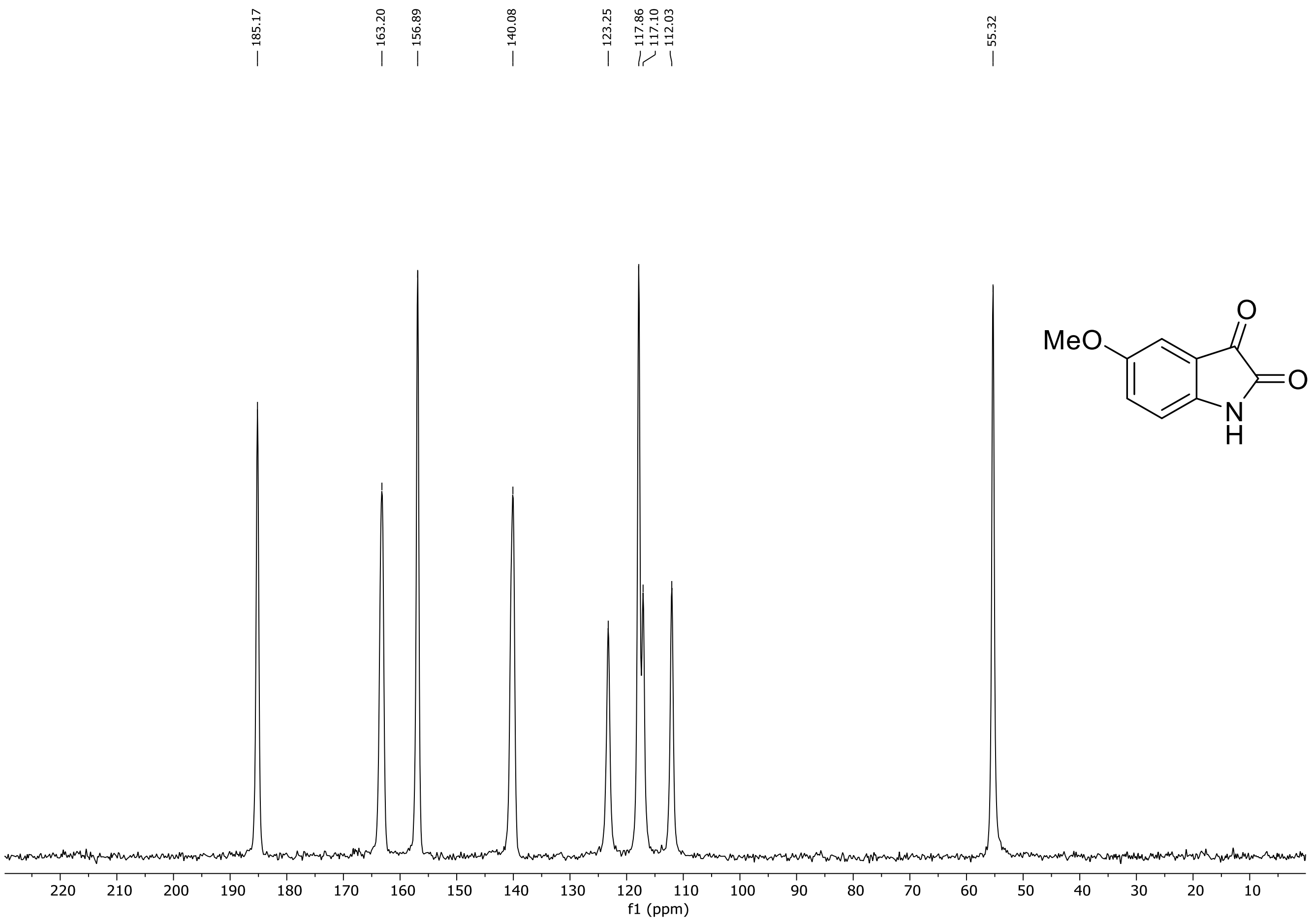

Solid-state carbon-13 NMR spectrum recorded with a 2 s recycle delay and 4 ms contact time.

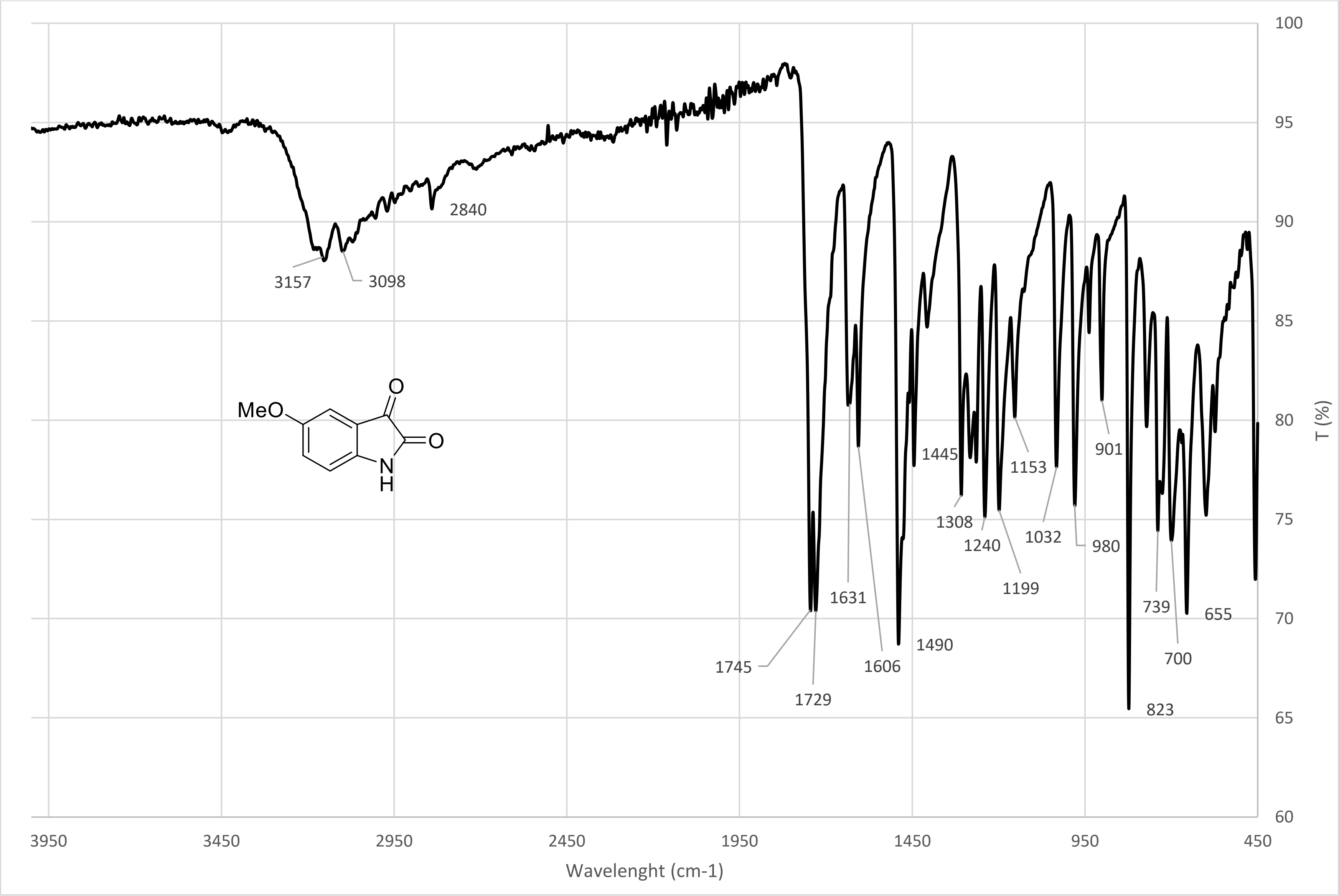

100
95
90
85
80
75
70
65
60
T (%)
3950
3450
2950
2450
1950
1450
950
450
Wavelenght (cm-1)
3157
3098
2840
MeO
O
O
N
H
1745
1729
1631
1606
1490
1445
1308
1240
1199
1153
1032
980
901
823
739
700
655

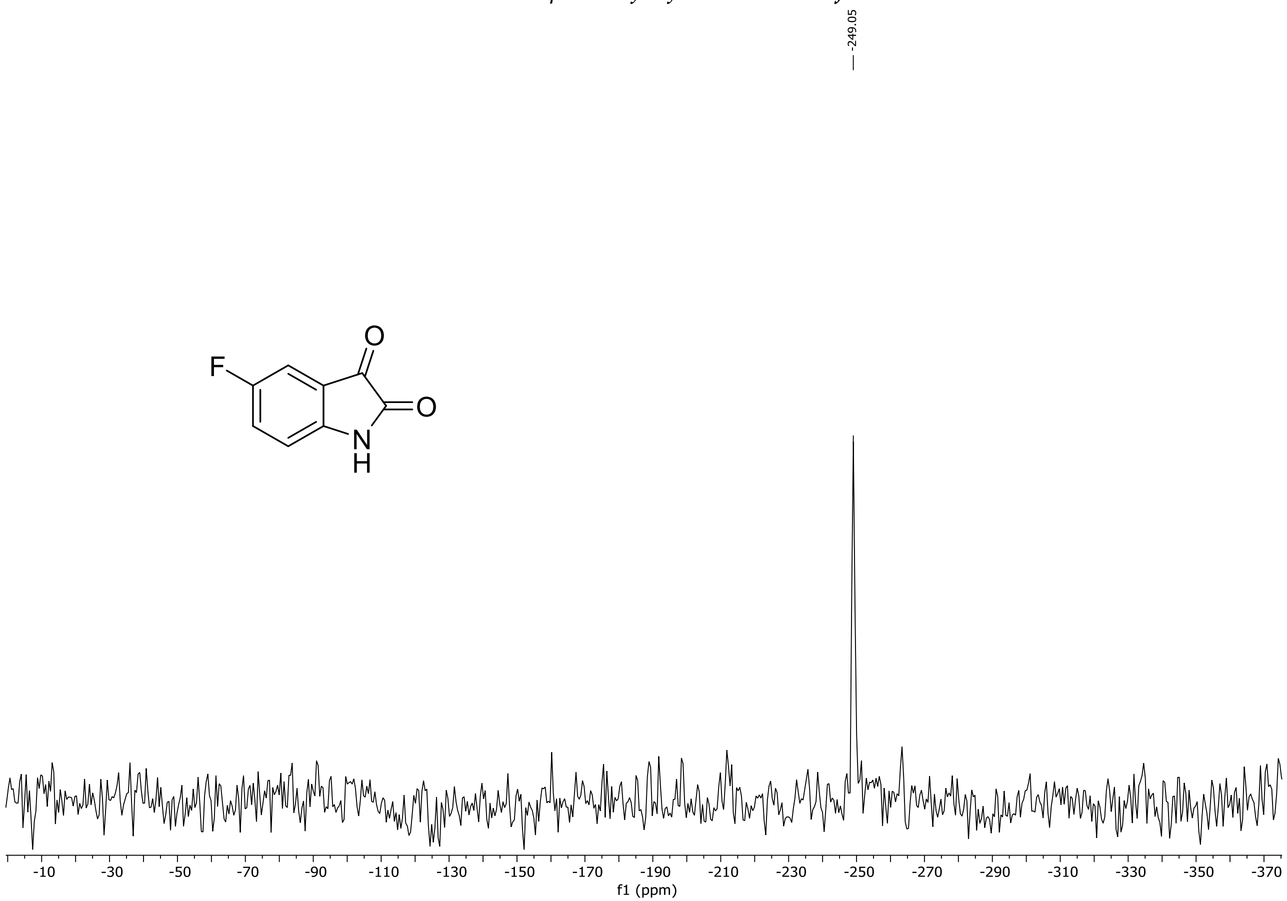

Solid-state nitrogen-15 NMR spectrum recorded with a 90 s recycle delay and 10 ms contact time.

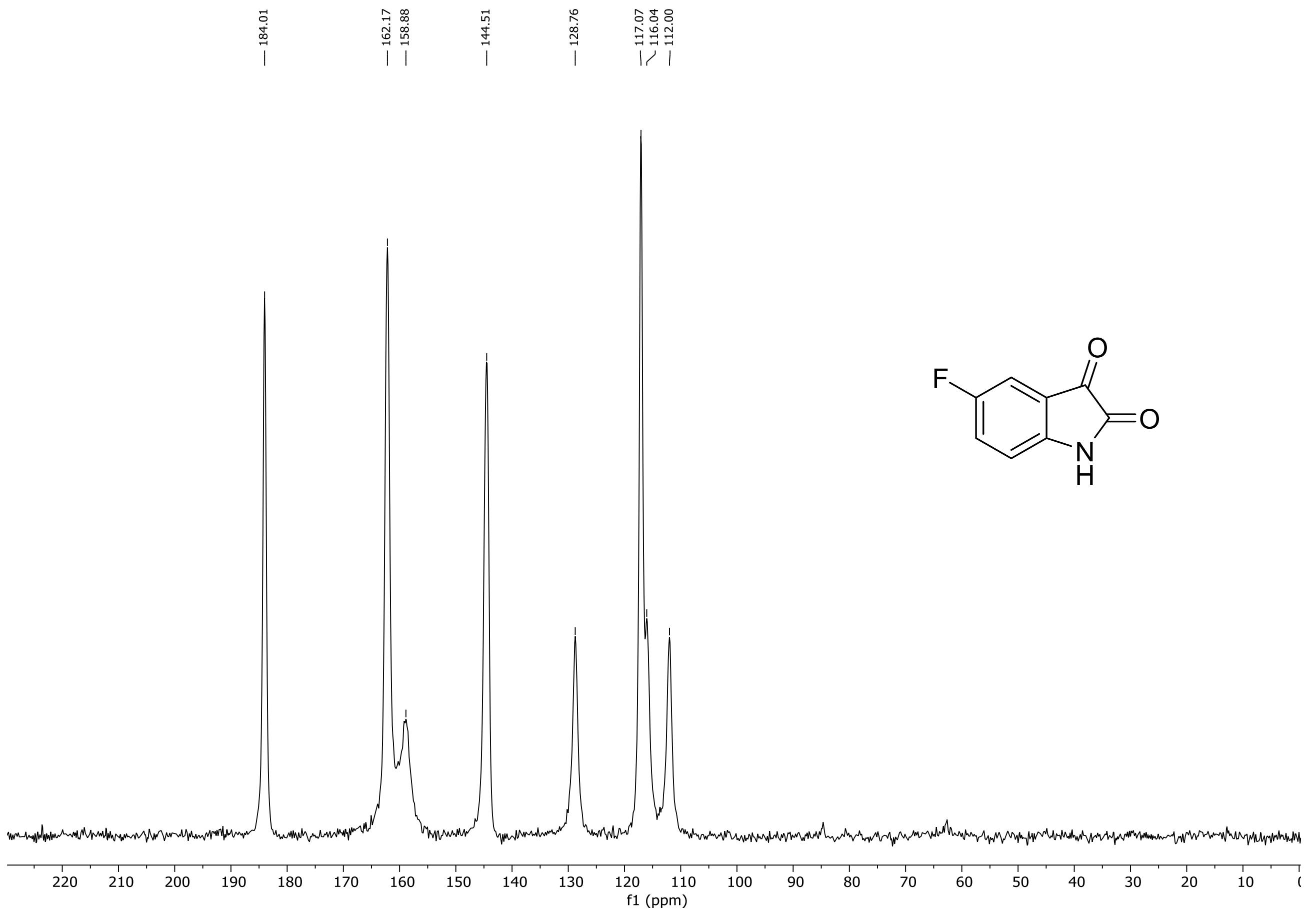

Solid-state carbon-13 NMR spectrum recorded with a 60 s recycle delay and 4 ms contact time.

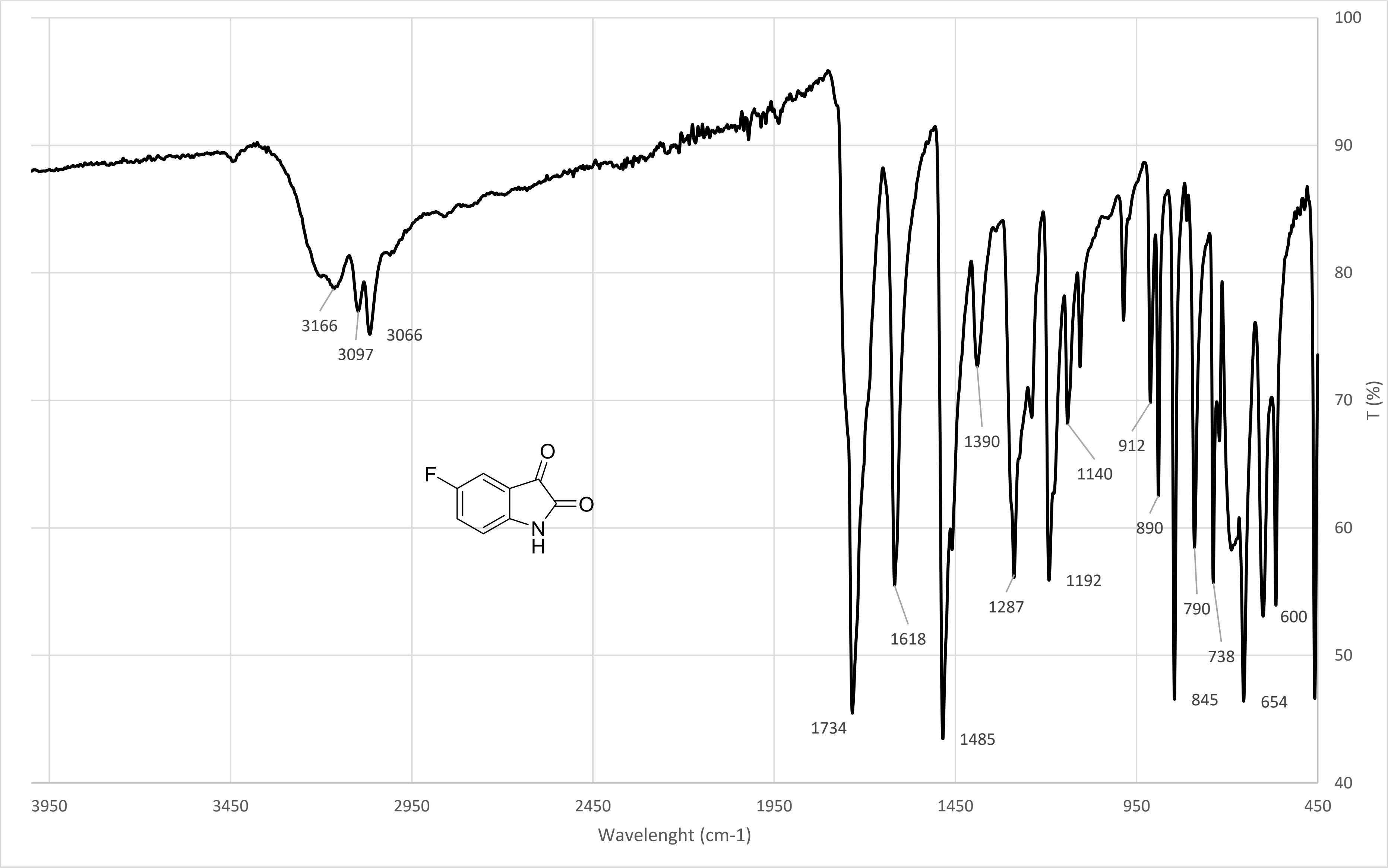

100
90
80
70
60
50
40
T (%)
3166
3097
3066
1734
1618
1485
1390
1287
1192
1140
912
890
845
790
738
654
600
F
O
O
N
H
3950
3450
2950
2450
1950
1450
950
450
Wavelenght (cm-1)

*1.24 Spectra of 7-trifluoromethylisatin (**13i**)*

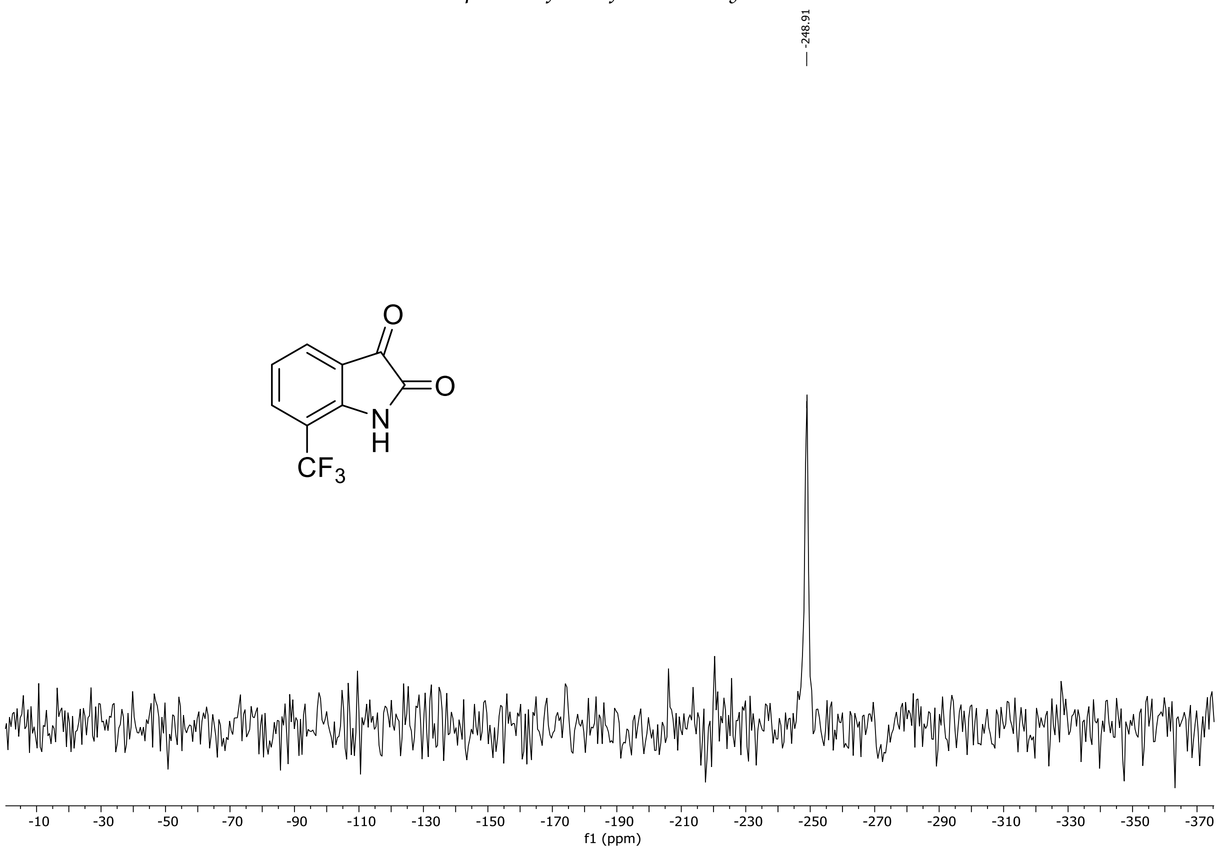

Solid-state nitrogen-15 NMR spectrum recorded with a 30 s recycle delay and 10 ms contact time.

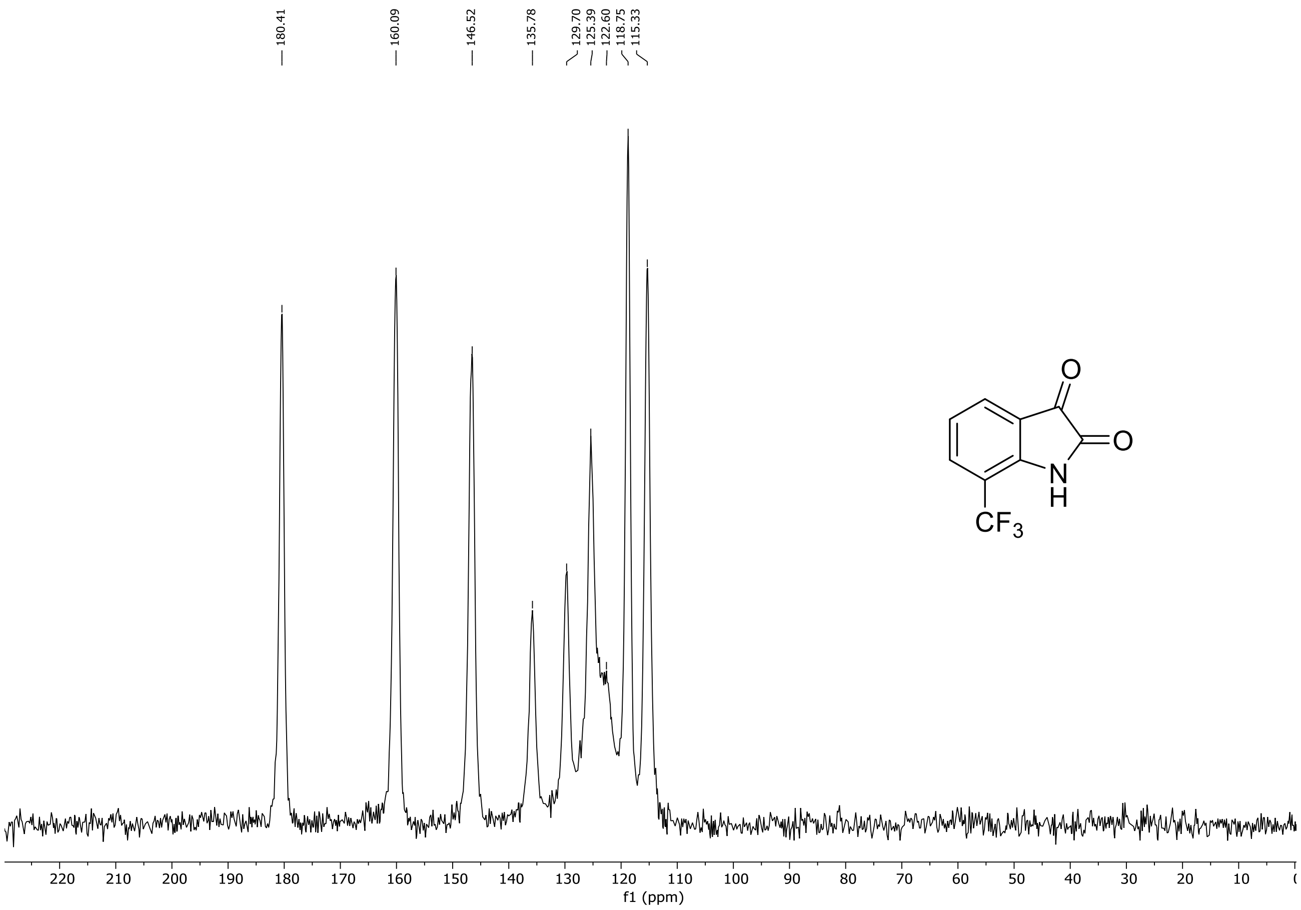

Solid-state carbon-13 NMR spectrum recorded with a 30 s recycle delay and 4 ms contact time.

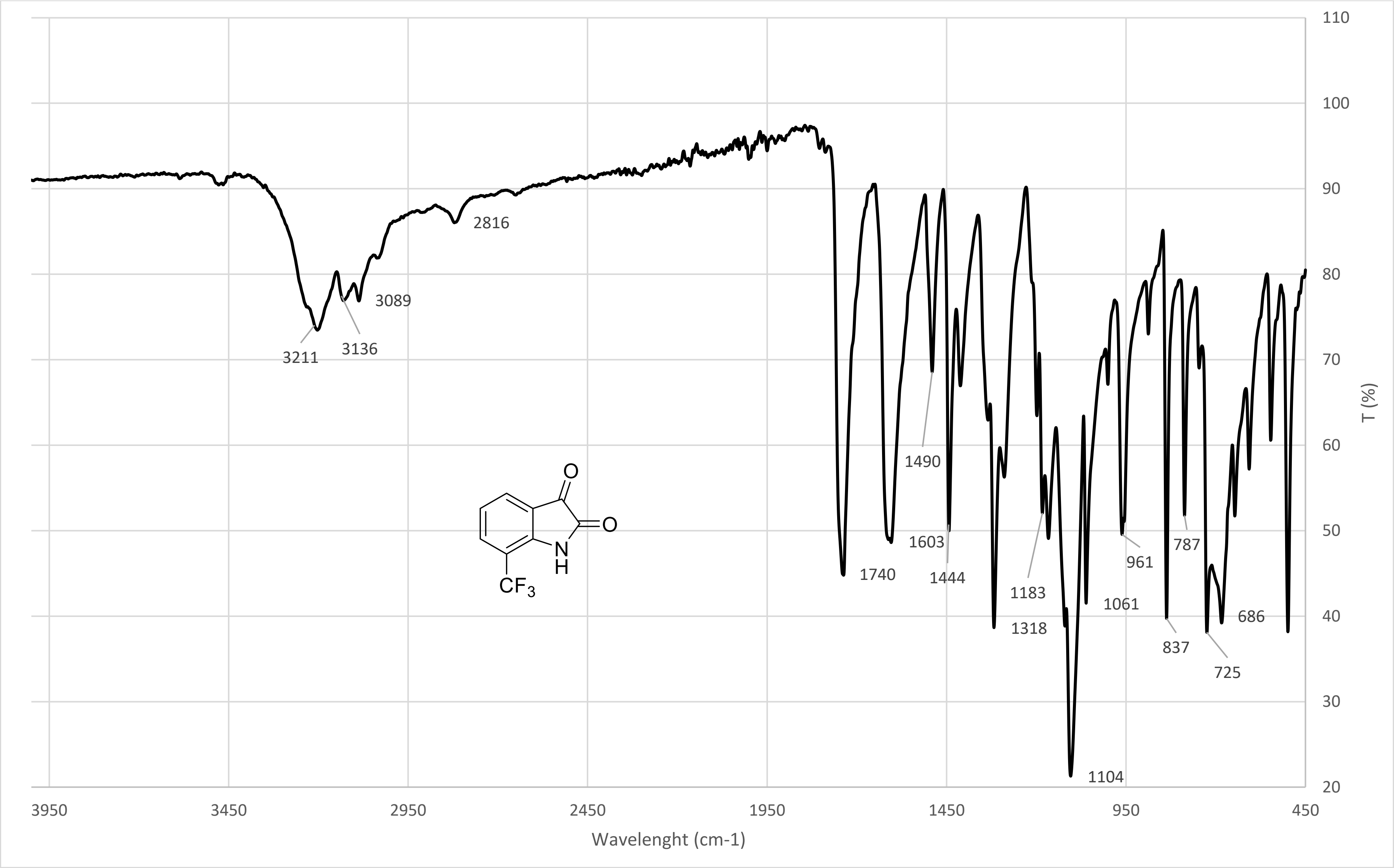
3211
3136
3089
2816
1740
1603
1490
1444
1318
1183
1104
1061
961
837
787
725
686
T (%)
Wavelenght (cm-1)
3950
3450
2950
2450
1950
1450
950
450
110
100
90
80
70
60
50
40
30
20
O
O
N
H
CF3

*1.25 Spectra of 7-fluroisatin (**13j**)*

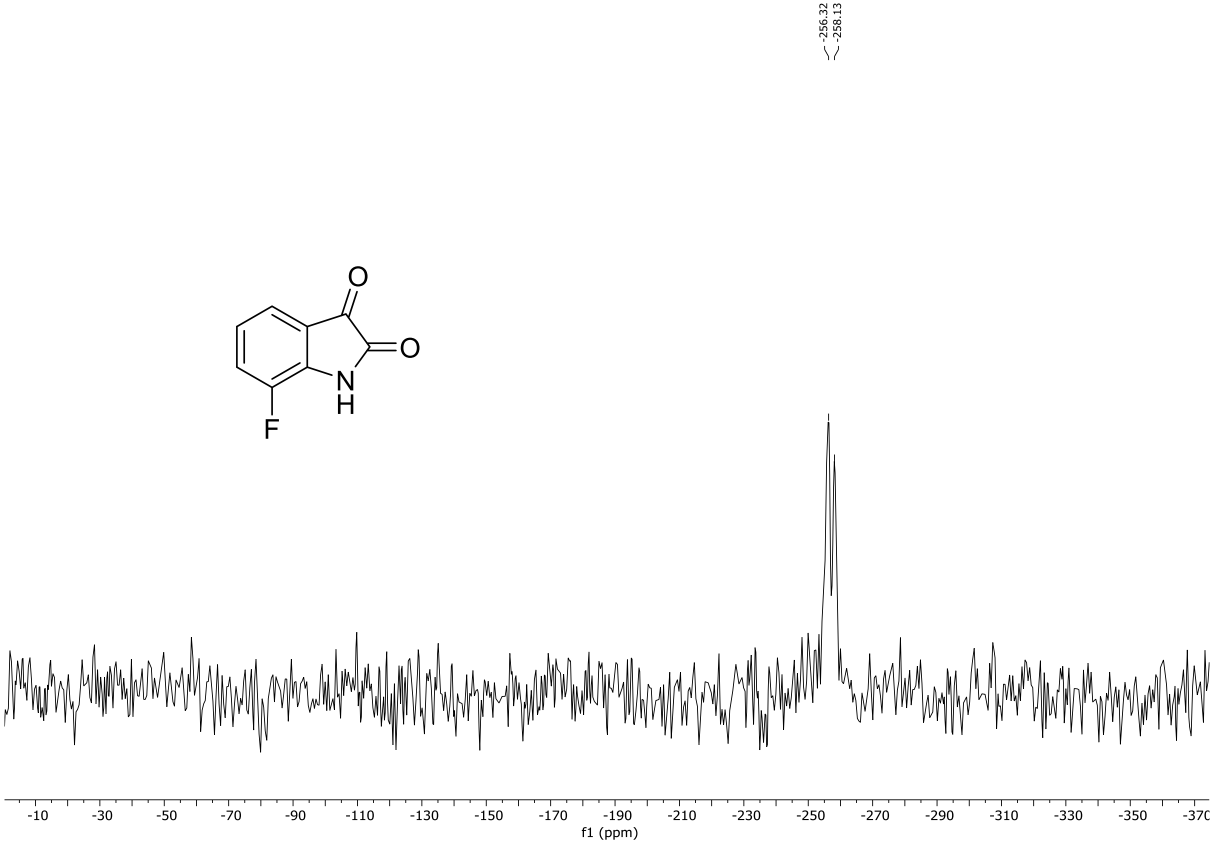

Solid-state nitrogen-15 NMR spectrum recorded with a 60 s recycle delay and 10 ms contact time.

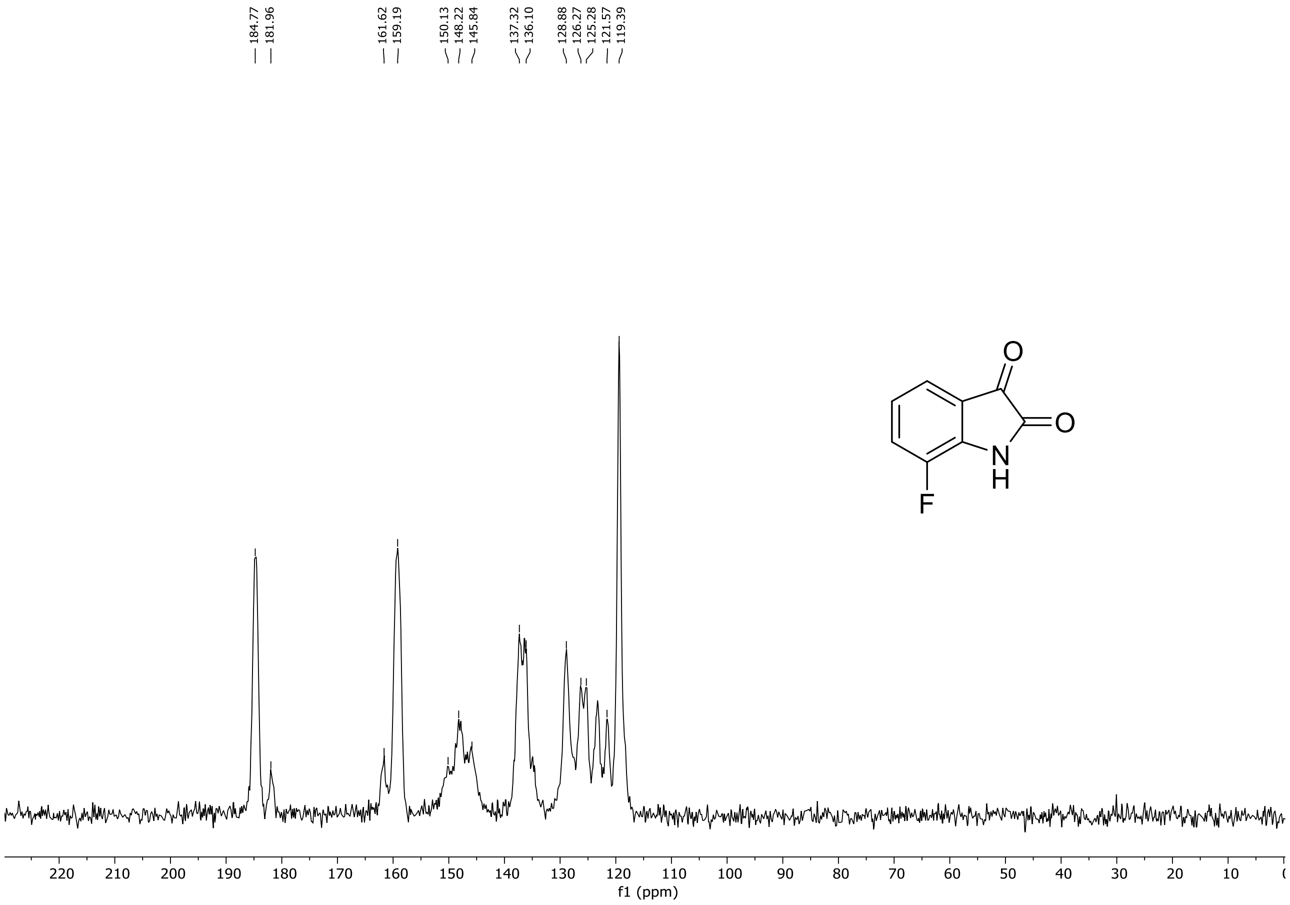

Solid-state carbon-13 NMR spectrum recorded with a 60 s recycle delay and 4 ms contact time.

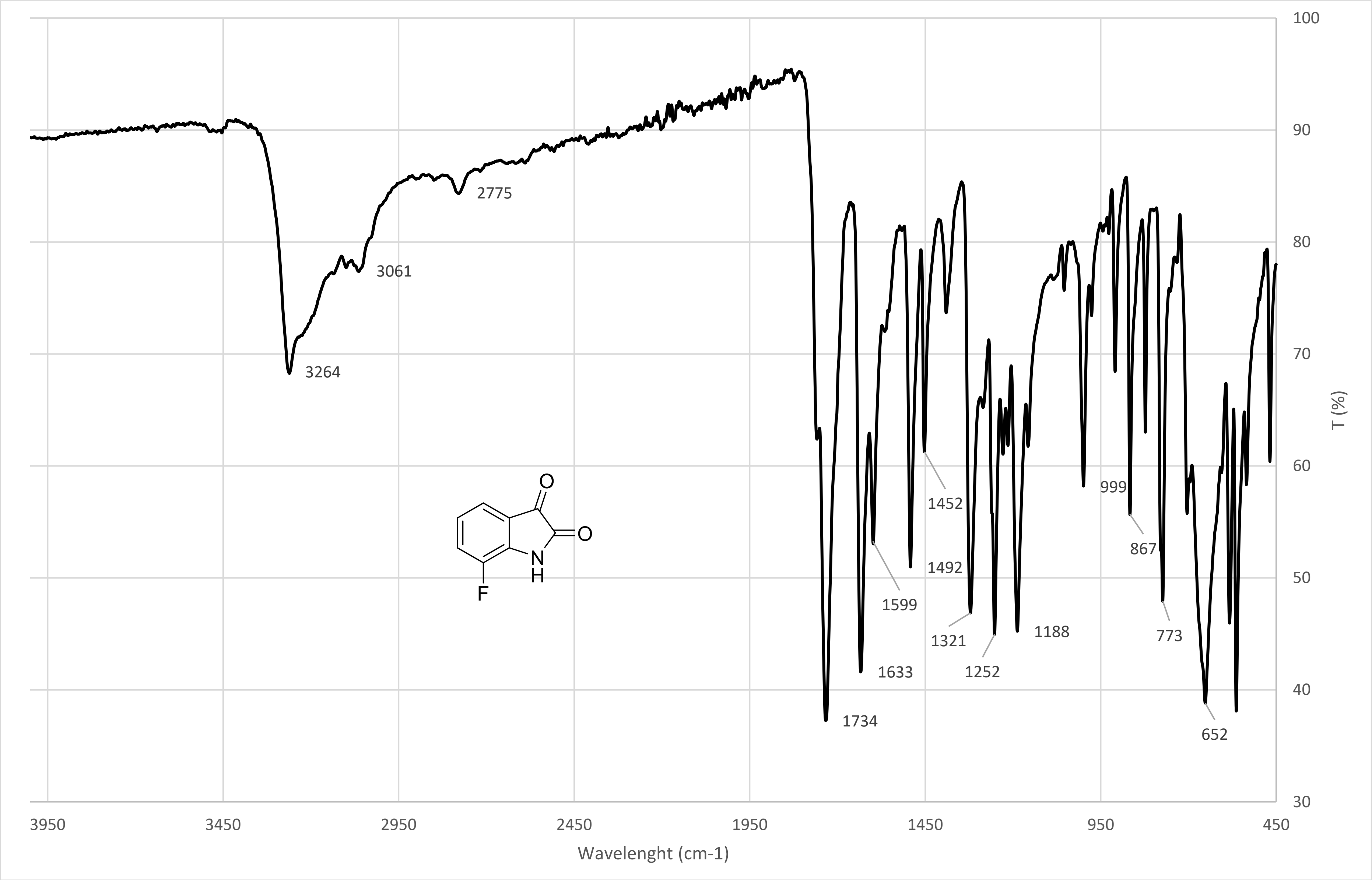
100
90
80
70
60
50
40
30
T (%)
3950
3450
2950
2450
1950
1450
950
450
Wavelenght (cm-1)
3264
3061
2775
1734
1633
1599
1492
1452
1321
1252
1188
999
867
773
652
O
O
N
H
F
